# Prosaposin activates the androgen receptor and potentiates resistance to endocrine treatment in breast cancer

**DOI:** 10.1186/s13058-015-0636-6

**Published:** 2015-09-04

**Authors:** Azlena Ali, Laura Creevey, Yuan Hao, Damian McCartan, Peadar O’Gaora, Arnold Hill, Leonie Young, Marie McIlroy

**Affiliations:** Endocrine Oncology Research, Department of Surgery, Royal College of Surgeons in Ireland, St. Stephens Green, Dublin 2, Ireland; Department of Surgery, Beaumont Hospital, Dublin 9, Ireland; School of Medicine and Medical Science, UCD Conway Institute, University College Dublin, Dublin, Ireland

## Abstract

**Introduction:**

*HOX* genes play vital roles in growth and development, however, atypical redeployment of these genes is often associated with steroidal adaptability in endocrine cancers. We previously identified HOXC11 to be an indicator of poor response to hormonal therapy in breast cancer. In this study we aimed to elucidate genes regulated by HOXC11 in the endocrine resistant setting.

**Methods:**

RNA-sequencing paired with transcription factor motif-mapping was utilised to identify putative HOXC11 target genes in endocrine resistant breast cancer. Validation and functional evaluation of the target gene, prosaposin (PSAP), was performed in a panel of endocrine sensitive and resistant breast cancer cell lines. The clinical significance of this finding was explored in clinical cohorts at both mRNA and protein level.

**Results:**

PSAP was shown to be regulated by HOXC11 in both tamoxifen and aromatase inhibitor (AI) resistant cell lines. Transcript levels of HOXC11 and PSAP correlated strongly in samples of primary breast tumours (r = 0.7692, n = 51). PSAP has previously been reported to activate androgen receptor (AR) in prostate cancer cells. In a panel of breast cancer cell lines it was shown that endocrine resistant cells exhibit innately elevated levels of AR compared to their endocrine sensitive counterparts. Here, we demonstrate that stimulation with PSAP can drive AR recruitment to a hormone response element (HRE) in AI resistant breast cancer cells. Functionally, PSAP promotes cell migration and invasion only in AI resistant cells and not in their endocrine sensitive counterparts. In a cohort of breast cancer patients (n = 34), elevated serum levels of PSAP were found to associate significantly with poor response to endocrine treatment (p = 0.04). Meta-analysis of combined PSAP and AR mRNA are indicative of poor disease-free survival in endocrine treated breast cancer patients (hazard ratio (HR): 2.2, P = 0.0003, n = 661).

**Conclusion:**

The HOXC11 target gene, PSAP, is an AR activator which facilitates adaptation to a more invasive phenotype *in vitro*. These findings have particular relevance to the development of resistance to AI therapy which is an emerging clinical issue. PSAP is a secreted biomarker which has potential in identifying patients failing to exhibit sustained response to hormonal treatment.

**Electronic supplementary material:**

The online version of this article (doi:10.1186/s13058-015-0636-6) contains supplementary material, which is available to authorized users.

## Introduction

Breast cancer has the highest incidence and second highest mortality rate among cancers in women worldwide [1]. The median age of diagnosis for breast cancer is 61 years and the majority of patients are postmenopausal at time of diagnosis. Endocrine therapy has been the mainstay for patients with hormone receptor-positive breast cancer (approximately 75 %) since the inception of the steroid receptor modulator, tamoxifen, in the latter part of the 20th century. One of the major advances in breast cancer therapy in the past decade has been the widespread endorsement of aromatase inhibitors (AIs) as first-line therapy for postmenopausal patients with hormone receptor-positive breast cancer. AIs work by abrogating the activity of the enzyme aromatase (Cyp 19), which converts circulating androgens into estrogen within different body compartments including mammary adipose tissue [2–4]. Clinical trials into the efficacy and side-effects of these drugs in comparison to tamoxifen have heralded them as the main choice of adjuvant endocrine therapy for postmenopausal breast cancer [5]. Despite the huge advances in the treatment and management of breast cancer the development of drug resistance remains an unresolved problem. For hormone receptor-positive breast cancer, drug resistance occurs in approximately 25 % of cases, which accounts for approximately 50,000 breast cancer recurrences/annum in the US alone [6]. In some studies the development of resistance to endocrine therapy is estimated to be as high as 30−60 % [7]. This may be due in part to adaptive hypersensitivity of the intact estrogen receptor (ER) [5], selective co-activator enhancement [8], or a shift to growth-factor pathway-dependent cell growth which is well-known to contribute to endocrine treatment failure [9, 10]. Approximately 75 % of breast cancers also express androgen receptor, which includes a proportion of triple-negative tumours [11, 12]. With regards to the role of androgen signalling and breast cancer survival there is conflicting evidence. In ER-positive tumours it is reported that androgen receptor (AR) expression is beneficial and it is suggested that it may compete for ER binding sites on the DNA, thereby blocking estrogen-stimulated transcription of pro-proliferative genes [13]. Other reports however claim that high circulating levels of androgens are associated with increased breast cancer risk in both the premenopausal and postmenopausal setting [14] and that elevated levels of AR are pro-metastatic [15]. More recent studies have highlighted the tumour promotional effects of the AR particularly in the event of estrogen disruption [16–20].

Previous work from this laboratory investigating endocrine resistance in breast cancer has shown the developmental transcription factor HOXC11 to be a strong predictor of metastasis and poor disease-free survival (DFS), independent of receptor status, tumour size, nodal status and grade [21]. Homeobox genes encode a family of 39 proteins of which HOXC11 is a member. These proteins act as developmental transcription factors involved in growth and differentiation [22, 23]. They play essential roles in body patterning and spatial identity, hence they are akin to a form of cellular global positioning system [24]. Maintenance of *HOX* gene expression patterns are under complex epigenetic regulation. The homeobox transcription factors are known to be regulated by steroids during embryogenesis; however, there is a growing body of evidence to suggest that these genes are also architects of steroidal regulation in endocrine tumours [21, 25–27]. Studies by Norris et al. [28] demonstrate that HOXB13 can interact with the AR to alternately suppress or activate AR-responsive genes in a promoter-dependent manner. Moreover, recent studies into the upregulation of the HOXC locus in prostate cancer have demonstrated that its ability to modulate androgen signalling is due to the abrogation of coactivator recruitment to direct androgen target genes [25]. Thus, there is rapidly accumulating evidence to suggest that HOX genes and in particular HOXC genes are key players in modulating steroid signalling in endocrine tumours.

To further our understanding of HOXC11 and the role it plays in the development of endocrine resistance and steroidal adaptability we undertook an RNA-seq experiment to identify HOXC11 target genes in resistant breast cancer. We identified 1,919 genes, and conducted motif mapping to identify potential direct target genes of the transcription factor HOXC11. Analysis of the target genes identified a common novel motif with significant similarity to an AR response element. From these studies we identified prosaposin (PSAP) as a HOXC11 regulated gene. PSAP is a purported AR activator associated with metastatic potential in a number of neoplasms [29–31]. This study supports the hypothesis that expression of HOXC11 and the subsequent secretion of PSAP can expedite endocrine resistance to aromatase inhibitor therapy via tumour promotional activation of the AR.

## Methods

### Cell culture

Endocrine-sensitive MCF-7 (American Type Culture Collection (ATCC) Virginia, USA ) and tamoxifen-resistant LY2 cells (kind gift from R. Clarke, Georgetown, DC, USA) were grown as previously described [21]. MCF7-derived AI-sensitive cells (Aro) were developed in house. MCF7 Aro-derived letrozole-resistant cells (LetR) and anastrozole-resistant cells (AnaR) were created by long-term treatment of Aro with letrozole (Novartis, Basel, Switzerland) or anastrozole (AstraZeneca, Macclesfield, UK) [21]. LY2, LetR and AnaR cells were utilised to model endocrine resistance developed through long-term adaptation to hormonal therapy. MDA-MB-453, SKBR3 and LNCaP cells were acquired from ATCC and maintained as recommended. Cells were maintained in steroid-depleted medium for 72 hours before treatment with hormones. All cells were incubated at 37 °C under 5 % CO_2_ in a humidified incubator. All in-house cells were authenticated and are routinely verified as endocrine resistant.

### siRNA transfection

HOXC11 was silenced by transient transfection using an experimentally verified pool of siRNA (Flexitube, Qiagen, Manchester, UK) as previously described [21]. All transfections were carried out using Lipofectamine 2000 transfection reagent according to manufacturer’s instructions (Invitrogen, Thermo Fisher, Warrington, UK) and a non-targeting siRNA negative control (Ambion, Thermo Fisher, Warrington, UK) was used as a control for all siRNA experiments.

### RNA extraction, library preparation and RNA sequencing (RNA-seq)

To assess the global effects of HOXC11 in endocrine-resistant breast cancer cells we performed RNA-seq on LY2 cells, which were transfected with either siRNA targeting HOXC11 (siRNA-HOXC11) or a scrambled negative control siRNA (scrambled) in the presence of tamoxifen (10^−8^ M). Knockdown was verified by Taq-man quantitative reverse transcription PCR (qRT-PCR) prior to library preparation. RNA (10 μg) was extracted using an Oligotex mRNA kit (Qiagen) as per manufacturer’s instructions (n = 4). RNA was reverse transcribed followed by mRNA library preparation and sequencing based on a protocol outlined by Wilhelm *et al*. [32]. Sequencing was performed on an Illumina Genome Analyzer II (GAII) (54 million reads per sample on average). Four independent biological libraries were prepared for each sample to facilitate the detection of expression and estimation of variance. Multiplexing was achieved using barcoding adapters designed in house. After quality checks two of the replicates per sample group were then subjected to further downstream analysis.

Short reads of 36 bp in length were aligned to human reference genome (UCSC hg19, Ensembl GRCh37 release 64) using the splice-aware aligner Tophat [33] allowing up to 50 multiple mapping locations and no more than 2 mismatches across each read. Differential expression genes (DEGs) were detected by using the Cufflinks/Cuffdiff program (v v1.0.2) [34].

### Estrogen response element (ERE) and androgen response element (ARE) motif analysis

The AR and ER position frequency matrices (PFM) were downloaded from the JASPAR database [35]. The PFM was then converted to positive weight matrices (PWM) using the MEME suite programme [36]. A 400-bp-sized window surrounding starting sites of HOXC11 target genes was selected for motif searching. FIMO [37], a MEME suite programme, was used to identify the AR/ER motifs near the HOXC11 target gene start site with a *p* value significance cutoff set at 0.001. Genes having at least one significant hit were kept, and for genes with multiple significant hits, only the best one is reported.

### HOXC11 motif analysis

The global mapping of the HOXC11 motif was performed using the TFfind programme [38]. The HOXC11 motif frequency matrix (PFM) and representing logo were identified from the UniPROBE database [39]. The first bp and the last 3 bp of this motif were deemed to have low information content and removed from further consideration, resulting in a full motif of 12 bp (consensus sequence: AANGTCGTAAAA) being employed for binding site discovery. Mapping was carried out in the promoter region (5000 bp upstream of TSSs) of all annotated human genes (UCSC hg19, Ensembl GRCh37 release 64). Resultant hits that met the predefined cutoff (≥0.9; 1 denotes a perfect match) were kept and associated with corresponding gene symbols based on the UCSC kgXref table. Overlaps between the DEGs from the siRNA-HOXC11 RNA-seq data and those containing HOXC11 motif in their promoters were reported.

### Chromatin immunoprecipitation

LY2 cells were treated with tamoxifen (10^−8^ M), estrogen (10^−8^ M) or vehicle. LetR cells were treated with androstenedione (Andro, 10^−7^ M), estrogen (10^−8^ M) or vehicle. Chromatin immunoprecipitation (ChIP) was performed as previously described [21]. Mouse anti-HOXC11 (6 μg) (Santa Cruz Biotechnology (SCBT), Texas, USA) was added to the supernatant fraction and incubated overnight at 4 °C with rotation. Proteins were un-crosslinked, and primers were used to amplify the DNA −581 to −116 of the PSAP proximal promoter that harbors a HOXC11 binding site and a hormone response element (HRE). PSAP proximal promoter primers were forward: CCCGCTACTACAATGGGCTA, and reverse: GGGGAGGAGTGAGGAAGAAC. Distal non-promoter control primers were forward: TGGTGAGGTTGTATCCACGA, and reverse: CCACTCATGCAATGACCGTA.

### PSAP ELISA

A commercially available PSAP ELISA (Cusabio, Stratech, Suffolk, UK) was used to assess serum levels of secreted PSAP in conditioned medium from breast cancer cell lines *in vitro* and also in blood serum from consenting patients with breast cancer (n = 34) (see “Clinical Samples” for more detail).

### Western blotting

Protein from breast cancer cell lines were lysed, electrophoresed, and immunoblotted with a rabbit anti-human AR (1:1000 dilution) (sc-816, SCBT), β-actin loading control (Sigma Aldrich, UK) and a corresponding horseradish peroxidase-conjugated secondary antibody (Dako, Den).

### HOXC11 transfection

A vector construct pCMV.SPORT-HOXC11 (Life technologies, Thermo Fisher, UK) was used to transiently overexpress HOXC11 in MCF7 cells. MCF7 were seeded at 2.5 × 10^5^ cells in a 6-well plate and incubated overnight. Lipofectamine 2000 (Life Technologies) was used to transfect the cells with pCMV.SPORT-HOXC11 versus empty vector (2 μg) according to the manufacturer’s instructions.

### TransAM AR assay

The TransAM assay (Active Motif, California, USA) was modified for use with an AR rabbit anti-human antibody (sc-816, SCBT). Optimal nuclear lysate and antibody concentrations were determined using steroid-dependent LNCaP prostate cancer cell nuclear lysate as a positive control (Additional file 1: Figures S1A and B). LetR breast cancer cells were cultured in the presence of metribolone, a synthetic androgen (R1881, Sigma Aldrich) (10 nmol/L) or recombinant human PSAP (rhPSAP) (10 ng/ml) for 1 hour. Cells were pelleted and nuclear protein isolated using a NE-PER kit (Pierce, Thermo Fisher). The assay was then performed according to manufacturer’s instructions using LetR nuclear cell lysate (20 μl) and AR antibody at a 1:250 dilution (Additional file 1: Figure S1B). On completion of the assay the plate was read at an absorbance of 450 nm (0.1sec) using a Victor2 plate reader (Perkin Elmer, Dublin, Ire).

### Treatment of cells in vitro with rhPSAP

Lyophilized rhPSAP protein (Abnova, Taiwan) was reconstituted in storage buffer (50 mM Tris-HCI, 10 mM reduced Glutathione, pH = 8.0). LetR cells were steroid-depleted for 72 hours prior to the addition of recombinant PSAP protein (10 ng/ml). Cells were cultured for a further hour (TransAM) or for 24 hours (protein analysis) under standard conditions, before the cell monolayer was washed with PBS, trypsinised and the cells pelleted for further analysis.

### Nuclear translocation assay

Uncoated glass microscope slides (BDH Laboratory supplies, UK) were cleaned with 100 % ethanol, air-dried and placed in sterile 6-well tissue culture dishes. MCF7 and LetR cells were seeded at 6 × 10^2^ onto a coverslip in steroid-depleted media for 72 hours prior to treatment. For individual treatments, rhPSAP (10 ng/ml) and enzalutamide (Enza, 10 μM, Selleckchem, Stratatech) were added to the cells for 3 hours. For dual rhPSAP and Enza treatment, cells were pretreated with Enza (10 μM) for 2 hours and then co-treated with rhPSAP (10 ng/ml) in the presence of Enza for a further hour (3 hours total treatment time). Cells were washed in PBS and fixed in methanol for 10 minutes and permeabilised with 0.1 % triton X-100. Samples were then blocked with 10 % goat serum for 1 hour at room temperature (RT) and incubated with an antibody against AR (N20, sc-815 1:50; SCBT) in 10 % human serum for 90 minutes at RT. Samples were incubated with secondary antibody anti-rabbit Alexa Fluor 488 (1:200) (Thermo Fisher) in 10 % human serum for 1 hour at RT. The nuclei were stained with 4′,6-diamidino-2-phenylindole (1 μg/ml) for 1 minute. Samples washed with dH_2_O and coverslips containing treated samples were mounted onto slides using fluorescent mounting media (Dako). Cells were visualised by fluorescent microscopy using the CellSens Olympus software. The nuclear translocation of AR (the intensity of AR signal within the nucleus) was quantified in a minimum of 50 cells using ImageJ software.

### In vitro cell migration assay

Uncoated glass microscope slides were cleaned with 100 % ethanol, air-dried and placed in sterile 6-well tissue culture dishes. Cells were plated (LetR 3 × 10^5^ cells/2ml growth medium per well, MCF7 2.5 × 10^5^ cells/2ml growth medium per well) and allowed to adhere for 24 hours at 37 °C under standard conditions. Cell monolayers were wounded using the pointed edge of a 20−200-μl yellow pipette tip to score laterally and through the longest length of the cell monolayer. All slides were wounded aseptically at the same time. Cells were treated with 0.1, 1.0, and 10.0 ng/ml rhPSAP and vehicle (Tris–HCl 5 nM). Five images were captured along the length of the scratch at 0, 6 and 24 hours. Five measurements were made within each image using CellSens Entry 1.8 software. Each treatment group consisted of three slides and the entire experiment was repeated in triplicate.

### Transwell invasion assay

Twenty-four-well Biocoat matrigel invasion chambers (Corning, USA) were prepared as per manufacturer’s instructions: 5 × 10^4^ cells/500 μl (either MCF7 or LetR) were seeded in serum-free medium into the upper chamber of the insert, and 10 % serum medium was added to the lower chamber to act as a chemoattractant. rhPSAP (10 ng/ml) was added to the upper chambers versus Tris–HCl (5 nM) control. Transwell plates were incubated for 48 hours under normal conditions, 37 °C, 5 % CO_2_. If cells had been transfected with siRNA the experiment was extended to 60 hours. Inserts were then washed with PBS (×2) and cells in the upper chamber removed using a cotton tip (Johnston & Johnston, Ire); cells remaining in the underside of the insert were then fixed in methanol (10 minutes), and 0.5 % crystal violet (Cruinn, Ire) was added to the lower chamber to visualize cells that had invaded across the membrane. Inserts were washed in dH_2_O (×2). Representative images (×4 magnification) (CellSens Olympus software) were then evaluated using Image J software to quantify the results. A minimum of five fields of view were evaluated per well.

### MTS assay

LetR cells were steroid-depleted for 72 hours and then seeded into a 24-well plate prior to the addition of bicalutamide (Bica) (1 μM) (Sigma Aldrich) or vehicle (dimethyl sulfoxide DMSO; 0.01 %) to regular growth medium. MTS reagent (Sigma Aldrich) was added after 2, 3 and 4 days respectively and the resultant colorimetric outputs analyzed by measuring the absorbance at 490nm using a spectrophotometer (Perkin Elmer).

### Colony forming assay

LetR cells were steroid depleted for 72 hours prior to being seeded into 6-well plates at a density of 5 × 10^2^ cells per well. The cells were treated with vehicle (DMSO; 0.01 %) or Bica (1 μM) and androstenedione (100 nmol/L), and each plate was incubated over 4, 8, or 12 days. Media containing treatments were changed every 48 hours up until the end of each time point. After each time point was reached, cells were washed with PBS, fixed with methanol-glacial acetic acid and stained with 0.5 % crystal violet solution (Cruinn).

### Clinical samples

Preoperative blood serum was collected from patients undergoing surgery for the resection of a clinically diagnosed primary breast tumour: 7-ml blood samples were taken in non-heparinised tubes, allowed to clot at RT for approximately 30 minutes and separated in a cooled (4 °C) centrifuge at 2,000 g for 10 minutes. Serum was stored as 0.5-ml aliquots/cryovials and placed in a freezer at −80 °C within 2 hours of being obtained. All patients provided written consent and are currently enrolled on a clinical trial (ICORG – 09–07), ethical approval was sought and granted by the appropriate Research (Medical Ethics) Committees at Beaumont and Waterford Regional Hospital (Ire). Clinical pathology data including receptor status, tumour grade, nodal status, and endocrine therapy have been collated. The median follow-up period for the cohort was 35 months.

### HOXC11 and PSAP mRNA correlation in clinical datasets

The Cancer Genome Atlas (TCGA) breast cancer RNA-seq dataset was evaluated to determine correlation between PSAP and HOXC11 mRNA in a supplementary clinical cohort. The TCGA data were classified based on breast cancer subtypes (luminal A, luminal B, human epidermal growth factor receptor (Her)2 over-expressing and basal) and each subtype was assessed for correlation (Spearman) between PSAP and HOXC11. In addition, samples with high AR expression (upper quartile) were also selected and the Spearman’s correlation between HOXC11 and PSAP calculated for this subgroup. A hazard ratio (HR) curve was also generated for PSAP based on these data.

### Meta-analysis of PSAP mRNA expression and breast cancer patient survival

BreastMark [40] is an algorithm that enables the identification of subsets of gene transcript/miRNAs that are associated with disease progression in breast cancer and its subtypes. High levels of PSAP and/or AR mRNA were evaluated in endocrine-treated datasets.

### Statistical analysis

Graphpad Prism was used for the majority of statistical analysis. Univariate analysis was conducted using Fisher exact test for categorical variables. A *p* value of less than 0.05 was considered to be significant. Significance for quantitative data was evaluated by repeated measures one-way analysis of variance (ANOVA) for treatments over time and the unpaired, two-tailed Student *t* test was used to compare means. Spearman’s rank coefficient was used to determine correlation between variables.

### Data accession code

RNA-seq data are available from the Gene Expression Omnibus (GEO) database (GSE71139).

## Results

### Prosaposin (PSAP) was identified as a putative HOXC11 target gene from RNA-sequencing of endocrine-resistant breast cancer cells in which HOXC11 was knocked down

**Fig. 1:**
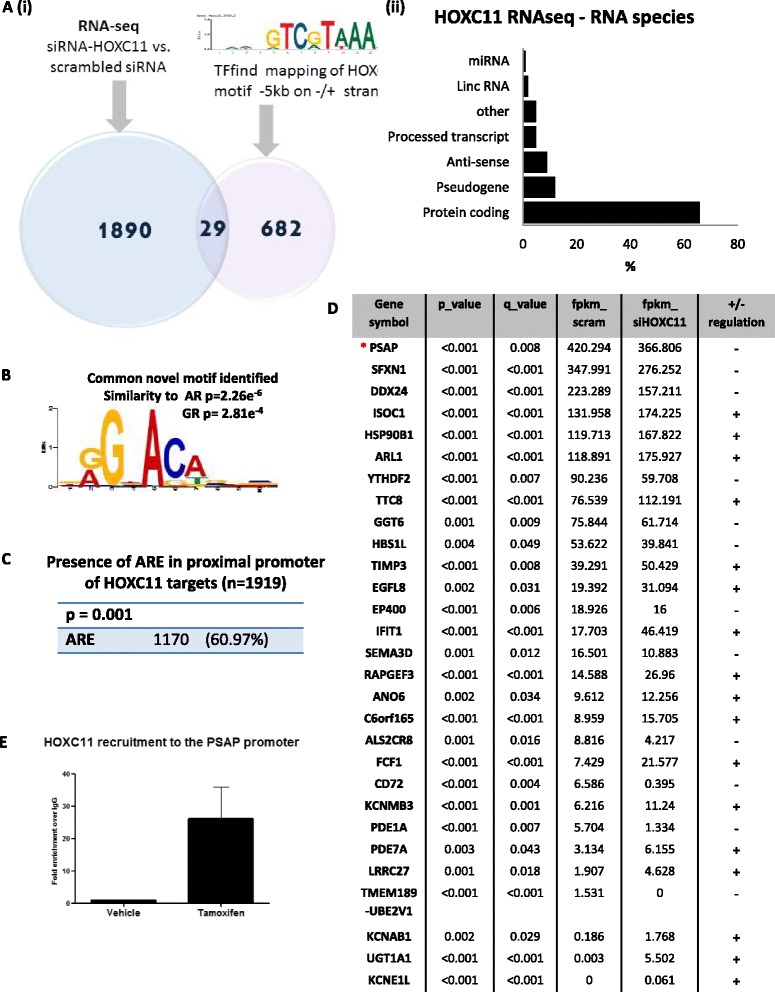
Prosaposin (*PSAP*) was identified as a putative HOXC11 target gene from RNA-sequencing (*RNA-seq*) of endocrine-resistant breast cancer cells in which HOXC11 was knocked down. **a** (*i*) RNA-seq was performed on mRNA from endocrine-resistant breast cancer cells (LY2) in which HOXC11 had been silenced (n = 2): 1,919 hits (cutoff >= 0.95), corresponding to 1,243 Entrez gene IDs were identified. TFFind, a motif mapping programme, was used to search for the HOXC11 motif (consensus sequence: GTCGTAAA) in all annotated human genes from UCSC (26,648 sequences). The analysis of these data resulted in the identification of 711 genes putatively regulated by HOXC11. (*ii*) Breakdown of RNA subtype percentages identified in RNA-seq screening. **b** De novo motif identification was performed on sequences of HOXC11 targets that harbour hormone response elements using the MEME program resulting in the identification of a novel motif (e-value: 1.3e-1337). The novel motif has significant similarity to androgen receptor (*AR*) (*p* value: 2.26e-6) and NR3C1 (glucocorticoid receptor (*GR*)) (*p* value: 2.81e-4). **c** AR motif analysis of total HOXC11 target genes shows that approximately 60 % contain an androgen response element (*ARE*) in the proximal promoter region. **d** Merging RNA-sequencing and HOXC11 transcription factor motif-mapping datasets yielded a total of 29 genes, which were then ranked by magnitude of fragments per Kilobase of transcript per million. **e** Validation of the putative HOXC11 target gene, PSAP, was confirmed by performing chromatin immunoprecipitation to determine recruitment of HOXC11 to the *PSAP* promoter in LY2 (tamoxifen resistant) cells cultured in the presence of tamoxifen versus vehicle. Results are representative of three separate experiments

Expression levels of 1,919 genes were shown to be significantly altered after HOXC11 knockdown in endocrine-resistant breast cancer cells *in vitro* (Fig. 1a (i)). The DEGs included 977 downregulated genes (see Additional file 2) and 942 upregulated genes (see Additional file 3). The sequencing experiment was designed to focus on poly (A) transcripts and as a result over 64 % (1,256) of these DEGs were protein-coding (Fig. 1a (ii)). HOXC11 was verified to be silenced from the RNA-seq data along with selected target gene validation (Additional file 4: Figure S2a (i-ii)).

ARE and ERE motif analysis was performed on all target genes and 1,170 genes were found to harbour an AR binding element (Additional file 5) and 1,558 genes containing an ER binding site (Additional file 6). A total of 949 gene targets were identified that harbour both HREs (Fig. 1b). In order to evaluate whether these HREs are predominately ARE or ERE, we evaluated the left half of both motifs by selecting 10 bp from each of the matched-sequences, which covers either position 2–11 of ER or position 1–10 of AR. De novo motif identification was performed on these sequences using the MEME programme. This resulted in the identification of a novel motif (e-value: 1.3e-1337) (Fig. 1b) returned from MEME which was then compared to all known motifs annotated in the JARSPAR database (JARSPAR CORE 2014) using the TOMTOM programme [35, 36]. The top matched motif was shown to be AR (*p* value: 2.26e-6) (Additional file 4: Figure S2b) and the second most similar motif is NR3C1 (glucocorticoid receptor (GR)) (*p* value: 2.81e-4). Further analysis of the AR binding sites in HOXC11 target genes showed that approximately 60 % of the 1,919 genes are positive for a proximal promoter ARE (Fig. 1c).

RNA-seq data was then merged with HOXC11 motif mapping resulting in the identification of 29 putative direct target genes (Fig. 1d). The list of genes was ranked by magnitude of fragments per kilobase of transcript per million (FPKM) mapped reads to ensure ample abundance of transcript for validation purposes. This resulted in PSAP being identified and validated as the top ranked gene exhibiting differential expression when HOXC11 is knocked down. HOXC11 recruitment to the *PSAP* promoter was verified by ChIP analysis performed on the endocrine-resistant LY2 cells. It was noted that HOXC11 is recruited to the *PSAP* promoter (−581 to −116) under basal conditions but that levels are enhanced when cells are cultured in the presence of tamoxifen (Fig. 1e).

### HOXC11 is recruited to the *PSAP* promoter in vitro when estrogen signalling is disrupted

**Fig. 2:**
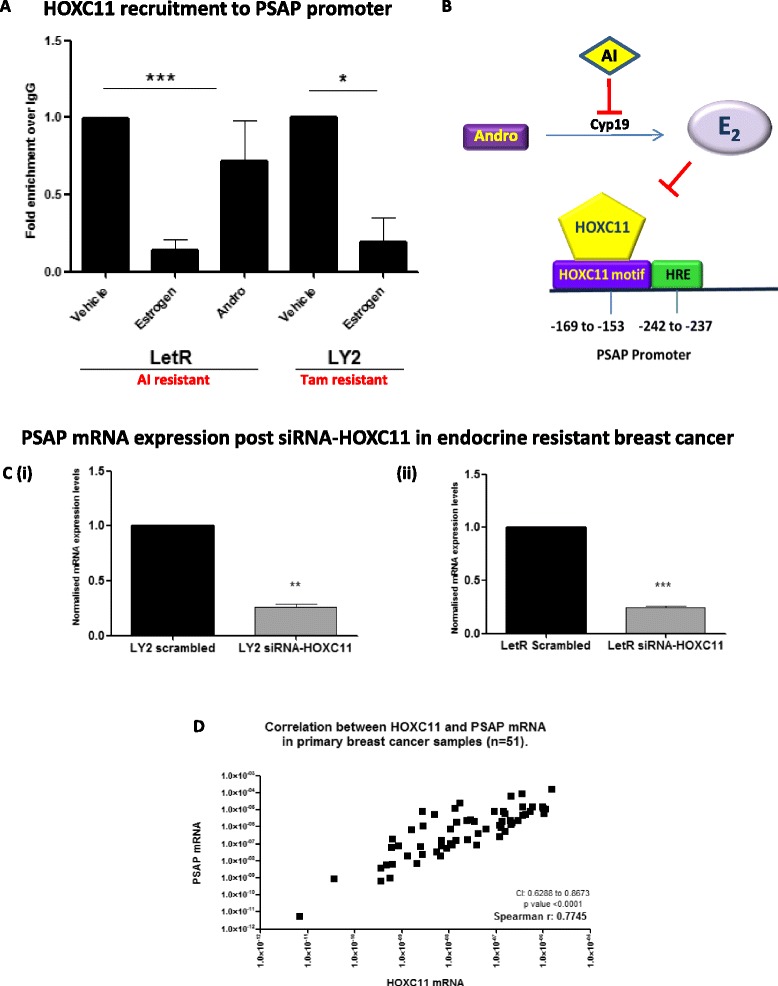
HOXC11 is recruited to the prosaposin (*PSAP*) promoter when estrogen signalling is disrupted. **a** HOXC11 is present on the *PSAP* promoter in letrozole-resistant (*LetR*) cells cultured in androstenedione but not estrogen. HOXC11 is not present on the *PSAP* promoter in LY2 (tamoxifen (*Tam*) resistant) cell cultures in estrogen. Cycle threshold (ct) values were not detected for distal-promoter control reactions for any sample. Error bars are representative of mean ± standard error of the mean from three separate experiments (LetR) and two separate experiments (LY2). **b** In summary, HOXC11 is recruited to the PSAP promoter in endocrine-resistant breast cancer cells in which estrogen signalling has been disrupted. **c** (*i*) Expression of PSAP mRNA is significantly reduced in LY2 cells following silencing of HOXC11 by siRNA. (*ii*) Expression of PSAP mRNA is significantly reduced in LetR cells following silencing of HOXC11 by siRNA. Results are representative of three separate experimental replicates. **d** HOXC11 and PSAP mRNA levels were evaluated by qRT-PCR from RNA extracted from primary breast cancer specimens (n = 51). Spearman’s rank test was used to evaluate correlation between PSAP and HOXC11 transcripts (*r* = 0.7745: *p* <0.0001). *p <0.05, **p <0.001, ***p <0.0001). *Andro* androstenedione, *HRE* hormone response element

Evaluation of HOXC11 recruitment to the *PSAP* promoter in LetR cells was evaluated by ChIP. HOXC11 was present on the promoter when cells were treated with androstenedione but its recruitment was markedly diminished when cells were treated with estrogen, this loss of HOXC11 recruitment was also observed in LY2 cells treated with estrogen (Fig. 2a). These data collectively led us to hypothesize that HOXC11 recruitment to, and subsequent transcription of, the *PSAP* gene occurs when normal estrogen signalling pathways have been disrupted by long-term endocrine treatment (Fig. 2b). Evaluation of HOXC11 regulation of PSAP in tamoxifen and AI-resistant breast cancer cell lines showed a concomitant decrease in PSAP mRNA when HOXC11 was knocked down (Fig. 2c (i-ii)). To determine if these genes are correlated in clinical samples, HOXC11 and PSAP mRNA was assessed in a cohort of primary breast tumours (n = 51). Transcript levels of HOXC11 and PSAP mRNA were found to correlate significantly in primary breast tumour tissue (*r*_S_ = 0.7745, *p* <0.0001) (Fig. 2d).

### PSAP, a known AR activator, is readily detectable in breast cancer cells that have high endogenous levels of HOXC11 and AR protein: over-expression of HOXC11 induces nuclear translocation of AR in MCF7 cells

**Fig. 3:**
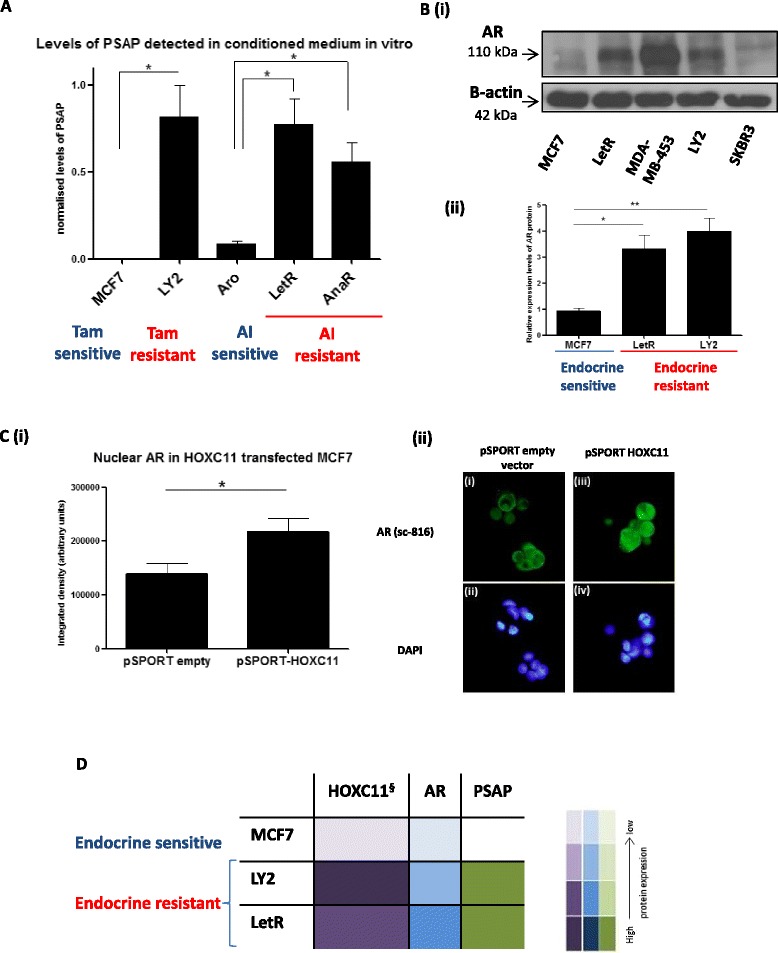
Prosaposin (*PSAP*), a known androgen receptor (*AR*) activator, is readily detectable in breast cancer cells that have high endogenous levels of HOXC11 and AR protein. **a** Secreted PSAP protein levels were evaluated in conditioned media harvested from a range of breast cancer cell lines that were designated endocrine-sensitive (MCF7, Aro) or endocrine-resistant (LY2, letrozole-resistant (*LetR*) and anastrozole-resistant (*AnaR*)). PSAP ELISA detected significant levels of protein in the endocrine-resistant cells. **b** (*i*) Protein levels of AR were determined by western blot analysis of nuclear protein cell lysate extracted from breast cancer cells (MCF7, LetR, MDA-MB-453, LY2 and SKBR3). (*ii*) Endocrine-resistant cells LY2 and LetR exhibited >2 fold increase in AR nuclear protein expression compared to their endocrine-sensitive MCF7 counterparts. Image representative of three experimental replicates. Error bars are representative of mean ± standard error of the mean (SEM) of three separate experiments. **c** (*i*) Transient overexpression of HOXC11 in MCF7 cells resulted in a significant increase in the level of nuclear AR. Image representative of three experimental replicates. Error bars are representative of mean ± SEM of three separate experiments. **c** (*ii*) Representative images of AR in MCF7 cells with transient overexpression of pSPORT empty vector versus pSPORT HOXC11. **d** Elevated levels of PSAP, HOXC11 and AR in endocrine-resistant cells. ^§^McIlroy *et al*., 2010. *p <0.05 **p <0.001. *Tam* tamoxifen, *AI* aromatase inhibitor

PSAP is a secreted protein that is detectable in conditioned medium from breast cancer cell lines. A commercially available ELISA was used to assess levels of secreted PSAP in breast cancer cell-conditioned media. Higher levels of PSAP protein were detectable in all endocrine-resistant cell lines (LY2, LetR and AnaR) compared to their endocrine-sensitive counterparts (MCF7 and Aro) (Fig. 3a). PSAP has been previously reported to induce AR protein in prostate cancer cells [30]. Western blot analysis of a range of cell lines was performed to evaluate if there is an association between elevated levels of PSAP and nuclear AR protein in breast cancer cells. MDA-MB-453, an apocrine breast cancer cell line (ER^−^, PR^−^, HER2^−^, AR^+^) was used as a positive control and SKBR3 (ER^−^, PR^−^, HER2^+^, AR^-low^) as a negative control. Elevated levels of AR were detected in both tamoxifen (LY2)- and AI (LetR)-resistant cell lines and were virtually undetectable in the parental MCF7 cells (Fig. 3b (i-ii)). Transient overexpression of HOXC11 in MCF7 cells resulted in a significant increase in the level of nuclear AR compared to the levels observed in an empty vector control (Fig. 3c (i-ii)). Consolidation of these results with HOXC11 protein expression status [21] shows that endocrine-resistant cells have elevated levels of HOXC11, PSAP and AR protein when compared to endocrine-sensitive MCF7 cells (illustrated in Fig. 3d).

### AR is upregulated and transcriptionally activated in LetR cells treated with recombinant PSAP; AR activation by PSAP can be diminished by co-treatment with the anti-AR drug enzalutamide

**Fig. 4:**
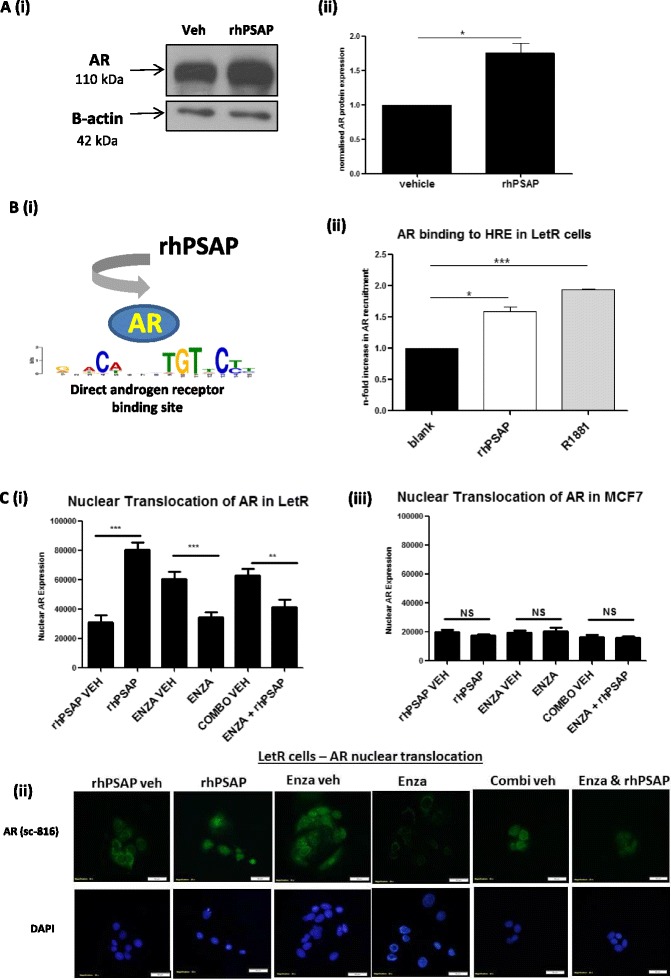
Androgen receptor (*AR*) protein is upregulated and transcriptionally activated in letrozole-resistant (*LetR*) cells treated with recombinant prosaposin (PSAP); the effect of PSAP can be diminished by co-treatment with the anti-AR drug enzalutamide (*Enza*). **a** (*i*) Western blot analysis was used to quantify AR protein levels in LetR cells treated with recombinant human PSAP (*rhPSAP*) (10 ng/ml) versus control (Tris–HCl). (*ii*) Densitometry values from separate experiments. Error bars are representative of mean ± standard error of the mean (SEM) of three separate experiments. **b** (*i*) Modified TransAM assay was used to evaluate AR recruitment to a direct AR binding sequence 5′ – TGTTCT – 3′ when LetR cells are cultured in the presence of rhPSAP. Results are representative of two separate experiments (*ii*) In LetR cells the AR binds the direct AR consensus sequence in the presence of either R1881 or rhPSAP **c**. Nuclear translocation assays were used to observe the trafficking of AR in aromatase-inhibitor (AI)-resistant LetR cells, as visualised by the detection of immunofluorescently labelled AR within the nucleus. **c** (*i*) rhPSAP treatment significantly increased AR nuclear translocation in LetR cells (*p* <0.0001). rhPSAP and Enza combination treatment significantly decreased AR nuclear translocation in LetR cells (*p* <0.05). Error bars are representative of mean ± SEM of three separate experiments. Anti-AR drug treatment (Enza) significantly decreased AR nuclear translocation in LetR cells (*p* <0.05). (*ii*) There was no change in AR nuclear translocation in MCF7 cells following treatment with either rhPSAP or Enza individually or with a combination of both rhPSAP and Enza. Error bars are representative of mean ± SEM of three separate experiments. (*iii*) Representative images of AR nuclear translocation in LetR cells following individual treatments with rhPSAP and Enza and combination treatments: *p <0.05, **p <0.001, ***p <0.0001. *HRE* hormone response element, *Veh* vehicle, *DAPI* 4′, 6-diamidino-2-phenylindole

Treatment of LetR cells with rhPSAP (10 ng/ml) upregulates AR protein expression (Fig. 4a (i-ii)). A modified TransAM transcription factor ELISA (45496, Active Motif) was used to evaluate activation of the AR. The TransAM assay is a DNA-binding ELISA that facilitates the study of transcription factor activation in nuclear cell extracts. The plate consists of an immobilized oligonucleotide containing the HRE binding site (5′-GGTACAnnnTGTTCT-3′) to which AR is known to have high affinity [41]. AR binding to the oligonucleotide was significantly increased in the presence of rhPSAP (10 ng/ml) and R1881 (10 nmol/L) compared to control (Fig. 4b (i-ii)). Nuclear translocation assays were then utilised to monitor AR nuclear trafficking in the presence of rhPSAP plus or minus the anti-AR drug, enzalutamide. Treatment of LetR cells with rhPSAP induced significant translocation of the AR into the nucleus. Enzalutamide alone successfully inhibited the nuclear presence of AR in unstimulated cells. Combined treatment of rhPSAP and enzalutamide significantly reduced the nuclear-trafficking effect of rhPSAP (Fig. 4c (i) with representative images in 4c (ii). When this experiment was carried out in the endocrine-sensitive MCF7 cells there was much less AR detected and no significant change in AR nuclear localisation with treatments (Fig. 4c (iii), for representative images see Additional file 7: Figure S3).

### PSAP increases cell motility and invasiveness of endocrine-resistant breast cancer cells with no significant impact on the function of endocrine-sensitive cells

**Fig. 5:**
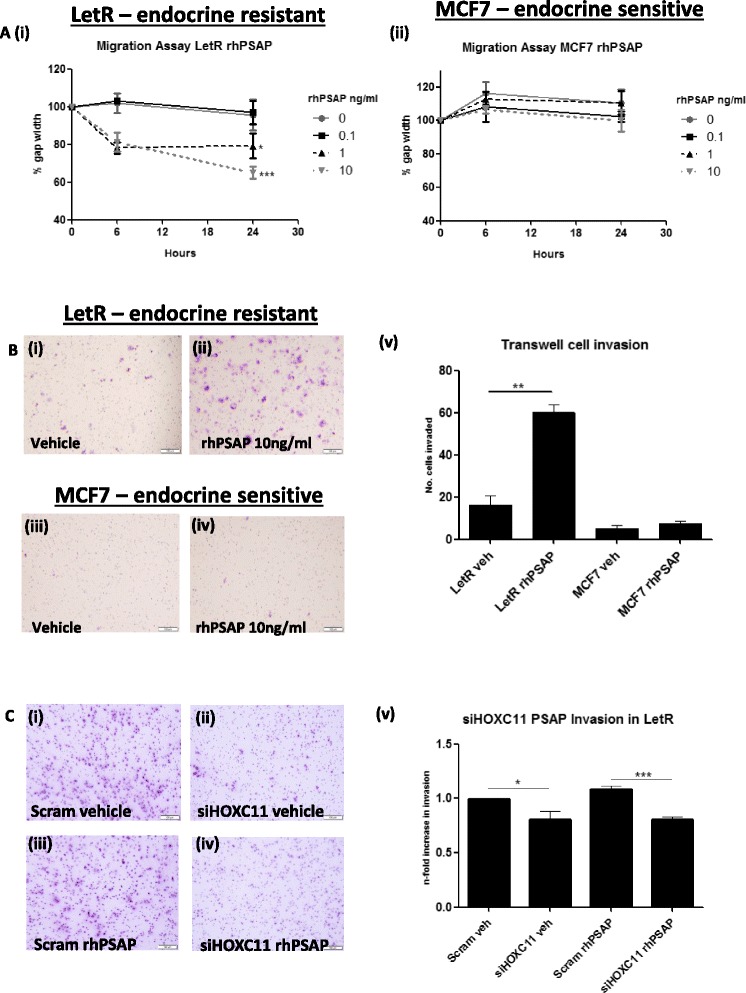
Prosaposin (PSAP) increases cell motility and invasiveness of letrozole-resistant (*LetR*) breast cancer cells with negligible impact on the function of endocrine-sensitive cells. **a** (*i*) In vitro scratch assays were used to evaluate the impact of recombinant human PSAP (*rhPSAP*) on cell migration. Cell migration was significantly enhanced in LetR cells exposed to increasing doses of rhPSAP with maximal impact observed with 10 ng/ml PSAP treatment (*p* <0.0001) (*ii*), conversely, there were no changes in cell migration observed in MCF7 cells (not significant (n.s.). Error bars are representative of mean ± standard error of the mean (SEM) of three separate experiments. **b** (*i*-*ii*) Using matrigel invasion chambers it was determined that the invasive potential of LetR cells was greatly amplified when cells were cultured in the presence of rhPSAP (10 ng/ml) (40 hours) compared to their endocrine-sensitive counterparts, MCF7 which were unresponsive to treatment (*iii*-*iv*). **b** (*v*) Bar-chart representing data from rhPSAP invasion assay in LetR and MCF7. Error bars are representative of mean ± SEM of three separate experiments. **c** Transfection of LetR cells with siRNA-HOXC11 significantly inhibits cell migration (*i*-*ii*), furthermore, treatment of cells with rhPSAP (10 ng/ml) (60 hours) does not stimulate cell invasion when HOXC11 is knocked down (*iii*-*iv*). **c** (*v*) Data from rhPSAP invasion assay in LetR +/− rhPSAP. Error bars are representative of mean ± SEM from four separate experiments: *p <0.05, **p <0.001, ***p <0.0001. *Scram veh* scrambled vehicle

The impact of rhPSAP on cell migration in the endocrine-resistant and sensitive cells was evaluated using a scratch assay. rhPSAP at a range of 1−10 ng/ml induced a significant increase in LetR cell migration after 24 hours (Fig. 5a (i)), conversely, there was no impact on endocrine-sensitive MCF7 cell migration following rhPSAP treatment ((Fig. 5a (ii)). Further functional studies investigated the impact of rhPSAP on cell invasiveness. A Biocoat matrigel invasion assay determined that LetR cells have innate invasive properties that are not apparent in the endocrine-sensitive MCF7 cells (Fig. 5b (i and iii). Upon treatment with rhPSAP there was a significant increase in the numbers of invasive LetR cells (Fig. 5b (ii)), whereas rhPSAP treatment made no significant impact on the invasive potential of the MCF7 cells (Fig. 5b (iv-v)). Knockdown of HOXC11 markedly impacted upon the invasive capacity of LetR cells when cultured under vehicle and rhPSAP treatments (Fig. 5c (i-v).

### Inhibition of AR can diminish letrozole-resistant cell proliferation and the pro-migratory impact of rhPSAP

**Fig. 6:**
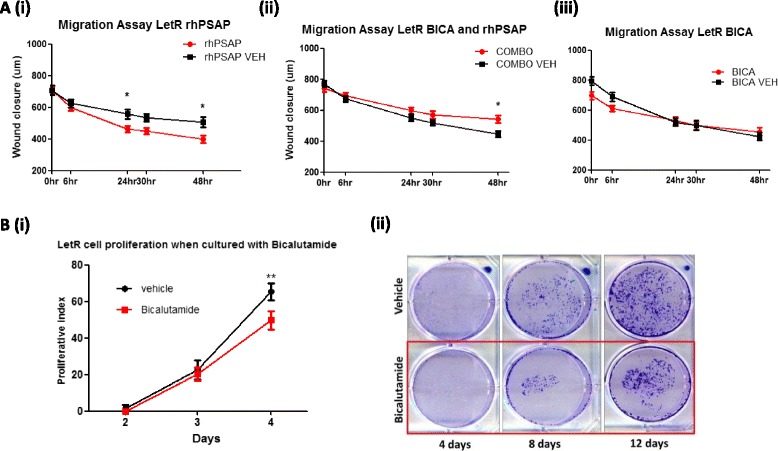
Inhibition of androgen receptor (*AR*) can diminish letrozole-resistant (*LetR*) cell proliferation and the pro-migratory impact of recombinant human prosaposin (*rhPSAP*). **a** Scratch assays were utilised to investigate the impact of rhPSAP on the migratory capacity of aromatase inhibitor (AI)-resistant LetR cells. Anti-AR, bicalutamide (*Bica*), treatment did not significantly impact on the migratory capacity of AI-resistant LetR cells over 48 hours (*i*); rhPSAP treatment (10 ng/ml) significantly increased LetR cell migration over 48 hours (*ii*). Combination treatment with rhPSAP and bicalutamide significantly inhibited rhPSAP-mediated cell migration in AI-resistant LetR cells over 48 hours (*iii*). **b** (*i*) MTS and (*ii*) colony forming assays were performed to assess the impact of bicalutamide on AI cell proliferation. Treatment of AI-resistant cells with the anti-AR drug, bicalutamide, reduces cell proliferation in LetR. Error bars are representative of mean ± standard error of the mean from three separate experiments. *p <0.05, **p <0.001. *VEH* vehicle, *COMBO* combination

The pro-migratory influence of PSAP could be successfully attenuated by co-treating with the anti-AR drug (bicalutamide) (Fig. 6a (i-ii)). Bicalutamide treatment alone did not have any impact on cell migration (Fig. 6a (iii)). Follow-on studies investigated the impact of anti-AR treatment on LetR cell growth and it was observed that bicalutamide significantly inhibited cell proliferation in both MTS and colony forming assays (Fig. 6b (i-ii)).

### Pre-operative serum levels of PSAP are significantly higher in breast cancer patients whose disease subsequently recurred: high levels of both PSAP and AR mRNA associate very strongly with poor disease-free survival in endocrine-treated breast cancers

**Fig. 7:**
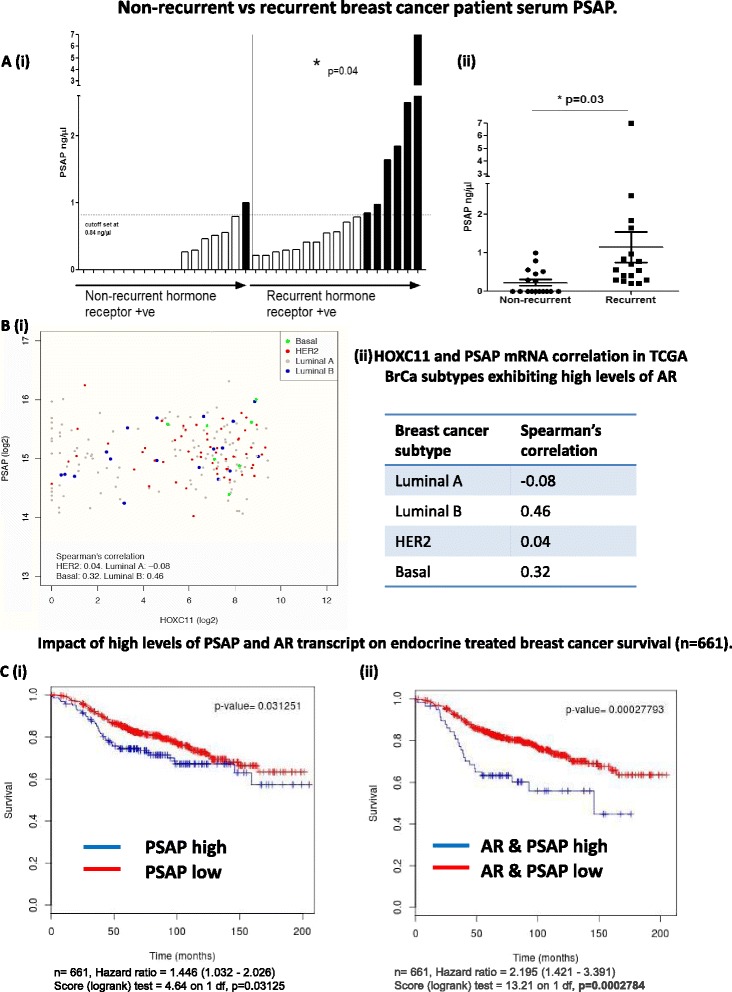
Pre-operative serum levels of prosaposin (*PSAP*) are significantly higher in breast cancer patients whose disease subsequently recurred. High levels of both PSAP and androgen receptor (*AR*) mRNA are very strongly associated with poor disease-free survival (DFS) in endocrine-treated breast cancer. **a** A commercially available ELISA kit for human PSAP was used to quantify serum levels of the secreted protein in sera from breast cancer patients. PSAP levels were evaluated in a subset of endocrine-resistant patients versus age-matched controls. (*i*) The non-recurrent control group was used to generate a training set to establish a cutoff value of 0.84 ng/μl (median + 2 SD) to distinguish between patients sensitive and resistant to endocrine therapy (*dashed horizontal line*). The median follow up of the cohort was 35 months. Preliminary findings indicate approximately 35 % of postmenopausal breast cancer patients who had recurrent disease whilst on endocrine therapy expressed PSAP serum levels elevated above the cutoff (*p* = 0.04). (*ii*) Levels of PSAP detected in sera from patients with recurrent breast cancer was significantly higher than those of endocrine-responsive patients (*p* = 0.03). **b** (*i*) Breast cancer patients (The Cancer Genome Atlas dataset (*TGCA*) with high androgen receptor (AR) expression (upper quartile) were selected and Spearman’s correlation between HOXC11 and PSAP was calculated for each subtype: *gray* luminal A, *blue* luminal B, *red* basal, *green* HER2 (n = 199). (*ii*) Tabulated results show Spearman’s correlation between HOXC11 and PSAP mRNA in AR-high breast cancers per subtype. **c** (*i*) Meta-analysis of transcript levels in breast cancer patients (n = 661) indicates that PSAP mRNA is a significant predictor of poor response to endocrine treatment in endocrine-treated patients (*p* = 0.03; hazard ratio (HR) 1.4). (*ii*) Patients expressing high mRNA levels of both PSAP and AR experience much shorter periods of DFS reflected in an HR of 2.2 (*p* = 0.0003). *BrCa* breast cancer, *HER2* human epidermal growth factor 2

Evaluation of secreted PSAP levels in a cohort of endocrine resistant patient sera along with age-matched, non-recurrent controls (n = 34) were used to generate a training set; with the aim to determine whether or not PSAP could distinguish between patients sensitive or resistant to endocrine therapy. The cutoff was set at 0.84 ng/μl, which was established from the mean + 2 SD of PSAP serum levels in the non-recurrent group (illustrated by dashed line Fig. 7a (i)). There was a significant difference in PSAP expression levels between the recurrent and non-recurrent samples (*p* = 0.03) (Fig. 7a (ii)). The median follow up of patients in this cohort was 35 months. These data indicate that approximately 35 % of patients with postmenopausal breast cancer, who have recurrence whilst on endocrine therapy, express PSAP serum levels elevated above the threshold. Serum PSAP levels elevated above the cutoff were significantly associated with recurrence in an endocrine-treated cohort of patients with breast cancer (*p* = 0.04) (Fig. 7a (i)).

Analysis of TCGA breast cancer dataset showed there to be a weakly positive correlation between HOXC11 and PSAP mRNA only in the luminal B subtype (*r*_s_ 0.24). When patients were further stratified to focus on those expressing high levels of AR, the correlation between the two genes was again only observed in subtypes of poorer prognosis, in particular luminal B (*r*_s_ 0.46) and basal (*r*_s_ 0.32) (Fig. 7b (i-ii)).

We then evaluated the impact of PSAP transcript levels on endocrine-treated breast cancer patient outcome using the Breastmark meta-analysis software [40]. The high expression group in each plot (blue) accounts for the upper quartile of expression levels for a particular transcript and the low expression group (red) the remaining 75 %. This was applied to each of the individual datasets and the information was then combined to perform a global pooled survival analysis; 661 samples were used in this comparison. The HR was generated using Cox regression and the logrank test was used to assign significance to the HR. High expression levels of PSAP transcript were found to be strongly predictive of poor DFS (*p* = 0.03) with an HR of 1.45 (Fig. 7c (i)). The association of PSAP with poor outcome is also observed in an HR curve generated for PSAP in TCGA dataset (Additional file 8: Figure S4a). Moreover, when we combined high expression of both PSAP and AR mRNA there was a more pronounced detrimental impact on patient DFS (*p* = 0.0003) with an HR of 2.2 (Fig. 7c (ii)) compared to either transcript alone (Additional file 8: Figure S4b).

## Discussion

*HOX* genes have been implicated in the development of haematological and solid tumour malignancies [42], with many studies focusing on their potential role in endocrine cancers [27, 28, 43]. *HOX* genes play vital roles in body mapping during development and posterior *HOX* genes in particular are under very tight regulation by estrogen [44]. It is therefore of interest to understand how the abnormal rebooting of posterior *HOXC* genes in mammary epithelial tumour cells can potentiate endocrine-resistant cancer and the development of metastasis [21, 27, 45]. In this current study we wanted to further elucidate the role of HOXC11 with regard to endocrine resistance and steroidal adaptability in breast cancer. RNA-seq experiments identified 1,919 DEGs when HOXC11 was silenced in endocrine-resistant breast cancer. Analysis of genes harbouring an HRE in the proximal promoter resulted in a novel motif with significant sequence similarity to AR and GR. Filtering of the data using motif mapping identified 29 putative direct HOXC11 target genes including PSAP which is a known AR activator [46]. HOXC11 recruitment to the DNA was found to be highly responsive to the steroid environment. We observed that HOXC11 recruitment to the proximal *PSAP* promoter is impaired by treatment with estrogen in endocrine-resistant cell lines. This is likely due to the fact that a number of posterior *HOXC* genes are estrogen-repressed [27], the exact mechanism is unknown but it could be postulated to be due to chromatin remodelling [47]. It is therefore notable that there was no significant alteration in HOXC11 recruitment in the AI-resistant cells treated with androstenedione; suggesting alternate patterns of HOXC11 recruitment can be dictated by the steroid microenvironment.

The upregulation of PSAP gene expression by HOXC11 in endocrine-resistant breast cancer cells was of interest primarily due to its association with tamoxifen resistance [29], but also because of its potential as an AR activator. Previous studies by Koochekpour *et al*. demonstrated PSAP to cause ligand-independent activation of AR in prostate cancer via activation of the PI3K pathway [46]. To this end we decided to explore whether or not PSAP can also activate AR in AI-resistant breast cancer cells in the presence of unconverted endogenous androgens. AR transcription factor activity assays demonstrated successful activation of AR by treatment with rhPSAP, which was of similar magnitude to R1881 exposure. This was further validated by nuclear translocation experiments demonstrating that AR activation by PSAP in resistant cells could be attenuated by treatment with an anti-AR drug. The majority of research articles investigating the role of AR and/or androgens in breast cancer have concluded that male sex hormones and receptors have an inhibitory impact on breast cancer cell growth [13, 48, 49]. However, if we consider the uniquely androgenic steroid environment that arises from treatment with an AI it should be queried whether this assumption is still relevant in the event of recurrence with this class of drug.

PSAP had previously been reported to stimulate ER-positive endocrine-sensitive breast cancer cell growth [50], however, in the resistant setting, no significant impact on cell growth was observed (Additional file 9: Figure S5). The focus of functional studies was shifted to evaluate the impact of PSAP on cell migration. Treatment of cells with rhPSAP demonstrated that only endocrine-resistant breast cancer cells respond to the pro-migratory and pro-invasive effects of the protein. Further experiments indicated that HOXC11 upregulation is required for the pro-invasive impact of PSAP to manifest fully, suggesting that HOXC11 expression is key to development of the aggressive phenotype. Increased responsiveness in resistant cells may be due to a number of unexplored factors including expression of cell surface receptors capable of binding PSAP [51] or activation of specific signalling pathways [30]. Indeed, PSAP does appear to induce activation of p-AKT in breast cancer cells *in vitro* (Additional file 10: Figure S6a and b). This leads to the appealing possibility of identifying new hallmarks of resistance to endocrine therapy and the elucidation of novel drug targets. The clinical potential of PSAP is highlighted by the significantly elevated levels of the protein detected in serum from breast cancer patients who experienced disease recurrence on endocrine therapy.

HOXC11 and PSAP mRNA levels are strongly correlated in a primary breast cancer cohort (*r*_s_ = 0.7692, n = 51). Further analysis of TCGA datasets also demonstrate a correlation between HOXC11 and PSAP transcript levels, specifically in luminal B breast cancer in which AR is elevated (*r*_s_ = 0.46). HOXC11 over-expression in MCF7 cells results in a significant increase in nuclear AR suggesting that HOXC11 upregulation may be a major determinant of the adaptive process. In our AI-resistant breast cancer model we have shown that PSAP is capable of upregulating and activating AR in the context of high HOXC11 expression. Collectively these data suggest that the tumour promotional activation of AR may be an adaptive response primarily in cancers exhibiting dysregulated estrogen signalling. Importantly these findings open up the possibility of utilizing anti-androgens in the treatment of a specific subtype of breast cancer expressing high levels of AR and PSAP, which may not exhibit sustained response to endocrine therapy. Studies by other groups [16] have shown AR to be co-opted as a transcription factor in place of ER in the apocrine subtype; in our model we have also observed AR recruitment to some, but not all, ER targets evaluated (unpublished observations). Further investigations into the AR transcriptome in AI-resistant breast cancer will help elucidate which processes are being impacted. We would purport from these observations in AI-treated breast cancer (in which estrogen signalling has been dysregulated) that tumour cells have the potential to adapt and utilize bioavailable steroids such as those of adrenal origin: 75 % of breast tumours are positive for AR and so it is of interest to evaluate how these cells adapt to the preponderance of circulating androgens that occurs during prolonged treatment with AI therapy. Evidence that this may be a mechanism of resistance is emerging from clinical data suggesting that levels of androgens, and more specifically androstenedione, are increased in breast cancers refractory to AI therapy [52]. Such disruption of normal hormonal homeostasis of the breast will result in perturbation of normal mammary epithelial maintenance [53] and this is what we hypothesize may be contributing to the development of endocrine resistance.

## Conclusion

This study is supportive of a growing consensus that high levels of AR may have a crucial role to play in the development of endocrine resistance [19, 20]. Notably, we have identified a secreted marker, PSAP, which may have utility in directing anti-AR second-line therapy for patients failing on conventional endocrine treatment. In conclusion, steroid receptor interplay may exert a more dynamic and influential role in endocrine resistance than previously thought. To this end a further exploration of the role of the AR, in the development of AI resistance in particular, is warranted.

## Additional files

Additional file 1: Figure S1.
**a** Modified TransAM assay was used to evaluate androgen receptor (*AR*) recruitment to a direct AR binding sequence 5′ – TGTTCT – 3′. LNCaP steroid-dependent prostate cancer cells were evaluated as a positive control. Both LNCaP and letrozole-resistant (*LetR*) cells were cultured in the presence of R1881 (10 nmol/L), recombinant human prosaposin (*rhPSAP*) (10 ng/ml) and androstenedione (100 nmol/L). **b** Optimal AR antibody concentration was evaluated using R1881 (10 nmol/L) LNCaP nuclear lysate. An antibody dilution of 1:250 provided optimal signal-noise ratio. *HRE* human response element. (PDF 94 kb) Additional file 2:
**Table: HOXC11 KD downregulated genes.** HOXC11 differentially expressed genes (DEGs) downregulated after HOXC11 knockdown, ranked by *p* value. (PDF 1500 kb) Additional file 3:
**Table: HOXC11 KD upregulated genes.** HOXC11 differentially expressed genes (DEGs) upregulated after HOXC11 knockdown, ranked by *p* value. (PDF 259 kb) Additional file 4: Figure S2.
**a** *(i)* Validation of HOXC11 target gene, GREB1, in LY2 cells in which HOXC11 was knocked down by siRNA. *(ii)* Levels of concomitant GREB1 decrease when HOXC11 is silenced is comparable to alterations observed in HOXC11 RNA-seq data. **b** The top novel motif returned from MEME was then compared to all known motifs annotated in the JARSPAR database (JARSPAR CORE 2014) using the TOMTOM program. The top matched motif is shown to be androgen receptor (AR) (*p* value: 2.26e-6) and the second most similar motif is NR3C1 glucocorticoid receptor (GR) (*p* value: 2.81e-4). (PDF 209 kb) Additional file 5:
**Table: Androgen receptor (AR) motif results** ***p*** **<0.001.** A 400-bp-sized window surrounding starting sites of HOXC11 target genes was selected for AR motif searching. The searching process was performed using the FIMO program available in MEME-suite with the *p* value significant cutoff set at 0.001. (PDF 804 kb) Additional file 6:
**Table: Estrogen receptor (ER) motif results (** ***p*** **<0.001).** A 400-bp-sized window surrounding starting sites of HOXC11 target genes was selected for ER motif searching. The searching process was performed using the FIMO program available in MEME-suite with the *p* value significant cutoff set at 0.001. (PDF 198 kb) Additional file 7: Figure S3. Representative images of androgen receptor (*AR*) nuclear translocation in MCF7 cells following individual treatments with recombinant human prosaposin (*rhPSAP*) and enzalutamide (*Enza*) and combination treatments. (PDF 114 kb) Additional file 8: Figure S4.
**a** Hazard ratio (*HR*) curve was generated for prosaposin (*PSAP*) using the TCGA dataset. PSAP expressions between 25 and 75 % quantiles have a *HR* consistently >1. **b** Androgen receptor (*AR*) mRNA does not associate with poor disease-free survival (DFS) in endocrine-treated breast cancer. Kaplan-Meier survival curves were generated to assess the impact of high androgen receptor transcript levels on survival of endocrine-treated patients with breast cancer (n = 661). (PDF 204 kb) Additional file 9: Figure S5. An MTS assay was utilised to assay the impact of increasing doses of recombinant human prosaposin (*rhPSAP*) on letrozole-resistant (*LetR*) cell proliferation after 24 hours treatment. No change in *LetR* cell proliferation was detected following increased doses in *rhPSAP* (n = 3). (PDF 86 kb) Additional file 10: Figure S6.
**a** Letrozole-resistant (*LetR*) cells were treated with increasing doses of recombinant human prosaposin (*rhPSAP*). p-AKT expression in these cells showed a dose-dependent increase with *rhPSAP* treatment (n = 3). **b** In *LetR* cells, there is increased protein expression of p-AKT when treated with *rhPSAP*. However, when treated with BEZ235 (PI3K inhibitor), there is marked reduction in p-AKT expression. This reduction is also similarly seen in the combination group of *rhPSAP* and BEZ235 treatments (n = 3). (PDF 138 kb)

## Abbreviations

AI: aromatase inhibitor
AnaR: anastrozole-resistant
Andro: androstenedione
AR: androgen receptor
ARE: androgen response element
Aro: MCF7aromatase stable cell line
ATCC: American Type Culture Collection
Bica: bicalutamide
bp: base pairs
ChIP: chromatin immunoprecipitation
DEG: differentially expressed gene
DFS: disease-free survival
ELISA: enzyme-linked immunosorbent assay
Enza: enzalutamide
ER: estrogen receptor
ERE: estrogen response element
GR: glucocorticoid receptor
HR: hazard ratio
HRE: hormone response element
LetR: letrozole-resistant
PBS: phosphate-buffered saline
PFM: position frequency matrices
PSAP: prosaposin
PWM: positive weight matrices
qRT-PCR: quantitative reverse-transcribed PCR
rhPSAP: recombinant human prosaposin
RNA-seq: RNA sequencing
RT: room temperature
siRNA: small interfering RNA
TCGA: The Cancer Genome Atlas
