# Supplementary material for: Prosaposin activates the androgen receptor and potentiates resistance to endocrine treatment in breast cancer
Source: Breast Cancer Res. 2015 Sep 4;17(1):123. doi: 10.1186/s13058-015-0636-6 (PMC4560928; doi:10.1186/s13058-015-0636-6)

Supplemental Figure 1

A

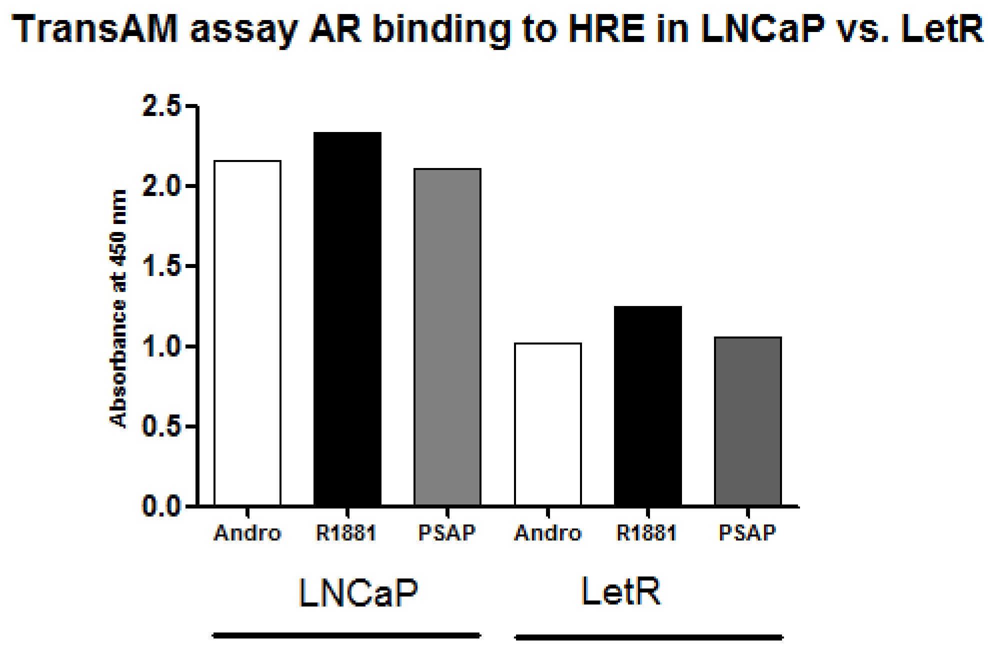

B

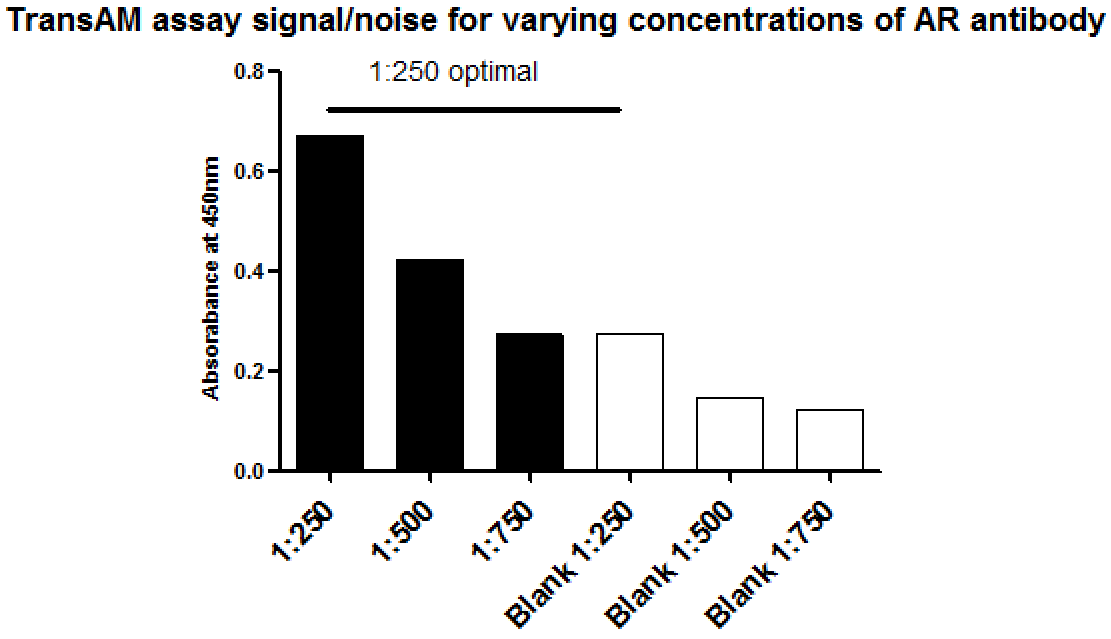

Supplement: Additional file 1: Figure S1. — a Modified TransAM assay was used to evaluate androgen receptor (AR) recruitment to a direct AR binding sequence 5′ – TGTTCT – 3′. LNCaP steroid-dependent prostate cancer cells were evaluated as a positive control. Both LNCaP and letrozole-resistant (LetR) cells were cultured in the presence of R1881 (10 nmol/L), recombinant human prosaposin (rhPSAP) (10 ng/ml) and androstenedione (100 nmol/L). b Optimal AR antibody concentration was evaluated using R1881 (10 nmol/L) LNCaP nuclear lysate. An antibody dilution of 1:250 provided optimal signal-noise ratio. HRE human response element. (PDF 94 kb) [file 13058_2015_636_MOESM1_ESM.pdf]
