# Supplementary material for: Prosaposin activates the androgen receptor and potentiates resistance to endocrine treatment in breast cancer
Source: Breast Cancer Res. 2015 Sep 4;17(1):123. doi: 10.1186/s13058-015-0636-6 (PMC4560928; doi:10.1186/s13058-015-0636-6)
Supplement: Additional file 2: — Table: HOXC11 KD downregulated genes. HOXC11 differentially expressed genes (DEGs) downregulated after HOXC11 knockdown, ranked by p value. (PDF 1500 kb) [file 13058_2015_636_MOESM2_ESM.pdf]

test_id	gene_id	gene	locus	value_1	value_2	ln.fold_cha	test_stat	p_value	q_value	significant	q1_FPKM	q1_conf_lo	q1_conf_hi	q2_FPKM	q2_conf_lo	q2_conf_hi
ENSG00000248399	ENSG00000248399	RP11-503N18.4	4:2420700	7.445	0.012	-6.456	11.522	0	0	yes	7.44544	0	15.7793	0.0116921	0.011048	0.012337
ENSG00000169314	ENSG00000169314	C22orf15	22:2410520	14.526	0.025	-6.354	47.898	0	0	yes	14.5263	11.0916	17.961	0.0252643	0.022223	0.028306
ENSG00000223692	ENSG00000223692	DIP2A-IT1	21:478788	0.615	0.003	-5.432	6.58	0	0	yes	0.615422	0	1.63085	0.00269305	0.002544	0.002842
ENSG00000258790	ENSG00000258790	RP11-561B11.2	14:355141	0.251	0.001	-5.305	16.465	0	0	yes	0.250977	0.0897284	0.412225	0.00124626	0.001184	0.001309
ENSG00000258657	ENSG00000258657	RP11-104E19.1	14:250649	0.338	0.002	-5.039	4.92	0	0	yes	0.337772	0	0.838304	0.00218794	0	0.005283
ENSG00000207783	ENSG00000207783	MIR564	3:4480320	302.5	2.28	-4.888	12.253	0	0	yes	302.5	64.6701	540.33	2.28012	1.97055	2.58969
ENSG00000240758	ENSG00000240758	RP11-155G14.5	7:1280958	14.938	0.145	-4.637	31.462	0	0	yes	14.9376	10.7281	19.1471	0.144725	0.13222	0.157231
ENSG00000125848	ENSG00000125848	FLRT3	20:138298	0.864	0.01	-4.426	3.986	0	0.001	yes	0.863568	0	2.75647	0.010335	0.006681	0.013989
ENSG00000174564	ENSG00000174564	IL20RB	3:1365810	10.307	0.174	-4.082	8.71	0	0	yes	10.307	8.62059	11.9933	0.173962	0.013423	0.334501
ENSG00000233927	ENSG00000233927	RPS28	19:838638	229.943	4.919	-3.845	13.736	0	0	yes	229.943	197.971	261.914	4.91892	2.25163	7.58622
ENSG00000171056	ENSG00000171056	SOX7	8:1058127	0.362	0.008	-3.835	9.332	0	0	yes	0.362295	0.0753251	0.649264	0.00782442	0.006107	0.009542
ENSG00000256804	ENSG00000256804	RP13-977J11.5	12:132568	3.048	0.084	-3.592	6.566	0	0	yes	3.04824	0	6.35748	0.0839903	0.072587	0.095394
ENSG00000204311	ENSG00000204311	DFNB59	2:1792786	4.311	0.12	-3.582	4.2	0	0.001	yes	4.31108	2.36436	6.25781	0.119922	0	0.31716
ENSG00000125245	ENSG00000125245	GPR18	13:998530	3.602	0.101	-3.573	3.86	0	0.002	yes	3.60199	2.53487	4.6691	0.101066	0	0.285763
ENSG00000149380	ENSG00000149380	P4HA3	11:737237	0.09	0.003	-3.489	4.619	0	0	yes	0.0901365	0	0.226221	0.002752	0.002608	0.002896
ENSG00000133808	ENSG00000133808	MICALCL	11:122976	5.722	0.184	-3.435	6.113	0	0	yes	5.72228	1.82795	9.61661	0.184348	0.019462	0.349233
ENSG00000100385	ENSG00000100385	IL2RB	22:375151	2.847	0.098	-3.371	3.512	0	0.008	yes	2.84711	0	7.39999	0.0977709	0	0.201623
ENSG00000254827	ENSG00000254827	SLC22A18AS	11:290900	23.618	0.839	-3.337	6.473	0	0	yes	23.6183	19.2936	27.943	0.8392	0	1.69075
ENSG00000250251	ENSG00000250251	PKD1P6	16:150685	24.63	0.884	-3.327	6.459	0	0	yes	24.6301	19.3329	29.9273	0.884398	0	1.77541
ENSG00000182177	ENSG00000182177	ASB18	2:2370738	6.251	0.226	-3.318	5.025	0	0	yes	6.25121	4.17993	8.32249	0.226494	0	0.516015
ENSG00000112182	ENSG00000112182	BACH2	6:9063624	5.044	0.185	-3.307	5.708	0	0	yes	5.04438	1.24759	8.84118	0.184819	0.022033	0.347605
ENSG00000101082	ENSG00000101082	SLA2	20:352341	4.083	0.16	-3.241	5.107	0	0	yes	4.08261	3.24966	4.91556	0.159721	0	0.3598
ENSG00000214184	ENSG00000214184	AC012487.2	2:1090650	24.198	0.98	-3.207	5.706	0	0	yes	24.198	19.1795	29.2165	0.979511	0	2.06172
ENSG00000234925	ENSG00000234925	RP11-254B13.3	12:690046	6.147	0.256	-3.178	11.934	0	0	yes	6.14738	3.22737	9.0674	0.256257	0.194557	0.317956
ENSG00000137210	ENSG00000137210	TMEM14B	6:1074797	6.81	0.286	-3.171	9.147	0	0	yes	6.80978	2.78398	10.8356	0.285683	0.182154	0.389212
ENSG00000226312	ENSG00000226312	CFLAR-AS1	2:2019808	8.63	0.375	-3.136	3.965	0	0.002	yes	8.62961	0	17.4233	0.375152	0	0.828907
ENSG00000241790	ENSG00000241790	ENO1P4	2:2024849	1.312	0.058	-3.122	13.898	0	0	yes	1.3122	0.751951	1.87245	0.0578067	0.049712	0.065901
ENSG00000235910	ENSG00000235910	AP006216.12	11:116706	0.128	0.006	-3.117	40.688	0	0	yes	0.128288	0.115394	0.141181	0.00568023	0.005023	0.006337
ENSG00000244122	ENSG00000244122	UGT1A7	2:2345262	5.092	0.23	-3.096	6.488	0	0	yes	5.09177	4.03585	6.14768	0.230352	0.015774	0.444929
ENSG00000234036	ENSG00000234036	RP11-123J14.2	4:7787085	139.457	6.779	-3.024	7.902	0	0	yes	139.457	116.376	162.538	6.77888	1.71339	11.8444
ENSG00000258725	ENSG00000258725	AC068831.8	15:915092	2.639	0.129	-3.022	3.578	0	0.006	yes	2.63857	1.05682	4.22032	0.128563	0	0.33159
ENSG00000155962	ENSG00000155962	CLIC2	X:1545054	3.151	0.159	-2.987	5.081	0	0	yes	3.15067	1.41478	4.88655	0.158953	0	0.324033
ENSG00000178796	ENSG00000178796	RIIAD1	1:1516735	18.002	0.926	-2.967	16.093	0	0	yes	18.0017	12.8089	23.1945	0.926428	0.713673	1.13918
ENSG00000174527	ENSG00000174527	MYO1H	12:109826	1.44	0.075	-2.957	5.737	0	0	yes	1.43952	0	2.92205	0.0748325	0.071599	0.078066
ENSG00000228925	ENSG00000228925	AC016722.4	2:4712641	0.272	0.015	-2.92	6.315	0	0	yes	0.271946	0.0208325	0.52306	0.0146735	0.013961	0.015386
ENSG00000249119	ENSG00000249119	MTND6P4	5:1342405	0.568	0.031	-2.912	4.004	0	0.001	yes	0.567641	0	1.38721	0.0308725	0.025457	0.036288
ENSG00000143627	ENSG00000143627	PKLR	1:1552326	0.349	0.02	-2.884	6.702	0	0	yes	0.349047	0.0494582	0.648636	0.0195231	0.018311	0.020735
ENSG00000258509	ENSG00000258509	RP11-857B24.3	14:453460	30.421	1.747	-2.857	3.756	0	0.003	yes	30.4206	20.1423	40.699	1.74656	0	4.33766
ENSG00000151117	ENSG00000151117	TMEM86A	11:187146	4.276	0.253	-2.826	4.148	0	0.001	yes	4.27584	0	9.14331	0.253346	0.063645	0.443046
ENSG00000137101	ENSG00000137101	CD72	9:3560528	6.586	0.395	-2.814	3.739	0	0.004	yes	6.58604	5.24258	7.92949	0.394945	0	0.983918
ENSG00000103023	ENSG00000103023	PRSS54	16:582838	2.957	0.18	-2.798	3.82	0	0.003	yes	2.95661	2.0841	3.82911	0.180117	0	0.43861
ENSG00000214597	ENSG00000214597	GS1-393G12.10	8:1455758	5.081	0.333	-2.726	6.036	0	0	yes	5.08064	3.34341	6.81787	0.332689	0.054542	0.610836
ENSG00000090530	ENSG00000090530	LEPREL1	3:1896745	2.702	0.178	-2.72	5.45	0	0	yes	2.70206	1.08222	4.3219	0.177942	0.035891	0.319993
ENSG00000169184	ENSG00000169184	MN1	22:281442	3.244	0.228	-2.655	6.335	0	0	yes	3.2435	1.06119	5.42582	0.227976	0.113988	0.341963
ENSG00000256028	ENSG00000256028	RP11-197N18.4	12:123405	0.965	0.068	-2.65	5.194	0	0	yes	0.965272	0.723595	1.20695	0.0681775	0.000722	0.135633
ENSG00000229951	ENSG00000229951	AC104695.3	2:2860727	4.253	0.303	-2.64	7.084	0	0	yes	4.25331	3.47754	5.02908	0.303433	0.084135	0.522731
ENSG00000178836	ENSG00000178836	AC114812.5	2:2345262	0.001	0	-2.588	37.18	0	0	yes	0.000645009	0.0005833	0.000706719	4.85E-05	4.36E-05	5.34E-05
ENSG00000245281	ENSG00000245281	CTD-2547L16.1	8:1791393	1.169	0.088	-2.585	6.396	0	0	yes	1.16921	0.22709	2.11133	0.088175	0.082601	0.093749
ENSG00000253213	ENSG00000253213	RP11-546B8.3	5:1702888	0.028	0.002	-2.556	25.109	0	0	yes	0.0283536	0.0242823	0.032425	0.00220047	0.001883	0.002518

ENSG00000164621	ENSG00000164621	SMAD5-AS1	5:1354651	0.018	0.001	-2.526	39.234	0	0	yes	0.0179531	0.0163846	0.0195217	0.00143569	0.0013	0.001572
ENSG00000151743	ENSG00000151743	AMN1	12:318000	13.765	1.123	-2.506	7.436	0	0	yes	13.7649	9.38501	18.1448	1.1229	0.455577	1.79023
ENSG00000224437	ENSG00000224437	RP11-161E22.2	9:7314994	3.129	0.261	-2.483	5.8	0	0	yes	3.12938	1.32263	4.93612	0.261175	0.095993	0.426357
ENSG00000070601	ENSG00000070601	FRMPD1	9:3751088	0.055	0.005	-2.469	5.865	0	0	yes	0.0549545	0.00872542	0.101184	0.00465102	0.004471	0.004831
ENSG00000255905	ENSG00000255905	AL391319.1	6:1688418	0.047	0.004	-2.46	15.107	0	0	yes	0.0470497	0.036395	0.0577044	0.00401902	0.003078	0.00496
ENSG00000104976	ENSG00000104976	SNAPC2	19:798519	393.498	34.137	-2.445	4.413	0	0	yes	393.498	0	826.025	34.1372	29.3828	38.8916
ENSG00000175892	ENSG00000175892	AC009336.1	2:1769863	0.423	0.037	-2.439	6.238	0	0	yes	0.422905	0.103128	0.742682	0.0369142	0.029568	0.044261
ENSG00000237779	ENSG00000237779	ZNF638-IT1	2:7150369	0.722	0.064	-2.422	5.28	0	0	yes	0.722333	0.0608015	1.38386	0.0641116	0.060744	0.067479
ENSG00000160844	ENSG00000160844	GATS	7:9977518	1.514	0.135	-2.414	4.2	0	0.001	yes	1.51418	1.03823	1.99013	0.135487	0	0.285275
ENSG00000206874	ENSG00000206874	SNORD26	11:626194	316.353	28.919	-2.392	4.047	0	0.001	yes	316.353	0	689.155	28.9186	26.1295	31.7078
ENSG00000226055	ENSG00000226055	PAICSP1	9:3751088	0.436	0.04	-2.379	5.156	0	0	yes	0.435887	0.0340191	0.837755	0.0403932	0.03883	0.041957
ENSG00000162643	ENSG00000162643	WDR63	1:8546482	0.331	0.031	-2.36	11.254	0	0	yes	0.331315	0.227287	0.435344	0.0312811	0.022584	0.039979
ENSG00000230165	ENSG00000230165	CTB-1144G6.5	10:115999	7.864	0.754	-2.345	4.621	0	0	yes	7.86361	3.12149	12.6057	0.753668	0.138301	1.36903
ENSG00000237317	ENSG00000237317	RP4-809F4.1	1:1740844	0.996	0.096	-2.34	6.267	0	0	yes	0.996419	0.27848	1.71436	0.09599	0.077165	0.114815
ENSG00000111780	ENSG00000111780	GATC	12:120875	16.285	1.571	-2.338	5.138	0	0	yes	16.2847	12.0873	20.482	1.57116	0.199486	2.94284
ENSG00000244723	ENSG00000244723	ASLP1	22:239506	0.218	0.022	-2.28	26.344	0	0	yes	0.217937	0.191262	0.244613	0.0222906	0.019562	0.025019
ENSG00000235939	ENSG00000235939	RP11-123B3.2	10:506273	1.754	0.18	-2.274	4.687	0	0	yes	1.75362	0.0654733	3.44177	0.18044	0.158518	0.202361
ENSG00000124920	ENSG00000124920	C11orf9	11:614479	6.545	0.682	-2.261	5.071	0	0	yes	6.54518	5.70639	7.38397	0.682177	0.080161	1.28419
ENSG00000136842	ENSG00000136842	TMOD1	9:1002634	4.629	0.484	-2.258	7.558	0	0	yes	4.62912	3.82934	5.42889	0.484214	0.207318	0.761111
ENSG00000228172	ENSG00000228172	RP1-317E23.3	1:2612666	10.216	1.074	-2.253	3.664	0	0.005	yes	10.216	6.24467	14.1874	1.07391	0	2.32683
ENSG00000240038	ENSG00000240038	AMY2B	1:1040683	5.695	0.599	-2.251	3.907	0	0.002	yes	5.69469	2.51115	8.87822	0.59947	0	1.20366
ENSG00000149089	ENSG00000149089	APIP	11:348746	13.39	1.465	-2.213	7.268	0	0	yes	13.3902	11.048	15.7324	1.46468	0.610332	2.31902
ENSG00000140009	ENSG00000140009	ESR2	14:643196	0.788	0.09	-2.17	6.142	0	0	yes	0.787721	0.41898	1.15646	0.0899757	0.042363	0.137588
ENSG00000214050	ENSG00000214050	FBXO16	8:2820310	20.786	2.379	-2.168	6.89	0	0	yes	20.7858	15.9272	25.6444	2.37908	0.989211	3.76895
ENSG00000120068	ENSG00000120068	HOXB8	17:466845	0.555	0.064	-2.154	11.432	0	0	yes	0.554991	0.375949	0.734033	0.0643983	0.051859	0.076937
ENSG00000253256	ENSG00000253256	CTB-11I22.1	5:1585844	0.085	0.01	-2.145	24.853	0	0	yes	0.0845695	0.07414	0.0949991	0.00989616	0.0087	0.011092
ENSG00000139626	ENSG00000139626	ITGB7	12:535851	7.422	0.881	-2.131	6.777	0	0	yes	7.42219	4.52156	10.3228	0.881039	0.446925	1.31515
ENSG00000233547	ENSG00000233547	RP11-57H14.2	10:114710	0.293	0.035	-2.124	27.256	0	0	yes	0.292525	0.262552	0.322498	0.0349714	0.030864	0.039079
ENSG00000099958	ENSG00000099958	DERL3	22:241291	1.443	0.174	-2.117	3.524	0	0.008	yes	1.44348	0.329431	2.55753	0.173812	0.013794	0.333831
ENSG00000250604	ENSG00000250604	RP11-597D13.8	4:1590456	3.931	0.493	-2.077	5.391	0	0	yes	3.93124	0.91327	6.94921	0.492775	0.460544	0.525006
ENSG00000220739	ENSG00000220739	RP11-277I20.3	6:1446068	1.697	0.213	-2.076	3.683	0	0.004	yes	1.69744	0	3.60712	0.212815	0.196234	0.229395
ENSG00000228775	ENSG00000228775	RP5-894A10.5	7:1414041	12.069	1.526	-2.068	6.327	0	0	yes	12.0694	10.0148	14.124	1.52574	0.562603	2.48887
ENSG00000258232	ENSG00000258232	RP11-161H23.5	12:495785	6.075	0.783	-2.049	3.642	0	0.005	yes	6.0752	3.86754	8.28285	0.782994	0	1.61684
ENSG00000235681	ENSG00000235681	AC008746.5	19:549559	9.122	1.243	-1.993	3.881	0	0.002	yes	9.12152	0	18.4835	1.2429	1.19272	1.29308
ENSG00000235529	ENSG00000235529	AGAP1-IT1	2:2364027	1.628	0.222	-1.992	5.578	0	0	yes	1.628	0.472879	2.78312	0.222202	0.204483	0.239922
ENSG00000164129	ENSG00000164129	NPY5R	4:1642451	3.679	0.503	-1.991	4.559	0	0	yes	3.67878	2.66747	4.69009	0.502546	0.085965	0.919127
ENSG00000184669	ENSG00000184669	RP11-466H18.2	11:167998	6.594	0.902	-1.99	3.589	0	0.006	yes	6.59384	1.29453	11.8932	0.901762	0.213245	1.59028
ENSG00000226363	ENSG00000226363	AC009336.24	2:1769863	0.807	0.115	-1.952	5.265	0	0	yes	0.806851	0.230583	1.38312	0.1146	0.091794	0.137406
ENSG00000248930	ENSG00000248930	CTC-250P20.1-001	5:7225180	1.084	0.155	-1.948	4.009	0	0.001	yes	1.08448	0.042193	2.12676	0.154624	0.132479	0.17677
ENSG00000164241	ENSG00000164241	AC004507.1	5:1263782	6.496	0.94	-1.933	4.045	0	0.001	yes	6.49591	2.33663	10.6552	0.939593	0.27248	1.60671
ENSG00000254140	ENSG00000254140	RP11-731F5.1	14:106064	1.735	0.257	-1.91	3.642	0	0.005	yes	1.73525	0.319154	3.15134	0.257064	0.087816	0.426313
ENSG00000168028	ENSG00000168028	RPSA	3:3944817	108.764	16.116	-1.909	12.332	0	0	yes	108.764	96.1954	121.333	16.1156	11.4858	20.7454
ENSG00000241762	ENSG00000241762	AC002314.3	19:361954	1.166	0.175	-1.899	4.108	0	0.001	yes	1.16571	0.215626	2.1158	0.174551	0.098371	0.250732
ENSG00000255282	ENSG00000255282	RP11-725K16.4	11:102617	2.15	0.328	-1.88	3.918	0	0.002	yes	2.14966	0.722497	3.57682	0.328062	0.100775	0.555349
ENSG00000145040	ENSG00000145040	UCN2	3:4855511	0.074	0.011	-1.873	17.881	0	0	yes	0.0743972	0.0636025	0.0851919	0.0114275	0.0097	0.013155
ENSG00000232626	ENSG00000232626	RP11-175B9.2	1:2023178	2.971	0.459	-1.867	3.608	0	0.006	yes	2.97148	1.89502	4.04795	0.459477	0.01413	0.904823
ENSG00000119703	ENSG00000119703	FAM164C	14:755199	10.295	1.634	-1.841	4.747	0	0	yes	10.2951	7.32895	13.2612	1.63419	0.457685	2.81069
ENSG00000226746	ENSG00000226746	SMCR5	17:175847	0.203	0.033	-1.818	4.331	0	0	yes	0.203313	0.032611	0.374014	0.0329964	0.03244	0.033553
ENSG00000110719	ENSG00000110719	TCIRG1	11:678064	303.607	50.932	-1.785	10.184	0	0	yes	303.607	252.907	354.308	50.9323	35.2305	66.6341
ENSG00000223534	ENSG00000223534	HLA-DQB1-AS1	6:3262724	0.27	0.046	-1.778	23.65	0	0	yes	0.269941	0.242946	0.296935	0.0455955	0.040474	0.050717

ENSG00000180385	ENSG00000180385	AC034193.5	3:1000422	36.92	6.395	-1.753	9.842	0	0	yes	36.9198	30.161	43.6786	6.39469	4.44025	8.34914
ENSG00000124279	ENSG00000124279	FASTKD3	5:7830490	8.195	1.429	-1.746	5.202	0	0	yes	8.19503	6.39848	9.99158	1.42942	0.522344	2.3365
ENSG00000230371	ENSG00000230371	KARSP2	13:113777	0.029	0.005	-1.735	3.964	0	0.002	yes	0.0294834	0.0142582	0.0447085	0.00519893	0.001523	0.008875
ENSG00000164053	ENSG00000164053	ATRIP	3:4848811	13.15	2.36	-1.718	10.74	0	0	yes	13.1496	10.9684	15.3307	2.36011	1.71465	3.00558
ENSG00000139974	ENSG00000139974	SLC38A6	14:612014	55.478	9.977	-1.716	10.091	0	0	yes	55.4781	47.3364	63.6197	9.97678	6.91646	13.0371
ENSG00000159082	ENSG00000159082	SYNJ1	21:339967	22.883	4.123	-1.714	3.579	0	0.006	yes	22.8832	11.603	34.1633	4.12343	0.738114	7.50875
ENSG00000227199	ENSG00000227199	ST7-AS1	7:1165924	0.267	0.048	-1.709	3.696	0	0.004	yes	0.266919	0.022151	0.511686	0.0483484	0.04269	0.054007
ENSG00000138495	ENSG00000138495	COX17	3:1193553	8.081	1.476	-1.7	3.785	0	0.003	yes	8.08109	5.19408	10.9681	1.47591	0.259302	2.69251
ENSG00000258388	ENSG00000258388	XXbac-BPG300A18.12	6:3211613	4.809	0.881	-1.697	5.342	0	0	yes	4.80856	3.43357	6.18354	0.88139	0.38141	1.38137
ENSG00000258136	ENSG00000258136	RP11-864J10.4	12:108126	2.595	0.48	-1.688	4.052	0	0.001	yes	2.59527	1.22852	3.96203	0.479982	0.170201	0.789762
ENSG00000134013	ENSG00000134013	LOXL2	8:2315470	13.169	2.439	-1.686	5.697	0	0	yes	13.1694	9.81376	16.525	2.43855	1.13529	3.74182
ENSG00000163762	ENSG00000163762	TM4SF18	3:1489471	22.081	4.268	-1.643	7.098	0	0	yes	22.0809	17.745	26.4168	4.26847	2.47835	6.0586
ENSG00000188818	ENSG00000188818	ZDHHC11	5:710470-	6.765	1.308	-1.643	3.954	0	0.002	yes	6.76507	3.99297	9.53716	1.30806	0.3621	2.25403
ENSG00000241738	ENSG00000241738	ZNF90P1	3:1010430	0.016	0.003	-1.633	9.557	0	0	yes	0.0155059	0.0117725	0.0192393	0.00302894	0.002294	0.003764
ENSG00000083535	ENSG00000083535	PIBF1	13:733020	32.787	6.444	-1.627	7.618	0	0	yes	32.7873	26.8371	38.7376	6.44361	3.95208	8.93514
ENSG00000251003	ENSG00000251003	RP11-152P17.2	8:1056029	4.437	0.872	-1.627	4.28	0	0	yes	4.43745	2.293	6.58191	0.872254	0.360479	1.38403
ENSG00000114270	ENSG00000114270	COL7A1	3:4860150	29.065	5.785	-1.614	7.213	0	0	yes	29.0651	23.2309	34.8992	5.78497	3.4707	8.09924
ENSG00000127928	ENSG00000127928	GNGT1	7:9322088	19	3.898	-1.584	5.274	0	0	yes	18.9999	14.6244	23.3753	3.89848	1.7357	6.06126
ENSG00000042317	ENSG00000042317	SPATA7	14:888512	23.975	4.934	-1.581	8.538	0	0	yes	23.9745	19.644	28.305	4.93377	3.33885	6.5287
ENSG00000237149	ENSG00000237149	NCRNA00245	10:770295	28.511	5.865	-1.581	8.479	0	0	yes	28.5111	23.3639	33.6582	5.86522	3.95105	7.77939
ENSG00000185278	ENSG00000185278	ZBTB37	1:1738312	9.636	2.034	-1.555	8.307	0	0	yes	9.63578	7.86874	11.4028	2.03404	1.36992	2.69817
ENSG00000208038	ENSG00000208038	MIR492	12:952279	1890.11	401.821	-1.548	5.745	0	0	yes	1890.11	1462.02	2318.19	401.821	205.263	598.38
ENSG00000109475	ENSG00000109475	RPL34	4:1095417	44.562	9.519	-1.544	6.383	0	0	yes	44.5616	35.7264	53.3969	9.5191	5.31971	13.7185
ENSG00000114735	ENSG00000114735	HEMK1	3:5059546	5.22	1.115	-1.544	4.945	0	0	yes	5.21977	3.67959	6.75994	1.11459	0.501185	1.72799
ENSG00000185482	ENSG00000185482	STAC3	12:576372	10.835	2.33	-1.537	5.981	0	0	yes	10.8345	8.54669	13.1224	2.32985	1.23818	3.42152
ENSG00000185633	ENSG00000185633	NDUFA4L2	12:576231	15.954	3.45	-1.531	9.419	0	0	yes	15.9541	13.3605	18.5477	3.44969	2.47812	4.42127
ENSG00000100399	ENSG00000100399	CHADL	22:416012	8.562	1.867	-1.523	5.598	0	0	yes	8.5624	6.86899	10.2558	1.86655	0.920084	2.81302
ENSG00000174748	ENSG00000174748	RPL15	3:2393315	192.602	44.092	-1.474	20.306	0	0	yes	192.602	173.422	211.782	44.0924	39.4324	48.7525
ENSG00000139351	ENSG00000139351	SYCP3	12:102090	0.941	0.218	-1.463	4.451	0	0	yes	0.940636	0.32458	1.55669	0.217798	0.205423	0.230172
ENSG00000227802	ENSG00000227802	DNAJB3	2:2345262	0.002	0.001	-1.461	20.993	0	0	yes	0.00225155	0.00203614	0.00246696	0.00052216	0.000469	0.000575
ENSG00000254208	ENSG00000254208	RP11-219B4.3	8:8608945	0.122	0.028	-1.455	14.104	0	0	yes	0.121523	0.103937	0.13911	0.0283656	0.024195	0.032537
ENSG00000115252	ENSG00000115252	PDE1A	2:1830047	5.704	1.334	-1.453	3.568	0	0.007	yes	5.70444	3.36286	8.04601	1.33442	0.396046	2.2728
ENSG00000166046	ENSG00000166046	TCP11L2	12:106631	47.16	11.105	-1.446	3.545	0	0.007	yes	47.1602	11.4617	82.8586	11.1046	7.72573	14.4836
ENSG00000166181	ENSG00000166181	API5	11:433335	49.6	11.861	-1.431	9.765	0	0	yes	49.6003	41.8889	57.3118	11.8611	8.91492	14.8074
ENSG00000197128	ENSG00000197128	ZNF772	19:579809	3.012	0.73	-1.417	4.461	0	0	yes	3.01181	2.02817	3.99545	0.729938	0.332091	1.12779
ENSG00000137877	ENSG00000137877	SPTBN5	15:421202	0.915	0.222	-1.416	6.216	0	0	yes	0.915274	0.72372	1.10683	0.222036	0.132156	0.311916
ENSG00000000938	ENSG00000000938	FGR	1:2793857	5.893	1.445	-1.406	3.839	0	0.003	yes	5.89346	3.90753	7.87938	1.44463	0.50524	2.38401
ENSG00000142875	ENSG00000142875	PRKACB	1:8454374	18.09	4.45	-1.403	10.209	0	0	yes	18.0904	14.5041	21.6766	4.44986	3.60332	5.29641
ENSG00000111817	ENSG00000111817	DSE	6:1165753	25.54	6.372	-1.388	12.408	0	0	yes	25.5403	21.5957	29.4848	6.37196	5.34004	7.40388
ENSG00000230536	ENSG00000230536	RP11-379C10.4	9:1308235	0.033	0.008	-1.382	33.475	0	0	yes	0.0328582	0.0310459	0.0346704	0.00824886	0.007742	0.008756
ENSG00000020922	ENSG00000020922	MRE11A	11:941528	11.58	2.91	-1.381	3.603	0	0.006	yes	11.5801	3.22346	19.9367	2.90984	2.15578	3.66391
ENSG00000224424	ENSG00000224424	PRKAR2A-AS1	3:4878202	15.135	3.894	-1.358	5.416	0	0	yes	15.1354	11.6676	18.6032	3.89429	2.15803	5.63055
ENSG00000229920	ENSG00000229920	AC016734.3	2:6334851	0.135	0.035	-1.342	31.994	0	0	yes	0.134884	0.127138	0.142629	0.0352537	0.033098	0.037409
ENSG00000231365	ENSG00000231365	RP11-418J17.1	1:1195738	6.348	1.669	-1.336	3.771	0	0.003	yes	6.34803	4.17998	8.51608	1.66923	0.633184	2.70527
ENSG00000156931	ENSG00000156931	VPS8	3:1845299	45.103	11.97	-1.327	6.386	0	0	yes	45.1025	28.629	61.5761	11.9696	9.59972	14.3395
ENSG00000181826	ENSG00000181826	RELL1	4:3745556	13.886	3.687	-1.326	6.674	0	0	yes	13.8863	11.3665	16.4061	3.6871	2.3836	4.99061
ENSG00000247828	ENSG00000247828	CTC-358I24.1	5:8748544	13.217	3.518	-1.324	4.12	0	0.001	yes	13.2166	8.39732	18.036	3.51829	1.65706	5.37953
ENSG00000198003	ENSG00000198003	CCDC151	19:115312	4.428	1.18	-1.322	5.406	0	0	yes	4.42804	3.47168	5.38439	1.18031	0.662233	1.69839
ENSG00000227328	ENSG00000227328	TEKT4P2	21:990718	2.792	0.752	-1.312	3.725	0	0.004	yes	2.79195	1.92203	3.66186	0.751825	0.276888	1.22676
ENSG00000244926	ENSG00000244926	RP11-613D13.7	11:439023	0.223	0.06	-1.31	10.84	0	0	yes	0.222589	0.182863	0.262315	0.0600518	0.050262	0.069841

ENSG00000100600	ENSG00000100600	LGMN	14:931701	73.709	20.151	-1.297	11.106	0	0	yes	73.7089	62.7823	84.6356	20.1513	16.5145	23.7881
ENSG00000185567	ENSG00000185567	AHNAK2	14:105403	1.466	0.402	-1.295	3.919	0	0.002	yes	1.46621	0.690674	2.24174	0.401762	0.242725	0.560799
ENSG00000235423	ENSG00000235423	RP11-282O18.3	12:123636	18.16	5	-1.29	4.299	0	0	yes	18.1602	10.1397	26.1806	4.99984	2.96862	7.03106
ENSG00000118849	ENSG00000118849	RARRES1	3:1584146	0.193	0.054	-1.283	16.998	0	0	yes	0.19318	0.173148	0.213213	0.0535308	0.047655	0.059407
ENSG00000147403	ENSG00000147403	RPL10	X:1536183	127.057	35.298	-1.281	14.346	0	0	yes	127.057	114.563	139.55	35.298	30.0371	40.559
ENSG00000111775	ENSG00000111775	COX6A1	12:120875	58.055	16.138	-1.28	8.405	0	0	yes	58.0548	48.9214	67.1882	16.1383	11.9287	20.3478
ENSG00000131876	ENSG00000131876	SNRPA1	15:101821	17.471	4.86	-1.28	5.495	0	0	yes	17.4709	11.4359	23.5058	4.85985	3.34189	6.37782
ENSG00000151093	ENSG00000151093	OXSM	3:2576043	31.742	8.843	-1.278	9.318	0	0	yes	31.7422	27.2379	36.2466	8.84289	6.76682	10.919
ENSG00000155755	ENSG00000155755	TMEM237	2:2024849	21.226	5.919	-1.277	7.708	0	0	yes	21.2261	17.026	25.4261	5.91876	4.34568	7.49184
ENSG00000130997	ENSG00000130997	POLN	4:2073644	30.19	8.466	-1.271	9.729	0	0	yes	30.1904	24.7912	35.5895	8.46629	6.85268	10.0799
ENSG00000185684	ENSG00000185684	EP400NL	12:132568	43.468	12.289	-1.263	10.302	0	0	yes	43.4676	36.8079	50.1274	12.2892	9.93574	14.6426
ENSG00000254093	ENSG00000254093	PINX1	8:1058127	24.103	6.835	-1.26	5.602	0	0	yes	24.1034	15.0943	33.1126	6.83537	5.12386	8.54688
ENSG00000175147	ENSG00000175147	C1orf126	1:1492519	7.391	2.098	-1.259	8.062	0	0	yes	7.39064	6.27297	8.50831	2.09827	1.52484	2.6717
ENSG00000004766	ENSG00000004766	CCDC132	7:9286165	64.422	18.359	-1.255	10.495	0	0	yes	64.4223	56.5939	72.2508	18.3593	14.5762	22.1423
ENSG00000008130	ENSG00000008130	NADK	1:1682670	132.405	37.896	-1.251	9.874	0	0	yes	132.405	113.615	151.194	37.8964	29.9406	45.8522
ENSG00000114391	ENSG00000114391	RPL24	3:1013999	67.447	19.388	-1.247	7.376	0	0	yes	67.4473	54.7954	80.0991	19.3875	13.9355	24.8394
ENSG00000164142	ENSG00000164142	FAM160A1	4:1523303	9.128	2.669	-1.23	5.337	0	0	yes	9.12793	5.86033	12.3955	2.66924	1.89496	3.44351
ENSG00000207165	ENSG00000207165	SNORA70	X:1536183	8.619	2.53	-1.226	35.845	0	0	yes	8.61949	8.24499	8.99398	2.53028	2.39665	2.66392
ENSG00000258466	ENSG00000258466	RP11-1012A1.4	14:680865	136.321	40.496	-1.214	7.201	0	0	yes	136.321	110.615	162.027	40.4964	29.18	51.8128
ENSG00000113231	ENSG00000113231	PDE8B	5:7650627	4.952	1.477	-1.21	3.973	0	0.002	yes	4.9521	4.02809	5.8761	1.47688	0.620542	2.33321
ENSG00000114395	ENSG00000114395	CYB561D2	3:5038476	80.982	24.294	-1.204	13.216	0	0	yes	80.9819	73.1584	88.8055	24.2939	20.5407	28.0471
ENSG00000129226	ENSG00000129226	CD68	17:748292	8.242	2.533	-1.18	6.221	0	0	yes	8.2416	6.72076	9.76245	2.5326	1.69313	3.37206
ENSG00000160712	ENSG00000160712	IL6R	1:1543748	17.878	5.518	-1.175	4.801	0	0	yes	17.8775	13.7502	22.0049	5.51827	3.13531	7.90123
ENSG00000162244	ENSG00000162244	RPL29	3:5202764	49.486	15.472	-1.163	8.137	0	0	yes	49.4862	41.7862	57.1863	15.4721	11.7634	19.1808
ENSG00000106336	ENSG00000106336	FBXO24	7:1001698	4.809	1.507	-1.161	4.025	0	0.001	yes	4.80913	3.59566	6.02259	1.50655	0.725321	2.28778
ENSG00000011198	ENSG00000011198	ABHD5	3:4332800	27.323	8.643	-1.151	6.328	0	0	yes	27.3233	22.2085	32.4382	8.64344	5.9474	11.3395
ENSG00000152977	ENSG00000152977	ZIC1	3:1471038	28.516	9.018	-1.151	5.827	0	0	yes	28.5161	23.0663	33.966	9.01805	5.89945	12.1367
ENSG00000122432	ENSG00000122432	SPATA1	1:8496400	0.822	0.264	-1.136	11.171	0	0	yes	0.821601	0.694203	0.948998	0.26384	0.229121	0.298559
ENSG00000236939	ENSG00000236939	C8orf56	8:1041332	0.304	0.099	-1.125	29.283	0	0	yes	0.303575	0.287111	0.32004	0.0985945	0.093232	0.103957
ENSG00000219481	ENSG00000219481	NBPF1	1:1688881	31.872	10.419	-1.118	5.208	0	0	yes	31.8723	25.4999	38.2447	10.4189	6.45955	14.3783
ENSG00000235897	ENSG00000235897	TM4SF19-AS1	3:1959383	3.124	1.03	-1.11	3.612	0	0.006	yes	3.12377	1.3516	4.89593	1.02989	0.786796	1.27299
ENSG00000125354	ENSG00000125354	Sep-06	X:1187496	5.614	1.864	-1.102	3.542	0	0.007	yes	5.61351	2.36065	8.86637	1.86398	1.44002	2.28795
ENSG00000184117	ENSG00000184117	NIPSNAP1	22:299018	190.563	63.935	-1.092	16.021	0	0	yes	190.563	174.702	206.425	63.9352	57.0317	70.8387
ENSG00000234062	ENSG00000234062	RP11-308D16.4	X:1359915	9.97	3.347	-1.092	3.751	0	0.003	yes	9.96961	7.078	12.8612	3.34671	1.65802	5.0354
ENSG00000239775	ENSG00000239775	AC017116.11	7:4408423	32.714	11.025	-1.088	6.516	0	0	yes	32.714	27.1647	38.2632	11.0245	7.85471	14.1942
ENSG00000234264	ENSG00000234264	RP4-694A7.4	1:6893983	0.092	0.031	-1.082	4.15	0	0.001	yes	0.0920689	0.0589967	0.125141	0.031203	0.019409	0.042997
ENSG00000142937	ENSG00000142937	RPS8	1:4520548	66.025	22.505	-1.076	10.21	0	0	yes	66.0252	58.381	73.6694	22.5046	18.5393	26.4699
ENSG00000150527	ENSG00000150527	CTAGE5	14:396994	13.876	4.779	-1.066	5.649	0	0	yes	13.8763	10.1372	17.6155	4.77949	3.51679	6.0422
ENSG00000225505	ENSG00000225505	RP11-330C7.3	1:9297425	0.138	0.048	-1.064	7.43	0	0	yes	0.138479	0.110358	0.1666	0.047778	0.038125	0.057431
ENSG00000160746	ENSG00000160746	ANO10	3:4332800	17.335	6.007	-1.06	6.147	0	0	yes	17.3353	14.1107	20.5598	6.00739	4.2631	7.75168
ENSG00000121578	ENSG00000121578	B4GALT4	3:1189305	69.509	24.265	-1.052	8.803	0	0	yes	69.5086	61.9101	77.1071	24.2653	19.1056	29.4251
ENSG00000249319	ENSG00000249319	AC068533.7	7:6554078	10.845	3.792	-1.051	4.492	0	0	yes	10.8445	8.2935	13.3955	3.79211	2.25876	5.32547
ENSG00000152061	ENSG00000152061	RABGAP1L	1:1740844	8.769	3.091	-1.043	3.989	0	0.001	yes	8.76911	6.00896	11.5293	3.09108	1.80079	4.38137
ENSG00000163738	ENSG00000163738	MTHFD2L	4:7497989	11.308	4.023	-1.033	3.733	0	0.004	yes	11.308	6.42496	16.1911	4.0232	2.62928	5.41712
ENSG00000155016	ENSG00000155016	CYP2U1	4:1087457	4.016	1.431	-1.032	5.303	0	0	yes	4.01553	3.20683	4.82423	1.43109	0.954601	1.90758
ENSG00000174791	ENSG00000174791	RIN1	11:660977	5.07	1.828	-1.02	3.738	0	0.004	yes	5.07002	3.63937	6.50068	1.82829	0.974115	2.68246
ENSG00000213865	ENSG00000213865	C8orf44	8:6757983	0.294	0.108	-1.004	4.338	0	0	yes	0.294028	0.205624	0.382432	0.107687	0.069756	0.145619
ENSG00000234353	ENSG00000234353	AP000347.4	22:239506	0.295	0.108	-1.004	11.603	0	0	yes	0.295413	0.259255	0.331571	0.108219	0.094973	0.121466
ENSG00000241839	ENSG00000241839	PLEKHO2	15:651340	3.071	1.129	-1.001	4.773	0	0	yes	3.07126	2.43069	3.71184	1.12864	0.717873	1.5394
ENSG00000105877	ENSG00000105877	DNAH11	7:2158283	0.458	0.168	-1	4.342	0	0	yes	0.458099	0.262116	0.654082	0.168459	0.139622	0.197296

ENSG00000115239	ENSG00000115239	ASB3	2:53759809	73.534	27.221	-0.994	9.698	0	0	yes	73.5337	64.4662	82.6011	27.2205	22.7647	31.6762
ENSG00000177868	ENSG00000177868	CCDC23	1:43272722	32.818	12.257	-0.985	5.539	0	0	yes	32.8182	27.435	38.2015	12.2568	8.38887	16.1246
ENSG00000189050	ENSG00000189050	RNFT1	17:5802960	34.067	12.819	-0.977	7.063	0	0	yes	34.0669	28.3491	39.7847	12.8191	9.99843	15.6399
ENSG00000242247	ENSG00000242247	ARFGAP3	22:4319250	47.362	17.83	-0.977	8.888	0	0	yes	47.3617	42.2627	52.4607	17.8298	14.4123	21.2474
ENSG00000243156	ENSG00000243156	MICAL3	22:1827041	33.752	12.77	-0.972	8.447	0	0	yes	33.7521	30.3464	37.1578	12.7702	10.129	15.4113
ENSG00000078018	ENSG00000078018	MAP2	2:21028878	23.359	8.927	-0.962	4.273	0	0	yes	23.3591	14.8242	31.8939	8.92686	6.57851	11.2752
ENSG00000021762	ENSG00000021762	OSBPL5	11:3108345	57.331	22.34	-0.942	5.93	0	0	yes	57.3312	40.989	73.6735	22.3396	19.1981	25.481
ENSG00000255526	ENSG00000255526	NEDD8-MDP1	14:2465834	35.206	13.746	-0.94	5.66	0	0	yes	35.2055	28.8239	41.587	13.7458	9.91696	17.5746
ENSG00000134508	ENSG00000134508	CABLES1	18:2071452	12.879	5.088	-0.929	7.597	0	0	yes	12.8791	11.1359	14.6224	5.08767	4.05157	6.12377
ENSG00000163291	ENSG00000163291	PAQR3	4:79697495	11.53	4.62	-0.914	4.21	0	0.001	yes	11.5303	9.02432	14.0363	4.62048	2.88224	6.35872
ENSG00000077157	ENSG00000077157	PPP1R12B	1:20231782	25.154	10.179	-0.905	7.044	0	0	yes	25.1535	20.3341	29.973	10.1785	8.43721	11.9198
ENSG00000240950	ENSG00000240950	RP11-372E1.1	3:14253470	24.797	10.068	-0.901	4.818	0	0	yes	24.7971	19.6296	29.9645	10.0684	6.93931	13.1975
ENSG00000237765	ENSG00000237765	FAM200B	4:15606162	30.097	12.256	-0.898	4.896	0	0	yes	30.0968	24.1056	36.0879	12.2555	8.47629	16.0348
ENSG00000137710	ENSG00000137710	RDX	11:1100450	11.982	4.916	-0.891	5.788	0	0	yes	11.9822	9.17763	14.7869	4.91612	3.93301	5.89922
ENSG00000256500	ENSG00000256500	RP11-73M18.2	14:1040197	23.567	9.773	-0.88	4.008	0	0.001	yes	23.5674	17.954	29.1808	9.77252	6.16566	13.3794
ENSG00000172728	ENSG00000172728	FUT10	8:33228342	19.006	7.921	-0.875	4.693	0	0	yes	19.0064	16.1252	21.8876	7.9212	5.22187	10.6205
ENSG00000151466	ENSG00000151466	SCLT1	4:12973077	6.318	2.638	-0.873	4.786	0	0	yes	6.31758	4.93354	7.70163	2.63811	1.86823	3.40798
ENSG00000250644	ENSG00000250644	RP11-295K3.1	11:1753639	9.336	3.9	-0.873	4.304	0	0	yes	9.33586	7.25968	11.412	3.8998	2.57691	5.22269
ENSG00000147316	ENSG00000147316	MCPH1	8:62610714	18.902	7.909	-0.871	5.343	0	0	yes	18.9015	14.7011	23.102	7.90888	6.021	9.79676
ENSG00000115661	ENSG00000115661	STK16	2:22009447	72.449	30.396	-0.869	8.039	0	0	yes	72.4491	64.1438	80.7543	30.3956	24.8279	35.9634
ENSG00000229732	ENSG00000229732	AC019349.5	17:3965723	40.181	16.914	-0.865	4.744	0	0	yes	40.1805	31.8857	48.4753	16.914	11.8274	22.0005
ENSG00000253966	ENSG00000253966	CTC-455F18.3	5:17028887	0.025	0.011	-0.865	8.498	0	0	yes	0.0249819	0.0213947	0.0285691	0.0105177	0.009	0.012036
ENSG00000257337	ENSG00000257337	RP11-983P16.4	12:5339994	42.507	17.906	-0.865	6.217	0	0	yes	42.5069	35.8447	49.169	17.9063	13.7924	22.0202
ENSG00000206535	ENSG00000206535	LNP1	3:10008227	11.374	4.802	-0.862	6.164	0	0	yes	11.3736	9.59159	13.1555	4.80248	3.68961	5.91535
ENSG00000196588	ENSG00000196588	MKL1	22:4080628	36.814	15.723	-0.851	6.844	0	0	yes	36.8136	28.971	44.6562	15.7227	13.7079	17.7376
ENSG00000254501	ENSG00000254501	AP003068.9	11:6485169	20.54	8.779	-0.85	3.835	0	0.003	yes	20.5403	15.6408	25.4398	8.77909	5.49899	12.0592
ENSG00000253950	ENSG00000253950	AC020907.6	19:3548568	25.454	10.947	-0.844	4.798	0	0	yes	25.4543	20.3079	30.6007	10.9467	7.79538	14.0981
ENSG00000079819	ENSG00000079819	EPB41L2	6:13116048	33.855	14.76	-0.83	7.579	0	0	yes	33.8549	27.4601	40.2497	14.7603	13.1225	16.3982
ENSG00000226232	ENSG00000226232	RP11-419C5.2	16:7001019	6.899	3.025	-0.824	3.508	0	0.008	yes	6.89898	5.36178	8.43617	3.0252	1.77341	4.27699
ENSG00000088298	ENSG00000088298	EDEM2	20:3370315	42.499	18.697	-0.821	9.889	0	0	yes	42.4992	38.358	46.6405	18.6974	16.1833	21.2114
ENSG00000149735	ENSG00000149735	GPHA2	11:6468502	0.204	0.09	-0.821	5.06	0	0	yes	0.203949	0.157148	0.25075	0.089741	0.06915	0.110332
ENSG00000128590	ENSG00000128590	DNAJB9	7:10811080	26.773	11.815	-0.818	4.961	0	0	yes	26.773	21.7392	31.8069	11.8147	8.61355	15.0159
ENSG00000218186	ENSG00000218186	KRT8P43	6:24544331	0.028	0.012	-0.817	5.018	0	0	yes	0.0276978	0.0214255	0.0339701	0.0122338	0.00937	0.015098
ENSG00000126522	ENSG00000126522	ASL	7:65540784	263.941	116.99	-0.814	4.565	0	0	yes	263.941	214.609	313.273	116.99	81.4751	152.505
ENSG00000182985	ENSG00000182985	CADM1	11:1150399	47.01	20.84	-0.813	5.107	0	0	yes	47.0102	39.9158	54.1045	20.8401	14.9925	26.6877
ENSG00000065518	ENSG00000065518	NDUFB4	3:12031515	129.774	57.865	-0.808	5.789	0	0	yes	129.774	106.253	153.294	57.8646	45.5881	70.1411
ENSG00000227973	ENSG00000227973	AC023356.3	15:4416290	0.241	0.107	-0.808	7.969	0	0	yes	0.240561	0.207749	0.273374	0.107273	0.091192	0.123354
ENSG00000102312	ENSG00000102312	PORCN	X:48367349	9.944	4.466	-0.8	3.775	0	0.003	yes	9.94368	6.61131	13.2761	4.46614	3.30567	5.62662
ENSG00000149292	ENSG00000149292	TTC12	11:1131852	30.663	13.801	-0.798	4.815	0	0	yes	30.6628	24.3431	36.9825	13.8007	10.216	17.3853
ENSG00000134970	ENSG00000134970	TMED7	5:11491433	61.338	27.638	-0.797	7.116	0	0	yes	61.3384	53.0469	69.6298	27.6379	22.6992	32.5765
ENSG00000117523	ENSG00000117523	PRRC2C	1:17145465	82.301	37.201	-0.794	4.203	0	0.001	yes	82.3005	55.2907	109.31	37.2005	30.2321	44.1689
ENSG00000168291	ENSG00000168291	PDHB	3:58413350	67.141	30.373	-0.793	6.099	0	0	yes	67.1413	56.4923	77.7903	30.3732	24.1114	36.635
ENSG00000145868	ENSG00000145868	FBXO38	5:14776349	54.428	24.695	-0.79	4.684	0	0	yes	54.4277	40.3181	68.5373	24.6948	19.3618	30.0278
ENSG00000123104	ENSG00000123104	ITPR2	12:2627295	15.403	7.01	-0.787	3.977	0	0.001	yes	15.4026	11.6235	19.1817	7.0101	4.83249	9.18771
ENSG00000137817	ENSG00000137817	PARP6	15:7249130	72.619	33.221	-0.782	12.242	0	0	yes	72.619	67.0182	78.2198	33.2205	29.8367	36.6043
ENSG00000232940	ENSG00000232940	HCG25	6:33217310	33.532	15.364	-0.78	4.148	0	0.001	yes	33.5318	26.4102	40.6534	15.3643	10.591	20.1376
ENSG00000255046	ENSG00000255046	RP11-297N6.4	8:11653082	0.098	0.045	-0.777	24.962	0	0	yes	0.0980361	0.0937544	0.102318	0.0450745	0.043075	0.047074
ENSG00000138382	ENSG00000138382	METTL5	2:17064844	144.29	66.389	-0.776	4.166	0	0.001	yes	144.29	114.74	173.84	66.3891	45.7192	87.059
ENSG00000165238	ENSG00000165238	WNK2	9:95947197	25.71	11.83	-0.776	7.505	0	0	yes	25.7101	21.3679	30.0523	11.83	10.4169	13.2431
ENSG00000256029	ENSG00000256029	RP11-190A12.7	1:15979652	0.283	0.131	-0.775	3.843	0	0.002	yes	0.283478	0.198006	0.36895	0.130662	0.095708	0.165616

ENSG00000020577	ENSG00000020577	SAMD4A	14:550338	12.035	5.591	-0.767	3.713	0	0.004	yes	12.035	8.27043	15.7995	5.59066	4.08335	7.09796
ENSG00000157657	ENSG00000157657	ZNF618	9:1166385	46.468	21.628	-0.765	8.221	0	0	yes	46.4675	41.8119	51.1232	21.6281	18.2372	25.019
ENSG00000147813	ENSG00000147813	NAPRT1	8:1446556	34.856	16.358	-0.757	4.502	0	0	yes	34.8555	29.0234	40.6876	16.3578	11.5905	21.1251
ENSG00000117983	ENSG00000117983	MUC5B	11:124429	55.709	26.33	-0.749	5.488	0	0	yes	55.7088	47.2644	64.1532	26.3304	20.3494	32.3115
ENSG00000137642	ENSG00000137642	SORL1	11:121318	18.52	8.774	-0.747	4.179	0	0.001	yes	18.5196	14.9394	22.0999	8.77364	6.13507	11.4122
ENSG00000256762	ENSG00000256762	STH	17:439717	0.052	0.025	-0.742	16.628	0	0	yes	0.0520904	0.0489898	0.0551909	0.0248001	0.02315	0.02645
ENSG00000240224	ENSG00000240224	UGT1A5	2:2345262	8.098	3.874	-0.737	5.01	0	0	yes	8.09768	6.64731	9.54804	3.87448	2.96985	4.77911
ENSG00000168000	ENSG00000168000	BSCL2	11:624577	366.222	175.794	-0.734	3.572	0	0.007	yes	366.222	276.746	455.698	175.794	117.714	233.873
ENSG00000134333	ENSG00000134333	LDHA	11:184159	196.046	94.509	-0.73	9.51	0	0	yes	196.046	175.376	216.716	94.5092	83.9722	105.046
ENSG00000198301	ENSG00000198301	SDAD1	4:7683180	37.783	18.36	-0.722	5.61	0	0	yes	37.7827	31.5638	44.0015	18.3596	14.7284	21.9907
ENSG00000197728	ENSG00000197728	RPS26	12:564356	118.735	57.726	-0.721	7.453	0	0	yes	118.735	104.007	133.464	57.7255	49.151	66.3
ENSG00000128335	ENSG00000128335	APOL2	22:366222	56.205	27.497	-0.715	8.027	0	0	yes	56.205	49.4869	62.9232	27.4969	23.8657	31.1282
ENSG00000235109	ENSG00000235109	ZNF323	6:2829246	13.809	6.798	-0.709	3.829	0	0.003	yes	13.8088	10.4076	17.21	6.79829	4.92005	8.67653
ENSG00000114942	ENSG00000114942	EEF1B2	2:2069802	40.934	20.315	-0.701	5.434	0	0	yes	40.9341	35.0871	46.7812	20.3152	15.9543	24.676
ENSG00000174374	ENSG00000174374	WBSCR16	7:7444122	43.123	21.396	-0.701	7.22	0	0	yes	43.1233	37.2765	48.9702	21.3963	18.4233	24.3693
ENSG00000137843	ENSG00000137843	PAK6	15:404532	15.432	7.663	-0.7	8.242	0	0	yes	15.4321	13.7759	17.0883	7.66311	6.65416	8.67205
ENSG00000183020	ENSG00000183020	AP2A2	11:924893	137.772	68.483	-0.699	5.89	0	0	yes	137.772	117.834	157.709	68.4833	55.5994	81.3673
ENSG00000135631	ENSG00000135631	RAB11FIP5	2:7316916	46.407	23.085	-0.698	5.699	0	0	yes	46.407	39.6275	53.1865	23.0848	18.5426	27.6269
ENSG00000240854	ENSG00000240854	RP11-64K7.1	3:1312523	23.913	11.941	-0.694	5.433	0	0	yes	23.9132	20.1397	27.6867	11.9411	9.53919	14.343
ENSG00000235706	ENSG00000235706	AL356017.3	14:955525	29.299	14.753	-0.686	5.374	0	0	yes	29.2988	24.955	33.6426	14.753	11.6859	17.8202
ENSG00000135916	ENSG00000135916	ITM2C	2:2317293	328.54	165.694	-0.685	12.053	0	0	yes	328.54	302.852	354.228	165.694	152.043	179.345
ENSG00000162458	ENSG00000162458	FBLIM1	1:1608310	30.319	15.277	-0.685	3.977	0	0.001	yes	30.3193	24.0781	36.5606	15.2767	11.0522	19.5012
ENSG00000110011	ENSG00000110011	DNAJC4	11:639741	108.793	54.918	-0.684	5.313	0	0	yes	108.793	91.723	125.863	54.918	43.7166	66.1195
ENSG00000213782	ENSG00000213782	DDX47	12:128788	16.731	8.495	-0.678	4.039	0	0.001	yes	16.7307	13.7323	19.7291	8.49533	6.08517	10.9055
ENSG00000111252	ENSG00000111252	SH2B3	12:111843	5.86	2.977	-0.677	4.168	0	0.001	yes	5.85959	4.76303	6.95615	2.97743	2.18691	3.76795
ENSG00000145907	ENSG00000145907	G3BP1	5:1511218	131.669	66.97	-0.676	9.263	0	0	yes	131.669	118.018	145.321	66.9697	60.0886	73.8509
ENSG00000154146	ENSG00000154146	NRGN	11:124609	12.139	6.193	-0.673	4.219	0	0.001	yes	12.1385	9.84958	14.4275	6.19281	4.59898	7.78664
ENSG00000203896	ENSG00000203896	LIME1	20:622891	32.328	16.504	-0.672	3.585	0	0.006	yes	32.3283	25.6996	38.957	16.5039	11.3208	21.6869
ENSG00000240682	ENSG00000240682	ISY1	3:1288064	35.619	18.347	-0.663	5.798	0	0	yes	35.6186	30.7083	40.5289	18.3466	14.9951	21.698
ENSG00000196670	ENSG00000196670	ZFP62	5:1802746	39.378	20.306	-0.662	4.73	0	0	yes	39.3775	29.8669	48.8881	20.3057	17.427	23.1844
ENSG00000166278	ENSG00000166278	C2	6:3186556	17.216	8.91	-0.659	3.626	0	0.005	yes	17.2157	13.1051	21.3263	8.91032	6.47046	11.3502
ENSG00000127080	ENSG00000127080	IPPK	9:9505963	13.269	6.95	-0.647	4.477	0	0	yes	13.2687	10.9993	15.5381	6.94975	5.33152	8.56799
ENSG00000162441	ENSG00000162441	LZIC	1:9982170	19.174	10.087	-0.642	4.075	0	0.001	yes	19.174	15.4925	22.8556	10.0873	7.56541	12.6092
ENSG00000121671	ENSG00000121671	CRY2	11:458686	69.161	36.72	-0.633	3.758	0	0.003	yes	69.1606	60.1812	78.1401	36.7203	25.3045	48.1361
ENSG00000138297	ENSG00000138297	TIMM23	10:513713	25.333	13.529	-0.627	4.172	0	0.001	yes	25.3326	20.6548	30.0105	13.5291	10.3183	16.7399
ENSG00000170860	ENSG00000170860	LSM3	3:1418631	4.716	2.526	-0.624	4.315	0	0	yes	4.71642	3.86692	5.56592	2.52631	1.95429	3.09834
ENSG00000247372	ENSG00000247372	CTD-2235C13.2	5:7461597	0.264	0.142	-0.622	15.035	0	0	yes	0.264334	0.248977	0.279692	0.141961	0.133607	0.150315
ENSG00000119414	ENSG00000119414	PPP6C	9:1279088	76.461	41.321	-0.615	6.752	0	0	yes	76.4609	67.8622	85.0595	41.3211	35.3924	47.2497
ENSG00000215450	ENSG00000215450	RP5-890O15.3	20:455232	0.107	0.058	-0.614	9.131	0	0	yes	0.106832	0.0967372	0.116926	0.0578419	0.052314	0.06337
ENSG00000010322	ENSG00000010322	NISCH	3:5248913	76.101	41.229	-0.613	6.052	0	0	yes	76.1005	64.0777	88.1233	41.2285	36.0016	46.4555
ENSG00000137522	ENSG00000137522	RNF121	11:716397	79.681	43.236	-0.611	5.176	0	0	yes	79.6811	65.6259	93.7362	43.2364	36.4428	50.0299
ENSG00000142330	ENSG00000142330	CAPN10	2:2415261	15.714	8.528	-0.611	4.164	0	0.001	yes	15.7135	12.5463	18.8808	8.52774	6.70738	10.3481
ENSG00000100442	ENSG00000100442	FKBP3	14:455533	42.53	23.128	-0.609	5.307	0	0	yes	42.5304	36.4306	48.6302	23.1282	18.982	27.2745
ENSG00000131508	ENSG00000131508	UBE2D2	5:1389060	191.105	104.488	-0.604	4.378	0	0	yes	191.105	161.545	220.666	104.488	80.628	128.347
ENSG00000072201	ENSG00000072201	LNX1	4:5424380	17.546	9.603	-0.603	3.991	0	0.001	yes	17.5458	14.7783	20.3133	9.60287	7.12942	12.0763
ENSG00000123388	ENSG00000123388	HOXC11	12:543560	11.539	6.33	-0.6	4.574	0	0	yes	11.5387	9.62472	13.4526	6.33033	5.04235	7.61831
ENSG00000145592	ENSG00000145592	RPL37	5:4082536	459.234	253.026	-0.596	7.967	0	0	yes	459.234	416.473	501.995	253.026	223.389	282.663
ENSG00000178922	ENSG00000178922	HYI	1:4388642	106.079	58.461	-0.596	7.217	0	0	yes	106.079	95.7307	116.427	58.4612	50.6727	66.2497
ENSG00000182909	ENSG00000182909	LENG9	19:549559	16.957	9.394	-0.591	4.845	0	0	yes	16.9574	14.3509	19.5639	9.39438	7.61674	11.172
ENSG00000120159	ENSG00000120159	C9orf82	9:2684068	33.889	18.823	-0.588	4.777	0	0	yes	33.889	27.8673	39.9106	18.823	15.6153	22.0308

ENSG00000136383	ENSG00000136383	ALPK3	15:853599	1.21	0.675	-0.584	3.731	0	0.004	yes	1.21035	0.973055	1.44765	0.674759	0.509924	0.839595
ENSG00000170421	ENSG00000170421	KRT8	12:532909	1083.56	606.012	-0.581	11.237	0	0	yes	1083.56	999.646	1167.47	606.012	564.468	647.557
ENSG00000141027	ENSG00000141027	NCOR1	17:158798	114.845	64.88	-0.571	4.648	0	0	yes	114.845	97.4441	132.245	64.8796	52.3291	77.4301
ENSG00000176973	ENSG00000176973	FAM89B	11:653379	44.388	25.134	-0.569	6.515	0	0	yes	44.3884	39.3996	49.3772	25.1342	21.7761	28.4922
ENSG00000189269	ENSG00000189269	C22orf43	22:239506	0.063	0.036	-0.567	6.552	0	0	yes	0.0627378	0.0550588	0.0704168	0.0355846	0.031229	0.03994
ENSG00000167491	ENSG00000167491	GATAD2A	19:194966	205.321	116.725	-0.565	9.523	0	0	yes	205.321	189.148	221.493	116.725	106.375	127.075
ENSG00000198211	ENSG00000198211	TUBB3	16:899785	22.568	12.828	-0.565	4.053	0	0.001	yes	22.5679	19.2008	25.9349	12.8277	9.80658	15.8488
ENSG00000089101	ENSG00000089101	C20orf26	20:200150	0.001	0.001	-0.555	8.176	0	0	yes	0.00112768	0.00101394	0.00124141	0.000647319	0.000588	0.000706
ENSG00000179222	ENSG00000179222	MAGED1	X:5154610	356.326	205.229	-0.552	10.915	0	0	yes	356.326	332.425	380.226	205.229	189.706	220.753
ENSG00000113621	ENSG00000113621	TXNDC15	5:1342094	59.404	34.387	-0.547	4.207	0	0.001	yes	59.4043	48.1194	70.6891	34.387	28.2887	40.4854
ENSG00000116874	ENSG00000116874	WARS2	1:1195738	20.727	12.033	-0.544	3.619	0	0.006	yes	20.7272	17.1533	24.3011	12.0333	9.0721	14.9945
ENSG00000138641	ENSG00000138641	HERC3	4:8944213	50.005	29.053	-0.543	5.081	0	0	yes	50.0048	43.5811	56.4286	29.0532	24.0905	34.016
ENSG00000197958	ENSG00000197958	RPL12	9:1302099	357.263	207.537	-0.543	8.24	0	0	yes	357.263	326.414	388.113	207.537	186.861	228.214
ENSG00000005022	ENSG00000005022	SLC25A5	X:1185999	330.8	193.511	-0.536	8.68	0	0	yes	330.8	303.554	358.047	193.511	175.692	211.331
ENSG00000077782	ENSG00000077782	FGFR1	8:3826865	56.541	33.196	-0.533	3.6	0	0.006	yes	56.5408	46.1743	66.9073	33.1957	25.4893	40.9021
ENSG00000232485	ENSG00000232485	AC098820.3	2:2172500	0.184	0.108	-0.532	15.394	0	0	yes	0.184203	0.175903	0.192503	0.108248	0.102582	0.113914
ENSG00000159884	ENSG00000159884	CCDC107	9:3565830	22.05	13.013	-0.527	3.873	0	0.002	yes	22.0496	18.4832	25.6159	13.0132	10.1626	15.8638
ENSG00000137055	ENSG00000137055	PLAA	9:2690408	17.238	10.19	-0.526	3.557	0	0.007	yes	17.2379	14.2892	20.1867	10.1904	7.73425	12.6466
ENSG00000155508	ENSG00000155508	CNOT8	5:1541980	138.936	82.111	-0.526	8.452	0	0	yes	138.936	127.891	149.982	82.1108	74.249	89.9726
ENSG00000184182	ENSG00000184182	UBE2F	2:2388754	59.871	35.411	-0.525	5.191	0	0	yes	59.8713	52.1328	67.6097	35.4114	29.8992	40.9237
ENSG00000229212	ENSG00000229212	AC044860.3	15:857471	0.004	0.003	-0.525	10.675	0	0	yes	0.00449926	0.00420418	0.00479434	0.0026614	0.002466	0.002857
ENSG00000151491	ENSG00000151491	EPS8	12:157730	95.135	56.376	-0.523	4.094	0	0.001	yes	95.1348	74.5608	115.709	56.3759	48.6933	64.0585
ENSG00000139154	ENSG00000139154	AEBP2	12:195569	29.591	17.561	-0.522	4.833	0	0	yes	29.5905	25.5014	33.6797	17.5614	14.6476	20.4753
ENSG00000161980	ENSG00000161980	POLR3K	16:96406-1	52.566	31.195	-0.522	5.496	0	0	yes	52.5655	46.1634	58.9676	31.1952	26.6512	35.7393
ENSG00000168724	ENSG00000168724	DNAJC21	5:3492969	19.737	11.709	-0.522	4.987	0	0	yes	19.7372	17.0731	22.4013	11.7089	9.83445	13.5833
ENSG00000152464	ENSG00000152464	RPP38	10:151373	26.531	15.798	-0.518	4.383	0	0	yes	26.5307	22.7236	30.3379	15.7979	12.8264	18.7693
ENSG00000185885	ENSG00000185885	IFITM1	11:307630	70.098	41.771	-0.518	5.159	0	0	yes	70.0979	61.9707	78.2252	41.7712	34.9283	48.6141
ENSG00000162623	ENSG00000162623	TYW3	1:7501808	23.637	14.127	-0.515	4.788	0	0	yes	23.6371	20.7728	26.5013	14.127	11.6176	16.6363
ENSG00000142669	ENSG00000142669	SH3BGRL3	1:2660566	122.208	73.079	-0.514	6.065	0	0	yes	122.208	108.356	136.061	73.0789	63.863	82.2949
ENSG00000240053	ENSG00000240053	LY6G5B	6:3162900	32.205	19.265	-0.514	4.898	0	0	yes	32.2051	28.5024	35.9078	19.2654	15.8847	22.646
ENSG00000006740	ENSG00000006740	ARHGAP44	17:126928	7.363	4.407	-0.513	4.859	0	0	yes	7.36255	6.39	8.3351	4.40662	3.68	5.13324
ENSG00000161960	ENSG00000161960	EIF4A1	17:747602	178.636	107.233	-0.51	8.016	0	0	yes	178.636	163.497	193.775	107.233	97.0433	117.424
ENSG00000072210	ENSG00000072210	ALDH3A2	17:195514	97.54	58.642	-0.509	4.127	0	0.001	yes	97.5401	86.9337	108.146	58.6421	45.663	71.6211
ENSG00000172113	ENSG00000172113	NME6	3:4828258	38.907	23.401	-0.508	3.993	0	0.001	yes	38.9072	33.3797	44.4346	23.4005	18.4543	28.3468
ENSG00000041802	ENSG00000041802	LSG1	3:1943047	93.899	56.712	-0.504	6.684	0	0	yes	93.8991	85.2387	102.56	56.7123	49.9406	63.4841
ENSG00000172954	ENSG00000172954	LCLAT1	2:3067009	34.498	20.832	-0.504	3.924	0	0.002	yes	34.4976	28.4521	40.543	20.8318	16.9139	24.7497
ENSG00000065923	ENSG00000065923	SLC9A7	X:4646475	44.675	27.051	-0.502	3.855	0	0.002	yes	44.6746	37.061	52.2883	27.0512	21.7291	32.3733
ENSG00000133316	ENSG00000133316	WDR74	11:625997	92.857	56.363	-0.499	6.023	0	0	yes	92.8569	81.7185	103.995	56.3631	49.9142	62.812
ENSG00000165675	ENSG00000165675	ENOX2	X:1297517	23.799	14.462	-0.498	3.819	0	0.003	yes	23.7985	19.8974	27.6997	14.4619	11.5273	17.3965
ENSG00000196357	ENSG00000196357	ZNF565	19:366731	13.697	8.339	-0.496	4.302	0	0	yes	13.6967	11.7113	15.6821	8.33937	6.84285	9.83589
ENSG00000134825	ENSG00000134825	C11orf10	11:614479	110.991	67.689	-0.495	4.546	0	0	yes	110.991	96.1714	125.81	67.6885	56.0616	79.3154
ENSG00000181163	ENSG00000181163	NPM1	5:1708141	1023.6	623.678	-0.495	6.049	0	0	yes	1023.6	918.371	1128.83	623.678	544.146	703.211
ENSG00000224660	ENSG00000224660	AC087590.3	3:1529569	28.742	17.525	-0.495	3.599	0	0.006	yes	28.7422	23.8807	33.6038	17.5246	13.7254	21.3238
ENSG00000182944	ENSG00000182944	EWSR1	22:296558	133.792	82.072	-0.489	8.489	0	0	yes	133.792	123.355	144.228	82.0717	75.1216	89.0218
ENSG00000131469	ENSG00000131469	RPL27	17:411504	294.74	181.163	-0.487	5.386	0	0	yes	294.74	259.861	329.619	181.163	156.418	205.909
ENSG00000108298	ENSG00000108298	RPL19	17:373565	151.998	93.909	-0.482	5.335	0	0	yes	151.998	134.015	169.98	93.9094	81.1033	106.716
ENSG00000106682	ENSG00000106682	EIF4H	7:7358857	47.145	29.349	-0.474	3.743	0	0.004	yes	47.1453	39.3327	54.9579	29.3491	23.7295	34.9686
ENSG00000171346	ENSG00000171346	KRT15	17:396699	22.433	13.998	-0.472	4.376	0	0	yes	22.4334	19.3828	25.484	13.9976	11.6566	16.3387
ENSG00000124444	ENSG00000124444	ZNF576	19:441005	34.292	21.454	-0.469	3.972	0	0.002	yes	34.292	29.1044	39.4797	21.4543	17.5633	25.3453
ENSG00000126217	ENSG00000126217	MCF2L	13:113548	54.66	34.201	-0.469	4.564	0	0	yes	54.66	48.5819	60.7381	34.2009	28.2911	40.1107

ENSG00000161800	ENSG00000161800	RACGAP1	12:503609	42.455	26.576	-0.468	4.348	0	0	yes		42.4553	36.3109	48.5998	26.5762	22.334	30.8184
ENSG00000168575	ENSG00000168575	SLC20A2	8:4227399	53.668	33.638	-0.467	4.831	0	0	yes		53.6684	46.9611	60.3757	33.6377	28.6733	38.6021
ENSG00000206053	ENSG00000206053	HN1L	16:166233	232.065	145.737	-0.465	8.927	0	0	yes		232.065	215.991	248.139	145.737	134.387	157.086
ENSG00000122778	ENSG00000122778	KIAA1549	7:1383910	6.317	3.971	-0.464	4.714	0	0	yes		6.31745	5.32008	7.31481	3.97111	3.50332	4.43891
ENSG00000099901	ENSG00000099901	RANBP1	22:200677	36.658	23.09	-0.462	4.349	0	0	yes		36.6578	31.4239	41.8916	23.0904	19.4541	26.7267
ENSG00000095139	ENSG00000095139	ARCN1	11:118307	173.509	109.376	-0.461	9.529	0	0	yes		173.509	163.244	183.774	109.376	100.989	117.764
ENSG00000132394	ENSG00000132394	EEFSEC	3:1277704	32.783	20.744	-0.458	4.51	0	0	yes		32.7825	28.597	36.968	20.7436	17.4709	24.0164
ENSG00000139163	ENSG00000139163	ETNK1	12:227780	21.062	13.349	-0.456	3.761	0	0.003	yes		21.0621	17.3744	24.7498	13.3485	11.1086	15.5884
ENSG00000171853	ENSG00000171853	TTC15	2:3383445	180.861	114.746	-0.455	6.348	0	0	yes		180.861	160.79	200.932	114.746	104.332	125.16
ENSG00000055609	ENSG00000055609	MLL3	7:1518320	14.2	9.018	-0.454	3.629	0	0.005	yes		14.1995	11.8148	16.5841	9.01792	7.34531	10.6905
ENSG00000090006	ENSG00000090006	LTBP4	19:410990	66.974	42.571	-0.453	8.452	0	0	yes		66.9739	61.9162	72.0316	42.5709	39.3304	45.8114
ENSG00000089022	ENSG00000089022	MAPKAPK5	12:112277	37.663	24.044	-0.449	4.654	0	0	yes		37.663	33.7491	41.5768	24.0443	20.1378	27.9508
ENSG00000163900	ENSG00000163900	TMEM41A	3:1850007	13.435	8.576	-0.449	3.498	0	0.008	yes		13.4352	10.9937	15.8767	8.57573	7.02097	10.1305
ENSG00000241279	ENSG00000241279	AC110615.1	4:7678101	98.509	62.87	-0.449	3.875	0	0.002	yes		98.5093	83.7164	113.302	62.8698	51.7683	73.9713
ENSG00000250012	ENSG00000250012	RP11-124N2.1	3:1257251	0.193	0.123	-0.448	6.414	0	0	yes		0.192771	0.174328	0.211214	0.123123	0.110573	0.135673
ENSG00000168066	ENSG00000168066	SF1	11:645320	584.34	373.814	-0.447	11.516	0	0	yes		584.34	554.071	614.61	373.814	352.223	395.404
ENSG00000110171	ENSG00000110171	TRIM3	11:646984	127.786	81.948	-0.444	5.475	0	0	yes		127.786	112.926	142.647	81.9475	72.669	91.226
ENSG00000075292	ENSG00000075292	ZNF638	2:7150369	114.804	73.746	-0.443	6.325	0	0	yes		114.804	103.888	125.72	73.7459	66.1724	81.3193
ENSG00000134759	ENSG00000134759	ELP2	18:337094	67.406	43.318	-0.442	4.744	0	0	yes		67.4061	59.0699	75.7422	43.3181	37.2755	49.3607
ENSG00000172936	ENSG00000172936	MYD88	3:3817996	133.25	85.798	-0.44	7.87	0	0	yes		133.25	121.914	144.585	85.7979	79.5629	92.0328
ENSG00000164542	ENSG00000164542	KIAA0895	7:3636382	23.036	14.852	-0.439	3.5	0	0.008	yes		23.0355	18.9295	27.1414	14.8515	12.2313	17.4717
ENSG00000258930	ENSG00000258930	RP5-1163L11.2	14:713741	1.036	0.67	-0.436	9.705	0	0	yes		1.03642	0.973862	1.09897	0.670359	0.625792	0.714926
ENSG00000206612	ENSG00000206612	SNORA2A	12:490471	11.249	7.282	-0.435	6.371	0	0	yes		11.2491	10.1728	12.3253	7.28243	6.57342	7.99143
ENSG00000175309	ENSG00000175309	AGXT2L2	5:1776315	131.311	85.373	-0.431	6.956	0	0	yes		131.311	121.656	140.966	85.3732	76.8707	93.8758
ENSG00000107165	ENSG00000107165	TYRP1	9:1268543	53.706	34.963	-0.429	3.701	0	0.004	yes		53.7056	45.4455	61.9657	34.9629	28.8927	41.033
ENSG00000130714	ENSG00000130714	POMT1	9:1343782	60.949	39.723	-0.428	4.633	0	0	yes		60.9492	54.2637	67.6348	39.7232	33.8155	45.6308
ENSG00000145022	ENSG00000145022	TCTA	3:4939657	26.444	17.233	-0.428	3.795	0	0.003	yes		26.4444	22.5486	30.3402	17.2331	14.2872	20.179
ENSG00000175768	ENSG00000175768	TOMM5	9:3751088	95.123	62.025	-0.428	4.881	0	0	yes		95.1228	85.0285	105.217	62.0245	53.3769	70.6721
ENSG00000185728	ENSG00000185728	YTHDF3	8:6408111	41.894	27.307	-0.428	4.162	0	0.001	yes		41.8944	36.3096	47.4792	27.3074	23.0308	31.584
ENSG00000130294	ENSG00000130294	KIF1A	2:2416531	76.09	49.733	-0.425	3.506	0	0.008	yes		76.0899	61.7445	90.4353	49.7334	42.1421	57.3247
ENSG00000112419	ENSG00000112419	PHACTR2	6:1438579	24.441	15.99	-0.424	3.694	0	0.004	yes		24.4407	20.556	28.3254	15.9903	13.3384	18.6422
ENSG00000167608	ENSG00000167608	TMC4	19:546638	198.977	130.242	-0.424	5.123	0	0	yes		198.977	173.784	224.17	130.242	116.371	144.112
ENSG00000080815	ENSG00000080815	PSEN1	14:736031	138.963	91.106	-0.422	5.424	0	0	yes		138.963	126.048	151.879	91.1063	79.7295	102.483
ENSG00000153936	ENSG00000153936	HS2ST1	1:8738033	8.608	5.647	-0.422	3.542	0	0.007	yes		8.60822	7.32794	9.8885	5.64693	4.59714	6.69671
ENSG00000196712	ENSG00000196712	NF1	17:294219	34.065	22.511	-0.414	3.49	0	0.009	yes		34.0651	30.1863	37.9439	22.5109	17.8207	27.2011
ENSG00000171401	ENSG00000171401	KRT13	17:396572	424.673	280.95	-0.413	8.744	0	0	yes		424.673	398.538	450.807	280.95	260.801	301.099
ENSG00000198492	ENSG00000198492	YTHDF2	1:2906313	90.236	59.708	-0.413	3.552	0	0.007	yes		90.2361	76.101	104.371	59.708	49.4489	69.9671
ENSG00000075151	ENSG00000075151	EIF4G3	1:2113297	79.434	52.653	-0.411	5.567	0	0	yes		79.4336	71.4956	87.3717	52.6526	46.9241	58.3811
ENSG00000153046	ENSG00000153046	CDYL	6:4706344	25.657	17.015	-0.411	4.658	0	0	yes		25.6571	22.5061	28.808	17.0148	14.8613	19.1684
ENSG00000204618	ENSG00000204618	RNF39	6:3003448	21.949	14.588	-0.409	3.733	0	0.004	yes		21.9487	18.9341	24.9632	14.588	12.1022	17.0738
ENSG00000078687	ENSG00000078687	TNRC6C	17:760002	20.719	13.781	-0.408	4.211	0	0.001	yes		20.7191	17.9064	23.5318	13.7808	11.8769	15.6847
ENSG00000138061	ENSG00000138061	CYP1B1	2:3815032	60.375	40.213	-0.406	3.752	0	0.003	yes		60.3745	52.0558	68.6932	40.2129	33.4907	46.9352
ENSG00000133027	ENSG00000133027	PEMT	17:174088	209.161	139.705	-0.404	4.487	0	0	yes		209.161	188.603	229.719	139.705	118.655	160.754
ENSG00000109452	ENSG00000109452	INPP4B	4:1429443	90.791	60.673	-0.403	3.832	0	0.003	yes		90.7913	81.811	99.7717	60.6732	49.4099	71.9365
ENSG00000196208	ENSG00000196208	GREB1	2:1167424	46.778	31.369	-0.4	3.734	0	0.004	yes		46.7775	41.6344	51.9206	31.3688	25.6083	37.1294
ENSG00000156170	ENSG00000156170	C8orf38	8:9582553	75.834	51.069	-0.395	5.258	0	0	yes		75.8344	68.2014	83.4675	51.0694	45.3624	56.7764
ENSG00000113645	ENSG00000113645	WWC1	5:1677186	864.398	582.949	-0.394	7.153	0	0	yes		864.398	801.285	927.511	582.949	534.873	631.025
ENSG00000115204	ENSG00000115204	MPV17	2:2750525	186.979	126.1	-0.394	6.246	0	0	yes		186.979	172.609	201.348	126.1	113.488	138.712
ENSG00000129083	ENSG00000129083	COPB1	11:144649	146.958	99.071	-0.394	5.721	0	0	yes		146.958	134.18	159.737	99.0709	88.4724	109.669
ENSG00000167397	ENSG00000167397	VKORC1	16:310857	127.567	86.054	-0.394	5.24	0	0	yes		127.567	116.295	138.84	86.0541	75.5953	96.5129

ENSG00000145741	ENSG00000145741	BTF3	5:7279423	191.99	129.861	-0.391	4.149	0	0.001	yes	191.99	167.494	216.486	129.861	111.844	147.878
ENSG00000135414	ENSG00000135414	GDF11	12:5613700	13.948	9.461	-0.388	3.498	0	0.008	yes	13.9477	11.8894	16.006	9.46059	7.89226	11.0289
ENSG00000142541	ENSG00000142541	RPL13A	19:499908	355.581	241.819	-0.386	6.765	0	0	yes	355.581	332.726	378.435	241.819	219.054	264.583
ENSG00000164742	ENSG00000164742	ADCY1	7:4561373	17.177	11.68	-0.386	4.093	0	0.001	yes	17.1766	14.8683	19.4849	11.6797	10.1366	13.2229
ENSG00000148344	ENSG00000148344	PTGES	9:1325006	52.179	35.492	-0.385	4.273	0	0	yes	52.1791	45.9758	58.3823	35.4918	30.6772	40.3064
ENSG00000197381	ENSG00000197381	ADARB1	21:464916	19.036	12.993	-0.382	4.984	0	0	yes	19.0356	17.2641	20.8072	12.9925	11.4104	14.5746
ENSG00000008710	ENSG00000008710	PKD1	16:208981	93.522	63.877	-0.381	5.423	0	0	yes	93.5217	84.76	102.283	63.8772	57.1795	70.5748
ENSG00000230259	ENSG00000230259	AC008738.2	19:337908	25.148	17.172	-0.381	3.772	0	0.003	yes	25.1483	21.7929	28.5038	17.1723	14.5615	19.783
ENSG00000136044	ENSG00000136044	APPL2	12:105567	99.533	68.044	-0.38	4.493	0	0	yes	99.5329	88.7033	110.362	68.0438	59.2178	76.8697
ENSG00000117419	ENSG00000117419	ERI3	1:4468674	89.66	61.506	-0.377	4.261	0	0	yes	89.6595	77.6982	101.621	61.5058	54.3624	68.6492
ENSG00000177192	ENSG00000177192	PUS1	12:132409	65.884	45.303	-0.375	3.683	0	0.004	yes	65.8837	59.3146	72.4529	45.3029	37.2737	53.3321
ENSG00000068724	ENSG00000068724	TTC7A	2:4712641	43.402	29.941	-0.371	4.295	0	0	yes	43.4022	38.9982	47.8062	29.9413	25.7508	34.1318
ENSG00000112715	ENSG00000112715	VEGFA	6:4373792	121.115	83.682	-0.37	5.087	0	0	yes	121.115	110.062	132.167	83.6822	74.216	93.1484
ENSG00000140961	ENSG00000140961	OSGIN1	16:839826	96.352	66.635	-0.369	4.932	0	0	yes	96.3523	86.4921	106.212	66.6346	59.3666	73.9026
ENSG00000116455	ENSG00000116455	WDR77	1:1119812	57.273	39.652	-0.368	4.024	0	0.001	yes	57.2732	50.5572	63.9891	39.6523	34.094	45.2106
ENSG00000169583	ENSG00000169583	CLIC3	9:1398890	583.564	404.938	-0.365	8.058	0	0	yes	583.564	547.572	619.557	404.938	378.013	431.863
ENSG00000001631	ENSG00000001631	KRIT1	7:9182828	109.497	76.266	-0.362	4.31	0	0	yes	109.497	97.7524	121.242	76.2662	66.4204	86.112
ENSG00000138794	ENSG00000138794	CASP6	4:1104813	79.549	55.38	-0.362	4.155	0	0.001	yes	79.5492	70.5063	88.5922	55.3797	48.0596	62.6997
ENSG00000115274	ENSG00000115274	INO80B	2:7468214	96.473	67.223	-0.361	5.182	0	0	yes	96.4726	87.3476	105.598	67.2226	60.3368	74.1083
ENSG00000233885	ENSG00000233885	YEATS2-AS1	3:1834156	0.152	0.106	-0.36	4.906	0	0	yes	0.151812	0.137153	0.166471	0.105963	0.09428	0.117647
ENSG00000084754	ENSG00000084754	HADHA	2:2641350	68.058	47.558	-0.358	4.222	0	0.001	yes	68.0581	60.4435	75.6726	47.5584	41.4847	53.6322
ENSG00000135486	ENSG00000135486	HNRNPA1	12:546247	198.144	138.684	-0.357	5.192	0	0	yes	198.144	179.943	216.345	138.684	124.506	152.862
ENSG00000258017	ENSG00000258017	RP11-386G11.10	12:495215	176.005	123.167	-0.357	4.047	0	0.001	yes	176.005	155.765	196.245	123.167	106.689	139.646
ENSG00000150893	ENSG00000150893	FREM2	13:392612	95.834	67.107	-0.356	5.242	0	0	yes	95.8341	87.1759	104.492	67.1074	60.2898	73.9249
ENSG00000218014	ENSG00000218014	RP11-263F15.1	6:7229442	179.419	125.761	-0.355	4.742	0	0	yes	179.419	161.22	197.617	125.761	111.887	139.635
ENSG00000238862	ENSG00000238862	SNORD19B	3:5252935	16.082	11.273	-0.355	16.095	0	0	yes	16.0816	15.5855	16.5778	11.2731	10.9171	11.629
ENSG00000106628	ENSG00000106628	POLD2	7:4415428	462.402	324.634	-0.354	4.868	0	0	yes	462.402	414.445	510.36	324.634	291.589	357.679
ENSG00000230042	ENSG00000230042	AK3P3	7:2314535	0.034	0.024	-0.352	5.381	0	0	yes	0.0340106	0.030883	0.0371382	0.0239193	0.021694	0.026145
ENSG00000089737	ENSG00000089737	DDX24	14:945172	223.289	157.211	-0.351	6.962	0	0	yes	223.289	206.039	240.54	157.211	147.032	167.389
ENSG00000251357	ENSG00000251357	AP000350.10	22:241988	82.276	57.936	-0.351	3.492	0	0.009	yes	82.2757	71.2951	93.2563	57.9357	49.2392	66.6323
ENSG00000167085	ENSG00000167085	PHB	17:474814	88.972	62.822	-0.348	3.568	0	0.007	yes	88.9724	78.7257	99.2191	62.8218	52.9305	72.7132
ENSG00000124302	ENSG00000124302	CHST8	19:341128	35.163	24.843	-0.347	4.185	0	0.001	yes	35.1629	31.2229	39.1029	24.843	21.7991	27.8869
ENSG00000147642	ENSG00000147642	SYBU	8:1105862	98.268	69.484	-0.347	3.828	0	0.003	yes	98.2675	82.787	113.748	69.4837	63.2805	75.6868
ENSG00000145016	ENSG00000145016	KIAA0226	3:1973982	70.13	50.05	-0.337	3.718	0	0.004	yes	70.1301	61.9107	78.3495	50.05	43.1157	56.9844
ENSG00000164414	ENSG00000164414	SLC35A1	6:8811770	47.037	33.61	-0.336	3.79	0	0.003	yes	47.0368	41.5658	52.5078	33.6103	29.1096	38.111
ENSG00000175224	ENSG00000175224	ATG13	11:466244	164.478	117.54	-0.336	5.793	0	0	yes	164.478	152.011	176.945	117.54	107.217	127.862
ENSG00000234751	ENSG00000234751	AP002381.2	11:725253	0.2	0.143	-0.335	6.661	0	0	yes	0.200341	0.186288	0.214393	0.143352	0.133033	0.153671
ENSG00000110047	ENSG00000110047	EHD1	11:646191	178.1	127.535	-0.334	3.562	0	0.007	yes	178.1	162.53	193.671	127.535	106.382	148.688
ENSG00000185963	ENSG00000185963	BICD2	9:9547364	15.986	11.463	-0.333	4.473	0	0	yes	15.9864	14.4177	17.555	11.463	10.1819	12.7441
ENSG00000167548	ENSG00000167548	MLL2	12:493889	90.079	64.636	-0.332	4.614	0	0	yes	90.0785	81.7078	98.4491	64.6355	57.5367	71.7344
ENSG00000119711	ENSG00000119711	ALDH6A1	14:743185	33.467	24.039	-0.331	3.542	0	0.007	yes	33.4671	29.3899	37.5444	24.0387	20.6339	27.4436
ENSG00000184047	ENSG00000184047	DIABLO	12:122688	137.147	98.797	-0.328	5.501	0	0	yes	137.147	126.025	148.27	98.7971	90.159	107.435
ENSG00000187514	ENSG00000187514	PTMA	2:2325716	759.594	547.774	-0.327	5.23	0	0	yes	759.594	698.163	821.024	547.774	495.551	599.996
ENSG00000164032	ENSG00000164032	H2AFZ	4:1008692	242.292	175.645	-0.322	4.338	0	0	yes	242.292	217.363	267.22	175.645	156.888	194.403
ENSG00000099337	ENSG00000099337	KCNK6	19:388104	55.498	40.397	-0.318	4.045	0	0.001	yes	55.4979	49.6018	61.3941	40.3968	35.7249	45.0687
ENSG00000122085	ENSG00000122085	MTERFD2	2:2419382	66.694	48.543	-0.318	3.643	0	0.005	yes	66.6937	59.0734	74.3141	48.5428	42.1457	54.94
ENSG00000135473	ENSG00000135473	PAN2	12:566654	106.969	77.828	-0.318	4.153	0	0.001	yes	106.969	96.8023	117.135	77.8277	68.4792	87.1761
ENSG00000172794	ENSG00000172794	RAB37	17:726667	107.707	78.351	-0.318	5.158	0	0	yes	107.707	97.9555	117.459	78.3508	71.7823	84.9194
ENSG00000257529	ENSG00000257529	RP1-164F3.9	X:1006458	127.803	93.033	-0.318	3.486	0	0.009	yes	127.803	112.23	143.377	93.0326	80.4305	105.635
ENSG00000105778	ENSG00000105778	AVL9	7:3253503	94.311	68.683	-0.317	4.625	0	0	yes	94.3111	85.6643	102.958	68.683	61.6796	75.6865

ENSG00000159423	ENSG00000159423	ALDH4A1	1:1916609	51.229	37.31	-0.317	4.491	0	0	yes	51.2291	46.5424	55.9158	37.3098	33.2978	41.3219
ENSG00000131051	ENSG00000131051	RBM39	20:342915	178.394	130.063	-0.316	5.003	0	0	yes	178.394	163.354	193.434	130.063	117.828	142.297
ENSG00000165792	ENSG00000165792	METTL17	14:214579	106.148	77.365	-0.316	4.176	0	0.001	yes	106.148	95.853	116.443	77.3651	68.3631	86.3671
ENSG00000157077	ENSG00000157077	ZFYVE9	1:5260804	9.508	6.937	-0.315	3.53	0	0.008	yes	9.5078	8.40115	10.6144	6.93744	5.99785	7.87704
ENSG00000116299	ENSG00000116299	KIAA1324	1:1096485	396.589	290.516	-0.311	3.953	0	0.002	yes	396.589	373.478	419.7	290.516	248.02	333.012
ENSG00000163812	ENSG00000163812	ZDHHC3	3:4495674	85.699	62.827	-0.31	3.728	0	0.004	yes	85.6994	76.1289	95.27	62.8274	55.0651	70.5897
ENSG00000105662	ENSG00000105662	CRTC1	19:187944	11.359	8.341	-0.309	3.686	0	0.004	yes	11.3588	10.0891	12.6286	8.34118	7.29998	9.38238
ENSG00000085831	ENSG00000085831	TTC39A	1:5175292	260.022	191.049	-0.308	5.972	0	0	yes	260.022	242.013	278.032	191.049	176.428	205.671
ENSG00000182899	ENSG00000182899	RPL35A	3:1976159	312.066	230.482	-0.303	4.22	0	0.001	yes	312.066	286.373	337.759	230.482	203.362	257.602
ENSG00000144118	ENSG00000144118	RALB	2:1209976	101.051	74.782	-0.301	3.695	0	0.004	yes	101.051	87.9152	114.187	74.7824	67.4354	82.1295
ENSG00000168350	ENSG00000168350	DEGS2	14:100612	283.732	210.042	-0.301	4.363	0	0	yes	283.732	257.575	309.89	210.042	188.511	231.572
ENSG00000248487	ENSG00000248487	ABHD14A	3:5198932	63.762	47.256	-0.3	3.61	0	0.006	yes	63.7615	56.7719	70.7512	47.2555	41.3653	53.1457
ENSG00000057608	ENSG00000057608	GDI2	10:580718	152.525	113.183	-0.298	4.119	0	0.001	yes	152.525	136.671	168.38	113.183	101.766	124.601
ENSG00000173402	ENSG00000173402	DAG1	3:4950614	145.924	108.416	-0.297	4.492	0	0	yes	145.924	132.914	158.933	108.416	97.8218	119.01
ENSG00000113716	ENSG00000113716	HMGXB3	5:1493402	64.296	47.846	-0.296	4.138	0	0.001	yes	64.2963	58.065	70.5276	47.8457	42.8267	52.8648
ENSG00000072274	ENSG00000072274	TFRC	3:1957540	476.146	354.854	-0.294	5.191	0	0	yes	476.146	438.299	513.993	354.854	326.218	383.49
ENSG00000137411	ENSG00000137411	VARS2	6:3087596	79.519	59.259	-0.294	4.074	0	0.001	yes	79.519	72.5484	86.4896	59.2593	52.4615	66.057
ENSG00000149485	ENSG00000149485	FADS1	11:615670	134.164	100.069	-0.293	4.202	0	0.001	yes	134.164	121.748	146.58	100.069	89.6178	110.52
ENSG00000003989	ENSG00000003989	SLC7A2	8:1735459	23.774	17.746	-0.292	3.791	0	0.003	yes	23.7742	21.3004	26.248	17.7464	15.7252	19.7677
ENSG00000205476	ENSG00000205476	CCDC85C	14:999475	118.334	88.339	-0.292	4.392	0	0	yes	118.334	107.77	128.898	88.3389	79.6172	97.0606
ENSG00000198900	ENSG00000198900	TOP1	20:396574	39.673	29.802	-0.286	3.681	0	0.004	yes	39.6734	35.4841	43.8628	29.8023	26.4031	33.2014
ENSG00000143416	ENSG00000143416	SELENBP1	1:1513367	870.164	657.046	-0.281	5.77	0	0	yes	870.164	810.504	929.825	657.046	611.62	702.473
ENSG00000168769	ENSG00000168769	TET2	4:1060670	7.486	5.652	-0.281	4.16	0	0.001	yes	7.48648	6.80449	8.16847	5.6519	5.08769	6.21611
ENSG00000135052	ENSG00000135052	GOLM1	9:8864106	221.564	167.431	-0.28	4.432	0	0	yes	221.564	203.308	239.819	167.431	151.378	183.484
ENSG00000198755	ENSG00000198755	RPL10A	6:3543618	239.493	181.033	-0.28	4.4	0	0	yes	239.493	220.28	258.706	181.033	163.162	198.903
ENSG00000257727	ENSG00000257727	CNPY2	12:566654	129.97	98.572	-0.277	3.542	0	0.007	yes	129.97	116.167	143.772	98.5716	87.2899	109.853
ENSG00000255329	ENSG00000255329	CTA-797E19.3	11:701168	0.455	0.347	-0.272	16.487	0	0	yes	0.455056	0.444715	0.465398	0.34652	0.338203	0.354838
ENSG00000136883	ENSG00000136883	KIF12	9:1168484	367.963	281.431	-0.268	4.784	0	0	yes	367.963	340.027	395.899	281.431	258.224	304.638
ENSG00000100568	ENSG00000100568	VTI1B	14:680865	99.332	76.076	-0.267	3.504	0	0.008	yes	99.3319	89.4042	109.26	76.0764	67.3379	84.8149
ENSG00000184205	ENSG00000184205	TSPYL2	X:5311154	169.161	129.819	-0.265	4.685	0	0	yes	169.161	155.999	182.323	129.819	119.18	140.457
ENSG00000054148	ENSG00000054148	PHPT1	9:1397431	169.748	130.355	-0.264	4.031	0	0.001	yes	169.748	155.468	184.027	130.355	117.265	143.445
ENSG00000133872	ENSG00000133872	TMEM66	8:2992052	355.125	272.867	-0.263	4.988	0	0	yes	355.125	329.489	380.761	272.867	251.82	293.914
ENSG00000141753	ENSG00000141753	IGFBP4	17:385997	412.306	317.468	-0.261	5.249	0	0	yes	412.306	383.815	440.797	317.468	294.696	340.24
ENSG00000186868	ENSG00000186868	MAPT	17:439717	27.533	21.22	-0.26	3.954	0	0.002	yes	27.5327	25.0202	30.0452	21.2203	19.2051	23.2356
ENSG00000141524	ENSG00000141524	TMC6	17:761089	61.173	47.241	-0.258	3.785	0	0.003	yes	61.1732	56.2255	66.1209	47.2407	42.0431	52.4382
ENSG00000231500	ENSG00000231500	RPS18	6:3321731	728.882	565.655	-0.254	3.912	0	0.002	yes	728.882	663.025	794.74	565.655	513.079	618.23
ENSG00000171302	ENSG00000171302	CANT1	17:769877	163.074	126.8	-0.252	5.179	0	0	yes	163.074	152.261	173.888	126.8	117.796	135.803
ENSG00000177685	ENSG00000177685	EFCAB4A	11:818901	141.57	110.093	-0.251	4.848	0	0	yes	141.57	131.632	151.508	110.093	101.682	118.503
ENSG00000102882	ENSG00000102882	MAPK3	16:301254	282.598	220.036	-0.25	5.604	0	0	yes	282.598	264.577	300.62	220.036	206.282	233.791
ENSG00000139624	ENSG00000139624	CERS5	12:505057	141.861	110.553	-0.249	3.842	0	0.002	yes	141.861	129.308	154.414	110.553	100.053	121.053
ENSG00000087111	ENSG00000087111	PIGS	17:268804	137.538	107.282	-0.248	3.965	0	0.002	yes	137.538	125.915	149.16	107.282	97.3522	117.212
ENSG00000113758	ENSG00000113758	DBN1	5:1768836	321.979	252.334	-0.244	4.689	0	0	yes	321.979	299.083	344.874	252.334	233.195	271.474
ENSG00000182199	ENSG00000182199	SHMT2	12:576231	246.302	193.632	-0.241	5.196	0	0	yes	246.302	230.317	262.286	193.632	180.839	206.425
ENSG00000164924	ENSG00000164924	YWHAZ	8:1019308	249.648	196.779	-0.238	4.774	0	0	yes	249.648	233.268	266.027	196.779	182.007	211.551
ENSG00000120738	ENSG00000120738	EGR1	5:1378011	186.902	147.417	-0.237	4.499	0	0	yes	186.902	173.423	200.38	147.417	136.065	158.77
ENSG00000130702	ENSG00000130702	LAMA5	20:608771	695.803	550.322	-0.235	5.476	0	0	yes	695.803	656.124	735.481	550.322	515.136	585.507
ENSG00000100412	ENSG00000100412	ACO2	22:418651	73.865	58.462	-0.234	3.694	0	0.004	yes	73.8654	67.2088	80.522	58.4618	53.2605	63.6631
ENSG00000131759	ENSG00000131759	RARA	17:384654	173.216	137.447	-0.231	4.701	0	0	yes	173.216	161.23	185.202	137.447	127.832	147.063
ENSG00000164466	ENSG00000164466	SFXN1	5:1749040	347.991	276.252	-0.231	4.279	0	0	yes	347.991	324.937	371.046	276.252	252.72	299.784
ENSG00000171345	ENSG00000171345	KRT19	17:396798	2912.98	2316.13	-0.229	6.013	0	0	yes	2912.98	2758.02	3067.95	2316.13	2189.56	2442.7

ENSG00000172809	ENSG00000172809	RPL38	17:721997	876.643	700.706	-0.224	3.886	0	0.002	yes	876.643	807.855	945.431	700.706	641.528	759.883
ENSG00000072310	ENSG00000072310	SREBF1	17:175847	1697.02	1361.28	-0.22	7.539	0	0	yes	1697.02	1628.82	1765.23	1361.28	1303.45	1419.1
ENSG00000010404	ENSG00000010404	IDS	X:1485585	124.117	100.141	-0.215	3.741	0	0.004	yes	124.117	114.47	133.764	100.141	91.6882	108.594
ENSG00000157106	ENSG00000157106	SMG1	16:188161	18.612	15.02	-0.214	3.683	0	0.004	yes	18.6118	17.0723	20.1514	15.02	13.7891	16.2508
ENSG00000123349	ENSG00000123349	PFDN5	12:536890	609.964	497.054	-0.205	3.765	0	0.003	yes	609.964	565.67	654.258	497.054	456.814	537.293
ENSG00000175416	ENSG00000175416	CLTB	5:1757924	582.195	475.78	-0.202	4.048	0	0.001	yes	582.195	542.182	622.208	475.78	441.402	510.158
ENSG00000182473	ENSG00000182473	EXOC7	17:740752	179.121	146.738	-0.199	3.982	0	0.001	yes	179.121	169.694	188.549	146.738	134.235	159.24
ENSG00000100280	ENSG00000100280	AP1B1	22:297236	180.995	149.554	-0.191	4.449	0	0	yes	180.995	170.324	191.666	149.554	140.236	158.871
ENSG00000133112	ENSG00000133112	TPT1	13:459110	1041.63	863.239	-0.188	4.009	0	0.001	yes	1041.63	976.631	1106.62	863.239	802.886	923.593
ENSG00000110492	ENSG00000110492	MDK	11:464023	470.305	390.505	-0.186	3.535	0	0.007	yes	470.305	436.593	504.016	390.505	360.435	420.575
ENSG00000112655	ENSG00000112655	PTK7	6:4304400	227.101	188.712	-0.185	3.746	0	0.004	yes	227.101	211.228	242.974	188.712	175.52	201.904
ENSG00000127837	ENSG00000127837	AAMP	2:2191288	535.893	446.557	-0.182	4.042	0	0.001	yes	535.893	501.714	570.073	446.557	418.056	475.058
ENSG00000167972	ENSG00000167972	ABCA3	16:232587	138.976	115.922	-0.181	3.749	0	0.003	yes	138.976	129.435	148.518	115.922	108.017	123.826
ENSG00000163468	ENSG00000163468	CCT3	1:1562787	724.44	606.864	-0.177	4.815	0	0	yes	724.44	688.746	760.133	606.864	573.713	640.014
ENSG00000181222	ENSG00000181222	SLC35G6	17:736268	210.169	176.63	-0.174	4.455	0	0	yes	210.169	198.781	221.557	176.63	166.709	186.551
ENSG00000107331	ENSG00000107331	ABCA2	9:1399016	719.226	605.358	-0.172	3.971	0	0.002	yes	719.226	678.76	759.692	605.358	565.338	645.377
ENSG00000183495	ENSG00000183495	EP400	12:132434	18.926	16	-0.168	3.596	0	0.006	yes	18.9262	17.6996	20.1528	16.0002	14.9241	17.0763
ENSG00000142192	ENSG00000142192	APP	21:272528	420.199	356.214	-0.165	3.617	0	0.006	yes	420.199	393.374	447.025	356.214	332.945	379.483
ENSG00000196498	ENSG00000196498	NCOR2	12:124808	663.199	563.827	-0.162	3.866	0	0.002	yes	663.199	624.335	702.063	563.827	529.906	597.747
ENSG00000124222	ENSG00000124222	STX16	20:572263	213.601	182.651	-0.157	3.517	0	0.008	yes	213.601	200.684	226.519	182.651	170.721	194.582
ENSG00000007202	ENSG00000007202	KIAA0100	17:269046	131.683	112.958	-0.153	3.75	0	0.003	yes	131.683	124.106	139.261	112.958	106.391	119.526
ENSG00000197102	ENSG00000197102	DYNC1H1	14:102430	726.352	627.452	-0.146	3.856	0	0.002	yes	726.352	687.407	765.297	627.452	593.728	661.175
ENSG00000197746	ENSG00000197746	PSAP	10:735760	420.294	366.806	-0.136	3.499	0	0.008	yes	420.294	397.424	443.164	366.806	346.404	387.208
ENSG00000143569	ENSG00000143569	UBAP2L	1:1541791	431.456	383.399	-0.118	3.545	0	0.007	yes	431.456	411.579	451.333	383.399	364.948	401.85
ENSG00000111644	ENSG00000111644	ACRBP	12:672800	0.246	0	-1.79769e+	-1.79769e+	0	0.001	yes	0.246093	0.116939	0.375246	0	0	0
ENSG00000116701	ENSG00000116701	NCF2	1:1834300	0.031	0	-1.79769e+	-1.79769e+	0	0	yes	0.0305123	0.0292698	0.0317549	0	0	0
ENSG00000116783	ENSG00000116783	AC093158.1	1:7466392	0.193	0	-1.79769e+	-1.79769e+	0	0	yes	0.193163	0.153636	0.23269	0	0	0
ENSG00000121318	ENSG00000121318	TAS2R10	12:109775	0.062	0	-1.79769e+	-1.79769e+	0	0	yes	0.0616099	0.0510048	0.0722149	0	0	0
ENSG00000122986	ENSG00000122986	HVCN1	12:111051	0.018	0	-1.79769e+	-1.79769e+	0	0	yes	0.0175458	0.0152693	0.0198222	0	0	0
ENSG00000124208	ENSG00000124208	TMEM189-UBE2V1	20:486976	1.531	0	-1.79769e+	-1.79769e+	0	0	yes	1.53106	1.05472	2.00741	0	0	0
ENSG00000127364	ENSG00000127364	TAS2R4	7:1414041	0.006	0	-1.79769e+	-1.79769e+	0	0	yes	0.00639384	0.00580648	0.0069812	0	0	0
ENSG00000134398	ENSG00000134398	ERN2	16:236901	1.253	0	-1.79769e+	-1.79769e+	0	0	yes	1.25293	0.866207	1.63965	0	0	0
ENSG00000152672	ENSG00000152672	CLEC4F	2:7103577	1.11	0	-1.79769e+	-1.79769e+	0	0	yes	1.10985	0.586846	1.63286	0	0	0
ENSG00000165409	ENSG00000165409	TSHR	14:809433	0.184	0	-1.79769e+	-1.79769e+	0	0	yes	0.183985	0.148407	0.219564	0	0	0
ENSG00000167355	ENSG00000167355	AC104389.28	11:527441	0.046	0	-1.79769e+	-1.79769e+	0	0	yes	0.0457774	0.0364332	0.0551217	0	0	0
ENSG00000170509	ENSG00000170509	HSD17B13	4:8822494	1.619	0	-1.79769e+	-1.79769e+	0	0	yes	1.61937	1.09367	2.14508	0	0	0
ENSG00000171189	ENSG00000171189	GRIK1	21:305658	0.024	0	-1.79769e+	-1.79769e+	0	0	yes	0.0235733	0.0196704	0.0274763	0	0	0
ENSG00000172209	ENSG00000172209	GPR22	7:1068094	0.029	0	-1.79769e+	-1.79769e+	0	0	yes	0.0289486	0.0275876	0.0303096	0	0	0
ENSG00000173585	ENSG00000173585	CCR9	3:4586480	0.02	0	-1.79769e+	-1.79769e+	0	0.008	yes	0.0203228	0.00803473	0.032611	0	0	0
ENSG00000175206	ENSG00000175206	NPPA	1:1182184	0.053	0	-1.79769e+	-1.79769e+	0	0	yes	0.0529668	0.0490512	0.0568823	0	0	0
ENSG00000175773	ENSG00000175773	RP11-121M22.1	11:130096	0.014	0	-1.79769e+	-1.79769e+	0	0	yes	0.0143368	0.0120769	0.0165966	0	0	0
ENSG00000177182	ENSG00000177182	CLVS1	8:6196971	0.18	0	-1.79769e+	-1.79769e+	0	0	yes	0.180134	0.162576	0.197692	0	0	0
ENSG00000178965	ENSG00000178965	C1orf173	1:7501808	0.024	0	-1.79769e+	-1.79769e+	0	0	yes	0.0235562	0.0217334	0.0253791	0	0	0
ENSG00000179256	ENSG00000179256	C12orf69	12:149565	0.008	0	-1.79769e+	-1.79769e+	0	0	yes	0.00844278	0.00573892	0.0111466	0	0	0
ENSG00000183426	ENSG00000183426	NPIP	16:147664	0.006	0	-1.79769e+	-1.79769e+	0	0	yes	0.006221	0.00382647	0.00861554	0	0	0
ENSG00000184084	ENSG00000184084	CTD-2372A4.1	5:7292198	0.327	0	-1.79769e+	-1.79769e+	0	0	yes	0.326936	0.302602	0.351271	0	0	0
ENSG00000184434	ENSG00000184434	LRRC19	9:2690408	0.002	0	-1.79769e+	-1.79769e+	0	0	yes	0.00198668	0.00181193	0.00216143	0	0	0
ENSG00000187144	ENSG00000187144	SPATA21	1:1667906	0.023	0	-1.79769e+	-1.79769e+	0	0	yes	0.0232852	0.0221493	0.0244212	0	0	0
ENSG00000187721	ENSG00000187721	RP11-301J16.2	13:195208	0.229	0	-1.79769e+	-1.79769e+	0	0	yes	0.229221	0.155811	0.30263	0	0	0
ENSG00000187747	ENSG00000187747	OR52B6	11:527441	0.053	0	-1.79769e+	-1.79769e+	0	0	yes	0.0530712	0.0422381	0.0639044	0	0	0

ENSG00000197071	ENSG00000197071	AC010243.1	5:8748544	0.15	0	-1.79769e+	-1.79769e+	0	0	yes	0.150239	0.118912	0.181567	0	0	0
ENSG00000198553	ENSG00000198553	KCNRG	13:505700	7.476	0	-1.79769e+	-1.79769e+	0	0	yes	7.47569	6.03907	8.9123	0	0	0
ENSG00000199509	ENSG00000199509	5S_rRNA	20:211066	2.085	0	-1.79769e+	-1.79769e+	0	0	yes	2.08484	1.54654	2.62315	0	0	0
ENSG00000199719	ENSG00000199719	7SK	20:182691	0.556	0	-1.79769e+	-1.79769e+	0	0	yes	0.556108	0.454151	0.658064	0	0	0
ENSG00000201199	ENSG00000201199	snoU2_19	11:933948	57.05	0	-1.79769e+	-1.79769e+	0	0	yes	57.0499	52.0938	62.0061	0	0	0
ENSG00000202415	ENSG00000202415	7SK	1:9982170	0.229	0	-1.79769e+	-1.79769e+	0	0	yes	0.22915	0.193363	0.264937	0	0	0
ENSG00000203466	ENSG00000203466	RP11-182J1.4	15:851426	0.206	0	-1.79769e+	-1.79769e+	0	0	yes	0.206228	0.15914	0.253316	0	0	0
ENSG00000204422	ENSG00000204422	LY6G6E	6:3165472	1.205	0	-1.79769e+	-1.79769e+	0	0	yes	1.20522	0.742346	1.6681	0	0	0
ENSG00000204775	ENSG00000204775	AC145291.1	8:1453170	0.589	0	-1.79769e+	-1.79769e+	0	0.008	yes	0.589305	0.233941	0.944669	0	0	0
ENSG00000205231	ENSG00000205231	RP11-465B22.6	1:1108435	0.02	0	-1.79769e+	-1.79769e+	0	0.002	yes	0.0202302	0.00941671	0.0310437	0	0	0
ENSG00000205424	ENSG00000205424	AL592528.1	21:470636	0.01	0	-1.79769e+	-1.79769e+	0	0	yes	0.0101545	0.008244	0.012065	0	0	0
ENSG00000207145	ENSG00000207145	SNORA18	11:933948	7.662	0	-1.79769e+	-1.79769e+	0	0	yes	7.66211	6.99647	8.32775	0	0	0
ENSG00000207185	ENSG00000207185	U6	11:118307	10.857	0	-1.79769e+	-1.79769e+	0	0	yes	10.8572	10.5506	11.1638	0	0	0
ENSG00000207314	ENSG00000207314	SNORA44	1:2887959	13.188	0	-1.79769e+	-1.79769e+	0	0	yes	13.1884	11.7494	14.6274	0	0	0
ENSG00000207601	ENSG00000207601	MIR611	11:614479	18.648	0	-1.79769e+	-1.79769e+	0	0	yes	18.6482	17.4476	19.8488	0	0	0
ENSG00000212464	ENSG00000212464	SNORA12	10:101989	7.493	0	-1.79769e+	-1.79769e+	0	0	yes	7.49289	6.60676	8.37901	0	0	0
ENSG00000212496	ENSG00000212496	U6	12:505695	4.651	0	-1.79769e+	-1.79769e+	0	0	yes	4.65115	4.23091	5.07138	0	0	0
ENSG00000213703	ENSG00000213703	RP5-849H19.2	1:6292039	0.126	0	-1.79769e+	-1.79769e+	0	0	yes	0.125815	0.111836	0.139795	0	0	0
ENSG00000213707	ENSG00000213707	HMGB1P10	22:269214	0.233	0	-1.79769e+	-1.79769e+	0	0	yes	0.232937	0.189494	0.27638	0	0	0
ENSG00000214535	ENSG00000214535	RPS15AP1	20:211066	0.144	0	-1.79769e+	-1.79769e+	0	0	yes	0.143998	0.106818	0.181178	0	0	0
ENSG00000214593	ENSG00000214593	RP11-65N13.6	9:1280240	1.053	0	-1.79769e+	-1.79769e+	0	0	yes	1.05301	0.989077	1.11695	0	0	0
ENSG00000217624	ENSG00000217624	RP11-204C16.4	X:4137418	0.048	0	-1.79769e+	-1.79769e+	0	0	yes	0.048493	0.0437148	0.0532711	0	0	0
ENSG00000219790	ENSG00000219790	RP11-129K24.3	6:5681977	0.242	0	-1.79769e+	-1.79769e+	0	0	yes	0.241654	0.169607	0.313701	0	0	0
ENSG00000220891	ENSG00000220891	LL22NC03-63E9.3	22:228901	0.006	0	-1.79769e+	-1.79769e+	0	0	yes	0.00574986	0.00492423	0.00657549	0	0	0
ENSG00000221025	ENSG00000221025	MIR1250	17:790089	17.27	0	-1.79769e+	-1.79769e+	0	0	yes	17.2698	16.3487	18.191	0	0	0
ENSG00000221170	ENSG00000221170	MIR1304	11:933948	36.037	0	-1.79769e+	-1.79769e+	0	0	yes	36.037	32.9063	39.1677	0	0	0
ENSG00000221585	ENSG00000221585	MIR1226	3:4784439	61.999	0	-1.79769e+	-1.79769e+	0	0	yes	61.9988	59.3165	64.6811	0	0	0
ENSG00000221656	ENSG00000221656	MIR1225	16:208981	36.98	0	-1.79769e+	-1.79769e+	0	0	yes	36.9801	35.9619	37.9982	0	0	0
ENSG00000222383	ENSG00000222383	5S_rRNA	6:1074797	4.517	0	-1.79769e+	-1.79769e+	0	0	yes	4.51654	3.05118	5.9819	0	0	0
ENSG00000222482	ENSG00000222482	AC005071.1	7:9977518	34.744	0	-1.79769e+	-1.79769e+	0	0	yes	34.7435	32.3795	37.1075	0	0	0
ENSG00000222750	ENSG00000222750	U4	16:194218	4.629	0	-1.79769e+	-1.79769e+	0	0	yes	4.62928	3.83243	5.42613	0	0	0
ENSG00000223430	ENSG00000223430	AC011243.1	2:1334293	0.227	0	-1.79769e+	-1.79769e+	0	0	yes	0.226719	0.193823	0.259615	0	0	0
ENSG00000223522	ENSG00000223522	AC093690.1	2:2799458	0.039	0	-1.79769e+	-1.79769e+	0	0	yes	0.0390225	0.0362971	0.041748	0	0	0
ENSG00000223703	ENSG00000223703	AC027612.4	2:9192475	0.022	0	-1.79769e+	-1.79769e+	0	0	yes	0.0224049	0.0173312	0.0274786	0	0	0
ENSG00000223787	ENSG00000223787	RP4-593M8.1	1:9297425	0.195	0	-1.79769e+	-1.79769e+	0	0	yes	0.194666	0.155135	0.234196	0	0	0
ENSG00000223878	ENSG00000223878	AC005517.3	17:153393	0.113	0	-1.79769e+	-1.79769e+	0	0	yes	0.112684	0.0893143	0.136053	0	0	0
ENSG00000223998	ENSG00000223998	RP11-321N4.3	6:8621521	0.075	0	-1.79769e+	-1.79769e+	0	0	yes	0.0749178	0.0711578	0.0786779	0	0	0
ENSG00000224055	ENSG00000224055	GAPDHP55	15:441629	0.034	0	-1.79769e+	-1.79769e+	0	0	yes	0.0342241	0.0295559	0.0388922	0	0	0
ENSG00000224623	ENSG00000224623	RP11-247I13.8	22:317955	0.171	0	-1.79769e+	-1.79769e+	0	0	yes	0.170588	0.165104	0.176073	0	0	0
ENSG00000225133	ENSG00000225133	MORF4L1P4	13:417070	0.077	0	-1.79769e+	-1.79769e+	0	0	yes	0.0765724	0.0692963	0.0838485	0	0	0
ENSG00000225187	ENSG00000225187	AC073283.7	2:4712641	0.025	0	-1.79769e+	-1.79769e+	0	0	yes	0.0254039	0.0241777	0.02663	0	0	0
ENSG00000225205	ENSG00000225205	AC093818.1	2:1732920	0.039	0	-1.79769e+	-1.79769e+	0	0	yes	0.0393961	0.0324045	0.0463878	0	0	0
ENSG00000225839	ENSG00000225839	TRMT2B-AS1	X:1002643	0.26	0	-1.79769e+	-1.79769e+	0	0	yes	0.260069	0.23785	0.282288	0	0	0
ENSG00000226054	ENSG00000226054	MEMO1P1	21:375026	0.118	0	-1.79769e+	-1.79769e+	0	0	yes	0.117779	0.112214	0.123343	0	0	0
ENSG00000226059	ENSG00000226059	HMGB3P20	7:2670668	0.141	0	-1.79769e+	-1.79769e+	0	0	yes	0.140636	0.126412	0.154861	0	0	0
ENSG00000226143	ENSG00000226143	RP1-138B7.5	20:421363	0.079	0	-1.79769e+	-1.79769e+	0	0	yes	0.0790259	0.0557224	0.102329	0	0	0
ENSG00000226784	ENSG00000226784	PGAM4	X:7716619	0.023	0	-1.79769e+	-1.79769e+	0	0	yes	0.0234246	0.0192343	0.0276149	0	0	0
ENSG00000226953	ENSG00000226953	AC010890.1	2:1334293	0.177	0	-1.79769e+	-1.79769e+	0	0	yes	0.177085	0.151386	0.202785	0	0	0
ENSG00000226963	ENSG00000226963	AC078883.4	2:1732920	0.017	0	-1.79769e+	-1.79769e+	0	0	yes	0.0166753	0.0137159	0.0196347	0	0	0
ENSG00000227200	ENSG00000227200	RP11-121A14.3	9:1270198	0.004	0	-1.79769e+	-1.79769e+	0	0	yes	0.00357693	0.00315093	0.00400294	0	0	0

ENSG00000227528	ENSG00000227528	DIAPH3-AS1	13:602397	0	0	-1.79769e+	-1.79769e+	0	0	yes	0.000453266	0.00037282	0.000533707	0	0	0
ENSG00000227589	ENSG00000227589	RP5-1092A11.5	1:3547330	0.068	0	-1.79769e+	-1.79769e+	0	0	yes	0.0679361	0.063327	0.0725453	0	0	0
ENSG00000228021	ENSG00000228021	RP11-383C5.3	10:127371	0.22	0	-1.79769e+	-1.79769e+	0	0	yes	0.219771	0.193735	0.245807	0	0	0
ENSG00000228595	ENSG00000228595	PAICSP2	9:9432537	0.035	0	-1.79769e+	-1.79769e+	0	0	yes	0.0349044	0.0281871	0.0416218	0	0	0
ENSG00000228838	ENSG00000228838	RP4-784A16.2	1:5371121	2.658	0	-1.79769e+	-1.79769e+	0	0.003	yes	2.65804	1.19932	4.11675	0	0	0
ENSG00000229534	ENSG00000229534	AC015691.7	11:527441	0.116	0	-1.79769e+	-1.79769e+	0	0	yes	0.115895	0.0922379	0.139552	0	0	0
ENSG00000229863	ENSG00000229863	RP11-157F20.1	3:5712400	0.207	0	-1.79769e+	-1.79769e+	0	0	yes	0.206743	0.12996	0.283525	0	0	0
ENSG00000230116	ENSG00000230116	RP11-163G10.2	1:4289599	0.053	0	-1.79769e+	-1.79769e+	0	0	yes	0.0527514	0.0466703	0.0588325	0	0	0
ENSG00000230194	ENSG00000230194	AC000081.2	22:191669	0.066	0	-1.79769e+	-1.79769e+	0	0	yes	0.0655779	0.052717	0.0784388	0	0	0
ENSG00000230340	ENSG00000230340	AC019257.1	8:1772141	0.062	0	-1.79769e+	-1.79769e+	0	0	yes	0.0618594	0.0545692	0.0691495	0	0	0
ENSG00000230698	ENSG00000230698	CTD-2330K9.2	3:4992443	0.115	0	-1.79769e+	-1.79769e+	0	0	yes	0.11532	0.101682	0.128958	0	0	0
ENSG00000230940	ENSG00000230940	RP11-32B11.2	9:1347354	0.397	0	-1.79769e+	-1.79769e+	0	0	yes	0.397251	0.287073	0.507429	0	0	0
ENSG00000230953	ENSG00000230953	RP4-631H13.6	1:5336165	0.733	0	-1.79769e+	-1.79769e+	0	0	yes	0.732656	0.659844	0.805467	0	0	0
ENSG00000231521	ENSG00000231521	RP11-244N9.4	9:1002634	1.06	0	-1.79769e+	-1.79769e+	0	0	yes	1.06029	0.999802	1.12078	0	0	0
ENSG00000231542	ENSG00000231542	TAB3-AS1	X:3084555	1.636	0	-1.79769e+	-1.79769e+	0	0	yes	1.63554	1.46217	1.8089	0	0	0
ENSG00000231792	ENSG00000231792	RP5-1198E17.1	1:1738312	0.12	0	-1.79769e+	-1.79769e+	0	0	yes	0.120466	0.101478	0.139454	0	0	0
ENSG00000231882	ENSG00000231882	F10-AS1	13:113777	0.171	0	-1.79769e+	-1.79769e+	0	0.001	yes	0.171405	0.0828921	0.259919	0	0	0
ENSG00000231887	ENSG00000231887	PRH1	12:109775	0.11	0	-1.79769e+	-1.79769e+	0	0	yes	0.10979	0.0908915	0.128688	0	0	0
ENSG00000231942	ENSG00000231942	RP13-923O23.5	8:8270405	0.033	0	-1.79769e+	-1.79769e+	0	0	yes	0.0325898	0.0217266	0.0434531	0	0	0
ENSG00000231951	ENSG00000231951	AC004837.4	7:3960597	3.38	0	-1.79769e+	-1.79769e+	0	0.003	yes	3.38026	1.50249	5.25803	0	0	0
ENSG00000232371	ENSG00000232371	ZNF878	19:120984	1.609	0	-1.79769e+	-1.79769e+	0	0	yes	1.60868	0.978736	2.23863	0	0	0
ENSG00000232739	ENSG00000232739	RP11-25G10.2	10:152536	0.018	0	-1.79769e+	-1.79769e+	0	0	yes	0.0178324	0.0131494	0.0225154	0	0	0
ENSG00000232830	ENSG00000232830	RP11-144C15.1	3:1010430	0.258	0	-1.79769e+	-1.79769e+	0	0	yes	0.257623	0.195532	0.319713	0	0	0
ENSG00000232912	ENSG00000232912	RP5-1115A15.1	1:8412456	0.067	0	-1.79769e+	-1.79769e+	0	0	yes	0.0670335	0.0641746	0.0698925	0	0	0
ENSG00000233033	ENSG00000233033	CASK-AS1	X:4137418	0.163	0	-1.79769e+	-1.79769e+	0	0	yes	0.162899	0.146848	0.178949	0	0	0
ENSG00000233246	ENSG00000233246	RP11-415J8.5	1:3378029	0.261	0	-1.79769e+	-1.79769e+	0	0	yes	0.261326	0.240601	0.28205	0	0	0
ENSG00000233579	ENSG00000233579	KRT8P15	2:2036406	0.068	0	-1.79769e+	-1.79769e+	0	0	yes	0.0684329	0.057368	0.0794979	0	0	0
ENSG00000233603	ENSG00000233603	JTBP1	22:411656	0.187	0	-1.79769e+	-1.79769e+	0	0	yes	0.186725	0.161076	0.212374	0	0	0
ENSG00000233793	ENSG00000233793	RP11-501H19.4	X:2773419	7.912	0	-1.79769e+	-1.79769e+	0	0.001	yes	7.9119	3.76568	12.0581	0	0	0
ENSG00000234076	ENSG00000234076	TPRG1-AS1	3:1886595	0.074	0	-1.79769e+	-1.79769e+	0	0	yes	0.0739971	0.0516861	0.0963081	0	0	0
ENSG00000234271	ENSG00000234271	RP1-138B7.4	20:421363	0.578	0	-1.79769e+	-1.79769e+	0	0	yes	0.578018	0.40757	0.748467	0	0	0
ENSG00000234324	ENSG00000234324	AC005517.2	17:153393	0.073	0	-1.79769e+	-1.79769e+	0	0	yes	0.0733416	0.0581313	0.0885519	0	0	0
ENSG00000234336	ENSG00000234336	AC005017.1	7:2777301	0.049	0	-1.79769e+	-1.79769e+	0	0	yes	0.0494904	0.0464743	0.0525065	0	0	0
ENSG00000234483	ENSG00000234483	AC010680.1	2:1793875	0.005	0	-1.79769e+	-1.79769e+	0	0	yes	0.00496976	0.00412057	0.00581895	0	0	0
ENSG00000234650	ENSG00000234650	PCCA-AS1	13:100741	0.294	0	-1.79769e+	-1.79769e+	0	0	yes	0.294356	0.238144	0.350569	0	0	0
ENSG00000235105	ENSG00000235105	RP11-329A14.1	1:4876104	0.016	0	-1.79769e+	-1.79769e+	0	0	yes	0.0155009	0.0116556	0.0193463	0	0	0
ENSG00000235652	ENSG00000235652	RP11-545I5.3	6:1461363	0.173	0	-1.79769e+	-1.79769e+	0	0	yes	0.172644	0.146316	0.198971	0	0	0
ENSG00000235763	ENSG00000235763	SNRPGP5	10:129386	0.529	0	-1.79769e+	-1.79769e+	0	0	yes	0.529468	0.477424	0.581512	0	0	0
ENSG00000236132	ENSG00000236132	CTA-440B3.1	22:321499	0.134	0	-1.79769e+	-1.79769e+	0	0	yes	0.133632	0.119585	0.147679	0	0	0
ENSG00000236256	ENSG00000236256	DIAPH2-AS1	X:9593966	0.114	0	-1.79769e+	-1.79769e+	0	0	yes	0.113508	0.0978028	0.129212	0	0	0
ENSG00000236439	ENSG00000236439	RP11-175B9.3	1:2023178	0.214	0	-1.79769e+	-1.79769e+	0	0	yes	0.213925	0.194753	0.233097	0	0	0
ENSG00000236472	ENSG00000236472	AC002401.1	17:482111	4.559	0	-1.79769e+	-1.79769e+	0	0.003	yes	4.5586	2.0112	7.10599	0	0	0
ENSG00000236474	ENSG00000236474	GCNT1P1	20:183640	0.02	0	-1.79769e+	-1.79769e+	0	0	yes	0.0201791	0.0172039	0.0231544	0	0	0
ENSG00000236546	ENSG00000236546	RP1-118J21.5	1:4036109	0.034	0	-1.79769e+	-1.79769e+	0	0	yes	0.0343344	0.0234769	0.0451918	0	0	0
ENSG00000237380	ENSG00000237380	AC009336.21	2:1769863	0.006	0	-1.79769e+	-1.79769e+	0	0	yes	0.00578445	0.00455802	0.00701089	0	0	0
ENSG00000237416	ENSG00000237416	RP11-465K1.2	1:9528589	0.149	0	-1.79769e+	-1.79769e+	0	0	yes	0.149225	0.114521	0.183929	0	0	0
ENSG00000237806	ENSG00000237806	RP11-262M14.1	3:1807014	0.339	0	-1.79769e+	-1.79769e+	0	0	yes	0.339071	0.242194	0.435948	0	0	0
ENSG00000237813	ENSG00000237813	AC002066.1	7:1158505	0.097	0	-1.79769e+	-1.79769e+	0	0	yes	0.0972139	0.0880546	0.106373	0	0	0
ENSG00000237916	ENSG00000237916	RP11-537E18.1	2:1103003	0.322	0	-1.79769e+	-1.79769e+	0	0	yes	0.321677	0.245325	0.39803	0	0	0
ENSG00000237996	ENSG00000237996	RP11-258A12.3	10:114710	0.015	0	-1.79769e+	-1.79769e+	0	0	yes	0.0151051	0.0135574	0.0166528	0	0	0

ENSG00000238111	ENSG00000238111	AC066692.3	2:2397153	0.015	0	-1.79769e+	-1.79769e+	0	0	yes	0.0154858	0.0138489	0.0171227	0	0	0
ENSG00000238298	ENSG00000238298	snoU13	9:1333200	35.545	0	-1.79769e+	-1.79769e+	0	0	yes	35.5452	23.3533	47.7371	0	0	0
ENSG00000238567	ENSG00000238567	snoU13	2:1718473	11.858	0	-1.79769e+	-1.79769e+	0	0	yes	11.8577	10.7786	12.9368	0	0	0
ENSG00000238868	ENSG00000238868	snoU13	7:1399934	1.959	0	-1.79769e+	-1.79769e+	0	0	yes	1.9586	1.75658	2.16061	0	0	0
ENSG00000238924	ENSG00000238924	U7	10:103433	186.086	0	-1.79769e+	-1.79769e+	0	0	yes	186.086	177.37	194.803	0	0	0
ENSG00000239344	ENSG00000239344	RP11-771F20.1	8:1044956	0.131	0	-1.79769e+	-1.79769e+	0	0	yes	0.130609	0.118521	0.142696	0	0	0
ENSG00000239783	ENSG00000239783	RP5-1050K3.3	20:455232	0.093	0	-1.79769e+	-1.79769e+	0	0	yes	0.0933234	0.0845052	0.102142	0	0	0
ENSG00000240695	ENSG00000240695	RP11-102M11.1	3:1359691	0.159	0	-1.79769e+	-1.79769e+	0	0	yes	0.158773	0.152074	0.165472	0	0	0
ENSG00000240766	ENSG00000240766	PLCXD2-AS1	3:1113935	0.007	0	-1.79769e+	-1.79769e+	0	0	yes	0.00740083	0.00534821	0.00945346	0	0	0
ENSG00000241420	ENSG00000241420	AC074338.1	7:3253503	0.171	0	-1.79769e+	-1.79769e+	0	0	yes	0.170921	0.165279	0.176562	0	0	0
ENSG00000241550	ENSG00000241550	AC104212.1	8:8083095	0.206	0	-1.79769e+	-1.79769e+	0	0	yes	0.205663	0.192524	0.218803	0	0	0
ENSG00000241651	ENSG00000241651	RP11-214O14.1	4:1289824	0.057	0	-1.79769e+	-1.79769e+	0	0	yes	0.0573459	0.0475112	0.0671807	0	0	0
ENSG00000241853	ENSG00000241853	RP11-147K6.1	4:8901141	0.433	0	-1.79769e+	-1.79769e+	0	0	yes	0.432756	0.37303	0.492482	0	0	0
ENSG00000241895	ENSG00000241895	AL157827.2	9:9505963	48.322	0	-1.79769e+	-1.79769e+	0	0	yes	48.3224	44.7055	51.9392	0	0	0
ENSG00000242094	ENSG00000242094	FOXP1-IT1	3:7100384	0.018	0	-1.79769e+	-1.79769e+	0	0	yes	0.01834	0.0168104	0.0198697	0	0	0
ENSG00000242180	ENSG00000242180	OR51B5	11:527441	0.029	0	-1.79769e+	-1.79769e+	0	0	yes	0.0294162	0.0234117	0.0354208	0	0	0
ENSG00000242219	ENSG00000242219	AC011442.1	19:110715	0.544	0	-1.79769e+	-1.79769e+	0	0	yes	0.543618	0.53001	0.557225	0	0	0
ENSG00000242719	ENSG00000242719	AC009634.1	8:4270477	1.199	0	-1.79769e+	-1.79769e+	0	0	yes	1.19883	1.03755	1.36011	0	0	0
ENSG00000242761	ENSG00000242761	RP11-115P21.1	18:320732	0.04	0	-1.79769e+	-1.79769e+	0	0	yes	0.0403414	0.0367687	0.0439141	0	0	0
ENSG00000243243	ENSG00000243243	AC073130.3	7:1158505	0.084	0	-1.79769e+	-1.79769e+	0	0	yes	0.0843188	0.0763761	0.0922614	0	0	0
ENSG00000243341	ENSG00000243341	RP11-436A20.3	3:1807014	0.128	0	-1.79769e+	-1.79769e+	0	0	yes	0.12763	0.0911645	0.164096	0	0	0
ENSG00000243806	ENSG00000243806	CTC-484P3.1	5:9348867	0.153	0	-1.79769e+	-1.79769e+	0	0	yes	0.153232	0.130131	0.176332	0	0	0
ENSG00000244052	ENSG00000244052	RPL5P24	8:1038385	0.119	0	-1.79769e+	-1.79769e+	0	0	yes	0.11915	0.113554	0.124747	0	0	0
ENSG00000244307	ENSG00000244307	AF235103.1	8:1459976	0.015	0	-1.79769e+	-1.79769e+	0	0	yes	0.0146098	0.0113223	0.0178973	0	0	0
ENSG00000244604	ENSG00000244604	RP11-713H12.1	17:833917	0.17	0	-1.79769e+	-1.79769e+	0	0	yes	0.170453	0.162083	0.178823	0	0	0
ENSG00000245043	ENSG00000245043	AC073210.1	7:6449873	0.066	0	-1.79769e+	-1.79769e+	0	0.005	yes	0.0663407	0.0280389	0.104642	0	0	0
ENSG00000245148	ENSG00000245148	ARAP1-AS2	11:723961	0.056	0	-1.79769e+	-1.79769e+	0	0	yes	0.0556607	0.0546498	0.0566716	0	0	0
ENSG00000245521	ENSG00000245521	AL139082.1	13:526110	0.012	0	-1.79769e+	-1.79769e+	0	0	yes	0.0116539	0.00944161	0.0138662	0	0	0
ENSG00000247872	ENSG00000247872	CTD-2198K18.1	5:710470-	0.112	0	-1.79769e+	-1.79769e+	0	0	yes	0.112229	0.103425	0.121033	0	0	0
ENSG00000248245	ENSG00000248245	CTB-49A3.2	5:1324961	0.033	0	-1.79769e+	-1.79769e+	0	0	yes	0.0326669	0.0214619	0.0438719	0	0	0
ENSG00000248394	ENSG00000248394	CTD-2034L19.1	4:1289824	0.061	0	-1.79769e+	-1.79769e+	0	0	yes	0.0614806	0.0509368	0.0720245	0	0	0
ENSG00000248470	ENSG00000248470	AL356752.1	13:113344	0.005	0	-1.79769e+	-1.79769e+	0	0	yes	0.00492642	0.00460523	0.00524762	0	0	0
ENSG00000248476	ENSG00000248476	BACH1-IT1	21:305658	0.048	0	-1.79769e+	-1.79769e+	0	0	yes	0.0481149	0.0401509	0.056079	0	0	0
ENSG00000248492	ENSG00000248492	AC015599.1	8:1354900	0	0	-1.79769e+	-1.79769e+	0	0	yes	0.000260489	0.00022907	0.000291905	0	0	0
ENSG00000248636	ENSG00000248636	RP11-768F21.1	12:119772	0.214	0	-1.79769e+	-1.79769e+	0	0	yes	0.214343	0.197617	0.231068	0	0	0
ENSG00000248820	ENSG00000248820	RP11-15B17.3	4:1008692	0.529	0	-1.79769e+	-1.79769e+	0	0	yes	0.529344	0.498326	0.560361	0	0	0
ENSG00000249098	ENSG00000249098	RP11-39E3.4	3:1305694	0.183	0	-1.79769e+	-1.79769e+	0	0	yes	0.183378	0.173344	0.193412	0	0	0
ENSG00000249310	ENSG00000249310	CTA-150C2.16	22:393783	0.186	0	-1.79769e+	-1.79769e+	0	0	yes	0.1856	0.132022	0.239178	0	0	0
ENSG00000249633	ENSG00000249633	OR52V1P	11:527441	0.115	0	-1.79769e+	-1.79769e+	0	0	yes	0.114996	0.0915227	0.13847	0	0	0
ENSG00000249715	ENSG00000249715	AC068539.1	2:9725890	0.011	0	-1.79769e+	-1.79769e+	0	0	yes	0.0114105	0.0107228	0.0120983	0	0	0
ENSG00000250068	ENSG00000250068	RP11-576C12.1	9:1151422	0.438	0	-1.79769e+	-1.79769e+	0	0	yes	0.437991	0.396789	0.479194	0	0	0
ENSG00000250174	ENSG00000250174	MYLK-AS2	3:1233043	0.058	0	-1.79769e+	-1.79769e+	0	0	yes	0.0576117	0.0439346	0.0712888	0	0	0
ENSG00000250487	ENSG00000250487	RP11-43D2.6	5:1239726	0.236	0	-1.79769e+	-1.79769e+	0	0	yes	0.23647	0.161692	0.311248	0	0	0
ENSG00000250623	ENSG00000250623	RP11-317B7.3	4:2073644	0.037	0	-1.79769e+	-1.79769e+	0	0	yes	0.0367498	0.0319855	0.041514	0	0	0
ENSG00000250697	ENSG00000250697	CTD-2066L21.3	5:3292574	11.434	0	-1.79769e+	-1.79769e+	0	0	yes	11.4335	6.0355	16.8315	0	0	0
ENSG00000250780	ENSG00000250780	AL356423.1	13:991024	0.027	0	-1.79769e+	-1.79769e+	0	0	yes	0.0271327	0.0248658	0.0293997	0	0	0
ENSG00000250963	ENSG00000250963	RP11-423H2.2	5:1772364	0.003	0	-1.79769e+	-1.79769e+	0	0	yes	0.00322967	0.00249829	0.00396104	0	0	0
ENSG00000251018	ENSG00000251018	RP11-80G7.1	5:1628871	0.059	0	-1.79769e+	-1.79769e+	0	0	yes	0.0594993	0.0525469	0.0664516	0	0	0
ENSG00000251095	ENSG00000251095	RP11-115D19.1	4:9047250	0.061	0	-1.79769e+	-1.79769e+	0	0	yes	0.0608597	0.0366994	0.08502	0	0	0
ENSG00000251131	ENSG00000251131	CTD-2035E11.3	5:4301451	0.536	0	-1.79769e+	-1.79769e+	0	0	yes	0.536257	0.484619	0.587896	0	0	0

ENSG00000251154	ENSG00000251154	AC006153.3	7:8996453	0.339	0	-1.79769e+	-1.79769e+	0	0	yes	0.339307	0.315463	0.363151	0	0	0
ENSG00000252494	ENSG00000252494	U6	11:778119	19.626	0	-1.79769e+	-1.79769e+	0	0	yes	19.6263	17.7486	21.504	0	0	0
ENSG00000252974	ENSG00000252974	AC121334.1	12:396870	92.337	0	-1.79769e+	-1.79769e+	0	0	yes	92.3373	84.3828	100.292	0	0	0
ENSG00000253172	ENSG00000253172	CTC-209H22.3	5:1725711	0.136	0	-1.79769e+	-1.79769e+	0	0	yes	0.136343	0.120805	0.151881	0	0	0
ENSG00000253174	ENSG00000253174	RP11-360L9.7	8:4138672	0.241	0	-1.79769e+	-1.79769e+	0	0	yes	0.240838	0.225959	0.255717	0	0	0
ENSG00000253302	ENSG00000253302	RP11-181D18.2	8:7433223	0.003	0	-1.79769e+	-1.79769e+	0	0	yes	0.0026705	0.00232717	0.00301383	0	0	0
ENSG00000253406	ENSG00000253406	AC012613.2	5:1485210	0.009	0	-1.79769e+	-1.79769e+	0	0	yes	0.00904555	0.00818113	0.00990998	0	0	0
ENSG00000253518	ENSG00000253518	CTD-2031P19.4	5:5523092	0.072	0	-1.79769e+	-1.79769e+	0	0	yes	0.0716044	0.0628237	0.0803851	0	0	0
ENSG00000253528	ENSG00000253528	RP11-347C18.4	8:9582553	0.418	0	-1.79769e+	-1.79769e+	0	0	yes	0.417953	0.400992	0.434914	0	0	0
ENSG00000254460	ENSG00000254460	RP11-939C17.4	11:751456	0.065	0	-1.79769e+	-1.79769e+	0	0	yes	0.0648234	0.0501438	0.079503	0	0	0
ENSG00000254633	ENSG00000254633	RP11-23J9.7	9:1000000	0.039	0	-1.79769e+	-1.79769e+	0	0	yes	0.0389954	0.031157	0.0468338	0	0	0
ENSG00000254788	ENSG00000254788	CKLF-CMTM1	16:665864	24.754	0	-1.79769e+	-1.79769e+	0	0	yes	24.7541	18.968	30.5402	0	0	0
ENSG00000255010	ENSG00000255010	RP11-113K21.3	11:827831	0.398	0	-1.79769e+	-1.79769e+	0	0	yes	0.397803	0.281752	0.513855	0	0	0
ENSG00000255158	ENSG00000255158	RP11-754B17.1	11:644232	0.053	0	-1.79769e+	-1.79769e+	0	0	yes	0.0531152	0.0511948	0.0550356	0	0	0
ENSG00000255276	ENSG00000255276	RP11-23F23.1	11:411593	0.638	0	-1.79769e+	-1.79769e+	0	0	yes	0.638439	0.602637	0.674241	0	0	0
ENSG00000255478	ENSG00000255478	RP11-867O8.5	11:651349	0.126	0	-1.79769e+	-1.79769e+	0	0	yes	0.126292	0.104791	0.147794	0	0	0
ENSG00000255581	ENSG00000255581	RP11-69M1.4	12:791781	0.158	0	-1.79769e+	-1.79769e+	0	0	yes	0.15786	0.145919	0.169802	0	0	0
ENSG00000255723	ENSG00000255723	RP11-529H2.2	4:8822494	0.198	0	-1.79769e+	-1.79769e+	0	0	yes	0.197576	0.133474	0.261679	0	0	0
ENSG00000255958	ENSG00000255958	RP11-656E20.5	12:103650	0.024	0	-1.79769e+	-1.79769e+	0	0	yes	0.0243083	0.0152804	0.0333362	0	0	0
ENSG00000255990	ENSG00000255990	AC034102.1	12:563676	0.643	0	-1.79769e+	-1.79769e+	0	0.001	yes	0.643244	0.316183	0.970305	0	0	0
ENSG00000256278	ENSG00000256278	RP11-182J1.5	15:851426	0.091	0	-1.79769e+	-1.79769e+	0	0	yes	0.0911565	0.07038	0.111933	0	0	0
ENSG00000256329	ENSG00000256329	AC006064.2	12:660114	0.898	0	-1.79769e+	-1.79769e+	0	0	yes	0.898272	0.855916	0.940629	0	0	0
ENSG00000256650	ENSG00000256650	RERG-IT1	12:152607	0.488	0	-1.79769e+	-1.79769e+	0	0	yes	0.487898	0.4253	0.550495	0	0	0
ENSG00000256757	ENSG00000256757	RP11-159N11.3	11:113276	0.196	0	-1.79769e+	-1.79769e+	0	0	yes	0.195803	0.124306	0.2673	0	0	0
ENSG00000256813	ENSG00000256813	RP11-804A23.4	11:606034	4.485	0	-1.79769e+	-1.79769e+	0	0.002	yes	4.48531	2.06111	6.90951	0	0	0
ENSG00000256898	ENSG00000256898	RP11-707G14.8	11:735837	0.068	0	-1.79769e+	-1.79769e+	0	0	yes	0.0675429	0.0635029	0.071583	0	0	0
ENSG00000257077	ENSG00000257077	RP11-467L20.6	11:614479	2.961	0	-1.79769e+	-1.79769e+	0	0	yes	2.96145	1.55389	4.369	0	0	0
ENSG00000257241	ENSG00000257241	RP11-611E13.3	12:701324	0.264	0	-1.79769e+	-1.79769e+	0	0	yes	0.263757	0.251526	0.275989	0	0	0
ENSG00000257355	ENSG00000257355	CTD-2006C1.10	19:120984	0.007	0	-1.79769e+	-1.79769e+	0	0	yes	0.00708522	0.00515687	0.00901357	0	0	0
ENSG00000257446	ENSG00000257446	AC022415.4	19:120984	1.611	0	-1.79769e+	-1.79769e+	0	0	yes	1.61128	0.98021	2.24235	0	0	0
ENSG00000257613	ENSG00000257613	RP11-320P7.1	12:701324	0.044	0	-1.79769e+	-1.79769e+	0	0	yes	0.0439745	0.0419352	0.0460137	0	0	0
ENSG00000258011	ENSG00000258011	HMGA1P3	12:110810	0.137	0	-1.79769e+	-1.79769e+	0	0	yes	0.136759	0.129734	0.143784	0	0	0
ENSG00000258571	ENSG00000258571	PTTG4P	14:713741	0.563	0	-1.79769e+	-1.79769e+	0	0	yes	0.562929	0.528952	0.596906	0	0	0
ENSG00000258653	ENSG00000258653	RP5-1021I20.4	14:743185	0.391	0	-1.79769e+	-1.79769e+	0	0.002	yes	0.391244	0.185322	0.597166	0	0	0
ENSG00000258830	ENSG00000258830	RP11-123K3.4	12:576372	0.563	0	-1.79769e+	-1.79769e+	0	0.007	yes	0.563133	0.228568	0.897697	0	0	0
ENSG00000258966	ENSG00000258966	RP5-1163L11.3	14:713741	0.119	0	-1.79769e+	-1.79769e+	0	0	yes	0.119113	0.111924	0.126303	0	0	0
ENSG00000259014	ENSG00000259014	RP4-647C14.5	14:737418	0.024	0	-1.79769e+	-1.79769e+	0	0	yes	0.0238332	0.0224115	0.025255	0	0	0
ENSG00000259118	ENSG00000259118	RP11-840I19.3	14:653810	0.052	0	-1.79769e+	-1.79769e+	0	0	yes	0.0520726	0.0505465	0.0535987	0	0	0
ENSG00000128645	ENSG00000128645	HOXD1	2:1769863	0.091	0.007	-2.546	3.309	0.001	0.015	yes	0.091424	0	0.230928	0.00716936	0.005743	0.008596
ENSG00000214274	ENSG00000214274	ANG	14:211522	27.75	2.566	-2.381	3.215	0.001	0.02	yes	27.7502	0	67.0814	2.56627	1.46159	3.67095
ENSG00000250234	ENSG00000250234	CTD-2024P10.1	5:3465634	0.961	0.094	-2.33	3.34	0.001	0.014	yes	0.961335	0	2.29373	0.0935134	0.078293	0.108733
ENSG00000249823	ENSG00000249823	RP11-311D14.2	4:1064737	1.113	0.158	-1.954	3.347	0.001	0.014	yes	1.11323	0	2.41174	0.157813	0.150634	0.164992
ENSG00000251467	ENSG00000251467	CTC-250P20.2	5:7225180	0.621	0.093	-1.894	3.473	0.001	0.009	yes	0.621435	0	1.29348	0.0934609	0.080075	0.106847
ENSG00000256690	ENSG00000256690	RP11-727F15.9	11:625997	6.394	1.077	-1.781	3.177	0.001	0.023	yes	6.39442	3.79148	8.99736	1.0769	0	2.20216
ENSG00000253502	ENSG00000253502	ATP6V1G1P2	8:4800119	3.563	0.601	-1.779	3.279	0.001	0.017	yes	3.56273	0.511492	6.61398	0.601327	0.200442	1.00221
ENSG00000253773	ENSG00000253773	KB-1047C11.2	8:9621668	9.601	1.682	-1.742	3.39	0.001	0.012	yes	9.60091	6.01322	13.1886	1.68177	0.071662	3.29188
ENSG00000250900	ENSG00000250900	CTC-338M12.6	5:1806189	17.277	3.072	-1.727	3.287	0.001	0.016	yes	17.2769	13.1595	21.3942	3.07197	0	6.21613
ENSG00000236404	ENSG00000236404	RP11-125B21.2	9:2422701	8.686	1.666	-1.651	3.332	0.001	0.014	yes	8.68645	6.36271	11.0102	1.66634	0.076157	3.25652
ENSG00000257851	ENSG00000257851	RP11-199A1.1	12:519840	0.455	0.087	-1.651	3.389	0.001	0.012	yes	0.454986	0.0259608	0.884012	0.0873091	0.065976	0.108642
ENSG00000092529	ENSG00000092529	CAPN3	15:425664	2.491	0.489	-1.629	3.446	0.001	0.01	yes	2.49108	1.13069	3.85146	0.488681	0.111638	0.865724

ENSG00000244078	ENSG00000244078	RP11-431I8.1	3:1599434	2.063	0.442	-1.541	3.384	0.001	0.012	yes	2.06284	0.187111	3.93856	0.441675	0.418674	0.464675
ENSG00000188001	ENSG00000188001	TPRG1	3:1886595	5.825	1.263	-1.529	3.414	0.001	0.011	yes	5.82502	3.39949	8.25055	1.26296	0.261598	2.26433
ENSG00000258559	ENSG00000258559	AC005519.4	14:747521	2.333	0.584	-1.385	3.186	0.001	0.022	yes	2.33307	1.46189	3.20426	0.583922	0.125382	1.04246
ENSG00000232630	ENSG00000232630	PRPS1P2	9:1279088	3.976	1.048	-1.333	3.248	0.001	0.018	yes	3.97586	2.54468	5.40703	1.04846	0.275232	1.82169
ENSG00000182993	ENSG00000182993	C12orf60	12:149565	6.368	1.775	-1.277	3.405	0.001	0.011	yes	6.36782	4.20394	8.5317	1.77511	0.587462	2.96275
ENSG00000122012	ENSG00000122012	SV2C	5:7537777	0.053	0.015	-1.237	3.278	0.001	0.017	yes	0.0525188	0.0172024	0.0878353	0.015242	0.010013	0.020471
ENSG00000109189	ENSG00000109189	USP46	4:5346033	14.124	5.046	-1.029	3.276	0.001	0.017	yes	14.124	8.10048	20.1475	5.04638	2.71755	7.37521
ENSG00000129535	ENSG00000129535	NRL	14:245493	4.92	1.793	-1.009	3.445	0.001	0.01	yes	4.9201	3.40095	6.43926	1.79326	0.900193	2.68633
ENSG00000148737	ENSG00000148737	TCF7L2	10:114710	22.169	8.328	-0.979	3.308	0.001	0.015	yes	22.1688	9.86692	34.4707	8.32827	6.61466	10.0419
ENSG00000159128	ENSG00000159128	IFNGR2	21:347572	28.974	10.894	-0.978	3.352	0.001	0.013	yes	28.9744	21.768	36.1808	10.8942	5.14167	16.6468
ENSG00000076258	ENSG00000076258	FMO4	1:1712833	6.554	2.703	-0.886	3.301	0.001	0.016	yes	6.55354	4.20691	8.90017	2.70261	1.62198	3.78323
ENSG00000171169	ENSG00000171169	NAIF1	9:1308235	22.408	9.44	-0.864	3.31	0.001	0.015	yes	22.4077	16.0485	28.7668	9.43959	5.29978	13.5794
ENSG00000235546	ENSG00000235546	AC087499.5	17:205159	5.31	2.337	-0.821	3.477	0.001	0.009	yes	5.30998	3.9731	6.64686	2.337	1.4039	3.2701
ENSG00000157107	ENSG00000157107	FCHO2	5:7225180	13.297	6.118	-0.776	3.288	0.001	0.016	yes	13.2972	9.23697	17.3575	6.11844	3.91506	8.32182
ENSG00000248168	ENSG00000248168	AC138028.2	16:887382	1.428	0.672	-0.754	3.304	0.001	0.015	yes	1.42833	1.07157	1.78508	0.672017	0.415334	0.928701
ENSG00000110013	ENSG00000110013	SIAE	11:124492	10.892	5.166	-0.746	3.437	0.001	0.01	yes	10.8921	8.07581	13.7084	5.16632	3.3656	6.96704
ENSG00000138380	ENSG00000138380	ALS2CR8	2:2037453	8.816	4.217	-0.737	3.297	0.001	0.016	yes	8.81626	6.37593	11.2566	4.2174	2.73565	5.69916
ENSG00000228834	ENSG00000228834	RP11-249L21.4	6:1091696	95.225	45.902	-0.73	3.298	0.001	0.016	yes	95.2248	71.8291	118.62	45.9018	29.0081	62.7955
ENSG00000110446	ENSG00000110446	SLC15A3	11:606806	18.19	8.933	-0.711	3.313	0.001	0.015	yes	18.1902	14.319	22.0614	8.93291	5.60256	12.2632
ENSG00000167165	ENSG00000167165	UGT1A6	2:2345262	0.218	0.109	-0.688	3.229	0.001	0.019	yes	0.21775	0.128466	0.307035	0.109403	0.096651	0.122155
ENSG00000166526	ENSG00000166526	ZNF3	7:9964738	77.199	38.858	-0.686	3.244	0.001	0.019	yes	77.1985	46.0071	108.39	38.8577	33.97	43.7454
ENSG00000198400	ENSG00000198400	NTRK1	1:1567760	0.136	0.069	-0.673	3.393	0.001	0.012	yes	0.136062	0.102974	0.169151	0.0693823	0.047616	0.091149
ENSG00000161813	ENSG00000161813	LARP4	12:507200	24.66	12.7	-0.664	3.3	0.001	0.016	yes	24.6603	15.8869	33.4336	12.6998	10.3172	15.0824
ENSG00000197429	ENSG00000197429	IPP	1:4609297	9.735	5.104	-0.646	3.409	0.001	0.011	yes	9.73531	6.91623	12.5544	5.10367	3.85708	6.35027
ENSG00000229638	ENSG00000229638	RP11-110C15.4	3:1850007	7.532	3.948	-0.646	3.338	0.001	0.014	yes	7.53171	5.8198	9.24361	3.94782	2.71119	5.18446
ENSG00000230870	ENSG00000230870	FBXW11P1	21:329994	7.219	3.954	-0.602	3.324	0.001	0.015	yes	7.21886	5.63991	8.7978	3.95408	2.81264	5.09553
ENSG00000186106	ENSG00000186106	ANKRD46	8:1015219	27.175	15.048	-0.591	3.175	0.001	0.023	yes	27.1745	21.238	33.111	15.0482	10.512	19.5845
ENSG00000204390	ENSG00000204390	HSPA1L	6:3177739	4.498	2.492	-0.591	3.324	0.001	0.015	yes	4.49841	3.57425	5.42257	2.49182	1.76908	3.21457
ENSG00000127838	ENSG00000127838	PNKD	2:2191351	84.416	46.81	-0.59	3.414	0.001	0.011	yes	84.4161	60.6006	108.232	46.8095	37.4763	56.1427
ENSG00000100522	ENSG00000100522	GNPNAT1	14:532419	72.817	40.733	-0.581	3.428	0.001	0.011	yes	72.8174	55.764	89.8708	40.7331	30.7517	50.7146
ENSG00000115541	ENSG00000115541	HSPE1	2:1983513	29.949	16.91	-0.572	3.271	0.001	0.017	yes	29.9486	24.1767	35.7206	16.9098	11.9794	21.8402
ENSG00000149313	ENSG00000149313	AASDHPPT	11:105921	18.782	10.661	-0.566	3.313	0.001	0.015	yes	18.7815	14.1048	23.4581	10.6608	8.16299	13.1585
ENSG00000255432	ENSG00000255432	RP11-831H9.11	11:624143	29.843	16.941	-0.566	3.443	0.001	0.01	yes	29.8434	23.7144	35.9723	16.9414	12.5899	21.2928
ENSG00000168496	ENSG00000168496	FEN1	11:614479	11.882	6.772	-0.562	3.367	0.001	0.013	yes	11.8824	9.41652	14.3483	6.77245	5.00081	8.54408
ENSG00000176261	ENSG00000176261	ZBTB8OS	1:3293065	31.113	17.802	-0.558	3.186	0.001	0.022	yes	31.1133	24.6677	37.5589	17.8018	12.7689	22.8347
ENSG00000114126	ENSG00000114126	TFDP2	3:1416632	46.817	27.637	-0.527	3.263	0.001	0.018	yes	46.817	39.0862	54.5479	27.6365	19.9613	35.3117
ENSG00000197620	ENSG00000197620	CXorf40A	X:1485585	25.311	15.167	-0.512	3.47	0.001	0.009	yes	25.3114	21.6916	28.9312	15.1667	11.2505	19.0829
ENSG00000138433	ENSG00000138433	CIR1	2:1752127	14.595	8.982	-0.485	3.411	0.001	0.011	yes	14.5948	11.5051	17.6845	8.98205	7.27284	10.6913
ENSG00000154277	ENSG00000154277	UCHL1	4:4122209	36.749	22.773	-0.479	3.329	0.001	0.014	yes	36.7488	27.7165	45.781	22.7732	19.3779	26.1686
ENSG00000174775	ENSG00000174775	HRAS	11:532241	598.726	372.76	-0.474	3.298	0.001	0.016	yes	598.726	462.738	734.714	372.76	307.146	438.374
ENSG00000174579	ENSG00000174579	MSL2	3:1358677	57.614	36.478	-0.457	3.377	0.001	0.012	yes	57.6144	50.1682	65.0606	36.4777	27.8016	45.1539
ENSG00000112365	ENSG00000112365	ZBTB24	6:1097652	4.057	2.58	-0.453	3.48	0.001	0.009	yes	4.05742	3.37722	4.73763	2.58032	2.06719	3.09345
ENSG00000233830	ENSG00000233830	AC073150.4	7:2749778	49.577	32.623	-0.418	3.347	0.001	0.014	yes	49.5765	41.5284	57.6245	32.6232	26.4186	38.8278
ENSG00000153993	ENSG00000153993	SEMA3D	7:8462486	16.501	10.883	-0.416	3.385	0.001	0.012	yes	16.501	13.8584	19.1435	10.8826	8.85174	12.9136
ENSG00000131381	ENSG00000131381	ZFYVE20	3:1511157	55.78	37.645	-0.393	3.395	0.001	0.012	yes	55.7796	50.4286	61.1306	37.6449	29.7069	45.5828
ENSG00000204859	ENSG00000204859	ZBTB48	1:6640060	18.697	12.714	-0.386	3.286	0.001	0.016	yes	18.6973	15.6714	21.7233	12.714	10.5526	14.8754
ENSG00000050426	ENSG00000050426	LETMD1	12:514417	96.095	65.539	-0.383	3.433	0.001	0.01	yes	96.0948	78.5555	113.634	65.5386	57.1485	73.9288
ENSG00000139116	ENSG00000139116	KIF21A	12:396870	11.035	7.667	-0.364	3.415	0.001	0.011	yes	11.0348	9.12121	12.9484	7.66728	6.71631	8.61825
ENSG00000249921	ENSG00000249921	CTC-329H14.1	5:1080835	57.057	39.947	-0.357	3.39	0.001	0.012	yes	57.0572	49.1265	64.988	39.9465	33.64	46.253
ENSG00000126226	ENSG00000126226	PCID2	13:113831	50.145	35.519	-0.345	3.42	0.001	0.011	yes	50.1448	44.137	56.1526	35.5187	29.7569	41.2805

ENSG00000162889	ENSG00000162889	MAPKAPK2	1:2068582	318.94	225.853	-0.345	3.214	0.001	0.02	yes	318.94	272.49	365.391	225.853	190.204	261.501
ENSG00000169967	ENSG00000169967	MAP3K2	2:1280563	40.604	28.771	-0.344	3.214	0.001	0.02	yes	40.6035	35.6812	45.5259	28.7713	23.6856	33.8569
ENSG00000204310	ENSG00000204310	AGPAT1	6:3211613	185.382	131.84	-0.341	3.44	0.001	0.01	yes	185.382	161.235	209.529	131.84	112.15	151.53
ENSG00000134905	ENSG00000134905	CARS2	13:111267	76.45	54.709	-0.335	3.311	0.001	0.015	yes	76.4498	67.169	85.7306	54.709	45.8675	63.5505
ENSG00000139289	ENSG00000139289	PHLDA1	12:764192	22.86	16.358	-0.335	3.193	0.001	0.022	yes	22.8596	19.6603	26.059	16.3582	13.806	18.9104
ENSG00000114279	ENSG00000114279	FGF12	3:1918596	30.063	21.631	-0.329	3.254	0.001	0.018	yes	30.063	25.8372	34.2889	21.6305	18.4833	24.7778
ENSG00000196126	ENSG00000196126	HLA-DRB1	6:3254654	61.389	44.382	-0.324	3.459	0.001	0.01	yes	61.3886	53.6992	69.078	44.3824	38.1878	50.5769
ENSG00000154889	ENSG00000154889	MPPE1	18:116891	16.73	12.189	-0.317	3.383	0.001	0.012	yes	16.7302	14.611	18.8494	12.1888	10.5086	13.8689
ENSG00000214014	ENSG00000214014	OVCA2	17:193343	102.289	74.567	-0.316	3.391	0.001	0.012	yes	102.289	89.5341	115.044	74.5667	64.2337	84.8997
ENSG00000121310	ENSG00000121310	ECHDC2	1:5336165	84.041	61.404	-0.314	3.281	0.001	0.017	yes	84.0409	73.3808	94.7009	61.4039	52.6124	70.1954
ENSG00000101654	ENSG00000101654	RNMT	18:137266	29.795	21.952	-0.306	3.32	0.001	0.015	yes	29.7953	25.9648	33.6258	21.9518	19.0614	24.8423
ENSG00000175029	ENSG00000175029	CTBP2	10:126605	55.443	40.811	-0.306	3.25	0.001	0.018	yes	55.4425	48.4635	62.4215	40.8105	35.0807	46.5402
ENSG00000215472	ENSG00000215472	RPL17	18:470148	69.481	51.467	-0.3	3.185	0.001	0.022	yes	69.4812	60.8911	78.0714	51.4671	44.1456	58.7886
ENSG00000197375	ENSG00000197375	SLC22A5	5:1315205	56.779	42.585	-0.288	3.187	0.001	0.022	yes	56.7789	50.1771	63.3807	42.5845	36.7021	48.4669
ENSG00000070366	ENSG00000070366	SMG6	17:196313	28.694	21.7	-0.279	3.392	0.001	0.012	yes	28.6939	26.2543	31.1334	21.6995	18.637	24.7619
ENSG00000143093	ENSG00000143093	FAM40A	1:1105741	43.937	33.309	-0.277	3.345	0.001	0.014	yes	43.937	38.9944	48.8796	33.3088	29.2608	37.3568
ENSG00000182481	ENSG00000182481	KPNA2	17:658216	60.936	46.561	-0.269	3.294	0.001	0.016	yes	60.9363	54.1701	67.7024	46.5613	40.9822	52.1403
ENSG00000148296	ENSG00000148296	SURF6	9:1361975	45.657	34.918	-0.268	3.238	0.001	0.019	yes	45.657	40.5973	50.7166	34.9182	30.6197	39.2168
ENSG00000186501	ENSG00000186501	TMEM222	1:2764865	63.6	49.031	-0.26	3.192	0.001	0.022	yes	63.6	56.1856	71.0145	49.0308	43.4435	54.6182
ENSG00000121067	ENSG00000121067	SPOP	17:476762	50.178	38.734	-0.259	3.305	0.001	0.015	yes	50.1783	45.2001	55.1566	38.7337	34.0388	43.4287
ENSG00000167552	ENSG00000167552	TUBA1A	12:495785	86.847	67.407	-0.253	3.259	0.001	0.018	yes	86.8471	78.4812	95.213	67.407	59.1778	75.6362
ENSG00000109079	ENSG00000109079	TNFAIP1	17:266627	38.237	29.841	-0.248	3.389	0.001	0.012	yes	38.2369	34.4644	42.0094	29.8406	26.616	33.0653
ENSG00000083845	ENSG00000083845	RPS5	19:588986	368.098	287.667	-0.247	3.396	0.001	0.012	yes	368.098	331.445	404.75	287.667	257.272	318.062
ENSG00000141219	ENSG00000141219	C17orf80	17:711887	23.94	18.74	-0.245	3.359	0.001	0.013	yes	23.9403	21.613	26.2676	18.7396	16.7028	20.7763
ENSG00000060339	ENSG00000060339	CCAR1	10:704807	145.787	114.883	-0.238	3.434	0.001	0.01	yes	145.787	132.878	158.697	114.883	102.611	127.156
ENSG00000126351	ENSG00000126351	THRA	17:382184	23.995	18.981	-0.234	3.236	0.001	0.019	yes	23.9947	21.6636	26.3258	18.9806	16.9401	21.021
ENSG00000198742	ENSG00000198742	SMURF1	7:9847555	42.51	33.909	-0.226	3.337	0.001	0.014	yes	42.5099	38.6013	46.4185	33.9088	30.5345	37.2831
ENSG00000109971	ENSG00000109971	HSPA8	11:122928	299.105	239.503	-0.222	3.252	0.001	0.018	yes	299.105	271.763	326.448	239.503	215.172	263.833
ENSG00000244462	ENSG00000244462	RBM12	20:342139	124.03	99.782	-0.218	3.437	0.001	0.01	yes	124.03	113.535	134.524	99.7823	90.3894	109.175
ENSG00000130222	ENSG00000130222	GADD45G	9:9221992	162.374	131.064	-0.214	3.237	0.001	0.019	yes	162.374	147.404	177.345	131.064	118.62	143.509
ENSG00000167741	ENSG00000167741	GGT6	17:446022	75.844	61.714	-0.206	3.473	0.001	0.009	yes	75.8441	69.5107	82.1774	61.7135	56.5045	66.9226
ENSG00000110075	ENSG00000110075	PPP6R3	11:682281	104.924	86.586	-0.192	3.186	0.001	0.022	yes	104.924	96.5619	113.286	86.5859	78.7489	94.4228
ENSG00000198925	ENSG00000198925	ATG9A	2:2200744	111.276	91.821	-0.192	3.202	0.001	0.021	yes	111.276	102.297	120.255	91.821	83.6599	99.9821
ENSG00000171604	ENSG00000171604	CXXC5	5:1390268	340.757	282.446	-0.188	3.189	0.001	0.022	yes	340.757	314.832	366.683	282.446	257.078	307.814
ENSG00000090905	ENSG00000090905	TNRC6A	16:247410	33.32	27.801	-0.181	3.224	0.001	0.02	yes	33.3195	30.9454	35.6936	27.8009	25.3872	30.2146
ENSG00000140836	ENSG00000140836	ZFHX3	16:728167	21.025	17.565	-0.18	3.323	0.001	0.015	yes	21.0248	19.4474	22.6023	17.5645	16.1945	18.9345
ENSG00000141380	ENSG00000141380	SS18	18:235962	87.538	73.596	-0.173	3.234	0.001	0.019	yes	87.5376	80.897	94.1783	73.5957	68.0119	79.1795
ENSG00000196923	ENSG00000196923	PDLIM7	5:1769103	259.595	218.581	-0.172	3.38	0.001	0.012	yes	259.595	241.327	277.862	218.581	202.514	234.649
ENSG00000023191	ENSG00000023191	RNH1	11:494511	188.036	159.457	-0.165	3.471	0.001	0.009	yes	188.036	175.98	200.093	159.457	148.278	170.636
ENSG00000170027	ENSG00000170027	YWHAG	7:7595611	368.16	314.519	-0.157	3.283	0.001	0.016	yes	368.16	343.827	392.494	314.519	292.656	336.382
ENSG00000100605	ENSG00000100605	ITPK1	14:934032	440.529	379.721	-0.149	3.29	0.001	0.016	yes	440.529	414.715	466.342	379.721	353.635	405.806
ENSG00000110092	ENSG00000110092	CCND1	11:694558	815.645	703.08	-0.149	3.295	0.001	0.016	yes	815.645	765.155	866.136	703.08	657.02	749.141
ENSG00000196557	ENSG00000196557	CACNA1H	16:120324	120.065	103.732	-0.146	3.179	0.001	0.023	yes	120.065	112.365	127.764	103.732	96.8913	110.573
ENSG00000177731	ENSG00000177731	FLII	17:181289	706.317	613.124	-0.141	3.376	0.001	0.012	yes	706.317	665.963	746.671	613.124	575.519	650.729
ENSG00000111907	ENSG00000111907	TPD52L1	6:1254401	813.46	710.92	-0.135	3.205	0.001	0.021	yes	813.46	766.22	860.699	710.92	667.697	754.143
ENSG00000039523	ENSG00000039523	FAM65A	16:675627	101.32	88.783	-0.132	3.211	0.001	0.021	yes	101.32	95.6602	106.98	88.783	83.4213	94.1447
ENSG00000105694	ENSG00000105694	AC104537.1	19:251121	5.392	0	-1.79769e+	-1.79769e+	0.001	0.019	yes	5.3921	1.83617	8.94803	0	0	0
ENSG00000120729	ENSG00000120729	MYOT	5:1371500	0.225	0	-1.79769e+	-1.79769e+	0.001	0.017	yes	0.225116	0.0783512	0.371881	0	0	0
ENSG00000159337	ENSG00000159337	PLA2G4D	15:423598	0.252	0	-1.79769e+	-1.79769e+	0.001	0.021	yes	0.251914	0.0839715	0.419857	0	0	0
ENSG00000172543	ENSG00000172543	CTSW	11:656472	0.333	0	-1.79769e+	-1.79769e+	0.001	0.014	yes	0.332506	0.121107	0.543905	0	0	0

ENSG00000207110	ENSG00000207110	U1	8:1288067	56.649	0	-1.79769e+	-1.79769e+	0.001	0.021	yes	56.6486	18.8005	94.4967	0	0	0
ENSG00000229780	ENSG00000229780	RP11-61L14.2	1:1545210	4.362	0	-1.79769e+	-1.79769e+	0.001	0.01	yes	4.36242	1.68251	7.04232	0	0	0
ENSG00000236507	ENSG00000236507	RP5-1050E16.1	9:9250114	1.199	0	-1.79769e+	-1.79769e+	0.001	0.019	yes	1.19927	0.408388	1.99015	0	0	0
ENSG00000254480	ENSG00000254480	RP11-23F23.2	11:411593	3.638	0	-1.79769e+	-1.79769e+	0.001	0.013	yes	3.6377	1.336	5.9394	0	0	0
ENSG00000257069	ENSG00000257069	RP11-783K16.10	11:640587	0.354	0	-1.79769e+	-1.79769e+	0.001	0.017	yes	0.353898	0.123799	0.583997	0	0	0
ENSG00000258034	ENSG00000258034	RP11-493P1.2	12:116395	3.347	0	-1.79769e+	-1.79769e+	0.001	0.01	yes	3.34692	1.28815	5.4057	0	0	0
ENSG00000258588	ENSG00000258588	TRIM6-TRIM34	11:527441	0.297	0	-1.79769e+	-1.79769e+	0.001	0.013	yes	0.296842	0.109102	0.484582	0	0	0
ENSG00000258953	ENSG00000258953	C5orf63	5:1263782	6.447	0	-1.79769e+	-1.79769e+	0.001	0.013	yes	6.44709	2.36969	10.5245	0	0	0
ENSG00000255471	ENSG00000255471	RP11-736K20.5	11:865021	4.267	0.246	-2.853	3.143	0.002	0.025	yes	4.26718	0	9.49861	0.246034	0	0.575525
ENSG00000139211	ENSG00000139211	AMIGO2	12:474694	0.946	0.113	-2.129	3.167	0.002	0.023	yes	0.94606	0.535906	1.35622	0.11257	0	0.255795
ENSG00000243725	ENSG00000243725	TTC4	1:5510745	6.637	1.156	-1.747	3.169	0.002	0.023	yes	6.63697	3.33564	9.93831	1.15649	0.018221	2.29476
ENSG00000170289	ENSG00000170289	CNGB3	8:8734550	1.805	0.498	-1.288	3.139	0.002	0.025	yes	1.80543	0.441404	3.16945	0.497754	0.338055	0.657454
ENSG00000236810	ENSG00000236810	RP5-886K2.3	1:2406964	3.916	1.107	-1.263	3.117	0.002	0.027	yes	3.91601	2.06877	5.76325	1.10719	0.377528	1.83686
ENSG00000163521	ENSG00000163521	GLB1L	2:2200944	6.3	2.472	-0.936	3.093	0.002	0.029	yes	6.30039	4.20175	8.39902	2.47177	1.22316	3.72037
ENSG00000244041	ENSG00000244041	RP1-90J20.7	6:2988200	5.527	2.172	-0.934	3.066	0.002	0.031	yes	5.52666	3.78372	7.26961	2.17194	1.03965	3.30424
ENSG00000246889	ENSG00000246889	AP000487.5	11:701168	12.542	5.205	-0.879	3.123	0.002	0.027	yes	12.5424	8.65673	16.428	5.20525	2.75747	7.65304
ENSG00000154134	ENSG00000154134	ROBO3	11:124735	21.424	8.973	-0.87	3.143	0.002	0.025	yes	21.4235	16.7976	26.0495	8.97344	4.39691	13.55
ENSG00000147650	ENSG00000147650	LRP12	8:1055014	10.715	4.527	-0.862	3.036	0.002	0.034	yes	10.7147	5.68758	15.7419	4.52675	3.08093	5.97257
ENSG00000214300	ENSG00000214300	SPDYE3	7:9990532	4.302	1.891	-0.822	3.146	0.002	0.025	yes	4.30208	3.18408	5.42009	1.8913	1.03402	2.74858
ENSG00000169964	ENSG00000169964	TMEM42	3:4480320	5.388	2.385	-0.815	3.125	0.002	0.027	yes	5.38774	3.8999	6.87558	2.38463	1.32905	3.44022
ENSG00000172345	ENSG00000172345	STARD5	15:814750	4.094	1.947	-0.743	3.113	0.002	0.027	yes	4.09377	2.92073	5.2668	1.94734	1.20402	2.69067
ENSG00000213398	ENSG00000213398	LCAT	16:679736	3.421	1.644	-0.733	3.147	0.002	0.025	yes	3.42127	2.54444	4.2981	1.64376	1.00433	2.2832
ENSG00000155984	ENSG00000155984	TMEM185A	X:1486782	56.22	28.166	-0.691	3.105	0.002	0.028	yes	56.2199	40.2284	72.2114	28.1663	18.5219	37.8108
ENSG00000255529	ENSG00000255529	GRINL1A	15:578841	3.07	1.58	-0.665	3.07	0.002	0.031	yes	3.0701	2.05789	4.08232	1.57955	1.13646	2.02265
ENSG00000189343	ENSG00000189343	AC004448.3	17:193424	9.429	4.959	-0.643	3.041	0.002	0.034	yes	9.42878	7.17343	11.6841	4.95907	3.2315	6.68664
ENSG00000240429	ENSG00000240429	LRRFIP1P1	3:1788659	3.628	1.922	-0.635	3.16	0.002	0.024	yes	3.62805	2.77779	4.4783	1.9224	1.29455	2.55025
ENSG00000076321	ENSG00000076321	KLHL20	1:1736840	27.103	14.523	-0.624	3.115	0.002	0.027	yes	27.1029	19.8536	34.3522	14.5229	10.1918	18.854
ENSG00000065970	ENSG00000065970	FOXJ2	12:791781	7.866	4.292	-0.606	3.141	0.002	0.025	yes	7.86552	6.01701	9.71403	4.29246	2.97976	5.60516
ENSG00000053747	ENSG00000053747	LAMA3	18:212695	3.971	2.253	-0.567	3.149	0.002	0.025	yes	3.9714	2.81869	5.1241	2.2532	1.77363	2.73277
ENSG00000186815	ENSG00000186815	TPCN1	12:113633	46.566	26.515	-0.563	3.083	0.002	0.03	yes	46.5661	35.1237	58.0085	26.5149	19.3454	33.6845
ENSG00000151422	ENSG00000151422	FER	5:1080835	10.849	6.464	-0.518	3.046	0.002	0.033	yes	10.8493	8.19463	13.504	6.46366	4.93744	7.98988
ENSG00000145819	ENSG00000145819	ARHGAP26	5:1421499	71.907	43.779	-0.496	3.106	0.002	0.028	yes	71.9067	55.6032	88.2102	43.7785	33.9222	53.6347
ENSG00000254531	ENSG00000254531	AP001816.1	4:1019445	33.566	20.553	-0.491	3.159	0.002	0.024	yes	33.5662	26.9375	40.1949	20.553	15.6269	25.4791
ENSG00000231770	ENSG00000231770	AC046143.5	3:1943047	14.179	8.782	-0.479	3.119	0.002	0.027	yes	14.1785	11.3874	16.9695	8.78199	6.71103	10.853
ENSG00000187796	ENSG00000187796	CARD9	9:1392219	7.02	4.371	-0.474	3.064	0.002	0.032	yes	7.01956	5.85563	8.18349	4.371	3.23004	5.51195
ENSG00000164051	ENSG00000164051	CCDC51	3:4847357	19.23	12.476	-0.433	3.084	0.002	0.03	yes	19.2296	15.6565	22.8026	12.4759	9.85353	15.0982
ENSG00000006062	ENSG00000006062	MAP3K14	17:433404	6.057	3.983	-0.419	3.1	0.002	0.028	yes	6.0567	5.00461	7.1088	3.98288	3.15721	4.80855
ENSG00000108797	ENSG00000108797	CNTNAP1	17:408346	3.335	2.195	-0.418	3.034	0.002	0.034	yes	3.33514	2.73947	3.93081	2.19504	1.73382	2.65626
ENSG00000135749	ENSG00000135749	PCNXL2	1:2331198	77.642	51.711	-0.406	3.06	0.002	0.032	yes	77.6418	66.7374	88.5462	51.7111	40.049	63.3733
ENSG00000100099	ENSG00000100099	HPS4	22:268252	40.475	27.361	-0.392	3.143	0.002	0.025	yes	40.4748	32.7725	48.1771	27.361	22.9619	31.7601
ENSG00000151725	ENSG00000151725	MLF1IP	4:1855707	22.82	15.499	-0.387	3.05	0.002	0.033	yes	22.8203	19.2061	26.4345	15.4988	12.4278	18.5698
ENSG00000162746	ENSG00000162746	FCRLB	1:1616913	23.986	16.38	-0.381	3.107	0.002	0.028	yes	23.9859	21.0747	26.897	16.3799	12.8849	19.875
ENSG00000144504	ENSG00000144504	ANKMY1	2:2414188	43.653	30.379	-0.363	3.048	0.002	0.033	yes	43.6528	35.8966	51.4089	30.3789	25.5737	35.1842
ENSG00000222019	ENSG00000222019	AC133919.6	16:900712	37.061	26.11	-0.35	3.118	0.002	0.027	yes	37.0614	31.7818	42.3409	26.1101	21.5734	30.6468
ENSG00000254901	ENSG00000254901	MEF2BNB	19:192563	30.636	21.808	-0.34	3.122	0.002	0.027	yes	30.6358	26.0925	35.1791	21.8081	18.3303	25.2859
ENSG00000033011	ENSG00000033011	ALG1	16:512181	17.957	12.897	-0.331	3.118	0.002	0.027	yes	17.9571	15.486	20.4283	12.8972	10.8119	14.9825
ENSG00000134982	ENSG00000134982	APC	5:1120431	30.565	22.1	-0.324	3.172	0.002	0.023	yes	30.5653	26.1537	34.9769	22.1004	18.9003	25.3005
ENSG00000227051	ENSG00000227051	C14orf132	14:965056	176.781	127.846	-0.324	3.039	0.002	0.034	yes	176.781	160.837	192.725	127.846	103.138	152.554
ENSG00000078487	ENSG00000078487	ZCWPW1	7:9999844	30.938	22.398	-0.323	3.132	0.002	0.026	yes	30.9382	26.8499	35.0265	22.3976	18.8491	25.946
ENSG00000115419	ENSG00000115419	GLS	2:1917455	51.704	37.993	-0.308	3.046	0.002	0.033	yes	51.7041	45.4213	57.9869	37.9932	31.8482	44.1383

ENSG00000143256	ENSG00000143256	PFDN2	1:1610703	110.888	81.729	-0.305	3.114	0.002	0.027	yes	110.888	96.369	125.408	81.7293	69.815	93.6435
ENSG00000234750	ENSG00000234750	CTD-2666L21.2	19:123580	0.061	0.045	-0.295	3.112	0.002	0.027	yes	0.0608837	0.0532283	0.0685392	0.0453351	0.038908	0.051763
ENSG00000197136	ENSG00000197136	PCNXL3	11:653832	277.044	206.976	-0.292	3.143	0.002	0.025	yes	277.044	242.843	311.246	206.976	178.317	235.636
ENSG00000127663	ENSG00000127663	KDM4B	19:496912	18.487	14.088	-0.272	3.048	0.002	0.033	yes	18.4865	16.2949	20.678	14.0879	12.2116	15.9642
ENSG00000103051	ENSG00000103051	COG4	16:705144	218.001	166.205	-0.271	3.132	0.002	0.026	yes	218.001	203.222	232.781	166.205	139.711	192.699
ENSG00000147804	ENSG00000147804	SLC39A4	8:1456351	180.295	141.699	-0.241	3.12	0.002	0.027	yes	180.295	160.121	200.469	141.699	126.617	156.78
ENSG00000179409	ENSG00000179409	GEMIN4	17:647660	43.456	34.45	-0.232	3.118	0.002	0.027	yes	43.4555	39.0428	47.8682	34.4498	30.6955	38.204
ENSG00000119682	ENSG00000119682	KIAA0317	14:751198	160.903	128.233	-0.227	3.091	0.002	0.029	yes	160.903	144.999	176.807	128.233	114.304	142.161
ENSG00000225733	ENSG00000225733	AC090937.2	3:1486046	46.55	37.555	-0.215	3.171	0.002	0.023	yes	46.5495	42.2521	50.847	37.5545	33.8337	41.2753
ENSG00000071054	ENSG00000071054	MAP4K4	2:1023133	51.527	42.105	-0.202	3.038	0.002	0.034	yes	51.5271	46.541	56.5132	42.1051	38.2656	45.9445
ENSG00000113282	ENSG00000113282	CLINT1	5:1572127	170.538	139.937	-0.198	3.045	0.002	0.033	yes	170.538	154.724	186.353	139.937	127.209	152.665
ENSG00000061938	ENSG00000061938	TNK2	3:1955902	286.695	235.558	-0.196	3.106	0.002	0.028	yes	286.695	261.399	311.99	235.558	214.2	256.915
ENSG00000161618	ENSG00000161618	ALDH16A1	19:499564	37.587	30.905	-0.196	3.121	0.002	0.027	yes	37.5865	34.388	40.785	30.9051	28.058	33.7522
ENSG00000204427	ENSG00000204427	ABHD16A	6:3165472	131.745	109.313	-0.187	3.054	0.002	0.032	yes	131.745	119.909	143.581	109.313	100.249	118.376
ENSG00000132510	ENSG00000132510	KDM6B	17:774322	35.059	29.327	-0.179	3.068	0.002	0.031	yes	35.0592	32.2608	37.8576	29.327	26.8429	31.8112
ENSG00000123358	ENSG00000123358	NR4A1	12:524166	196.592	165.142	-0.174	3.143	0.002	0.025	yes	196.592	182.66	210.524	165.142	151.047	179.238
ENSG00000164855	ENSG00000164855	TMEM184A	7:1583449	162.917	139.282	-0.157	3.114	0.002	0.027	yes	162.917	151.691	174.143	139.282	129.063	149.502
ENSG00000155506	ENSG00000155506	LARP1	5:1540924	525.586	451.108	-0.153	3.091	0.002	0.029	yes	525.586	490.803	560.368	451.108	417.964	484.251
ENSG00000127616	ENSG00000127616	SMARCA4	19:110715	174.734	153.668	-0.128	3.035	0.002	0.034	yes	174.734	163.74	185.728	153.668	144.966	162.371
ENSG00000218521	ENSG00000218521	RP11-420A21.1	6:3778727	2.711	0	-1.79769e+	-1.79769e+	0.002	0.033	yes	2.71079	0.796139	4.62545	0	0	0
ENSG00000233202	ENSG00000233202	AC006028.10	7:2719155	2.287	0	-1.79769e+	-1.79769e+	0.002	0.03	yes	2.28675	0.695573	3.87792	0	0	0
ENSG00000239737	ENSG00000239737	AC113171.1	3:6770512	19.265	0	-1.79769e+	-1.79769e+	0.002	0.033	yes	19.2652	5.64264	32.8877	0	0	0
ENSG00000255354	ENSG00000255354	RP11-148O21.2	8:1135150	0.279	0	-1.79769e+	-1.79769e+	0.002	0.033	yes	0.279034	0.0817271	0.47634	0	0	0
ENSG00000078319	ENSG00000078319	PMS2P1	7:9990532	3.286	0.193	-2.837	2.925	0.003	0.047	yes	3.28623	0	6.82941	0.192554	0	0.503037
ENSG00000215298	ENSG00000215298	AC051642.1	8:2338631	0.809	0.048	-2.83	3.011	0.003	0.037	yes	0.809296	0.467035	1.15156	0.0477377	0	0.135193
ENSG00000250709	ENSG00000250709	CCDC169-SOHLH2	13:367423	0.277	0.031	-2.201	2.958	0.003	0.043	yes	0.277458	0	0.646647	0.0307072	0.010236	0.051179
ENSG00000230798	ENSG00000230798	RP4-792G4.2	1:6378655	3.137	0.577	-1.694	2.957	0.003	0.043	yes	3.13696	1.34667	4.92726	0.576644	0.003866	1.14942
ENSG00000205089	ENSG00000205089	CCNI2	5:1320831	5.401	1.009	-1.678	2.997	0.003	0.038	yes	5.40109	2.28062	8.52155	1.00891	0.041542	1.97628
ENSG00000074416	ENSG00000074416	MGLL	3:1274079	27.297	5.328	-1.634	3.014	0.003	0.036	yes	27.2973	7.56742	47.0271	5.32811	1.02293	9.6333
ENSG00000257918	ENSG00000257918	RP11-482D24.3	12:106751	0.591	0.128	-1.532	2.974	0.003	0.041	yes	0.590593	0	1.19757	0.12757	0.117857	0.137283
ENSG00000154783	ENSG00000154783	FGD5	3:1486046	0.855	0.236	-1.288	2.936	0.003	0.045	yes	0.855072	0.443315	1.26683	0.235765	0.062756	0.408775
ENSG00000178297	ENSG00000178297	TMPRSS9	19:238976	1.007	0.319	-1.148	2.944	0.003	0.044	yes	1.00657	0.594794	1.41834	0.319353	0.10726	0.531445
ENSG00000139173	ENSG00000139173	TMEM117	12:442297	7.2	2.656	-0.997	2.978	0.003	0.04	yes	7.20016	4.62396	9.77635	2.65638	1.15238	4.16038
ENSG00000225302	ENSG00000225302	RP11-539I5.1	10:118592	9.35	4.217	-0.796	2.937	0.003	0.045	yes	9.34998	6.77824	11.9217	4.21666	2.2458	6.18751
ENSG00000253485	ENSG00000253485	PCDHGA5	5:1407102	1.028	0.467	-0.789	2.943	0.003	0.045	yes	1.02772	0.730322	1.32511	0.466692	0.255847	0.677538
ENSG00000125954	ENSG00000125954	FNTB	14:653810	78.205	39.432	-0.685	2.956	0.003	0.043	yes	78.2045	51.9922	104.417	39.4322	26.8216	52.0427
ENSG00000066136	ENSG00000066136	NFYC	1:4115731	64.935	38.572	-0.521	2.952	0.003	0.043	yes	64.9346	46.5467	83.3225	38.5717	30.4482	46.6952
ENSG00000226800	ENSG00000226800	C19orf29OS	19:361062	6.126	3.664	-0.514	2.924	0.003	0.047	yes	6.12596	4.78808	7.46384	3.66354	2.65379	4.67329
ENSG00000134014	ENSG00000134014	ELP3	8:2794718	44.023	26.961	-0.49	2.922	0.003	0.047	yes	44.0233	32.2823	55.7642	26.9606	21.4685	32.4528
ENSG00000164022	ENSG00000164022	AIMP1	4:1069654	18.421	11.452	-0.475	2.958	0.003	0.043	yes	18.4209	14.879	21.9628	11.4516	8.50244	14.4008
ENSG00000089775	ENSG00000089775	ZBTB25	14:648547	9.504	5.99	-0.462	2.955	0.003	0.043	yes	9.50394	7.37723	11.6307	5.98986	4.68373	7.296
ENSG00000215695	ENSG00000215695	RSC1A1	1:1594399	8.09	5.155	-0.451	2.983	0.003	0.04	yes	8.08981	6.51858	9.66103	5.15458	3.9609	6.34826
ENSG00000170144	ENSG00000170144	HNRNPA3	2:1778552	26.041	17.742	-0.384	2.985	0.003	0.04	yes	26.0412	21.3386	30.7439	17.7421	14.4947	20.9896
ENSG00000170873	ENSG00000170873	MTSS1	8:1255007	22.318	15.22	-0.383	2.98	0.003	0.04	yes	22.3177	19.4824	25.153	15.2197	11.8206	18.6188
ENSG00000168818	ENSG00000168818	STX18	4:4349866	27.034	19.045	-0.35	2.989	0.003	0.039	yes	27.0335	23.0081	31.059	19.0446	15.5972	22.4921
ENSG00000105675	ENSG00000105675	ATP4A	19:360410	11.565	8.399	-0.32	2.996	0.003	0.038	yes	11.565	9.92503	13.205	8.3989	7.05818	9.73962
ENSG00000235590	ENSG00000235590	GNAS-AS1	20:573939	42.762	31.113	-0.318	2.964	0.003	0.042	yes	42.7619	36.6718	48.8519	31.1131	26.1205	36.1057
ENSG00000133943	ENSG00000133943	C14orf159	14:913367	15.282	11.238	-0.307	2.938	0.003	0.045	yes	15.2815	13.1539	17.4092	11.2381	9.48338	12.9928
ENSG00000233558	ENSG00000233558	RP3-486I3.4	6:1165753	114.109	85.084	-0.294	2.928	0.003	0.047	yes	114.109	98.813	129.405	85.084	72.4007	97.7672
ENSG00000166200	ENSG00000166200	COPS2	15:494174	11.349	8.525	-0.286	2.987	0.003	0.039	yes	11.3491	9.78781	12.9103	8.52484	7.38801	9.66168

ENSG00000204209	ENSG00000204209	DAXX	6:3328633	134.173	102.334	-0.271	2.932	0.003	0.046	yes	134.173	113.208	155.139	102.334	92.2345	112.433
ENSG00000112079	ENSG00000112079	STK38	6:3646166	29.866	23.385	-0.245	2.95	0.003	0.044	yes	29.8656	26.4998	33.2314	23.3852	20.54	26.2304
ENSG00000172534	ENSG00000172534	HCFC1	X:1532130	114.268	89.7	-0.242	2.922	0.003	0.047	yes	114.268	100.942	127.594	89.7002	79.1439	100.257
ENSG00000161179	ENSG00000161179	YDJC	22:219823	104.886	82.58	-0.239	2.944	0.003	0.044	yes	104.886	92.4463	117.326	82.5804	73.4178	91.743
ENSG00000151148	ENSG00000151148	UBE3B	12:109826	57.402	45.745	-0.227	2.926	0.003	0.047	yes	57.4017	51.9016	62.9019	45.7451	40.164	51.3263
ENSG00000173281	ENSG00000173281	PPP1R3B	8:8993764	20.891	16.659	-0.226	2.936	0.003	0.045	yes	20.8914	18.7215	23.0612	16.6592	14.7602	18.5581
ENSG00000103037	ENSG00000103037	SETD6	16:585493	90.787	72.665	-0.223	2.945	0.003	0.044	yes	90.7872	81.7658	99.8085	72.6647	64.3819	80.9475
ENSG00000130717	ENSG00000130717	UCK1	9:1343782	88.451	70.81	-0.222	2.999	0.003	0.038	yes	88.4513	78.141	98.7616	70.81	64.3126	77.3073
ENSG00000196372	ENSG00000196372	ASB13	10:568082	174.972	140.729	-0.218	2.949	0.003	0.044	yes	174.972	157.518	192.427	140.729	125.396	156.063
ENSG00000005007	ENSG00000005007	UPF1	19:189427	101.494	83.855	-0.191	2.986	0.003	0.039	yes	101.494	92.4212	110.567	83.8551	76.1883	91.5219
ENSG00000122952	ENSG00000122952	ZWINT	10:581169	187.854	155.884	-0.187	2.989	0.003	0.039	yes	187.854	170.018	205.69	155.884	143.252	168.516
ENSG00000183283	ENSG00000183283	DAZAP2	12:516320	260.461	219.743	-0.17	3.019	0.003	0.036	yes	260.461	240.604	280.318	219.743	201.534	237.953
ENSG00000161642	ENSG00000161642	ZNF385A	12:547474	152.202	128.805	-0.167	2.979	0.003	0.04	yes	152.202	140.734	163.669	128.805	118.122	139.487
ENSG00000242583	ENSG00000242583	RP11-379K17.9	3:1697557	1.441	0	-1.79769e+	-1.79769e+	0.003	0.039	yes	1.44089	0.402497	2.47928	0	0	0
ENSG00000251639	ENSG00000251639	RP11-20I20.1	4:1050037	0.739	0	-1.79769e+	-1.79769e+	0.003	0.04	yes	0.738571	0.203002	1.27414	0	0	0
ENSG00000259133	ENSG00000259133	RP11-1085N6.3	14:569550	2.189	0	-1.79769e+	-1.79769e+	0.003	0.04	yes	2.18927	0.606416	3.77213	0	0	0
ENSG00000061455	ENSG00000061455	PRDM6	5:1224229	10.029	3.128	-1.165	2.919	0.004	0.048	yes	10.0286	3.64268	16.4145	3.12822	1.6228	4.63364
ENSG00000186352	ENSG00000186352	ANKRD37	4:1863171	8.247	2.891	-1.048	2.909	0.004	0.049	yes	8.24749	5.42123	11.0738	2.89106	1.05775	4.72437
ENSG00000182575	ENSG00000182575	NXPH3	17:475726	4.553	2.677	-0.531	2.908	0.004	0.049	yes	4.55333	3.55131	5.55535	2.67705	1.8963	3.45779
ENSG00000257108	ENSG00000257108	NHLRC4	16:616994	5.078	3.126	-0.485	2.913	0.004	0.048	yes	5.07811	4.06708	6.08915	3.12628	2.29177	3.9608
ENSG00000125733	ENSG00000125733	TRIP10	19:673969	88.638	59.327	-0.401	2.915	0.004	0.048	yes	88.6381	68.886	108.39	59.3273	49.7205	68.934
ENSG00000112339	ENSG00000112339	HBS1L	6:1352815	53.622	39.841	-0.297	2.911	0.004	0.049	yes	53.622	46.5563	60.6877	39.8412	33.6304	46.0519
ENSG00000233016	ENSG00000233016	SNHG7	9:1396070	39.393	29.427	-0.292	2.908	0.004	0.049	yes	39.3929	34.2458	44.54	29.4266	24.9467	33.9065
ENSG00000010256	ENSG00000010256	UQCRC1	3:4863643	412.61	359.839	-0.137	2.904	0.004	0.05	yes	412.61	384.709	440.512	359.839	336.208	383.47
ENSG00000140416	ENSG00000140416	TPM1	15:633348	450.536	395.21	-0.131	2.911	0.004	0.049	yes	450.536	424.424	476.648	395.21	367.986	422.434
ENSG00000229674	ENSG00000229674	AL121578.7	X:3720852	0.123	0	-1.79769e+	-1.79769e+	2.885429	3.236449	yes	0.122716	0.116257	0.129175	0	0	0
ENSG00000224386	ENSG00000224386	AC090939.2	3:1131399	0.29	0	-1.79769e+	-1.79769e+	8.649330	9.65954e	yes	0.289835	0.274496	0.305175	0	0	0
