# Supplementary material for: Prosaposin activates the androgen receptor and potentiates resistance to endocrine treatment in breast cancer
Source: Breast Cancer Res. 2015 Sep 4;17(1):123. doi: 10.1186/s13058-015-0636-6 (PMC4560928; doi:10.1186/s13058-015-0636-6)
Supplement: Additional file 3: — Table: HOXC11 KD upregulated genes. HOXC11 differentially expressed genes (DEGs) upregulated after HOXC11 knockdown, ranked by p value. (PDF 259 kb) [file 13058_2015_636_MOESM3_ESM.pdf]

test_id	gene_id	gene	locus	sample_1	sample_2	ln.fold_cha	test_stat	p_value	q_value	significant	q1_FPKM	q1_conf_lo	q1_conf_hi	q2_FPKM	q2_conf_lo	q2_conf_hi
ENSG00000254088	ENSG00000254088	CTD-3083F21.5	8:8734550	q1	q2	0.138	-6.064	0	0	yes	0.0653416	0.0632964	0.0673868	0.0750096	0.0725312	0.077488
ENSG00000148180	ENSG00000148180	GSN	9:1239404	q1	q2	0.143	-3.541	0	0.007	yes	266.804	252.157	281.452	307.83	289.588	326.072
ENSG00000127022	ENSG00000127022	CANX	5:1791055	q1	q2	0.174	-4.529	0	0	yes	1501.79	1420.2	1583.37	1786.8	1689.95	1883.65
ENSG00000109685	ENSG00000109685	WHSC1	4:1873150	q1	q2	0.176	-3.838	0	0.003	yes	219.244	204.592	233.897	261.558	245.033	278.083
ENSG00000238300	ENSG00000238300	SNORD121B	9:3392169	q1	q2	0.176	-4.437	0	0	yes	23.4885	22.1984	24.7786	28.0206	26.4088	29.6323
ENSG00000065361	ENSG00000065361	ERBB3	12:564736	q1	q2	0.18	-3.662	0	0.005	yes	558.411	520.192	596.631	668.321	621.329	715.313
ENSG00000075415	ENSG00000075415	SLC25A3	12:989873	q1	q2	0.183	-4.012	0	0.001	yes	610.908	573.155	648.661	733.341	684.291	782.39
ENSG00000121716	ENSG00000121716	PILRB	7:9990532	q1	q2	0.183	-4.205	0	0.001	yes	252.223	236.658	267.787	302.945	284.3	321.591
ENSG00000236658	ENSG00000236658	RP11-83J21.3	9:1337778	q1	q2	0.192	-3.823	0	0.003	yes	0.261529	0.243724	0.279334	0.316762	0.293458	0.340067
ENSG00000250893	ENSG00000250893	RP11-588L15.2	4:4042527	q1	q2	0.201	-5.087	0	0	yes	0.0553138	0.0523497	0.0582778	0.0676364	0.0637034	0.0715693
ENSG00000142949	ENSG00000142949	PTPRF	1:4399085	q1	q2	0.202	-3.755	0	0.003	yes	319.859	297.485	342.232	391.345	359.429	423.26
ENSG00000077097	ENSG00000077097	TOP2B	3:2563947	q1	q2	0.203	-4.017	0	0.001	yes	201.972	187.025	216.919	247.54	230.419	264.662
ENSG00000099622	ENSG00000099622	CIRBP	19:126926	q1	q2	0.207	-3.513	0	0.008	yes	151.313	138.795	163.832	186.024	170.48	201.568
ENSG00000092841	ENSG00000092841	MYL6	12:565445	q1	q2	0.21	-4.928	0	0	yes	841.64	790.789	892.492	1038.19	975.85	1100.53
ENSG00000147677	ENSG00000147677	EIF3H	8:1176543	q1	q2	0.21	-3.963	0	0.002	yes	241.053	222.892	259.215	297.505	275.248	319.762
ENSG00000168394	ENSG00000168394	TAP1	6:3280849	q1	q2	0.213	-3.845	0	0.002	yes	78.2332	72.0466	84.4197	96.7631	89.2844	104.242
ENSG00000156482	ENSG00000156482	RPL30	8:9888106	q1	q2	0.215	-4.7	0	0	yes	1552.52	1452.15	1652.9	1924.03	1800.01	2048.05
ENSG00000012963	ENSG00000012963	UBR7	14:936512	q1	q2	0.218	-3.676	0	0.005	yes	215.636	197.395	233.876	268.288	245.886	290.69
ENSG00000104320	ENSG00000104320	NBN	8:9094556	q1	q2	0.218	-3.876	0	0.002	yes	122.891	113.066	132.716	152.9	140.749	165.052
ENSG00000114503	ENSG00000114503	NCBP2	3:1966622	q1	q2	0.221	-3.719	0	0.004	yes	168.246	153.756	182.735	209.898	192.675	227.121
ENSG00000186298	ENSG00000186298	PPP1CC	12:111157	q1	q2	0.226	-3.533	0	0.007	yes	161.38	146.925	175.834	202.39	183.834	220.946
ENSG00000168883	ENSG00000168883	USP39	2:8582567	q1	q2	0.229	-4.104	0	0.001	yes	274.572	254.056	295.088	345.299	316.635	373.963
ENSG00000125991	ENSG00000125991	ERGIC3	20:341297	q1	q2	0.233	-6.004	0	0	yes	682.596	647.309	717.882	861.509	811.723	911.294
ENSG00000197451	ENSG00000197451	HNRNPAB	5:1776315	q1	q2	0.233	-4.593	0	0	yes	291.289	268.866	313.713	367.555	343.372	391.737
ENSG00000204498	ENSG00000204498	NFKBIL1	6:3149649	q1	q2	0.233	-3.711	0	0.004	yes	74.0863	67.2994	80.8732	93.5382	85.4951	101.581
ENSG00000111331	ENSG00000111331	OAS3	12:113344	q1	q2	0.234	-5.26	0	0	yes	300.426	281.054	319.798	379.639	356.362	402.916
ENSG00000245532	ENSG00000245532	NEAT1	11:651902	q1	q2	0.234	-3.496	0	0.008	yes	124.508	112.61	136.406	157.33	142.586	172.074
ENSG00000100316	ENSG00000100316	RPL3	22:397088	q1	q2	0.236	-3.671	0	0.005	yes	232.2	207.98	256.42	294.043	271.907	316.179
ENSG00000156508	ENSG00000156508	EEF1A1	6:7422547	q1	q2	0.241	-5.068	0	0	yes	318.631	296.894	340.368	405.581	378.636	432.527
ENSG00000196542	ENSG00000196542	SPTSSB	3:1610625	q1	q2	0.241	-4.286	0	0	yes	292.816	268.92	316.712	372.583	343.774	401.393
ENSG00000130589	ENSG00000130589	RP4-697K14.7	20:621894	q1	q2	0.243	-4.765	0	0	yes	64.1454	59.4528	68.8379	81.8203	75.9861	87.6544
ENSG00000110851	ENSG00000110851	PRDM4	12:108126	q1	q2	0.246	-4.502	0	0	yes	138.171	127.6	148.741	176.752	162.93	190.573
ENSG00000213281	ENSG00000213281	NRAS	1:1152470	q1	q2	0.246	-3.716	0	0.004	yes	67.9163	61.5001	74.3325	86.8712	78.8014	94.941
ENSG00000166710	ENSG00000166710	B2M	15:450036	q1	q2	0.249	-3.729	0	0.004	yes	489.045	441.975	536.114	627.431	569.256	685.606
ENSG00000229117	ENSG00000229117	RPL41	12:565103	q1	q2	0.249	-4.59	0	0	yes	1829.9	1692.94	1966.86	2347.77	2163.04	2532.5
ENSG00000100234	ENSG00000100234	TIMP3	22:329085	q1	q2	0.25	-3.527	0	0.008	yes	39.2913	35.6224	42.9601	50.4293	45.0662	55.7925
ENSG00000109854	ENSG00000109854	HTATIP2	11:203852	q1	q2	0.25	-3.928	0	0.002	yes	101.935	92.7618	111.108	130.932	119.11	142.753
ENSG00000198431	ENSG00000198431	TXNRD1	12:104609	q1	q2	0.251	-5.476	0	0	yes	246.484	229.866	263.103	316.952	297.19	336.715
ENSG00000104549	ENSG00000104549	SQLE	8:1260107	q1	q2	0.254	-3.485	0	0.009	yes	112.345	100.777	123.913	144.893	129.896	159.89
ENSG00000215301	ENSG00000215301	DDX3X	X:4119265	q1	q2	0.254	-4.097	0	0.001	yes	57.1094	52.1342	62.0846	73.5882	67.1204	80.056
ENSG00000115415	ENSG00000115415	STAT1	2:1917455	q1	q2	0.256	-6.145	0	0	yes	384.915	361.753	408.077	497.436	468.668	526.205
ENSG00000242517	ENSG00000242517	AC018607.3	8:5675503	q1	q2	0.257	-3.837	0	0.003	yes	2392.65	2163.94	2621.35	3093.48	2803.44	3383.51
ENSG00000071082	ENSG00000071082	RPL31	2:1014366	q1	q2	0.258	-3.651	0	0.005	yes	613.624	551.473	675.774	794.203	715.957	872.449
ENSG00000105397	ENSG00000105397	TYK2	19:104612	q1	q2	0.261	-3.837	0	0.003	yes	243.592	218.957	268.228	316.285	287.464	345.106
ENSG00000182934	ENSG00000182934	SRPR	11:126071	q1	q2	0.261	-4.455	0	0	yes	105.6	96.5743	114.626	137.047	126.095	147.998
ENSG00000163126	ENSG00000163126	ANKRD23	2:9748198	q1	q2	0.268	-3.861	0	0.002	yes	30.9762	27.8678	34.0845	40.5154	36.6156	44.4151
ENSG00000104388	ENSG00000104388	RAB2A	8:6142941	q1	q2	0.269	-3.667	0	0.005	yes	145.17	129.821	160.519	189.96	170.649	209.271
ENSG00000121152	ENSG00000121152	NCAPH	2:9700152	q1	q2	0.27	-3.494	0	0.008	yes	66.0496	58.6367	73.4626	86.5119	77.3276	95.6962
ENSG00000124201	ENSG00000124201	ZNFX1	20:478269	q1	q2	0.271	-3.793	0	0.003	yes	24.3481	21.8589	26.8373	31.9296	28.7397	35.1195

ENSG00000104529	ENSG00000104529	EEF1D	8:1446618	q1	q2	0.272	-5.54	0	0	yes	291.101	271.7	310.502	382.251	354.611	409.891
ENSG00000104325	ENSG00000104325	DECR1	8:9094556	q1	q2	0.274	-4.61	0	0	yes	205.44	188.311	222.568	270.063	247.262	292.864
ENSG00000066583	ENSG00000066583	ISOC1	5:1284303	q1	q2	0.278	-4.357	0	0	yes	131.958	119.8	144.116	174.225	158.855	189.595
ENSG00000148303	ENSG00000148303	RPL7A	9:1362150	q1	q2	0.281	-4.879	0	0	yes	254.209	235.548	272.869	336.609	306.767	366.451
ENSG00000249915	ENSG00000249915	PDCD6	5:271735-4	q1	q2	0.282	-3.578	0	0.006	yes	145.411	128.893	161.93	192.704	171.711	213.696
ENSG00000060656	ENSG00000060656	PTPRU	1:2956302	q1	q2	0.284	-3.543	0	0.007	yes	56.6664	50.1764	63.1564	75.2706	66.8348	83.7064
ENSG00000130638	ENSG00000130638	ATXN10	22:460676	q1	q2	0.287	-3.9	0	0.002	yes	90.9703	81.3846	100.556	121.266	108.765	133.767
ENSG00000145014	ENSG00000145014	TMEM44	3:1943047	q1	q2	0.289	-3.622	0	0.005	yes	42.2306	36.9308	47.5305	56.3977	50.8244	61.9711
ENSG00000063177	ENSG00000063177	RPL18	19:491185	q1	q2	0.291	-5.728	0	0	yes	420.757	389.528	451.986	562.849	523.811	601.886
ENSG00000035115	ENSG00000035115	SH3YL1	2:217729-2	q1	q2	0.292	-3.929	0	0.002	yes	78.0407	69.125	86.9563	104.529	94.5707	114.488
ENSG00000213741	ENSG00000213741	RPS29	14:500433	q1	q2	0.296	-4.217	0	0.001	yes	1786.86	1607.04	1966.68	2401.85	2167.09	2636.61
ENSG00000117632	ENSG00000117632	STMN1	1:2621067	q1	q2	0.301	-3.632	0	0.005	yes	78.5976	68.7119	88.4833	106.151	94.7368	117.566
ENSG00000257298	ENSG00000257298	RP3-405J10.3	12:505695	q1	q2	0.303	-4.772	0	0	yes	0.007575	0.0068906	0.00825942	0.0102592	0.0093416	0.0111768
ENSG00000115211	ENSG00000115211	EIF2B4	2:2750525	q1	q2	0.304	-5.364	0	0	yes	119.488	109.051	129.924	161.917	150.23	173.605
ENSG00000138496	ENSG00000138496	PARP9	3:1222468	q1	q2	0.308	-4.798	0	0	yes	51.5617	46.6803	56.4431	70.1782	64.0808	76.2756
ENSG00000198804	ENSG00000198804	MT-CO1	MT:5903-7	q1	q2	0.311	-4.147	0	0.001	yes	92.0499	82.0905	102.009	125.662	112.586	138.738
ENSG00000198840	ENSG00000198840	MT-ND3	MT:10058-	q1	q2	0.312	-4.981	0	0	yes	3783.89	3446.62	4121.17	5171.85	4715.17	5628.52
ENSG00000084234	ENSG00000084234	APLP2	11:129939	q1	q2	0.317	-6.572	0	0	yes	434.186	401.741	466.631	596.26	559.827	632.693
ENSG00000147684	ENSG00000147684	NDUFB9	8:1255007	q1	q2	0.318	-4.891	0	0	yes	213.408	193.247	233.568	293.33	267.101	319.559
ENSG00000244097	ENSG00000244097	RP11-411G7.1	17:411907	q1	q2	0.318	-6.376	0	0	yes	0.107101	0.09967	0.114531	0.147258	0.136679	0.157838
ENSG00000074582	ENSG00000074582	BCS1L	2:2195026	q1	q2	0.321	-4.568	0	0	yes	85.8338	77.2009	94.4668	118.335	106.712	129.959
ENSG00000106608	ENSG00000106608	URGCP	7:4391549	q1	q2	0.322	-4.338	0	0	yes	55.0616	49.5931	60.5301	75.9832	67.5949	84.3716
ENSG00000215845	ENSG00000215845	TSTD1	1:1609650	q1	q2	0.326	-4.758	0	0	yes	202.935	182.178	223.691	281.134	255.504	306.764
ENSG00000135317	ENSG00000135317	SNX14	6:8621521	q1	q2	0.327	-3.511	0	0.008	yes	97.6396	84.1754	111.104	135.432	118.453	152.412
ENSG00000143379	ENSG00000143379	SETDB1	1:1508987	q1	q2	0.331	-5.395	0	0	yes	99.475	90.7345	108.216	138.511	126.645	150.377
ENSG00000116857	ENSG00000116857	TMEM9	1:2011038	q1	q2	0.332	-5.455	0	0	yes	154.371	142.42	166.321	215.075	194.906	235.243
ENSG00000128595	ENSG00000128595	CALU	7:1283793	q1	q2	0.333	-4.067	0	0.001	yes	52.372	46.9089	57.8351	73.0973	63.8509	82.3437
ENSG00000227638	ENSG00000227638	HNRNPA1P14	9:9100333	q1	q2	0.333	-6.976	0	0	yes	0.132466	0.123615	0.141316	0.184724	0.172163	0.197286
ENSG00000003056	ENSG00000003056	M6PR	12:906649	q1	q2	0.334	-4.041	0	0.001	yes	152.664	137.144	168.184	213.199	185.41	240.988
ENSG00000122565	ENSG00000122565	CBX3	7:2622954	q1	q2	0.335	-3.809	0	0.003	yes	24.4464	21.289	27.6038	34.1862	30.0949	38.2774
ENSG00000167635	ENSG00000167635	ZNF146	19:366731	q1	q2	0.337	-3.863	0	0.002	yes	9.76752	8.51166	11.0234	13.6799	12.0674	15.2924
ENSG00000166598	ENSG00000166598	HSP90B1	12:104235	q1	q2	0.338	-5.24	0	0	yes	119.713	108.82	130.605	167.822	152.492	183.152
ENSG00000228779	ENSG00000228779	Z69666.2	16:127005	q1	q2	0.346	-13.598	0	0	yes	0.0245901	0.023726	0.0254543	0.0347725	0.0334892	0.0360558
ENSG00000129484	ENSG00000129484	PARP2	14:208112	q1	q2	0.348	-3.622	0	0.005	yes	36.4066	31.2439	41.5694	51.5452	44.875	58.2154
ENSG00000111335	ENSG00000111335	OAS2	12:113344	q1	q2	0.352	-6.292	0	0	yes	159.191	146.148	172.235	226.287	209.079	243.495
ENSG00000176444	ENSG00000176444	CLK2	1:1552326	q1	q2	0.353	-4.137	0	0.001	yes	52.0518	45.3076	58.796	74.0722	65.8585	82.2859
ENSG00000244336	ENSG00000244336	RP11-479G22.3	10:331892	q1	q2	0.356	-8.677	0	0	yes	0.107995	0.101905	0.114085	0.154195	0.144996	0.163393
ENSG00000170889	ENSG00000170889	RPS9	19:547046	q1	q2	0.357	-4.938	0	0	yes	66.9488	59.9392	73.9585	95.6346	86.1217	105.148
ENSG00000135245	ENSG00000135245	C7orf68	7:1280958	q1	q2	0.358	-3.866	0	0.002	yes	34.8293	30.1179	39.5407	49.8044	43.5178	56.091
ENSG00000133193	ENSG00000133193	FAM104A	17:711887	q1	q2	0.361	-4.081	0	0.001	yes	16.5171	14.3284	18.7058	23.7095	20.9213	26.4978
ENSG00000067182	ENSG00000067182	TNFRSF1A	12:643792	q1	q2	0.362	-4.977	0	0	yes	136.666	124.374	148.957	196.207	173.813	218.6
ENSG00000176532	ENSG00000176532	PRR15	7:2960342	q1	q2	0.364	-3.523	0	0.008	yes	50.1918	41.7147	58.669	72.253	63.6285	80.8776
ENSG00000172819	ENSG00000172819	RARG	12:536043	q1	q2	0.368	-4.262	0	0	yes	108.018	94.8999	121.137	156.119	136.92	175.317
ENSG00000109046	ENSG00000109046	WSB1	17:256211	q1	q2	0.37	-4.484	0	0	yes	25.9011	22.7393	29.0629	37.5032	33.3337	41.6726
ENSG00000127399	ENSG00000127399	LRRC61	7:1499410	q1	q2	0.37	-3.569	0	0.007	yes	17.5906	15.0793	20.102	25.4566	21.6373	29.2759
ENSG00000143776	ENSG00000143776	CDC42BPA	1:2271775	q1	q2	0.37	-3.667	0	0.005	yes	6.46025	5.71098	7.20953	9.35174	7.80829	10.8952
ENSG00000135722	ENSG00000135722	FBXL8	16:671938	q1	q2	0.374	-4.306	0	0	yes	47.9104	41.8484	53.9725	69.6598	61.3559	77.9637
ENSG00000139637	ENSG00000139637	C12orf10	12:536890	q1	q2	0.374	-5.537	0	0	yes	83.3832	74.6131	92.1532	121.17	110.916	131.424
ENSG00000072518	ENSG00000072518	MARK2	11:636063	q1	q2	0.375	-4.541	0	0	yes	44.9726	40.9514	48.9938	65.4346	56.3477	74.5215
ENSG00000124942	ENSG00000124942	AHNAK	11:622010	q1	q2	0.377	-5.151	0	0	yes	99.0161	87.5144	110.518	144.404	131.508	157.299

ENSG00000129810	ENSG00000129810	SGOL1	3:2020208	q1	q2	0.38	-3.795	0	0.003	yes	16.0828	13.6479	18.5176	23.5275	20.4346	26.6203
ENSG00000165533	ENSG00000165533	TTC8	14:892904	q1	q2	0.382	-5.117	0	0	yes	76.539	69.3122	83.7658	112.191	99.1927	125.189
ENSG00000089159	ENSG00000089159	PXN	12:120639	q1	q2	0.384	-7.632	0	0	yes	122.669	113.708	131.63	180.094	167.631	192.557
ENSG00000143375	ENSG00000143375	CGN	1:1514829	q1	q2	0.385	-4.945	0	0	yes	177.584	154.407	200.761	260.935	238.81	283.061
ENSG00000064763	ENSG00000064763	FAR2	12:293020	q1	q2	0.392	-3.594	0	0.006	yes	20.2483	17.1214	23.3752	29.9619	25.3495	34.5744
ENSG00000120805	ENSG00000120805	ARL1	12:101786	q1	q2	0.392	-4.688	0	0	yes	118.891	102.961	134.82	175.927	158.341	193.513
ENSG00000010361	ENSG00000010361	FUZ	19:502701	q1	q2	0.393	-4.292	0	0	yes	30.6322	26.3055	34.959	45.3722	40.0875	50.657
ENSG00000093167	ENSG00000093167	LRRFIP2	3:3703482	q1	q2	0.397	-7.075	0	0	yes	18.8671	13.7537	23.9805	28.063	0	NA
ENSG00000111229	ENSG00000111229	ARPC3	12:110810	q1	q2	0.398	-4.062	0	0.001	yes	105.099	90.919	119.279	156.554	134.25	178.857
ENSG00000156411	ENSG00000156411	C14orf2	14:104378	q1	q2	0.399	-5.308	0	0	yes	828.436	738.77	918.102	1234.56	1105.79	1363.32
ENSG00000110619	ENSG00000110619	CARS	11:302215	q1	q2	0.402	-3.808	0	0.003	yes	17.1404	14.7315	19.5493	25.6145	21.5838	29.6452
ENSG00000163918	ENSG00000163918	RFC4	3:1865009	q1	q2	0.403	-4.239	0	0.001	yes	48.6845	41.7434	55.6256	72.8333	63.6785	81.9881
ENSG00000175197	ENSG00000175197	DDIT3	12:578539	q1	q2	0.404	-4.521	0	0	yes	35.965	31.0379	40.8921	53.8518	47.682	60.0216
ENSG00000111669	ENSG00000111669	TPI1	12:697628	q1	q2	0.412	-4.808	0	0	yes	66.7194	59.2188	74.22	100.733	87.704	113.762
ENSG00000152778	ENSG00000152778	IFIT5	10:909733	q1	q2	0.423	-3.724	0	0.004	yes	10.5977	8.81923	12.3761	16.1774	13.7002	18.6547
ENSG00000172375	ENSG00000172375	C2CD2L	11:118967	q1	q2	0.425	-5.279	0	0	yes	40.0166	35.1711	44.8622	61.2074	54.7121	67.7027
ENSG00000112576	ENSG00000112576	CCND3	6:4190267	q1	q2	0.426	-6.576	0	0	yes	165.754	150.657	180.852	253.899	230.455	277.342
ENSG00000254964	ENSG00000254964	RP11-831H9.3	11:623696	q1	q2	0.426	-11.42	0	0	yes	33.2317	31.4856	34.9777	50.8586	48.1706	53.5465
ENSG00000258051	ENSG00000258051	RP11-474C8.3	12:485415	q1	q2	0.426	-7.063	0	0	yes	0.086981	0.0795834	0.0943787	0.133195	0.121794	0.144595
ENSG00000219545	ENSG00000219545	AC006465.3	7:7676148	q1	q2	0.428	-4.309	0	0	yes	47.0215	40.2742	53.7687	72.1327	62.2257	82.0398
ENSG00000180389	ENSG00000180389	ATP5EP2	13:285193	q1	q2	0.429	-3.794	0	0.003	yes	118.863	98.9912	138.736	182.481	154.745	210.218
ENSG00000240857	ENSG00000240857	RDH14	2:1873598	q1	q2	0.43	-3.779	0	0.003	yes	17.7217	14.7422	20.7012	27.2499	23.065	31.4348
ENSG00000175110	ENSG00000175110	MRPS22	3:1387228	q1	q2	0.431	-4.579	0	0	yes	50.493	42.8069	58.1792	77.721	69.0943	86.3477
ENSG00000168237	ENSG00000168237	GLYCTK	3:5228843	q1	q2	0.432	-3.672	0	0.005	yes	8.31756	6.81485	9.82028	12.8108	10.8805	14.7411
ENSG00000100296	ENSG00000100296	THOC5	22:299018	q1	q2	0.433	-6.328	0	0	yes	66.936	59.6736	74.1984	103.16	94.5798	111.74
ENSG00000130024	ENSG00000130024	PHF10	6:1701022	q1	q2	0.433	-4.214	0	0.001	yes	10.0873	8.54992	11.6246	15.5494	13.4087	17.6901
ENSG00000049323	ENSG00000049323	LTBP1	2:3317203	q1	q2	0.434	-4.072	0	0.001	yes	39.6932	35.5591	43.8272	61.2532	49.8649	72.6414
ENSG00000189376	ENSG00000189376	C8orf76	8:1242321	q1	q2	0.435	-4.309	0	0	yes	29.6231	25.1591	34.0871	45.7441	39.6117	51.8765
ENSG00000184979	ENSG00000184979	USP18	22:186326	q1	q2	0.437	-4.275	0	0	yes	16.0267	13.6061	18.4473	24.812	21.3926	28.2314
ENSG00000214941	ENSG00000214941	ZSWIM7	17:158798	q1	q2	0.437	-4.82	0	0	yes	52.2105	45.0257	59.3954	80.8181	71.28	90.3561
ENSG00000163743	ENSG00000163743	RCHY1	4:7640424	q1	q2	0.439	-3.782	0	0.003	yes	23.7594	19.478	28.0407	36.8363	31.4615	42.2112
ENSG00000258839	ENSG00000258839	MC1R	16:899785	q1	q2	0.439	-3.819	0	0.003	yes	7.06202	5.85909	8.26495	10.9522	9.26261	12.6418
ENSG00000204264	ENSG00000204264	PSMB8	6:3280849	q1	q2	0.444	-6.658	0	0	yes	91.3814	82.3551	100.408	142.525	129.72	155.331
ENSG00000152082	ENSG00000152082	MZT2B	2:1309089	q1	q2	0.445	-3.567	0	0.007	yes	63.4122	51.9017	74.9227	98.9065	82.0089	115.804
ENSG00000149532	ENSG00000149532	CPSF7	11:611701	q1	q2	0.448	-8.328	0	0	yes	97.8711	90.3932	105.349	153.151	141.562	164.74
ENSG00000177628	ENSG00000177628	GBA	1:1552042	q1	q2	0.448	-3.727	0	0.004	yes	48.7267	40.2446	57.2088	76.2754	63.6194	88.9315
ENSG00000085274	ENSG00000085274	MYNN	3:1694908	q1	q2	0.453	-4.833	0	0	yes	6.30059	5.48401	7.11718	9.91307	8.5694	11.2567
ENSG00000138434	ENSG00000138434	SSFA2	2:1827565	q1	q2	0.457	-4.624	0	0	yes	457.007	389.35	524.664	721.679	627.235	816.123
ENSG00000096093	ENSG00000096093	EFHC1	6:5228510	q1	q2	0.458	-5.006	0	0	yes	14.218	12.2673	16.1688	22.4718	19.7544	25.1891
ENSG00000221978	ENSG00000221978	CCNL2	1:1321090	q1	q2	0.458	-6.606	0	0	yes	80.5214	72.9302	88.1127	127.344	114.375	140.313
ENSG00000175175	ENSG00000175175	PPM1E	17:568332	q1	q2	0.46	-4.979	0	0	yes	4.95612	4.27749	5.63475	7.85109	6.87696	8.82521
ENSG00000118260	ENSG00000118260	CREB1	2:2081043	q1	q2	0.463	-3.599	0	0.006	yes	16.594	13.1879	20.0001	26.3592	22.2739	30.4445
ENSG00000142156	ENSG00000142156	COL6A1	21:474016	q1	q2	0.464	-4.017	0	0.001	yes	23.5832	19.943	27.2233	37.5047	31.06	43.9495
ENSG00000126062	ENSG00000126062	TMEM115	3:5038476	q1	q2	0.466	-4.331	0	0	yes	12.7209	10.6882	14.7536	20.272	17.3498	23.1942
ENSG00000154274	ENSG00000154274	C4orf19	4:3745556	q1	q2	0.466	-3.818	0	0.003	yes	6.95623	5.66352	8.24893	11.0813	9.33071	12.8319
ENSG00000106028	ENSG00000106028	SSBP1	7:1414041	q1	q2	0.468	-4.97	0	0	yes	72.1397	63.5928	80.6866	115.202	98.3302	132.073
ENSG00000215424	ENSG00000215424	MCM3AP-AS1	21:476491	q1	q2	0.472	-3.867	0	0.002	yes	5.0881	4.13843	6.03776	8.15632	6.87398	9.43867
ENSG00000130813	ENSG00000130813	C19orf66	19:101968	q1	q2	0.474	-6.235	0	0	yes	32.7819	29.1284	36.4355	52.6756	47.2207	58.1305
ENSG00000008988	ENSG00000008988	RPS20	8:5697985	q1	q2	0.477	-7.165	0	0	yes	322.494	291.853	353.136	519.413	471.042	567.784
ENSG00000175324	ENSG00000175324	LSM1	8:3796275	q1	q2	0.48	-4.588	0	0	yes	30.2944	25.2957	35.2932	48.9731	42.6626	55.2837

ENSG00000083454	ENSG00000083454	P2RX5	17:356619	q1	q2	0.484	-5.876	0	0	yes	24.1544	21.2479	27.0609	39.2057	34.7882	43.6233
ENSG00000176148	ENSG00000176148	TCP11L1	11:330609	q1	q2	0.486	-4.204	0	0.001	yes	11.1362	9.29664	12.9757	18.1019	15.1759	21.0279
ENSG00000183684	ENSG00000183684	THOC4	17:798457	q1	q2	0.488	-5.789	0	0	yes	181.427	157.386	205.467	295.563	264.745	326.381
ENSG00000151468	ENSG00000151468	CCDC3	10:129386	q1	q2	0.49	-7.112	0	0	yes	0.0237562	0.0214209	0.0260915	0.0387679	0.0350284	0.0425075
ENSG00000000419	ENSG00000000419	DPM1	20:495514	q1	q2	0.491	-3.985	0	0.001	yes	31.7254	27.0244	36.4264	51.8412	41.6314	62.0509
ENSG00000115841	ENSG00000115841	FAM82A1	2:3815032	q1	q2	0.491	-3.528	0	0.008	yes	9.66154	7.75826	11.5648	15.7857	12.6822	18.8892
ENSG00000140323	ENSG00000140323	DISP2	15:406504	q1	q2	0.491	-3.652	0	0.005	yes	2.47	1.97284	2.96717	4.03597	3.31632	4.75562
ENSG00000173137	ENSG00000173137	ADCK5	8:1455967	q1	q2	0.491	-6.605	0	0	yes	34.7945	30.8998	38.6892	56.8369	51.2831	62.3908
ENSG00000093100	ENSG00000093100	XXbac-B461K10.	22:182704	q1	q2	0.496	-3.952	0	0.002	yes	13.3067	10.629	15.9843	21.8569	18.5717	25.1422
ENSG00000197892	ENSG00000197892	KIF13B	8:2892479	q1	q2	0.499	-4.376	0	0	yes	15.5391	12.5685	18.5097	25.6035	22.4123	28.7947
ENSG00000171497	ENSG00000171497	PPID	4:1595878	q1	q2	0.501	-3.587	0	0.006	yes	12.575	9.98888	15.1611	20.7627	16.8295	24.6958
ENSG00000180398	ENSG00000180398	MCFD2	2:4712641	q1	q2	0.507	-5.198	0	0	yes	35.2825	29.6761	40.8889	58.5773	51.9514	65.2032
ENSG00000189308	ENSG00000189308	LIN54	4:8373981	q1	q2	0.51	-4.063	0	0.001	yes	9.13667	7.41938	10.854	15.2163	12.6833	17.7492
ENSG00000149273	ENSG00000149273	RPS3	11:751105	q1	q2	0.511	-8.798	0	0	yes	1049.06	963.965	1134.15	1748.81	1603.37	1894.25
ENSG00000199916	ENSG00000199916	RMRP	9:3565775	q1	q2	0.511	-4.073	0	0.001	yes	207.086	168.097	246.074	345.303	287.943	402.664
ENSG00000173163	ENSG00000173163	COMMD1	2:6209522	q1	q2	0.512	-4.915	0	0	yes	40.9777	34.4961	47.4593	68.3461	59.0973	77.5949
ENSG00000243364	ENSG00000243364	EFNA4	1:1550362	q1	q2	0.514	-5.065	0	0	yes	18.3957	15.5349	21.2566	30.7593	26.7456	34.773
ENSG00000173465	ENSG00000173465	SSSCA1	11:653379	q1	q2	0.517	-6.078	0	0	yes	62.9712	55.0993	70.843	105.554	93.3965	117.711
ENSG00000100320	ENSG00000100320	RBFOX2	22:361347	q1	q2	0.518	-3.942	0	0.002	yes	98.8157	78.054	119.577	165.852	139.695	192.008
ENSG00000169762	ENSG00000169762	TAPT1	4:1616212	q1	q2	0.518	-4.504	0	0	yes	34.1532	28.1035	40.2028	57.3292	48.9192	65.7391
ENSG00000152942	ENSG00000152942	RAD17	5:6864681	q1	q2	0.519	-4.477	0	0	yes	11.4727	9.72114	13.2243	19.2768	15.9139	22.6398
ENSG00000196141	ENSG00000196141	SPATS2L	2:2011706	q1	q2	0.52	-6.16	0	0	yes	62.1816	54.2242	70.1391	104.624	93.0882	116.16
ENSG00000138400	ENSG00000138400	MDH1B	2:2076024	q1	q2	0.522	-4.437	0	0	yes	7.64561	6.28569	9.00553	12.8883	10.9014	14.8752
ENSG00000229127	ENSG00000229127	AC007038.7	2:2108672	q1	q2	0.527	-5.623	0	0	yes	0.0300466	0.0260134	0.0340798	0.0509128	0.0442419	0.0575836
ENSG00000113068	ENSG00000113068	PFDN1	5:1395542	q1	q2	0.531	-3.942	0	0.002	yes	31.0809	25.4195	36.7423	52.8525	42.3634	63.3417
ENSG00000152503	ENSG00000152503	TRIM36	5:1144604	q1	q2	0.531	-5.977	0	0	yes	47.5393	42.4403	52.6382	80.8251	69.3857	92.2644
ENSG00000171863	ENSG00000171863	RPS7	2:3616112	q1	q2	0.533	-4.539	0	0	yes	20.5953	16.6692	24.5213	35.0819	30.2808	39.883
ENSG00000247315	ENSG00000247315	AL034548.2	20:277736	q1	q2	0.535	-3.842	0	0.002	yes	5.10382	4.03321	6.17444	8.71429	7.11842	10.3102
ENSG00000173210	ENSG00000173210	ABLIM3	5:1485210	q1	q2	0.537	-5.013	0	0	yes	11.9254	10.2995	13.5512	20.3983	17.0291	23.7676
ENSG00000181381	ENSG00000181381	DDX60L	4:1692778	q1	q2	0.542	-3.95	0	0.002	yes	8.18907	6.80969	9.56846	14.086	11.0307	17.1413
ENSG00000197548	ENSG00000197548	ATG7	3:1131399	q1	q2	0.542	-6.8	0	0	yes	76.5103	67.5007	85.5199	131.577	117.431	145.722
ENSG00000214549	ENSG00000214549	RP11-360G10.2	10:938083	q1	q2	0.544	-4.856	0	0	yes	0.184102	0.156108	0.212096	0.317096	0.264966	0.369227
ENSG00000151445	ENSG00000151445	VIPAR	14:778930	q1	q2	0.547	-6.275	0	0	yes	30.4652	25.9908	34.9395	52.6323	47.696	57.5686
ENSG00000198042	ENSG00000198042	MAK16	8:3322834	q1	q2	0.548	-3.896	0	0.002	yes	10.4381	8.22519	12.651	18.0543	14.7161	21.3926
ENSG00000134490	ENSG00000134490	C18orf45	18:207145	q1	q2	0.558	-4.919	0	0	yes	10.4934	8.49636	12.4905	18.3343	16.0689	20.5997
ENSG00000168887	ENSG00000168887	C2orf68	2:8582567	q1	q2	0.559	-7.116	0	0	yes	54.3149	47.9425	60.6873	94.9474	85.0464	104.848
ENSG00000125148	ENSG00000125148	MT2A	16:566421	q1	q2	0.56	-4.622	0	0	yes	27.8804	22.7877	32.9731	48.8031	41.0378	56.5684
ENSG00000160710	ENSG00000160710	ADAR	1:1545545	q1	q2	0.561	-10.811	0	0	yes	202.253	188.515	215.992	354.513	326.675	382.35
ENSG00000213204	ENSG00000213204	C6orf165	6:8811770	q1	q2	0.561	-5.185	0	0	yes	8.95912	7.41371	10.5045	15.7049	13.6499	17.76
ENSG00000115267	ENSG00000115267	IFIH1	2:1631235	q1	q2	0.565	-4.638	0	0	yes	25.8069	20.9724	30.6414	45.4255	38.3372	52.5139
ENSG00000124802	ENSG00000124802	EEF1E1	6:7726331	q1	q2	0.566	-3.807	0	0.003	yes	17.9549	13.6585	22.2513	31.6363	26.0403	37.2323
ENSG00000235092	ENSG00000235092	AC011747.7	2:8806765	q1	q2	0.567	-3.843	0	0.002	yes	21.4756	16.5959	26.3552	37.8493	30.7313	44.9673
ENSG00000231357	ENSG00000231357	AC006028.11	7:2719155	q1	q2	0.568	-3.708	0	0.004	yes	38.2741	29.3974	47.1509	67.5158	54.0235	81.0082
ENSG00000164543	ENSG00000164543	STK17A	7:4362235	q1	q2	0.573	-3.852	0	0.002	yes	5.32619	4.23804	6.41433	9.44333	7.40335	11.4833
ENSG00000152465	ENSG00000152465	NMT2	10:151373	q1	q2	0.575	-3.804	0	0.003	yes	6.29915	4.87122	7.72709	11.1892	8.95494	13.4235
ENSG00000166337	ENSG00000166337	TAF10	11:662487	q1	q2	0.576	-7.341	0	0	yes	133.459	117.665	149.253	237.448	212.965	261.932
ENSG00000140993	ENSG00000140993	TIGD7	16:331379	q1	q2	0.579	-4.963	0	0	yes	7.41936	6.10247	8.73626	13.2323	11.2322	15.2323
ENSG00000136108	ENSG00000136108	CKAP2	13:530294	q1	q2	0.581	-3.712	0	0.004	yes	24.1247	19.361	28.8884	43.1151	32.6538	53.5764
ENSG00000164808	ENSG00000164808	KIAA0146	8:4817316	q1	q2	0.59	-7.523	0	0	yes	92.1696	81.7585	102.581	166.244	148.163	184.324
ENSG00000171121	ENSG00000171121	KCNMB3	3:1788659	q1	q2	0.592	-3.999	0	0.001	yes	6.21588	4.71517	7.7166	11.24	9.31074	13.1694

ENSG00000242515	ENSG00000242515	UGT1A10	2:2345262	q1	q2	0.592	-8.51	0	0	yes	0.0009914	0.0008966	0.00108626	0.0017928	0.0016114	0.0019741
ENSG00000250264	ENSG00000250264	XXbac-BPG246D1	6:3278053	q1	q2	0.593	-5.368	0	0	yes	8.75302	7.29237	10.2137	15.8414	13.5455	18.1374
ENSG00000103056	ENSG00000103056	SMPD3	16:683922	q1	q2	0.595	-4.048	0	0.001	yes	1.64115	1.27475	2.00755	2.97442	2.40636	3.54247
ENSG00000112695	ENSG00000112695	COX7A2	6:7594739	q1	q2	0.598	-5.747	0	0	yes	148.55	131.156	165.944	270.202	223.692	316.713
ENSG00000127423	ENSG00000127423	C1orf135	1:2612666	q1	q2	0.606	-4.164	0	0.001	yes	5.52374	4.29344	6.75403	10.1205	8.22739	12.0137
ENSG00000126777	ENSG00000126777	KTN1	14:559659	q1	q2	0.608	-9.71	0	0	yes	142.524	129.532	155.516	261.667	239.234	284.099
ENSG00000086189	ENSG00000086189	DIMT1L	5:6160198	q1	q2	0.61	-6.375	0	0	yes	25.4009	21.7035	29.0984	46.7367	40.9366	52.5368
ENSG00000227323	ENSG00000227323	RP11-443A13.3	10:786373	q1	q2	0.61	-6.101	0	0	yes	0.0424528	0.0365082	0.0483974	0.0781508	0.0669864	0.0893152
ENSG00000147439	ENSG00000147439	BIN3	8:2246214	q1	q2	0.611	-4.199	0	0.001	yes	21.3315	16.2689	26.3941	39.3035	32.6791	45.928
ENSG00000079337	ENSG00000079337	RAPGEF3	12:480998	q1	q2	0.614	-5.823	0	0	yes	14.5881	11.9521	17.224	26.9599	24.0257	29.8941
ENSG00000257076	ENSG00000257076	RP11-546D6.2	12:123636	q1	q2	0.614	-4.313	0	0	yes	20.6937	16.0245	25.3629	38.2287	31.5976	44.8598
ENSG00000165819	ENSG00000165819	METTL3	14:219271	q1	q2	0.62	-5.235	0	0	yes	16.0878	13.0144	19.1612	29.9044	25.7173	34.0915
ENSG00000205413	ENSG00000205413	SAMD9	7:9272882	q1	q2	0.625	-5.501	0	0	yes	7.73459	6.19169	9.27749	14.4474	12.8775	16.0174
ENSG00000115268	ENSG00000115268	RPS15	19:143836	q1	q2	0.629	-7.479	0	0	yes	188.464	164.762	212.166	353.523	314.027	393.02
ENSG00000204642	ENSG00000204642	HLA-F	6:2969055	q1	q2	0.631	-6.655	0	0	yes	38.6678	33.1913	44.1443	72.6925	63.5193	81.8657
ENSG00000243916	ENSG00000243916	RP11-6K23.1	12:152607	q1	q2	0.632	-6.769	0	0	yes	0.0402162	0.0350565	0.045376	0.0756816	0.0654064	0.0859568
ENSG00000251403	ENSG00000251403	RP11-298J23.6	6:4176210	q1	q2	0.632	-3.599	0	0.006	yes	0.123573	0.0938204	0.153326	0.232588	0.173028	0.292148
ENSG00000138709	ENSG00000138709	LARP1B	4:1289824	q1	q2	0.634	-4.345	0	0	yes	5.53153	4.43143	6.63162	10.4233	8.19994	12.6466
ENSG00000250317	ENSG00000250317	C4orf52	4:2574905	q1	q2	0.636	-4.476	0	0	yes	13.9877	10.8877	17.0877	26.4166	21.7222	31.111
ENSG00000076554	ENSG00000076554	TPD52	8:8083095	q1	q2	0.638	-9.351	0	0	yes	91.1268	82.0318	100.222	172.395	156.377	188.413
ENSG00000143158	ENSG00000143158	BRP44	1:1678859	q1	q2	0.64	-5.501	0	0	yes	23.189	19.0106	27.3674	43.9581	37.4962	50.4199
ENSG00000116213	ENSG00000116213	WRAP73	1:3547330	q1	q2	0.641	-5.006	0	0	yes	14.1196	11.4227	16.8165	26.803	22.2319	31.374
ENSG00000258959	ENSG00000258959	RP11-1017G21.4	14:102430	q1	q2	0.646	-3.505	0	0.008	yes	18.4854	13.1331	23.8377	35.2517	27.2255	43.2779
ENSG00000148444	ENSG00000148444	COMMD3	10:226049	q1	q2	0.647	-4.798	0	0	yes	55.6977	42.2747	69.1207	106.407	93.4888	119.326
ENSG00000137965	ENSG00000137965	IFI44	1:7911548	q1	q2	0.654	-3.799	0	0.003	yes	19.1637	14.9745	23.3529	36.8627	27.0531	46.6724
ENSG00000224281	ENSG00000224281	RP3-404F18.2	X:1185999	q1	q2	0.654	-4.401	0	0	yes	2.96966	2.30362	3.6357	5.71	4.59719	6.82281
ENSG00000125691	ENSG00000125691	RPL23	17:370041	q1	q2	0.665	-6.395	0	0	yes	120.111	104.353	135.869	233.66	195.906	271.414
ENSG00000122482	ENSG00000122482	ZNF644	1:9138085	q1	q2	0.669	-3.59	0	0.006	yes	15.9959	10.5787	21.4132	31.2149	26.3774	36.0525
ENSG00000163870	ENSG00000163870	TPRA1	3:1272919	q1	q2	0.669	-8.717	0	0	yes	28.7864	25.1921	32.3808	56.1832	51.1736	61.1928
ENSG00000159214	ENSG00000159214	CCDC24	1:4445703	q1	q2	0.678	-6.183	0	0	yes	22.9872	18.7158	27.2587	45.2686	40.0058	50.5314
ENSG00000102572	ENSG00000102572	STK24	13:991024	q1	q2	0.68	-6.193	0	0	yes	22.0067	19.2473	24.7662	43.4462	35.6112	51.2812
ENSG00000248341	ENSG00000248341	AC115115.2	2:1130331	q1	q2	0.682	-7.487	0	0	yes	0.164758	0.143876	0.18564	0.325995	0.283282	0.368709
ENSG00000109689	ENSG00000109689	STIM2	4:2685929	q1	q2	0.686	-6.491	0	0	yes	7.11159	6.1931	8.03008	14.1282	11.762	16.4944
ENSG00000149564	ENSG00000149564	ESAM	11:124609	q1	q2	0.687	-5.068	0	0	yes	5.19324	4.08903	6.29745	10.3223	8.58596	12.0586
ENSG00000137821	ENSG00000137821	LRRC49	15:711238	q1	q2	0.688	-5.3	0	0	yes	3.91987	3.12335	4.71639	7.79652	6.53823	9.0548
ENSG00000168890	ENSG00000168890	TMEM150A	2:8582567	q1	q2	0.691	-6.869	0	0	yes	29.1952	24.6603	33.7302	58.2904	50.824	65.7569
ENSG00000218739	ENSG00000218739	AC007390.5	2:3739496	q1	q2	0.692	-3.757	0	0.003	yes	4.36053	3.09094	5.63013	8.70675	6.74622	10.6673
ENSG00000095794	ENSG00000095794	CREM	10:353869	q1	q2	0.694	-5.737	0	0	yes	29.9617	24.6107	35.3127	59.9975	50.1915	69.8035
ENSG00000145730	ENSG00000145730	PAM	5:1020896	q1	q2	0.695	-4.803	0	0	yes	22.4846	16.8859	28.0833	45.0437	38.4052	51.6822
ENSG00000257534	ENSG00000257534	RP11-834C11.10	12:545558	q1	q2	0.702	-8.706	0	0	yes	0.0820847	0.0729501	0.0912193	0.165593	0.146278	0.184908
ENSG00000153044	ENSG00000153044	CENPH	5:6848537	q1	q2	0.707	-3.488	0	0.009	yes	17.0121	12.3687	21.6556	34.4821	24.1621	44.8021
ENSG00000184381	ENSG00000184381	PLA2G6	22:385075	q1	q2	0.709	-4.155	0	0.001	yes	24.8214	17.2961	32.3467	50.4202	42.54	58.3003
ENSG00000116750	ENSG00000116750	UCHL5	1:1929848	q1	q2	0.712	-6.666	0	0	yes	16.9344	13.9135	19.9554	34.512	30.4575	38.5665
ENSG00000089127	ENSG00000089127	OAS1	12:113344	q1	q2	0.713	-8.461	0	0	yes	150.99	131.98	170	308.168	273.593	342.742
ENSG00000151150	ENSG00000151150	ANK3	10:617881	q1	q2	0.718	-5.86	0	0	yes	33.9704	27.581	40.3598	69.6773	58.7171	80.6374
ENSG00000170191	ENSG00000170191	NANP	20:255935	q1	q2	0.725	-4.574	0	0	yes	1.71362	1.29319	2.13404	3.53806	2.82774	4.24839
ENSG00000198056	ENSG00000198056	PRIM1	12:571062	q1	q2	0.725	-4.225	0	0.001	yes	15.0689	11.0433	19.0944	31.1083	24.4082	37.8084
ENSG00000129472	ENSG00000129472	RAB2B	14:219271	q1	q2	0.729	-3.881	0	0.002	yes	8.99395	6.30445	11.6834	18.649	14.4052	22.8927
ENSG00000184508	ENSG00000184508	HDDC3	15:914717	q1	q2	0.732	-6.929	0	0	yes	21.1956	17.6736	24.7176	44.052	38.3123	49.7917
ENSG00000108094	ENSG00000108094	CUL2	10:352974	q1	q2	0.735	-4.698	0	0	yes	9.93479	8.56752	11.302	20.7185	14.896	26.5411

ENSG00000213397	ENSG00000213397	HAUS7	X:1527101	q1	q2	0.737	-4.379	0	0	yes	37.1909	30.6633	43.7185	77.7378	55.3954	100.08
ENSG00000258168	ENSG00000258168	RP11-588H23.3	12:708618	q1	q2	0.746	-7.982	0	0	yes	0.113343	0.0992027	0.127484	0.238872	0.205666	0.272078
ENSG00000127720	ENSG00000127720	C12orf26	12:826174	q1	q2	0.747	-4.986	0	0	yes	16.6681	12.657	20.6793	35.1879	28.9021	41.4737
ENSG00000028116	ENSG00000028116	VRK2	2:5813478	q1	q2	0.749	-4.733	0	0	yes	6.68694	4.95365	8.42023	14.1446	11.5732	16.7159
ENSG00000012822	ENSG00000012822	CALCOCO1	12:541049	q1	q2	0.751	-5.094	0	0	yes	29.3097	21.5591	37.0604	62.0958	54.0131	70.1784
ENSG00000244663	ENSG00000244663	AC007599.1	16:474952	q1	q2	0.753	-9.242	0	0	yes	145.72	127.806	163.634	309.433	276.33	342.537
ENSG00000129696	ENSG00000129696	C8orf41	8:3322834	q1	q2	0.754	-5.208	0	0	yes	13.328	10.2652	16.3908	28.3297	23.3381	33.3214
ENSG00000138768	ENSG00000138768	USO1	4:7664976	q1	q2	0.761	-4.765	0	0	yes	19.8764	17.2183	22.5344	42.5258	30.1985	54.8532
ENSG00000167081	ENSG00000167081	PBX3	9:1285085	q1	q2	0.761	-7.233	0	0	yes	36.3758	30.4505	42.301	77.8935	67.5009	88.286
ENSG00000129515	ENSG00000129515	SNX6	14:350259	q1	q2	0.763	-6.058	0	0	yes	15.8629	13.2198	18.5059	34.0256	27.5955	40.4558
ENSG00000178878	ENSG00000178878	APOLD1	12:128788	q1	q2	0.764	-6.811	0	0	yes	2.95584	2.43677	3.4749	6.34384	5.45891	7.22876
ENSG00000107771	ENSG00000107771	FAM190B	10:860883	q1	q2	0.766	-4.42	0	0	yes	20.3912	14.0758	26.7066	43.8547	37.0381	50.6713
ENSG00000255729	ENSG00000255729	AC005618.1	5:1406980	q1	q2	0.766	-4.058	0	0.001	yes	11.675	8.15604	15.194	25.1255	19.4042	30.8467
ENSG00000164163	ENSG00000164163	ABCE1	4:1458882	q1	q2	0.772	-4.256	0	0	yes	7.13842	5.09101	9.18583	15.4545	12.0159	18.8931
ENSG00000147586	ENSG00000147586	MRPS28	8:8083095	q1	q2	0.773	-4.462	0	0	yes	19.7044	14.1797	25.2291	42.6965	33.9985	51.3945
ENSG00000052841	ENSG00000052841	TTC17	11:433335	q1	q2	0.778	-6.027	0	0	yes	19.1489	15.4218	22.8759	41.6886	34.6181	48.7591
ENSG00000159199	ENSG00000159199	ATP5G1	17:469522	q1	q2	0.778	-4.205	0	0.001	yes	17.6581	12.2147	23.1015	38.4314	30.5775	46.2852
ENSG00000111845	ENSG00000111845	PAK1IP1	6:1067165	q1	q2	0.789	-4.079	0	0.001	yes	2.47288	1.7074	3.23836	5.44243	4.18052	6.70435
ENSG00000162174	ENSG00000162174	ASRGL1	11:621043	q1	q2	0.791	-5.534	0	0	yes	17.3332	13.5643	21.1021	38.2451	31.1393	45.3509
ENSG00000134326	ENSG00000134326	CMPK2	2:6980700	q1	q2	0.794	-8.621	0	0	yes	44.4982	37.9942	51.0022	98.4091	87.3887	109.43
ENSG00000174483	ENSG00000174483	BBS1	11:662342	q1	q2	0.794	-5.126	0	0	yes	11.1253	8.23966	14.011	24.6118	20.442	28.7816
ENSG00000110536	ENSG00000110536	PTPMT1	11:474874	q1	q2	0.795	-8.917	0	0	yes	52.6761	45.4708	59.8813	116.688	103.326	130.049
ENSG00000071794	ENSG00000071794	HLTF	3:1487479	q1	q2	0.798	-6.104	0	0	yes	25.0488	20.9836	29.114	55.6575	44.2397	67.0754
ENSG00000255857	ENSG00000255857	RP1-278C19.3	12:120639	q1	q2	0.8	-6.357	0	0	yes	14.9677	12.0054	17.9301	33.2979	28.1264	38.4693
ENSG00000144554	ENSG00000144554	FANCD2	3:1006809	q1	q2	0.803	-5.127	0	0	yes	17.051	12.7758	21.3262	38.0756	30.9168	45.2344
ENSG00000255909	ENSG00000255909	RP11-841C19.3	12:195569	q1	q2	0.806	-11.834	0	0	yes	0.249049	0.227555	0.270544	0.557656	0.498874	0.616438
ENSG00000107201	ENSG00000107201	DDX58	9:3245529	q1	q2	0.807	-5.785	0	0	yes	18.871	15.831	21.911	42.2729	32.6532	51.8925
ENSG00000120306	ENSG00000120306	C5orf32	5:1395542	q1	q2	0.807	-4.816	0	0	yes	11.6899	8.50539	14.8745	26.1901	21.0834	31.2968
ENSG00000100142	ENSG00000100142	POLR2F	22:383395	q1	q2	0.809	-7.567	0	0	yes	69.0902	59.2197	78.9607	155.174	130.478	179.869
ENSG00000012048	ENSG00000012048	BRCA1	17:411963	q1	q2	0.81	-6.89	0	0	yes	7.80394	6.68617	8.92171	17.5422	14.2713	20.8131
ENSG00000135114	ENSG00000135114	OASL	12:121458	q1	q2	0.81	-6.322	0	0	yes	12.9576	10.2448	15.6704	29.1234	24.8225	33.4242
ENSG00000157734	ENSG00000157734	SNX22	15:644439	q1	q2	0.812	-5.904	0	0	yes	2.27315	1.77257	2.77373	5.12015	4.27609	5.9642
ENSG00000116791	ENSG00000116791	CRYZ	1:7501808	q1	q2	0.822	-7.474	0	0	yes	17.0372	13.8403	20.2342	38.7693	34.3158	43.2228
ENSG00000160957	ENSG00000160957	RECQL4	8:1457366	q1	q2	0.825	-12.962	0	0	yes	106.007	95.9861	116.027	242.005	221.349	262.661
ENSG00000166529	ENSG00000166529	ZSCAN21	7:9964738	q1	q2	0.826	-6.879	0	0	yes	9.44368	7.52955	11.3578	21.5793	18.7956	24.3629
ENSG00000167553	ENSG00000167553	TUBA1C	12:495785	q1	q2	0.827	-14.373	0	0	yes	111.69	102.125	121.255	255.306	235.694	274.917
ENSG00000112096	ENSG00000112096	SOD2	6:1601000	q1	q2	0.83	-11.401	0	0	yes	71.6372	63.8029	79.4714	164.366	148.546	180.186
ENSG00000100934	ENSG00000100934	SEC23A	14:395011	q1	q2	0.831	-9.035	0	0	yes	71.9846	61.0015	82.9678	165.215	148.249	182.18
ENSG00000129158	ENSG00000129158	SERGEF	11:178095	q1	q2	0.831	-6.276	0	0	yes	37.7388	31.2306	44.2471	86.6295	69.2207	104.038
ENSG00000215217	ENSG00000215217	C5orf49	5:7830490	q1	q2	0.834	-7.681	0	0	yes	0.0500651	0.0427394	0.0573908	0.11522	0.0967503	0.133689
ENSG00000228253	ENSG00000228253	J01415.25	MT:8365-9	q1	q2	0.836	-7.32	0	0	yes	536.127	440.886	631.368	1236.72	1059.24	1414.2
ENSG00000138111	ENSG00000138111	TMEM180	10:104221	q1	q2	0.839	-3.623	0	0.005	yes	2.03722	1.27054	2.80391	4.71273	3.44226	5.9832
ENSG00000167996	ENSG00000167996	FTH1	11:617172	q1	q2	0.845	-13.411	0	0	yes	247.937	221.381	274.493	577.1	538.81	615.391
ENSG00000164465	ENSG00000164465	DCBLD1	6:1176094	q1	q2	0.847	-4.762	0	0	yes	10.9167	7.95201	13.8813	25.4522	19.6111	31.2934
ENSG00000065328	ENSG00000065328	MCM10	10:132035	q1	q2	0.848	-5.489	0	0	yes	6.9251	5.75661	8.09359	16.1653	11.9827	20.3479
ENSG00000178623	ENSG00000178623	GPR35	2:2415261	q1	q2	0.852	-3.912	0	0.002	yes	2.14812	1.36561	2.93063	5.03663	3.83331	6.23995
ENSG00000187109	ENSG00000187109	NAP1L1	12:764386	q1	q2	0.867	-5.268	0	0	yes	32.0684	25.5616	38.5752	76.2997	56.5323	96.0671
ENSG00000258974	ENSG00000258974	AC002086.1	X:1193846	q1	q2	0.872	-4.831	0	0	yes	2.0961	1.48766	2.70453	5.01475	3.93745	6.09206
ENSG00000157985	ENSG00000157985	AGAP1	2:2364027	q1	q2	0.887	-9.926	0	0	yes	25.1248	21.8658	28.3838	61.0236	53.5139	68.5334
ENSG00000119640	ENSG00000119640	ACYP1	14:755199	q1	q2	0.894	-4.148	0	0.001	yes	7.65149	4.97151	10.3315	18.7146	14.0048	23.4244

ENSG00000155636	ENSG00000155636	RBM45	2:1789771	q1	q2	0.894	-3.734	0	0.004	yes	8.99241	5.15768	12.8271	21.9956	17.1909	26.8003
ENSG00000121753	ENSG00000121753	BAI2	1:3219271	q1	q2	0.9	-7.237	0	0	yes	11.0448	8.71151	13.378	27.1707	23.6007	30.7407
ENSG00000256566	ENSG00000256566	RP4-734P14.4	20:244227	q1	q2	0.904	-3.607	0	0.006	yes	3.51235	2.04891	4.97579	8.67496	6.25525	11.0947
ENSG00000166548	ENSG00000166548	TK2	16:665419	q1	q2	0.905	-4.166	0	0.001	yes	2.32987	1.83512	2.82461	5.7611	3.57616	7.94605
ENSG00000126803	ENSG00000126803	HSPA2	14:648547	q1	q2	0.906	-5.879	0	0	yes	12.9913	9.5313	16.4514	32.1379	27.1564	37.1193
ENSG00000185201	ENSG00000185201	IFITM2	11:307630	q1	q2	0.906	-9.56	0	0	yes	59.2737	50.0212	68.5262	146.696	130.915	162.476
ENSG00000175066	ENSG00000175066	GK5	3:1418824	q1	q2	0.917	-4.093	0	0.001	yes	7.63439	5.1189	10.1499	19.0928	13.3007	24.8849
ENSG00000157851	ENSG00000157851	DPYSL5	2:2707061	q1	q2	0.919	-6.757	0	0	yes	16.0265	12.4938	19.5592	40.1775	33.7714	46.5836
ENSG00000173457	ENSG00000173457	PPP1R14B	11:640119	q1	q2	0.93	-3.966	0	0.002	yes	5.21119	3.1778	7.24459	13.2123	9.77079	16.6537
ENSG00000169991	ENSG00000169991	IFFO2	1:1916609	q1	q2	0.931	-5.454	0	0	yes	5.94164	4.1593	7.72399	15.0799	12.6184	17.5414
ENSG00000213689	ENSG00000213689	TREX1	3:4848811	q1	q2	0.932	-6.705	0	0	yes	7.89936	5.92255	9.87617	20.07	17.6339	22.5061
ENSG00000198417	ENSG00000198417	MT1F	16:566918	q1	q2	0.936	-3.918	0	0.002	yes	16.9963	10.768	23.2245	43.3171	30.0524	56.5818
ENSG00000204438	ENSG00000204438	GPANK1	6:3162900	q1	q2	0.939	-9.624	0	0	yes	18.5908	15.4874	21.6943	47.5673	42.7506	52.3839
ENSG00000183531	ENSG00000183531	Z98256.1	22:329085	q1	q2	0.94	-3.869	0	0.002	yes	10.7821	6.40425	15.1599	27.6033	20.2337	34.9729
ENSG00000214283	ENSG00000214283	RP11-85F14.1	3:1365810	q1	q2	0.943	-9.206	0	0	yes	0.0178308	0.015334	0.0203276	0.0457738	0.0389312	0.0526164
ENSG00000135476	ENSG00000135476	ESPL1	12:536620	q1	q2	0.948	-9.375	0	0	yes	20.5529	17.8429	23.2629	53.0308	44.9009	61.1607
ENSG00000138385	ENSG00000138385	SSB	2:1706484	q1	q2	0.955	-7.915	0	0	yes	21.5039	17.2863	25.7216	55.8726	48.0225	63.7227
ENSG00000091039	ENSG00000091039	OSBPL8	12:767455	q1	q2	0.964	-6.565	0	0	yes	6.28457	5.46176	7.10738	16.4812	12.1477	20.8148
ENSG00000185745	ENSG00000185745	IFIT1	10:909733	q1	q2	0.964	-9.973	0	0	yes	17.7034	15.0296	20.3771	46.419	40.8172	52.0208
ENSG00000107371	ENSG00000107371	EXOSC3	9:3751088	q1	q2	0.965	-5.073	0	0	yes	21.0443	16.8946	25.194	55.2101	37.2596	73.1607
ENSG00000204314	ENSG00000204314	PRRT1	6:3211613	q1	q2	0.97	-10.08	0	0	yes	32.8292	27.8734	37.785	86.5642	76.2403	96.8881
ENSG00000224189	ENSG00000224189	AC009336.23	2:1769863	q1	q2	0.986	-5.438	0	0	yes	9.79845	6.88016	12.7167	26.267	20.8303	31.7037
ENSG00000251314	ENSG00000251314	CTD-2337A12.1	5:9522080	q1	q2	0.988	-25.541	0	0	yes	0.0481994	0.0455988	0.0508	0.129486	0.122303	0.136668
ENSG00000114473	ENSG00000114473	IQCG	3:1976159	q1	q2	0.993	-4.314	0	0	yes	4.91656	2.98014	6.85298	13.2773	10.1067	16.4479
ENSG00000054267	ENSG00000054267	ARID4B	1:2352944	q1	q2	0.994	-7.11	0	0	yes	12.9326	11.5959	14.2693	34.94	25.8637	44.0163
ENSG00000134321	ENSG00000134321	RSAD2	2:6980700	q1	q2	1.002	-8.312	0	0	yes	16.9812	13.5275	20.4348	46.2669	40.2688	52.265
ENSG00000119917	ENSG00000119917	IFIT3	10:909733	q1	q2	1.005	-9.031	0	0	yes	6.42996	5.27673	7.5832	17.5704	15.253	19.8878
ENSG00000136143	ENSG00000136143	SUCLA2	13:485106	q1	q2	1.008	-5.458	0	0	yes	7.01922	5.03448	9.00397	19.2411	14.6647	23.8175
ENSG00000114544	ENSG00000114544	SLC41A3	3:1257251	q1	q2	1.01	-10.551	0	0	yes	39.997	34.8023	45.1916	109.774	94.3429	125.205
ENSG00000223725	ENSG00000223725	AC007879.5	2:2081043	q1	q2	1.014	-17.121	0	0	yes	0.0739635	0.0677067	0.0802202	0.203789	0.186909	0.22067
ENSG00000083097	ENSG00000083097	DOPEY1	6:8377738	q1	q2	1.027	-5.484	0	0	yes	2.50174	1.61112	3.39237	6.9837	6.17474	7.79266
ENSG00000225920	ENSG00000225920	RIMKLBP2	1:2192599	q1	q2	1.032	-6.688	0	0	yes	0.0679035	0.0518985	0.0839085	0.190506	0.152594	0.228418
ENSG00000119509	ENSG00000119509	INVS	9:1028615	q1	q2	1.036	-5.164	0	0	yes	9.36207	5.9684	12.7557	26.37	21.8434	30.8966
ENSG00000202394	ENSG00000202394	SNORD47	1:1738312	q1	q2	1.038	-9.466	0	0	yes	29.417	24.7802	34.0537	83.0666	70.3991	95.7342
ENSG00000143353	ENSG00000143353	LYPLAL1	1:2192599	q1	q2	1.04	-5.328	0	0	yes	5.35593	3.79886	6.913	15.1583	11.2063	19.1104
ENSG00000158092	ENSG00000158092	NCK1	3:1365810	q1	q2	1.04	-4.03	0	0.001	yes	4.37678	2.28706	6.46651	12.3826	9.95731	14.8079
ENSG00000010030	ENSG00000010030	ETV7	6:3632241	q1	q2	1.053	-6.949	0	0	yes	4.2522	3.16885	5.33555	12.183	10.186	14.1801
ENSG00000173113	ENSG00000173113	TRMT112	11:640730	q1	q2	1.054	-9.195	0	0	yes	22.6609	18.2987	27.0231	65.0087	56.9176	73.0999
ENSG00000207556	ENSG00000207556	MIR636	17:747302	q1	q2	1.055	-42.934	0	0	yes	4.42696	4.27876	4.57517	12.7107	12.2536	13.1678
ENSG00000151006	ENSG00000151006	PRSS53	16:310857	q1	q2	1.06	-6.942	0	0	yes	4.31615	3.19323	5.43906	12.4572	10.4656	14.4488
ENSG00000227617	ENSG00000227617	AC009475.2	2:1693127	q1	q2	1.062	-5.525	0	0	yes	1.38579	0.943961	1.82762	4.00739	3.14701	4.86776
ENSG00000155744	ENSG00000155744	FAM126B	2:2017736	q1	q2	1.065	-6.226	0	0	yes	6.56554	4.88686	8.24423	19.0404	14.7141	23.3667
ENSG00000119616	ENSG00000119616	FCF1	14:751798	q1	q2	1.066	-5.003	0	0	yes	7.4291	4.73469	10.1235	21.5773	16.7465	26.4081
ENSG00000003509	ENSG00000003509	C2orf56	2:3739496	q1	q2	1.079	-9.05	0	0	yes	23.2746	18.547	28.0023	68.4453	59.9042	76.9864
ENSG00000228932	ENSG00000228932	RP11-88G17.2	9:1328157	q1	q2	1.079	-34.733	0	0	yes	0.0763171	0.0729997	0.0796344	0.224405	0.214451	0.234358
ENSG00000246982	ENSG00000246982	RP1-179N16.6	6:3611447	q1	q2	1.08	-32.368	0	0	yes	0.0029454	0.0028212	0.00306955	0.0086705	0.0082222	0.0091189
ENSG00000114388	ENSG00000114388	NPRL2	3:5038476	q1	q2	1.081	-9.051	0	0	yes	18.4409	14.6373	22.2445	54.3829	47.8193	60.9465
ENSG00000204856	ENSG00000204856	C12orf24	12:110890	q1	q2	1.086	-6.474	0	0	yes	5.56961	3.91807	7.22115	16.501	13.9098	19.0921
ENSG00000171097	ENSG00000171097	CCBL1	9:1315952	q1	q2	1.094	-6.19	0	0	yes	3.26793	2.33709	4.19878	9.75795	7.71598	11.7999
ENSG00000157654	ENSG00000157654	PALM2-AKAP2	9:1124030	q1	q2	1.098	-3.653	0	0.005	yes	0.7497	0.405493	1.09391	2.24787	1.37533	3.12041

ENSG00000129194	ENSG00000129194	SOX15	17:748292	q1	q2	1.103	-4.386	0	0	yes	1.80303	1.04622	2.55983	5.43019	3.92762	6.93275
ENSG00000177981	ENSG00000177981	ASB8	12:485415	q1	q2	1.106	-12.597	0	0	yes	39.415	33.8053	45.0247	119.105	106.86	131.35
ENSG00000088876	ENSG00000088876	ZNF343	20:244227	q1	q2	1.109	-9.974	0	0	yes	4.37142	3.59537	5.14747	13.2514	11.4765	15.0264
ENSG00000249293	ENSG00000249293	CTC-575I10.1	5:7292198	q1	q2	1.109	-19.528	0	0	yes	0.0855451	0.0791778	0.0919124	0.259376	0.237114	0.281637
ENSG00000159899	ENSG00000159899	NPR2	9:3579215	q1	q2	1.115	-3.567	0	0.007	yes	1.84315	0.935158	2.75114	5.62059	3.45761	7.78358
ENSG00000186862	ENSG00000186862	PDZD7	10:102756	q1	q2	1.116	-5.291	0	0	yes	3.09049	1.96542	4.21555	9.43215	7.42264	11.4417
ENSG00000080823	ENSG00000080823	RAGE	14:102692	q1	q2	1.122	-5.704	0	0	yes	8.44346	5.72976	11.1572	25.9296	20.0478	31.8115
ENSG00000249115	ENSG00000249115	HAUS5	19:361036	q1	q2	1.141	-11.053	0	0	yes	5.88639	4.95338	6.81941	18.4154	15.9814	20.8495
ENSG00000144401	ENSG00000144401	METTL21A	2:2081043	q1	q2	1.143	-4.016	0	0.001	yes	3.51839	1.78106	5.25572	11.0316	7.91006	14.1531
ENSG00000119922	ENSG00000119922	IFIT2	10:909733	q1	q2	1.175	-7.934	0	0	yes	2.06626	1.56792	2.5646	6.68999	5.54027	7.83971
ENSG00000256349	ENSG00000256349	CTD-3074O7.11	11:662342	q1	q2	1.176	-8.127	0	0	yes	2.18126	1.66736	2.69517	7.0714	5.88254	8.26026
ENSG00000105640	ENSG00000105640	RPL18A	19:179707	q1	q2	1.186	-11.363	0	0	yes	51.7198	43.0698	60.3699	169.353	148.188	190.517
ENSG00000122406	ENSG00000122406	RPL5	1:9329759	q1	q2	1.189	-3.756	0	0.003	yes	8.11115	3.5565	12.6658	26.6354	18.8474	34.4234
ENSG00000118894	ENSG00000118894	FAM86A	16:512181	q1	q2	1.19	-5.214	0	0	yes	1.13745	0.688554	1.58635	3.73851	2.88127	4.59575
ENSG00000254876	ENSG00000254876	RP11-23J9.5	9:1000000	q1	q2	1.192	-8.119	0	0	yes	0.025137	0.0200842	0.0301897	0.0827885	0.065068	0.100509
ENSG00000167100	ENSG00000167100	SAMD14	17:481721	q1	q2	1.201	-3.987	0	0.001	yes	0.633679	0.395312	0.872046	2.10542	1.11499	3.09585
ENSG00000254772	ENSG00000254772	EEF1G	11:623270	q1	q2	1.206	-10.669	0	0	yes	13.2579	10.9108	15.605	44.2835	38.0565	50.5104
ENSG00000249007	ENSG00000249007	RP11-510N19.5	1:2019514	q1	q2	1.209	-3.944	0	0.002	yes	1.70697	0.799568	2.61437	5.71898	3.97155	7.46641
ENSG00000189337	ENSG00000189337	KAZN	1:1492519	q1	q2	1.22	-5.341	0	0	yes	2.12773	1.31848	2.93697	7.20561	5.38275	9.02848
ENSG00000244513	ENSG00000244513	CTD-2013N24.2	3:6906327	q1	q2	1.223	-13.418	0	0	yes	0.0553102	0.0487691	0.0618513	0.187868	0.161815	0.213921
ENSG00000095574	ENSG00000095574	IKZF5	10:124690	q1	q2	1.225	-3.78	0	0.003	yes	2.90671	1.62243	4.19099	9.89225	5.20308	14.5814
ENSG00000255152	ENSG00000255152	MSH5-C6orf26	6:3170772	q1	q2	1.234	-5.643	0	0	yes	1.75903	1.1856	2.33245	6.04201	4.28016	7.80387
ENSG00000203279	ENSG00000203279	RP11-498P14.5	9:1000000	q1	q2	1.24	-8.744	0	0	yes	0.0380334	0.0303884	0.0456784	0.131387	0.105109	0.157664
ENSG00000156970	ENSG00000156970	BUB1B	15:404532	q1	q2	1.243	-11.432	0	0	yes	3.43313	2.8078	4.05847	11.9036	10.4884	13.3189
ENSG00000118263	ENSG00000118263	KLF7	2:2079388	q1	q2	1.259	-3.827	0	0.003	yes	5.24757	2.13092	8.36422	18.4892	13.2415	23.7369
ENSG00000154309	ENSG00000154309	DISP1	1:2229883	q1	q2	1.265	-4.668	0	0	yes	1.24465	0.71134	1.77795	4.41136	2.94646	5.87626
ENSG00000174444	ENSG00000174444	RPL4	15:667908	q1	q2	1.282	-10.208	0	0	yes	17.8381	14.1494	21.5268	64.3162	55.1346	73.4977
ENSG00000166348	ENSG00000166348	USP54	10:751961	q1	q2	1.286	-8.274	0	0	yes	9.51352	7.0577	11.9693	34.4077	28.4553	40.3601
ENSG00000121236	ENSG00000121236	TRIM6	11:527441	q1	q2	1.305	-4.224	0	0.001	yes	0.635799	0.342391	0.929208	2.34359	1.38159	3.30558
ENSG00000243135	ENSG00000243135	UGT1A3	2:2345262	q1	q2	1.307	-6.464	0	0	yes	1.46264	0.956513	1.96878	5.40433	4.27369	6.53498
ENSG00000160062	ENSG00000160062	ZBTB8A	1:3293065	q1	q2	1.31	-3.714	0	0.004	yes	0.573468	0.218483	0.928452	2.1248	1.40649	2.84311
ENSG00000256223	ENSG00000256223	ZNF10	12:133707	q1	q2	1.314	-4.559	0	0	yes	0.254865	0.135511	0.374219	0.948427	0.629537	1.26732
ENSG00000183077	ENSG00000183077	AFMID	17:761833	q1	q2	1.337	-8.612	0	0	yes	6.46081	5.00849	7.91314	24.6074	19.3344	29.8803
ENSG00000242257	ENSG00000242257	AC044839.1	11:458481	q1	q2	1.341	-3.917	0	0.002	yes	107.414	42.6409	172.187	410.733	277.473	543.992
ENSG00000144802	ENSG00000144802	NFKBIZ	3:1014980	q1	q2	1.346	-8.529	0	0	yes	5.42436	4.31009	6.53863	20.846	15.8494	25.8426
ENSG00000256142	ENSG00000256142	AL513327.1	1:3372214	q1	q2	1.347	-4.049	0	0.001	yes	0.962019	0.397731	1.52631	3.70155	2.53701	4.86609
ENSG00000147687	ENSG00000147687	TATDN1	8:1255007	q1	q2	1.35	-8.795	0	0	yes	10.8226	7.93491	13.7104	41.7491	35.4086	48.0897
ENSG00000105519	ENSG00000105519	CAPS	19:591138	q1	q2	1.352	-9.455	0	0	yes	4.04274	3.05306	5.03243	15.6181	13.3123	17.9239
ENSG00000100629	ENSG00000100629	CEP128	14:809433	q1	q2	1.357	-5.126	0	0	yes	3.83187	2.05679	5.60696	14.8778	11.0701	18.6855
ENSG00000138442	ENSG00000138442	WDR12	2:2037453	q1	q2	1.364	-7.628	0	0	yes	10.7902	8.00839	13.572	42.1896	31.7397	52.6396
ENSG00000169062	ENSG00000169062	UPF3A	13:115047	q1	q2	1.364	-4.033	0	0.001	yes	3.98767	1.95202	6.02333	15.5932	8.67727	22.5091
ENSG00000226763	ENSG00000226763	SRRM5	19:441005	q1	q2	1.365	-6.739	0	0	yes	1.20748	0.779482	1.63547	4.72629	3.80038	5.6522
ENSG00000164048	ENSG00000164048	ZNF589	3:4828258	q1	q2	1.371	-7.243	0	0	yes	1.4373	1.03256	1.84203	5.65917	4.22821	7.09014
ENSG00000242095	ENSG00000242095	AC062037.1	2:8556921	q1	q2	1.373	-31.29	0	0	yes	0.151867	0.142718	0.161017	0.599304	0.561074	0.637534
ENSG00000226099	ENSG00000226099	RP11-544L8__B.2	6:1142541	q1	q2	1.416	-39.999	0	0	yes	0.152785	0.14538	0.160189	0.629326	0.59686	0.661792
ENSG00000073910	ENSG00000073910	FRY	13:325994	q1	q2	1.432	-3.756	0	0.003	yes	1.21569	0.518242	1.91313	5.08765	2.53354	7.64176
ENSG00000119684	ENSG00000119684	MLH3	14:754804	q1	q2	1.443	-4.378	0	0	yes	7.10995	2.53915	11.6808	30.0914	25.7162	34.4665
ENSG00000234141	ENSG00000234141	AC009473.1	7:7676148	q1	q2	1.452	-27.733	0	0	yes	0.0456347	0.0422497	0.0490197	0.194955	0.180544	0.209366
ENSG00000226677	ENSG00000226677	RP11-85K15.1	14:353900	q1	q2	1.453	-35.472	0	0	yes	0.0560201	0.0528384	0.0592019	0.23942	0.225294	0.253547
ENSG00000255782	ENSG00000255782	Z83840.1	22:419567	q1	q2	1.47	-5.341	0	0	yes	0.948394	0.482716	1.41407	4.12498	3.09828	5.15168

ENSG00000182004	ENSG00000182004	SNRPE	1:2038307	q1	q2	1.474	-4.081	0	0.001	yes	13.2173	4.58458	21.85	57.7134	39.8985	75.5284
ENSG00000114670	ENSG00000114670	NEK11	3:1305694	q1	q2	1.481	-5.84	0	0	yes	2.89387	1.48122	4.30651	12.7272	10.9735	14.4809
ENSG00000179988	ENSG00000179988	PSTK	10:124690	q1	q2	1.484	-6.446	0	0	yes	2.40018	1.50954	3.29082	10.5886	7.70055	13.4766
ENSG00000225721	ENSG00000225721	RP11-269F19.2	1:4520548	q1	q2	1.501	-4.09	0	0.001	yes	2.46015	0.836469	4.08383	11.0388	7.49205	14.5856
ENSG00000134780	ENSG00000134780	DAGLA	11:614479	q1	q2	1.511	-14.846	0	0	yes	2.69819	2.23181	3.16458	12.2229	10.9097	13.5362
ENSG00000164306	ENSG00000164306	CCDC111	4:1855707	q1	q2	1.521	-5.466	0	0	yes	3.19395	1.52131	4.86659	14.6202	11.8661	17.3743
ENSG00000125966	ENSG00000125966	MMP24	20:337031	q1	q2	1.544	-7.513	0	0	yes	0.699035	0.440888	0.957181	3.27415	2.68302	3.86529
ENSG00000234134	ENSG00000234134	RP11-383C5.5	10:127371	q1	q2	1.558	-18.915	0	0	yes	0.197029	0.173687	0.220371	0.936042	0.828847	1.04324
ENSG00000105793	ENSG00000105793	GTPBP10	7:8996453	q1	q2	1.565	-11.032	0	0	yes	13.6827	10.1716	17.1938	65.4398	57.5209	73.3587
ENSG00000220378	ENSG00000220378	KRT8P42	6:1344903	q1	q2	1.568	-6.16	0	0	yes	0.012738	0.008492	0.016984	0.0611179	0.0375936	0.0846422
ENSG00000186615	ENSG00000186615	C14orf33	14:559659	q1	q2	1.57	-4.222	0	0.001	yes	1.37975	0.403104	2.3564	6.63106	5.11921	8.1429
ENSG00000066651	ENSG00000066651	TRMT11	6:1263075	q1	q2	1.582	-3.809	0	0.003	yes	1.29003	0.568527	2.01154	6.27442	2.42219	10.1267
ENSG00000213145	ENSG00000213145	CRIP1	14:105952	q1	q2	1.611	-7.881	0	0	yes	3.39633	2.12748	4.66517	17.0118	14.1839	19.8397
ENSG00000256390	ENSG00000256390	AC092143.2	16:899785	q1	q2	1.623	-6.014	0	0	yes	0.865724	0.445119	1.28633	4.38733	3.3559	5.41876
ENSG00000142794	ENSG00000142794	NBPF3	1:2176662	q1	q2	1.631	-5.902	0	0	yes	2.23188	1.40071	3.06305	11.397	6.74577	16.0482
ENSG00000136367	ENSG00000136367	ZFHX2	14:239809	q1	q2	1.651	-4.515	0	0	yes	0.735475	0.276591	1.19436	3.83244	2.37144	5.29343
ENSG00000111291	ENSG00000111291	GPRC5D	12:130806	q1	q2	1.671	-5.289	0	0	yes	3.29375	1.32185	5.26566	17.5101	13.9734	21.0467
ENSG00000250299	ENSG00000250299	RP11-78J21.2	13:531916	q1	q2	1.707	-4.123	0	0.001	yes	0.0037221	0.001368	0.0060761	0.0205182	0.0095508	0.0314857
ENSG00000111249	ENSG00000111249	CUX2	12:111471	q1	q2	1.713	-3.592	0	0.006	yes	0.270794	0.146545	0.395043	1.50103	0.246422	2.75564
ENSG00000184731	ENSG00000184731	FAM110C	2:38813-4	q1	q2	1.728	-5.676	0	0	yes	11.0912	4.56163	17.6209	62.4419	52.7203	72.1635
ENSG00000234741	ENSG00000234741	GAS5	1:1738312	q1	q2	1.73	-7.668	0	0	yes	8.78912	5.82769	11.7505	49.5578	34.6905	64.4251
ENSG00000166839	ENSG00000166839	ANKDD1A	15:651340	q1	q2	1.747	-5.333	0	0	yes	1.15169	0.490665	1.81271	6.60608	4.51959	8.69258
ENSG00000169239	ENSG00000169239	CA5B	X:1569305	q1	q2	1.747	-5.985	0	0	yes	2.33021	1.15874	3.50168	13.3703	9.40163	17.339
ENSG00000111671	ENSG00000111671	SPSB2	12:697628	q1	q2	1.755	-11.217	0	0	yes	5.09374	3.6579	6.52958	29.4681	25.4606	33.4755
ENSG00000232536	ENSG00000232536	RP11-74C1.4	1:1515127	q1	q2	1.775	-45.18	0	0	yes	0.0463243	0.0438248	0.0488237	0.273218	0.257618	0.288818
ENSG00000152056	ENSG00000152056	AP1S3	2:2246164	q1	q2	1.792	-4.886	0	0	yes	2.63897	1.17438	4.10356	15.8304	8.24296	23.4178
ENSG00000232073	ENSG00000232073	RP1-302D9.3	22:335600	q1	q2	1.792	-3.697	0	0.004	yes	0.204091	0.193981	0.214201	1.22482	0.0388741	2.41077
ENSG00000258395	ENSG00000258395	RP11-65I12.1	2:1139690	q1	q2	1.796	-3.559	0	0.007	yes	0.181099	0.165076	0.197122	1.09128	0	2.1884
ENSG00000243902	ENSG00000243902	RP1-63G5.5	22:377356	q1	q2	1.801	-6.327	0	0	yes	4.36843	2.06965	6.66721	26.4539	20.7099	32.1978
ENSG00000164404	ENSG00000164404	GDF9	5:1321968	q1	q2	1.812	-4.845	0	0	yes	0.558831	0.226037	0.891626	3.42103	1.87262	4.96943
ENSG00000226360	ENSG00000226360	RPL10AP6	3:6154724	q1	q2	1.819	-14.868	0	0	yes	19.5068	15.293	23.7205	120.258	106.439	134.077
ENSG00000236830	ENSG00000236830	AP000689.8	21:375026	q1	q2	1.849	-8.136	0	0	yes	3.06831	1.75581	4.38081	19.4865	16.4969	22.4761
ENSG00000254909	ENSG00000254909	RP11-110I1.5	11:118868	q1	q2	1.85	-3.704	0	0.004	yes	1.95934	1.8772	2.04147	12.4598	0.0256351	24.894
ENSG00000225374	ENSG00000225374	GS1-526D21.5	X:1375283	q1	q2	1.854	-12.866	0	0	yes	0.0511991	0.0414799	0.0609183	0.327023	0.256082	0.397965
ENSG00000228604	ENSG00000228604	RP5-872K7.7	20:211066	q1	q2	1.87	-10.281	0	0	yes	0.0331158	0.0245654	0.0416663	0.214808	0.159778	0.269839
ENSG00000175344	ENSG00000175344	CHRNA7	15:308571	q1	q2	1.906	-36.497	0	0	yes	0.049072	0.0455282	0.0526158	0.329945	0.305057	0.354834
ENSG00000186792	ENSG00000186792	HYAL3	3:5031645	q1	q2	1.913	-5.637	0	0	yes	1.78679	0.612038	2.96154	12.0995	10.0641	14.1349
ENSG00000212978	ENSG00000212978	AC016747.3	2:6129300	q1	q2	1.919	-6.15	0	0	yes	2.54299	1.10045	3.98553	17.3287	12.8206	21.8369
ENSG00000254673	ENSG00000254673	RP11-598P20.5	8:4270477	q1	q2	1.926	-3.575	0	0.006	yes	0.12693	0.109854	0.144007	0.871056	0	1.80229
ENSG00000179583	ENSG00000179583	CIITA	16:109710	q1	q2	1.927	-4.188	0	0.001	yes	0.190623	0.0403065	0.34094	1.30873	0.688707	1.92876
ENSG00000206567	ENSG00000206567	AC022007.5	3:1000422	q1	q2	1.931	-8.186	0	0	yes	2.50596	1.43209	3.57984	17.2758	13.8695	20.6821
ENSG00000139988	ENSG00000139988	RDH12	14:681686	q1	q2	1.938	-4.171	0	0.001	yes	0.185436	0.173165	0.197707	1.28808	0.0941229	2.48204
ENSG00000244468	ENSG00000244468	RP11-206M11.7	3:1489471	q1	q2	1.938	-6.53	0	0	yes	3.62047	1.63852	5.60241	25.1388	19.3734	30.9043
ENSG00000119638	ENSG00000119638	NEK9	14:755488	q1	q2	1.987	-7.377	0	0	yes	2.03784	1.06419	3.01149	14.8654	11.166	18.5648
ENSG00000101230	ENSG00000101230	ISM1	20:132024	q1	q2	2.018	-4.274	0	0	yes	0.216234	0.0251371	0.40733	1.62717	1.08489	2.16944
ENSG00000184635	ENSG00000184635	ZNF93	19:200117	q1	q2	2.032	-4.683	0	0	yes	0.504256	0.173782	0.834729	3.84715	1.659	6.03531
ENSG00000161970	ENSG00000161970	RPL26	17:828083	q1	q2	2.038	-10.251	0	0	yes	7.74294	4.96159	10.5243	59.4416	49.3006	69.5826
ENSG00000255185	ENSG00000255185	RP11-106J23.2	16:700101	q1	q2	2.043	-7.414	0	0	yes	2.87993	1.43939	4.32048	22.2224	17.0764	27.3685
ENSG00000106069	ENSG00000106069	CHN2	7:2903484	q1	q2	2.083	-5.755	0	0	yes	1.82408	0.608177	3.03999	14.6395	10.5117	18.7673
ENSG00000249755	ENSG00000249755	RP11-466G12.2	4:8944213	q1	q2	2.113	-40.3	0	0	yes	0.0148092	0.013727	0.0158915	0.122538	0.113321	0.131755

ENSG00000225673	ENSG00000225673	RP11-641C17.3	3:5973503	q1	q2	2.115	-3.548	0	0.007	yes	0.778658	0	1.65038	6.45328	3.813	9.09356
ENSG00000204977	ENSG00000204977	TRIM13	13:505700	q1	q2	2.127	-5.242	0	0	yes	1.13578	0.427189	1.84437	9.52789	4.58279	14.473
ENSG00000173451	ENSG00000173451	THAP2	12:720032	q1	q2	2.134	-5.739	0	0	yes	1.51426	0.476604	2.55192	12.7893	9.09748	16.4811
ENSG00000233221	ENSG00000233221	AC133785.1	2:1315885	q1	q2	2.162	-6.298	0	0	yes	0.0732072	0.0621392	0.0842751	0.635898	0.210067	1.06173
ENSG00000180815	ENSG00000180815	MAP3K15	X:1936201	q1	q2	2.194	-5.147	0	0	yes	0.0302326	0.0284919	0.0319732	0.271118	0.040568	0.501668
ENSG00000216324	ENSG00000216324	RP3-382I10.2	6:8811770	q1	q2	2.217	-4.735	0	0	yes	0.543705	0.500312	0.587097	4.99061	0.333962	9.64726
ENSG00000105825	ENSG00000105825	TFPI2	7:9322088	q1	q2	2.236	-4.713	0	0	yes	1.05783	0.112055	2.00361	9.89849	6.75333	13.0436
ENSG00000122026	ENSG00000122026	RPL21	13:278254	q1	q2	2.346	-11.79	0	0	yes	8.99035	5.66021	12.3205	93.9132	80.2432	107.583
ENSG00000146376	ENSG00000146376	ARHGAP18	6:1298972	q1	q2	2.352	-18.312	0	0	yes	4.86966	3.97277	5.76655	51.142	41.9876	60.2964
ENSG00000179902	ENSG00000179902	C1orf194	1:1096485	q1	q2	2.354	-3.841	0	0.003	yes	1.22358	0	2.68969	12.8782	9.55248	16.2039
ENSG00000234608	ENSG00000234608	AC003029.1	12:112277	q1	q2	2.364	-12.622	0	0	yes	4.09244	2.63422	5.55067	43.523	38.4902	48.5558
ENSG00000247708	ENSG00000247708	RP11-323F5.2	4:4349866	q1	q2	2.385	-3.545	0	0.007	yes	0.506728	0	1.15048	5.50166	3.06128	7.94204
ENSG00000198496	ENSG00000198496	NBR2	17:411963	q1	q2	2.413	-9.936	0	0	yes	0.136018	0.123022	0.149014	1.51897	0.79563	2.24231
ENSG00000168505	ENSG00000168505	GBX2	2:2370738	q1	q2	2.432	-5.077	0	0	yes	0.245329	0.0401342	0.450524	2.79161	1.48826	4.09495
ENSG00000197647	ENSG00000197647	ZNF433	19:120984	q1	q2	2.435	-6.956	0	0	yes	0.227162	0.143737	0.310587	2.59311	1.04765	4.13857
ENSG00000224295	ENSG00000224295	AC087380.14	11:527441	q1	q2	2.468	-5.296	0	0	yes	0.033674	0.0268004	0.0405477	0.397277	0.0360361	0.758519
ENSG00000185666	ENSG00000185666	SYN3	22:329085	q1	q2	2.483	-6.012	0	0	yes	0.104849	0.0988546	0.110843	1.2561	0.221008	2.29119
ENSG00000223442	ENSG00000223442	AC004041.2	5:1318771	q1	q2	2.529	-6.876	0	0	yes	3.69546	1.08099	6.30993	46.3407	37.0097	55.6718
ENSG00000234695	ENSG00000234695	AC002076.10	7:9322088	q1	q2	2.542	-4.749	0	0	yes	0.0924012	0.0723565	0.112446	1.17368	0	2.40397
ENSG00000237298	ENSG00000237298	AC009948.3	2:1793875	q1	q2	2.551	-13.328	0	0	yes	0.0103548	0.0074788	0.0132308	0.132683	0.0977447	0.167621
ENSG00000185736	ENSG00000185736	ADARB2	10:122807	q1	q2	2.578	-4.109	0	0.001	yes	0.210153	0	0.430822	2.76844	0.866335	4.67054
ENSG00000256980	ENSG00000256980	RP11-257K9.7	6:7391859	q1	q2	2.617	-3.584	0	0.006	yes	0.55489	0	1.34947	7.59817	5.41906	9.77729
ENSG00000180929	ENSG00000180929	GPR62	3:5198932	q1	q2	2.622	-4.169	0	0.001	yes	0.0097482	0.0092396	0.0102569	0.134177	0	0.302794
ENSG00000248710	ENSG00000248710	RP11-432B6.3	3:1599434	q1	q2	2.624	-4.874	0	0	yes	0.111685	0	0.227407	1.5407	1.0885	1.9929
ENSG00000167588	ENSG00000167588	GPD1	12:504976	q1	q2	2.673	-3.547	0	0.007	yes	0.0986082	0	0.212471	1.42809	0.04445	2.81173
ENSG00000248778	ENSG00000248778	RP11-311D14.1	4:1064737	q1	q2	2.693	-7.541	0	0	yes	0.0662568	0.0632788	0.0692348	0.978738	0.281156	1.67632
ENSG00000240793	ENSG00000240793	CTD-2600H12.1	16:482008	q1	q2	2.697	-4.227	0	0.001	yes	0.0954616	0.089927	0.100996	1.41577	0	3.22052
ENSG00000236449	ENSG00000236449	AC018890.6	2:1754118	q1	q2	2.702	-20.629	0	0	yes	0.0027134	0.0022427	0.00318401	0.0404733	0.0325257	0.0484208
ENSG00000023839	ENSG00000023839	ABCC2	10:101542	q1	q2	2.706	-3.774	0	0.003	yes	0.162058	0	0.361603	2.42529	0.642922	4.20766
ENSG00000232748	ENSG00000232748	AC135050.1	16:310721	q1	q2	2.728	-4.412	0	0	yes	0.607486	0	1.33301	9.2956	6.30971	12.2815
ENSG00000145425	ENSG00000145425	RPS3A	4:1520207	q1	q2	2.754	-8.012	0	0	yes	4.114	1.37413	6.85387	64.6073	53.5846	75.6299
ENSG00000240216	ENSG00000240216	CPHL1P	3:1489471	q1	q2	2.784	-4.117	0	0.001	yes	0.154785	0.0195554	0.290015	2.50556	0	5.09235
ENSG00000242128	ENSG00000242128	AC079767.2	2:2081043	q1	q2	2.786	-6.675	0	0	yes	0.524226	0.46535	0.583101	8.49849	1.46947	15.5275
ENSG00000254553	ENSG00000254553	RP1-27O5.3	1:3293065	q1	q2	2.812	-7.022	0	0	yes	0.0128008	0.0103924	0.0152092	0.213102	0.0471962	0.379009
ENSG00000225951	ENSG00000225951	RP11-339B21.9	9:1312174	q1	q2	2.813	-6.461	0	0	yes	0.16269	0.152663	0.172718	2.70964	0.35618	5.06309
ENSG00000249967	ENSG00000249967	RP11-548K23.11	10:993440	q1	q2	2.82	-6.113	0	0	yes	0.0179821	0.0165248	0.0194395	0.301567	0.0244568	0.578678
ENSG00000176386	ENSG00000176386	CDC26	9:1159838	q1	q2	2.857	-6.659	0	0	yes	1.08979	0.179418	2.00017	18.9701	15.2531	22.687
ENSG00000237307	ENSG00000237307	SRRM1P3	X:1359915	q1	q2	2.886	-3.802	0	0.003	yes	0.171164	0	0.423362	3.06757	1.9443	4.19085
ENSG00000243284	ENSG00000243284	VSIG8	1:1597965	q1	q2	2.923	-4.376	0	0	yes	0.0082348	0.0057519	0.0107176	0.153124	0	0.35241
ENSG00000158859	ENSG00000158859	ADAMTS4	1:1611595	q1	q2	2.926	-3.503	0	0.008	yes	0.0360954	0	0.0936158	0.67347	0.335593	1.01135
ENSG00000224489	ENSG00000224489	RP11-179H18.8	10:121410	q1	q2	2.949	-3.567	0	0.007	yes	0.604036	0	1.57732	11.5274	7.25468	15.8002
ENSG00000163467	ENSG00000163467	C1orf182	1:1562787	q1	q2	2.989	-7.226	0	0	yes	1.12327	0.243076	2.00346	22.3246	16.3929	28.2562
ENSG00000247853	ENSG00000247853	AC006064.1	12:667924	q1	q2	2.993	-5.067	0	0	yes	0.007559	0.0073446	0.00777333	0.150735	0	0.328735
ENSG00000249825	ENSG00000249825	CTD-2201I18.1	5:7928713	q1	q2	3.068	-4.826	0	0	yes	0.006362	0.0023352	0.0103888	0.136711	0	0.287433
ENSG00000111837	ENSG00000111837	MAK	6:1074797	q1	q2	3.083	-12.236	0	0	yes	0.0497479	0.0333577	0.0661382	1.08533	0.671553	1.4991
ENSG00000152256	ENSG00000152256	PDK1	2:1732920	q1	q2	3.109	-12.657	0	0	yes	0.185204	0.149251	0.221158	4.14895	2.27652	6.02138
ENSG00000250741	ENSG00000250741	NT5C1B-RDH14	2:1873598	q1	q2	3.111	-3.719	0	0.004	yes	0.0731667	0	0.192669	1.64175	1.04619	2.23732
ENSG00000080573	ENSG00000080573	COL5A3	19:100702	q1	q2	3.2	-4.606	0	0	yes	0.0475538	0	0.102464	1.16628	0.264895	2.06767
ENSG00000153071	ENSG00000153071	DAB2	5:3928436	q1	q2	3.248	-8.876	0	0		1.33349	0.529906	2.13708	34.3386	20.0717	48.6055
ENSG00000216306	ENSG00000216306	RP11-3K15.1	12:952279	q1	q2	3.409	-5.577	0	0	yes	0.346277	0	0.758161	10.4662	7.51591	13.4165

ENSG00000198189	ENSG00000198189	HSD17B11	4:8822494	q1	q2	3.482	-8.41	0	0	yes	0.156701	0.0962387	0.217164	5.09497	1.36234	8.8276
ENSG00000245562	ENSG00000245562	RP11-367J11.3	4:7096297	q1	q2	3.557	-4.11	0	0.001	yes	0.118576	0	0.246811	4.15765	0	9.77603
ENSG00000067601	ENSG00000067601	PMS2P4	7:6675252	q1	q2	3.559	-3.955	0	0.002	yes	0.20165	0	0.555821	7.08481	4.30618	9.86343
ENSG00000163815	ENSG00000163815	CLEC3B	3:4495674	q1	q2	3.661	-7.706	0	0	yes	0.609817	0.0412227	1.17841	23.7108	19.3952	28.0264
ENSG00000235471	ENSG00000235471	AL033532.1	1:1680487	q1	q2	3.725	-12.206	0	0	yes	0.0159195	0.0115461	0.020293	0.660224	0.300376	1.02007
ENSG00000159409	ENSG00000159409	CELF3	1:1516735	q1	q2	3.732	-14.479	0	0	yes	0.270483	0.176121	0.364844	11.2932	7.00741	15.5789
ENSG00000226352	ENSG00000226352	RP11-523H24.3	13:202487	q1	q2	3.759	-7.338	0	0	yes	0.0535864	0.0505624	0.0566105	2.29863	0	4.64989
ENSG00000258545	ENSG00000258545	RP4-755D9.1	X:1191702	q1	q2	3.931	-4.764	0	0	yes	0.0952527	0	0.243815	4.85456	2.23496	7.47415
ENSG00000219665	ENSG00000219665	CTD-2006C1.2	19:120984	q1	q2	4.038	-10.814	0	0	yes	0.0427405	0.0311052	0.0543757	2.42335	0.738139	4.10857
ENSG00000157510	ENSG00000157510	AFAP1L1	5:1485210	q1	q2	4.19	-8.857	0	0	yes	0.0078254	0.0070775	0.00857317	0.516824	0.030277	1.00337
ENSG00000155269	ENSG00000155269	GPR78	4:8560451	q1	q2	4.404	-36.812	0	0	yes	0.0018481	0.0015288	0.00216736	0.151127	0.126112	0.176142
ENSG00000222020	ENSG00000222020	AC062017.1	2:2399698	q1	q2	4.523	-31.32	0	0	yes	0.364812	0.30345	0.426175	33.6094	25.7176	41.5011
ENSG00000235374	ENSG00000235374	C21orf122	21:464916	q1	q2	4.523	-13.296	0	0	yes	0.0085369	0.0079049	0.0091688	0.786386	0.254543	1.31823
ENSG00000213641	ENSG00000213641	RP11-132J14.1	14:559659	q1	q2	5.031	-10.589	0	0	yes	0.0053556	0.0051617	0.00554955	0.819855	0.0413656	1.59834
ENSG00000241635	ENSG00000241635	UGT1A1	2:2345262	q1	q2	7.583	-66.221	0	0	yes	0.002802	0.0025302	0.00307385	5.50214	4.36078	6.64351
ENSG00000085563	ENSG00000085563	ABCB1	7:8713294	q1	q2	1.79769e+	1.79769e+	0	0.004	yes	0	0	0	0.197667	0.086185	0.309148
ENSG00000103832	ENSG00000103832	RP5-1086D14.1	15:308571	q1	q2	1.79769e+	1.79769e+	0	0	yes	0	0	0	0.058954	0.054507	0.0634009
ENSG00000124693	ENSG00000124693	HIST1H3B	6:2602190	q1	q2	1.79769e+	1.79769e+	0	0	yes	0	0	0	0.108234	0.0572119	0.159256
ENSG00000131435	ENSG00000131435	PDLIM4	5:1315205	q1	q2	1.79769e+	1.79769e+	0	0	yes	0	0	0	0.0751743	0.0711004	0.0792482
ENSG00000134571	ENSG00000134571	MYBPC3	11:472907	q1	q2	1.79769e+	1.79769e+	0	0	yes	0	0	0	0.0277969	0.0266422	0.0289516
ENSG00000147036	ENSG00000147036	LANCL3	X:3720852	q1	q2	1.79769e+	1.79769e+	0	0	yes	0	0	0	0.0342695	0.0322294	0.0363095
ENSG00000152580	ENSG00000152580	IGSF10	3:1508034	q1	q2	1.79769e+	1.79769e+	0	0	yes	0	0	0	2.41747	1.5936	3.24134
ENSG00000163661	ENSG00000163661	PTX3	3:1568930	q1	q2	1.79769e+	1.79769e+	0	0	yes	0	0	0	0.0329672	0.0205067	0.0454276
ENSG00000165182	ENSG00000165182	CXorf58	X:2385146	q1	q2	1.79769e+	1.79769e+	0	0	yes	0	0	0	0.057168	0.0403911	0.073945
ENSG00000167094	ENSG00000167094	TTC16	9:1303745	q1	q2	1.79769e+	1.79769e+	0	0	yes	0	0	0	0.0168734	0.0158601	0.0178868
ENSG00000168386	ENSG00000168386	FILIP1L	3:9953667	q1	q2	1.79769e+	1.79769e+	0	0	yes	0	0	0	0.0462304	0.0372395	0.0552212
ENSG00000173988	ENSG00000173988	LRRC63	13:467860	q1	q2	1.79769e+	1.79769e+	0	0	yes	0	0	0	1.01536	0.524328	1.50639
ENSG00000176029	ENSG00000176029	C11orf16	11:893268	q1	q2	1.79769e+	1.79769e+	0	0	yes	0	0	0	0.0325405	0.0235977	0.0414834
ENSG00000176076	ENSG00000176076	KCNE1L	X:1088669	q1	q2	1.79769e+	1.79769e+	0	0	yes	0	0	0	0.060796	0.0476844	0.0739077
ENSG00000177725	ENSG00000177725	AC105206.1	8:2202224	q1	q2	1.79769e+	1.79769e+	0	0	yes	0	0	0	0.0083735	0.0073562	0.0093908
ENSG00000178440	ENSG00000178440	RP11-324H6.6	10:513713	q1	q2	1.79769e+	1.79769e+	0	0	yes	0	0	0	12.3659	8.51555	16.2162
ENSG00000183273	ENSG00000183273	CCDC60	12:119772	q1	q2	1.79769e+	1.79769e+	0	0	yes	0	0	0	0.0285123	0.0260211	0.0310035
ENSG00000183395	ENSG00000183395	PMCH	12:102513	q1	q2	1.79769e+	1.79769e+	0	0	yes	0	0	0	0.0875544	0.0728596	0.102249
ENSG00000184608	ENSG00000184608	C8orf12	8:1119714	q1	q2	1.79769e+	1.79769e+	0	0	yes	0	0	0	4.0335	2.53197	5.53503
ENSG00000197882	ENSG00000197882	OR7E13P	11:865021	q1	q2	1.79769e+	1.79769e+	0	0	yes	0	0	0	0.142553	0.136699	0.148407
ENSG00000199545	ENSG00000199545	5S_rRNA	5:1382824	q1	q2	1.79769e+	1.79769e+	0	0	yes	0	0	0	15.7448	14.9644	16.5253
ENSG00000200091	ENSG00000200091	7SK	6:7631122	q1	q2	1.79769e+	1.79769e+	0	0	yes	0	0	0	0.7953	0.735181	0.855419
ENSG00000200851	ENSG00000200851	SNORD27	11:626194	q1	q2	1.79769e+	1.79769e+	0	0	yes	0	0	0	113.686	102.721	124.65
ENSG00000201207	ENSG00000201207	Y_RNA	6:3162900	q1	q2	1.79769e+	1.79769e+	0	0	yes	0	0	0	18.0368	17.3654	18.7081
ENSG00000201448	ENSG00000201448	SNORA63	1:3688142	q1	q2	1.79769e+	1.79769e+	0	0	yes	0	0	0	9.1308	6.75334	11.5083
ENSG00000201778	ENSG00000201778	Y_RNA	3:1568642	q1	q2	1.79769e+	1.79769e+	0	0	yes	0	0	0	24.8455	23.0323	26.6588
ENSG00000202058	ENSG00000202058	7SK	22:429496	q1	q2	1.79769e+	1.79769e+	0	0	yes	0	0	0	0.445664	0.277219	0.614109
ENSG00000202314	ENSG00000202314	SNORD6	11:933948	q1	q2	1.79769e+	1.79769e+	0	0	yes	0	0	0	79.3626	72.6311	86.094
ENSG00000202441	ENSG00000202441	RNY4P10	6:3316136	q1	q2	1.79769e+	1.79769e+	0	0	yes	0	0	0	0.764733	0.742707	0.786758
ENSG00000203276	ENSG00000203276	AL353898.2	1:5449734	q1	q2	1.79769e+	1.79769e+	0	0	yes	0	0	0	0.0348014	0.0326036	0.0369991
ENSG00000203301	ENSG00000203301	AL590822.1	1:1981908	q1	q2	1.79769e+	1.79769e+	0	0	yes	0	0	0	0.0170267	0.0159573	0.0180961
ENSG00000207392	ENSG00000207392	SNORA20	6:1601000	q1	q2	1.79769e+	1.79769e+	0	0	yes	0	0	0	0.526316	0.500106	0.552526
ENSG00000207437	ENSG00000207437	SNORD28	11:626194	q1	q2	1.79769e+	1.79769e+	0	0	yes	0	0	0	96.6995	87.373	106.026
ENSG00000208892	ENSG00000208892	SNORA49	12:132434	q1	q2	1.79769e+	1.79769e+	0	0	yes	0	0	0	11.7163	11.1599	12.2726
ENSG00000211535	ENSG00000211535	MIR711	3:4860150	q1	q2	1.79769e+	1.79769e+	0	0	yes	0	0	0	36.9303	31.1627	42.6978

ENSG00000212125	ENSG00000212125	TAS2R15	12:109775	q1	q2	1.79769e+	1.79769e+	0	0	yes	0	0	0	0.0522871	0.0431504	0.0614238
ENSG00000213443	ENSG00000213443	NPM1P5	12:980627	q1	q2	1.79769e+	1.79769e+	0	0	yes	0	0	0	0.0279169	0.0215123	0.0343215
ENSG00000213484	ENSG00000213484	EIF4A1P8	10:938083	q1	q2	1.79769e+	1.79769e+	0	0	yes	0	0	0	0.0696609	0.0582087	0.0811131
ENSG00000213592	ENSG00000213592	AP000662.9	11:574800	q1	q2	1.79769e+	1.79769e+	0	0	yes	0	0	0	0.0781918	0.0749246	0.081459
ENSG00000214188	ENSG00000214188	ST7OT4	7:1165924	q1	q2	1.79769e+	1.79769e+	0	0	yes	0	0	0	0.0296016	0.0261369	0.0330662
ENSG00000215127	ENSG00000215127	RP11-35D5.1	4:6856699	q1	q2	1.79769e+	1.79769e+	0	0	yes	0	0	0	0.0073672	0.0061393	0.0085951
ENSG00000215208	ENSG00000215208	RP11-316O1.1	12:656724	q1	q2	1.79769e+	1.79769e+	0	0	yes	0	0	0	0.0242847	0.0143705	0.0341989
ENSG00000215297	ENSG00000215297	RP11-62E14.2	9:3218296	q1	q2	1.79769e+	1.79769e+	0	0	yes	0	0	0	0.0607	0.0377576	0.0836425
ENSG00000215791	ENSG00000215791	AL645728.2	1:1477052	q1	q2	1.79769e+	1.79769e+	0	0	yes	0	0	0	3.48001	2.61589	4.34414
ENSG00000216642	ENSG00000216642	RP1-95L4.3	6:1433816	q1	q2	1.79769e+	1.79769e+	0	0	yes	0	0	0	0.309157	0.27582	0.342494
ENSG00000219433	ENSG00000219433	RP11-350J20.4	6:1500454	q1	q2	1.79769e+	1.79769e+	0	0	yes	0	0	0	0.0784738	0.0745316	0.082416
ENSG00000221182	ENSG00000221182	SNORD98	10:704807	q1	q2	1.79769e+	1.79769e+	0	0	yes	0	0	0	46.8607	43.9333	49.7881
ENSG00000221267	ENSG00000221267	MIR1236	6:3191986	q1	q2	1.79769e+	1.79769e+	0	0	yes	0	0	0	14.2076	13.6893	14.7259
ENSG00000221420	ENSG00000221420	SNORA81	3:1865009	q1	q2	1.79769e+	1.79769e+	0	0	yes	0	0	0	2.86714	2.67504	3.05925
ENSG00000222345	ENSG00000222345	SNORD19	3:5252935	q1	q2	1.79769e+	1.79769e+	0	0	yes	0	0	0	110.321	106.837	113.804
ENSG00000222489	ENSG00000222489	SNORA79	14:207795	q1	q2	1.79769e+	1.79769e+	0	0	yes	0	0	0	0.927389	0.745513	1.10926
ENSG00000223505	ENSG00000223505	RP11-397P13.7	1:2037647	q1	q2	1.79769e+	1.79769e+	0	0	yes	0	0	0	0.194362	0.179542	0.209182
ENSG00000223916	ENSG00000223916	RP11-353H3.1	3:4332800	q1	q2	1.79769e+	1.79769e+	0	0	yes	0	0	0	0.100165	0.0927608	0.10757
ENSG00000223969	ENSG00000223969	AC002456.2	7:8996453	q1	q2	1.79769e+	1.79769e+	0	0	yes	0	0	0	0.364801	0.339229	0.390374
ENSG00000224011	ENSG00000224011	AC111155.1	17:587551	q1	q2	1.79769e+	1.79769e+	0	0	yes	0	0	0	0.226652	0.221251	0.232053
ENSG00000224020	ENSG00000224020	MIR181A2HG	9:1272798	q1	q2	1.79769e+	1.79769e+	0	0	yes	0	0	0	0.104094	0.0782714	0.129916
ENSG00000224315	ENSG00000224315	RP4-633I8.2	1:8412456	q1	q2	1.79769e+	1.79769e+	0	0	yes	0	0	0	0.0809305	0.0772201	0.0846409
ENSG00000224356	ENSG00000224356	RP11-151A6.4	13:101183	q1	q2	1.79769e+	1.79769e+	0	0	yes	0	0	0	0.0757774	0.0485573	0.102997
ENSG00000224401	ENSG00000224401	RPL7P57	X:4769630	q1	q2	1.79769e+	1.79769e+	0	0	yes	0	0	0	0.0708961	0.062907	0.0788852
ENSG00000224502	ENSG00000224502	RP11-414H17.8	10:618688	q1	q2	1.79769e+	1.79769e+	0	0	yes	0	0	0	0.335206	0.302648	0.367764
ENSG00000224665	ENSG00000224665	RP11-197N18.2	12:123405	q1	q2	1.79769e+	1.79769e+	0	0	yes	0	0	0	0.68054	0.45105	0.91003
ENSG00000224675	ENSG00000224675	AC009227.2	2:1547284	q1	q2	1.79769e+	1.79769e+	0	0	yes	0	0	0	0.0317107	0.0228299	0.0405915
ENSG00000224884	ENSG00000224884	AC034187.2	3:7994491	q1	q2	1.79769e+	1.79769e+	0	0	yes	0	0	0	0.0908641	0.0824367	0.0992915
ENSG00000224892	ENSG00000224892	RPS4XP16	13:519099	q1	q2	1.79769e+	1.79769e+	0	0	yes	0	0	0	0.0572138	0.0482235	0.0662041
ENSG00000225251	ENSG00000225251	RP11-730A19.2	10:129386	q1	q2	1.79769e+	1.79769e+	0	0	yes	0	0	0	0.0534545	0.0482989	0.0586101
ENSG00000225408	ENSG00000225408	RP11-207C16.4	9:5629024	q1	q2	1.79769e+	1.79769e+	0	0	yes	0	0	0	0.263439	0.248223	0.278655
ENSG00000225611	ENSG00000225611	RP11-70C1.1	3:4273415	q1	q2	1.79769e+	1.79769e+	0	0	yes	0	0	0	0.0336616	0.0209387	0.0463845
ENSG00000225782	ENSG00000225782	RP11-322F10.2	1:2207015	q1	q2	1.79769e+	1.79769e+	0	0	yes	0	0	0	0.0575351	0.0462516	0.0688187
ENSG00000226769	ENSG00000226769	GAPDHP54	20:455232	q1	q2	1.79769e+	1.79769e+	0	0	yes	0	0	0	0.0447208	0.0404471	0.0489945
ENSG00000227056	ENSG00000227056	RP11-418J17.2	1:1195738	q1	q2	1.79769e+	1.79769e+	0	0	yes	0	0	0	0.072108	0.0622264	0.0819895
ENSG00000227694	ENSG00000227694	AC005884.1	17:587551	q1	q2	1.79769e+	1.79769e+	0	0	yes	0	0	0	0.213773	0.208679	0.218867
ENSG00000227848	ENSG00000227848	SUCLA2-AS1	13:485106	q1	q2	1.79769e+	1.79769e+	0	0	yes	0	0	0	0.290449	0.240819	0.340078
ENSG00000228063	ENSG00000228063	RP11-135J2.4	1:2192599	q1	q2	1.79769e+	1.79769e+	0	0.002	yes	0	0	0	2.76988	1.27856	4.26121
ENSG00000228093	ENSG00000228093	AC005162.3	7:2903484	q1	q2	1.79769e+	1.79769e+	0	0	yes	0	0	0	0.0539476	0.0396565	0.0682386
ENSG00000228477	ENSG00000228477	RP3-342P20.2	1:4042080	q1	q2	1.79769e+	1.79769e+	0	0	yes	0	0	0	0.0956921	0.0744272	0.116957
ENSG00000228501	ENSG00000228501	RPL15P18	13:100741	q1	q2	1.79769e+	1.79769e+	0	0	yes	0	0	0	0.230331	0.183797	0.276865
ENSG00000228676	ENSG00000228676	AC069259.1	3:1717574	q1	q2	1.79769e+	1.79769e+	0	0	yes	0	0	0	0.0595722	0.0523876	0.0667569
ENSG00000228703	ENSG00000228703	RP5-1160K1.6	1:1101587	q1	q2	1.79769e+	1.79769e+	0	0	yes	0	0	0	3.71391	1.92424	5.50359
ENSG00000229628	ENSG00000229628	AC073115.7	7:4603050	q1	q2	1.79769e+	1.79769e+	0	0.008	yes	0	0	0	0.347845	0.138086	0.557603
ENSG00000230005	ENSG00000230005	RP5-870F10.4	1:2279162	q1	q2	1.79769e+	1.79769e+	0	0	yes	0	0	0	0.0547927	0.0516932	0.0578923
ENSG00000230118	ENSG00000230118	AC092569.2	2:3094563	q1	q2	1.79769e+	1.79769e+	0	0	yes	0	0	0	1.10354	0.568243	1.63883
ENSG00000230424	ENSG00000230424	RP1-43E13.2	1:1953699	q1	q2	1.79769e+	1.79769e+	0	0.003	yes	0	0	0	4.61331	2.09545	7.13117
ENSG00000230741	ENSG00000230741	SC22CB-1D7.1	22:335600	q1	q2	1.79769e+	1.79769e+	0	0	yes	0	0	0	0.255544	0.242299	0.268789
ENSG00000230753	ENSG00000230753	RP4-553F4.6	20:323167	q1	q2	1.79769e+	1.79769e+	0	0	yes	0	0	0	0.0947133	0.0739183	0.115508
ENSG00000230955	ENSG00000230955	RP11-109P14.10	1:3832636	q1	q2	1.79769e+	1.79769e+	0	0	yes	0	0	0	0.258785	0.220629	0.296941

ENSG00000231125	ENSG00000231125	AF129075.5	21:304281	q1	q2	1.79769e+	1.79769e+	0	0	yes	0	0	0	0.54685	0.479021	0.614678
ENSG00000231563	ENSG00000231563	RP11-245P10.4	1:2285950	q1	q2	1.79769e+	1.79769e+	0	0	yes	0	0	0	0.307311	0.162443	0.452179
ENSG00000231799	ENSG00000231799	RP13-93L13.2	9:9197570	q1	q2	1.79769e+	1.79769e+	0	0	yes	0	0	0	0.0388102	0.0354575	0.0421629
ENSG00000231980	ENSG00000231980	AC011899.10	7:1573317	q1	q2	1.79769e+	1.79769e+	0	0	yes	0	0	0	0.0207197	0.0159025	0.025537
ENSG00000232021	ENSG00000232021	RP11-558N14.1	4:1089687	q1	q2	1.79769e+	1.79769e+	0	0	yes	0	0	0	0.0426107	0.0244177	0.0608036
ENSG00000232398	ENSG00000232398	TMPRSS11CP	4:6856699	q1	q2	1.79769e+	1.79769e+	0	0	yes	0	0	0	0.0332757	0.0277297	0.0388216
ENSG00000232479	ENSG00000232479	AC010900.2	2:2037453	q1	q2	1.79769e+	1.79769e+	0	0	yes	0	0	0	0.031262	0.0267614	0.0357626
ENSG00000232564	ENSG00000232564	RP4-591N18.2	22:408062	q1	q2	1.79769e+	1.79769e+	0	0	yes	0	0	0	0.0034625	0.0031814	0.0037435
ENSG00000232633	ENSG00000232633	CTD-2201G3.1	5:1123577	q1	q2	1.79769e+	1.79769e+	0	0	yes	0	0	0	0.0059553	0.005225	0.0066856
ENSG00000232682	ENSG00000232682	RP11-388P9.2	10:617881	q1	q2	1.79769e+	1.79769e+	0	0	yes	0	0	0	0.0696254	0.0640057	0.0752452
ENSG00000232788	ENSG00000232788	AC078883.3	2:1732920	q1	q2	1.79769e+	1.79769e+	0	0	yes	0	0	0	0.159263	0.129394	0.189131
ENSG00000233108	ENSG00000233108	AC006042.7	7:7676148	q1	q2	1.79769e+	1.79769e+	0	0	yes	0	0	0	0.135226	0.12523	0.145222
ENSG00000233436	ENSG00000233436	BTBD18	11:574800	q1	q2	1.79769e+	1.79769e+	0	0	yes	0	0	0	0.0409761	0.0392639	0.0426883
ENSG00000233509	ENSG00000233509	ZNF197-AS1	3:4459668	q1	q2	1.79769e+	1.79769e+	0	0	yes	0	0	0	0.119683	0.102883	0.136483
ENSG00000233589	ENSG00000233589	RP4-694A7.2	1:6893983	q1	q2	1.79769e+	1.79769e+	0	0	yes	0	0	0	0.0641141	0.0398812	0.0883469
ENSG00000233733	ENSG00000233733	H2AFZP6	22:317955	q1	q2	1.79769e+	1.79769e+	0	0	yes	0	0	0	0.0640848	0.0619578	0.0662118
ENSG00000233789	ENSG00000233789	RP11-44D15.2	10:979514	q1	q2	1.79769e+	1.79769e+	0	0	yes	0	0	0	0.0547752	0.0332906	0.0762599
ENSG00000234084	ENSG00000234084	RP3-388E23.2	6:1356046	q1	q2	1.79769e+	1.79769e+	0	0	yes	0	0	0	0.233023	0.196519	0.269526
ENSG00000234185	ENSG00000234185	RP5-1132H15.1	7:6554078	q1	q2	1.79769e+	1.79769e+	0	0	yes	0	0	0	0.178863	0.169602	0.188124
ENSG00000234233	ENSG00000234233	KCNH1-IT1	1:2108565	q1	q2	1.79769e+	1.79769e+	0	0	yes	0	0	0	0.126906	0.0862634	0.167548
ENSG00000234740	ENSG00000234740	RP11-272P10.2	9:1310570	q1	q2	1.79769e+	1.79769e+	0	0	yes	0	0	0	0.32308	0.291846	0.354313
ENSG00000234913	ENSG00000234913	XXbac-B476C20.1	22:182704	q1	q2	1.79769e+	1.79769e+	0	0	yes	0	0	0	0.0177054	0.0167002	0.0187106
ENSG00000235267	ENSG00000235267	AC074117.13	2:2766523	q1	q2	1.79769e+	1.79769e+	0	0	yes	0	0	0	0.347427	0.321126	0.373728
ENSG00000235586	ENSG00000235586	AC011247.3	2:3878911	q1	q2	1.79769e+	1.79769e+	0	0	yes	0	0	0	1.26492	1.15646	1.37339
ENSG00000235848	ENSG00000235848	AC016689.1	2:3815032	q1	q2	1.79769e+	1.79769e+	0	0	yes	0	0	0	0.276224	0.252774	0.299674
ENSG00000236842	ENSG00000236842	RP11-399K21.10	10:771903	q1	q2	1.79769e+	1.79769e+	0	0	yes	0	0	0	0.174538	0.109716	0.239359
ENSG00000236869	ENSG00000236869	RP11-944L7.4	3:4459668	q1	q2	1.79769e+	1.79769e+	0	0	yes	0	0	0	0.241311	0.207437	0.275184
ENSG00000236997	ENSG00000236997	RP5-934G17.2	1:1182184	q1	q2	1.79769e+	1.79769e+	0	0	yes	0	0	0	0.455473	0.420095	0.490851
ENSG00000237259	ENSG00000237259	RP4-568F9.3	20:182691	q1	q2	1.79769e+	1.79769e+	0	0	yes	0	0	0	0.307611	0.249736	0.365487
ENSG00000237263	ENSG00000237263	MAPK6PS3	13:426141	q1	q2	1.79769e+	1.79769e+	0	0	yes	0	0	0	0.0417547	0.0351936	0.0483158
ENSG00000237827	ENSG00000237827	RP11-332O19.2	10:104845	q1	q2	1.79769e+	1.79769e+	0	0	yes	0	0	0	0.241104	0.22222	0.259989
ENSG00000237953	ENSG00000237953	AC013267.1	2:2516650	q1	q2	1.79769e+	1.79769e+	0	0	yes	0	0	0	0.628773	0.500425	0.757121
ENSG00000238278	ENSG00000238278	RP11-803B1.5	3:7546422	q1	q2	1.79769e+	1.79769e+	0	0.008	yes	0	0	0	0.118791	0.0471573	0.190425
ENSG00000238650	ENSG00000238650	SNORD54	8:5697985	q1	q2	1.79769e+	1.79769e+	0	0	yes	0	0	0	38.6268	36.5606	40.6929
ENSG00000238711	ENSG00000238711	RNY4P25	1:1513751	q1	q2	1.79769e+	1.79769e+	0	0	yes	0	0	0	22.2016	21.2298	23.1733
ENSG00000238828	ENSG00000238828	hsa-mir-1974	5:1342405	q1	q2	1.79769e+	1.79769e+	0	0	yes	0	0	0	5105.05	3299.31	6910.79
ENSG00000239607	ENSG00000239607	AC087651.1	17:730321	q1	q2	1.79769e+	1.79769e+	0	0	yes	0	0	0	1.8814	1.40353	2.35928
ENSG00000239688	ENSG00000239688	AP000338.1	21:478788	q1	q2	1.79769e+	1.79769e+	0	0	yes	0	0	0	0.456502	0.43117	0.481834
ENSG00000240005	ENSG00000240005	RP11-293A21.1	4:2685929	q1	q2	1.79769e+	1.79769e+	0	0	yes	0	0	0	0.443848	0.400789	0.486908
ENSG00000240032	ENSG00000240032	RP11-274H2.3	3:1457818	q1	q2	1.79769e+	1.79769e+	0	0	yes	0	0	0	0.0887817	0.0795506	0.0980127
ENSG00000240210	ENSG00000240210	RP11-204K16.1	14:682864	q1	q2	1.79769e+	1.79769e+	0	0	yes	0	0	0	8.07061	5.67415	10.4671
ENSG00000240596	ENSG00000240596	KCNAB1-AS2	3:1557554	q1	q2	1.79769e+	1.79769e+	0	0	yes	0	0	0	0.28586	0.2284	0.34332
ENSG00000240776	ENSG00000240776	RP11-435F17.1	3:1135470	q1	q2	1.79769e+	1.79769e+	0	0	yes	0	0	0	0.0996068	0.0802575	0.118956
ENSG00000240848	ENSG00000240848	RP1-211D12.3	20:435951	q1	q2	1.79769e+	1.79769e+	0	0	yes	0	0	0	3.33148	2.29747	4.36548
ENSG00000241499	ENSG00000241499	RP11-85G20.2	14:937038	q1	q2	1.79769e+	1.79769e+	0	0	yes	0	0	0	0.70182	0.65077	0.75287
ENSG00000241830	ENSG00000241830	AC012640.1	5:1035381	q1	q2	1.79769e+	1.79769e+	0	0	yes	0	0	0	0.0715432	0.0670761	0.0760103
ENSG00000242251	ENSG00000242251	AC005004.1	22:321499	q1	q2	1.79769e+	1.79769e+	0	0	yes	0	0	0	0.708996	0.627534	0.790457
ENSG00000242683	ENSG00000242683	CTB-46B19.1	5:1382824	q1	q2	1.79769e+	1.79769e+	0	0	yes	0	0	0	0.0224788	0.0213645	0.023593
ENSG00000242777	ENSG00000242777	AL590704.1	6:1446068	q1	q2	1.79769e+	1.79769e+	0	0	yes	0	0	0	6.9184	6.37939	7.4574
ENSG00000242833	ENSG00000242833	AC100832.3	17:568332	q1	q2	1.79769e+	1.79769e+	0	0	yes	0	0	0	0.783262	0.768029	0.798494

ENSG00000242941	ENSG00000242941	CTD-2325P2.2	14:682864	q1	q2	1.79769e+	1.79769e+	0	0	yes	0	0	0	0.156312	0.115612	0.197012
ENSG00000243305	ENSG00000243305	RP11-362A9.3	3:1519616	q1	q2	1.79769e+	1.79769e+	0	0	yes	0	0	0	0.100245	0.094572	0.105918
ENSG00000243420	ENSG00000243420	AC011611.1	12:759569	q1	q2	1.79769e+	1.79769e+	0	0	yes	0	0	0	0.952258	0.647291	1.25722
ENSG00000243431	ENSG00000243431	RPL5P30	11:118307	q1	q2	1.79769e+	1.79769e+	0	0	yes	0	0	0	0.0990896	0.0961704	0.102009
ENSG00000243959	ENSG00000243959	AC091934.1	5:1759695	q1	q2	1.79769e+	1.79769e+	0	0	yes	0	0	0	0.899148	0.841408	0.956888
ENSG00000244134	ENSG00000244134	RPS12P20	11:723961	q1	q2	1.79769e+	1.79769e+	0	0	yes	0	0	0	0.287338	0.281747	0.292929
ENSG00000244158	ENSG00000244158	RP1-93H18.6	6:1167819	q1	q2	1.79769e+	1.79769e+	0	0.003	yes	0	0	0	0.226546	0.100881	0.352211
ENSG00000244266	ENSG00000244266	RP11-112N23.1	12:507200	q1	q2	1.79769e+	1.79769e+	0	0	yes	0	0	0	0.300422	0.266886	0.333958
ENSG00000244676	ENSG00000244676	AL109761.5	21:191356	q1	q2	1.79769e+	1.79769e+	0	0	yes	0	0	0	13.5596	8.22367	18.8956
ENSG00000244697	ENSG00000244697	RP11-378J18.5	1:2227914	q1	q2	1.79769e+	1.79769e+	0	0	yes	0	0	0	0.0157497	0.0150186	0.0164809
ENSG00000244741	ENSG00000244741	AC008670.5	5:1342405	q1	q2	1.79769e+	1.79769e+	0	0	yes	0	0	0	4360.55	2642.7	6078.39
ENSG00000247275	ENSG00000247275	AL160008.1	1:8947235	q1	q2	1.79769e+	1.79769e+	0	0	yes	0	0	0	0.0599556	0.0412286	0.0786826
ENSG00000248127	ENSG00000248127	CTC-235G5.3	5:7537777	q1	q2	1.79769e+	1.79769e+	0	0	yes	0	0	0	0.174925	0.125936	0.223914
ENSG00000248423	ENSG00000248423	RP11-235P11.1	8:1527472	q1	q2	1.79769e+	1.79769e+	0	0	yes	0	0	0	0.0306276	0.0282923	0.032963
ENSG00000248546	ENSG00000248546	ANP32C	4:1644454	q1	q2	1.79769e+	1.79769e+	0	0	yes	0	0	0	0.0445444	0.0385106	0.0505783
ENSG00000248772	ENSG00000248772	RP11-639F1.1	3:1550933	q1	q2	1.79769e+	1.79769e+	0	0	yes	0	0	0	6.48609	5.11104	7.86113
ENSG00000248785	ENSG00000248785	AC004067.4	4:1104813	q1	q2	1.79769e+	1.79769e+	0	0	yes	0	0	0	0.559994	0.507831	0.612157
ENSG00000248881	ENSG00000248881	CTC-366B18.2	5:7466431	q1	q2	1.79769e+	1.79769e+	0	0	yes	0	0	0	4.46479	2.62356	6.30601
ENSG00000249144	ENSG00000249144	RP1-117B12.2	17:484505	q1	q2	1.79769e+	1.79769e+	0	0.001	yes	0	0	0	7.38464	3.62535	11.1439
ENSG00000249249	ENSG00000249249	AC010226.4	5:1149143	q1	q2	1.79769e+	1.79769e+	0	0	yes	0	0	0	0.0381475	0.0350756	0.0412193
ENSG00000249417	ENSG00000249417	RP11-438D8.2	3:1409475	q1	q2	1.79769e+	1.79769e+	0	0	yes	0	0	0	0.157466	0.140362	0.174571
ENSG00000249494	ENSG00000249494	CTB-161M19.4	5:1183420	q1	q2	1.79769e+	1.79769e+	0	0	yes	0	0	0	0.293461	0.266385	0.320537
ENSG00000249540	ENSG00000249540	RP11-789L4.1	3:1409475	q1	q2	1.79769e+	1.79769e+	0	0	yes	0	0	0	0.552145	0.492169	0.612122
ENSG00000250073	ENSG00000250073	RP11-677M14.3	11:124609	q1	q2	1.79769e+	1.79769e+	0	0	yes	0	0	0	0.0715792	0.060684	0.0824744
ENSG00000250101	ENSG00000250101	RP11-1252I4.2	5:1772364	q1	q2	1.79769e+	1.79769e+	0	0	yes	0	0	0	0.123515	0.0970309	0.15
ENSG00000250328	ENSG00000250328	CTC-210G5.1	5:1216470	q1	q2	1.79769e+	1.79769e+	0	0	yes	0	0	0	0.137662	0.111403	0.16392
ENSG00000250641	ENSG00000250641	XXbac-BPG32J3.1	6:3165472	q1	q2	1.79769e+	1.79769e+	0	0	yes	0	0	0	0.0132871	0.0124455	0.0141287
ENSG00000250659	ENSG00000250659	RP11-864I4.3	11:622010	q1	q2	1.79769e+	1.79769e+	0	0	yes	0	0	0	0.24207	0.232719	0.25142
ENSG00000250869	ENSG00000250869	RP11-453N18.1	8:6787633	q1	q2	1.79769e+	1.79769e+	0	0	yes	0	0	0	0.0406274	0.0383077	0.042947
ENSG00000251186	ENSG00000251186	RP11-689P11.3	4:8368438	q1	q2	1.79769e+	1.79769e+	0	0	yes	0	0	0	0.15423	0.139372	0.169088
ENSG00000251447	ENSG00000251447	RP11-517B11.6	3:1312523	q1	q2	1.79769e+	1.79769e+	0	0	yes	0	0	0	16.0086	14.5622	17.455
ENSG00000251821	ENSG00000251821	U6	4:1288020	q1	q2	1.79769e+	1.79769e+	0	0	yes	0	0	0	15.3456	13.6612	17.03
ENSG00000252304	ENSG00000252304	AP001781.1	11:111656	q1	q2	1.79769e+	1.79769e+	0	0	yes	0	0	0	22.5664	19.4884	25.6444
ENSG00000252412	ENSG00000252412	Y_RNA	22:374068	q1	q2	1.79769e+	1.79769e+	0	0	yes	0	0	0	12.2797	11.4272	13.1322
ENSG00000252564	ENSG00000252564	5S_rRNA	2:1621645	q1	q2	1.79769e+	1.79769e+	0	0	yes	0	0	0	23.08	21.5336	24.6264
ENSG00000253384	ENSG00000253384	CTD-2547L16.3	8:1791393	q1	q2	1.79769e+	1.79769e+	0	0	yes	0	0	0	0.0402212	0.0376786	0.0427637
ENSG00000253536	ENSG00000253536	RP11-1174L13.2	5:7075144	q1	q2	1.79769e+	1.79769e+	0	0	yes	0	0	0	0.0162079	0.0144042	0.0180115
ENSG00000253720	ENSG00000253720	RP11-473O4.3	8:1308445	q1	q2	1.79769e+	1.79769e+	0	0	yes	0	0	0	0.239696	0.214841	0.264552
ENSG00000254732	ENSG00000254732	RP11-691N7.6	11:574800	q1	q2	1.79769e+	1.79769e+	0	0	yes	0	0	0	0.143536	0.137538	0.149533
ENSG00000255421	ENSG00000255421	CTD-2011F17.2	11:755144	q1	q2	1.79769e+	1.79769e+	0	0	yes	0	0	0	0.12565	0.11324	0.138061
ENSG00000255448	ENSG00000255448	RP1-59M18.2	11:178095	q1	q2	1.79769e+	1.79769e+	0	0	yes	0	0	0	0.201051	0.173564	0.228538
ENSG00000255730	ENSG00000255730	CTC-435M10.3	19:418368	q1	q2	1.79769e+	1.79769e+	0	0	yes	0	0	0	5.07839	3.92024	6.23653
ENSG00000256159	ENSG00000256159	RP11-820K3.4	12:315351	q1	q2	1.79769e+	1.79769e+	0	0	yes	0	0	0	0.144323	0.0797796	0.208866
ENSG00000256175	ENSG00000256175	RP11-377D9.2	12:130806	q1	q2	1.79769e+	1.79769e+	0	0	yes	0	0	0	0.0278694	0.0258529	0.029886
ENSG00000256185	ENSG00000256185	RP11-612B6.2	12:262729	q1	q2	1.79769e+	1.79769e+	0	0	yes	0	0	0	1.64958	1.47642	1.82274
ENSG00000256268	ENSG00000256268	RP11-221N13.3	12:656724	q1	q2	1.79769e+	1.79769e+	0	0	yes	0	0	0	0.0814185	0.0481652	0.114672
ENSG00000256326	ENSG00000256326	RP11-467L13.3	12:318000	q1	q2	1.79769e+	1.79769e+	0	0	yes	0	0	0	0.257375	0.167768	0.346981
ENSG00000256682	ENSG00000256682	TAS2R12	12:109775	q1	q2	1.79769e+	1.79769e+	0	0	yes	0	0	0	0.0822529	0.06788	0.0966258
ENSG00000256861	ENSG00000256861	RP11-512M8.5	12:122688	q1	q2	1.79769e+	1.79769e+	0	0.002	yes	0	0	0	0.570437	0.263993	0.876881
ENSG00000256885	ENSG00000256885	AP001877.1	11:955021	q1	q2	1.79769e+	1.79769e+	0	0	yes	0	0	0	0.130862	0.112355	0.149369

ENSG00000256967	ENSG00000256967	RP11-273B20.1	12:728167	q1	q2	1.79769e+	1.79769e+	0	0	yes	0	0	0	0.116146	0.100993	0.131299
ENSG00000256973	ENSG00000256973	RP11-359J14.2	12:226015	q1	q2	1.79769e+	1.79769e+	0	0	yes	0	0	0	0.0724275	0.0648141	0.0800409
ENSG00000257264	ENSG00000257264	RP11-719K4.5	16:147664	q1	q2	1.79769e+	1.79769e+	0	0	yes	0	0	0	0.0214009	0.0120608	0.030741
ENSG00000257809	ENSG00000257809	RP11-603J24.14	12:565445	q1	q2	1.79769e+	1.79769e+	0	0.002	yes	0	0	0	4.30222	1.99248	6.61196
ENSG00000257894	ENSG00000257894	RP1-78O14.1	12:792577	q1	q2	1.79769e+	1.79769e+	0	0	yes	0	0	0	0.0910434	0.0795	0.102587
ENSG00000258064	ENSG00000258064	RP11-293I14.2	12:720032	q1	q2	1.79769e+	1.79769e+	0	0.001	yes	0	0	0	3.03563	1.54028	4.53099
ENSG00000258088	ENSG00000258088	RP11-114H23.2	12:759569	q1	q2	1.79769e+	1.79769e+	0	0	yes	0	0	0	0.173636	0.118028	0.229244
ENSG00000258212	ENSG00000258212	RP11-624G19.1	12:442297	q1	q2	1.79769e+	1.79769e+	0	0	yes	0	0	0	0.0288165	0.0198158	0.0378173
ENSG00000258521	ENSG00000258521	RP11-638I2.9	14:100800	q1	q2	1.79769e+	1.79769e+	0	0	yes	0	0	0	0.014045	0.0134036	0.0146864
ENSG00000258607	ENSG00000258607	RP11-701B16.3	14:598957	q1	q2	1.79769e+	1.79769e+	0	0	yes	0	0	0	0.202763	0.188108	0.217418
ENSG00000258663	ENSG00000258663	RP11-123M6.2	14:101245	q1	q2	1.79769e+	1.79769e+	0	0	yes	0	0	0	0.334716	0.198069	0.471363
ENSG00000259163	ENSG00000259163	RP11-1078H9.5	14:910069	q1	q2	1.79769e+	1.79769e+	0	0	yes	0	0	0	0.0453081	0.0410921	0.0495241
ENSG00000178685	ENSG00000178685	PARP10	8:1450513	q1	q2	0.125	-3.177	0.001	0.023	yes	224.396	211.961	236.831	254.238	240.068	268.408
ENSG00000257755	ENSG00000257755	AC074032.1	12:505057	q1	q2	0.134	-3.329	0.001	0.014	yes	0.298423	0.282075	0.31477	0.34119	0.321086	0.361295
ENSG00000064787	ENSG00000064787	BCAS1	20:525533	q1	q2	0.141	-3.355	0.001	0.013	yes	294.229	276.814	311.644	338.644	318.553	358.734
ENSG00000143384	ENSG00000143384	MCL1	1:1505470	q1	q2	0.151	-3.26	0.001	0.018	yes	228.819	213.931	243.707	266.016	248.557	283.475
ENSG00000013810	ENSG00000013810	TACC3	4:1723226	q1	q2	0.161	-3.438	0.001	0.01	yes	285.442	266.244	304.64	335.304	313.451	357.157
ENSG00000115657	ENSG00000115657	ABCB6	2:2200744	q1	q2	0.167	-3.38	0.001	0.012	yes	172.689	160.556	184.821	204.078	189.897	218.259
ENSG00000244005	ENSG00000244005	NFS1	20:342139	q1	q2	0.174	-3.266	0.001	0.017	yes	121.423	111.965	130.882	144.557	133.997	155.116
ENSG00000166333	ENSG00000166333	ILK	11:662487	q1	q2	0.181	-3.238	0.001	0.019	yes	126.573	117.032	136.114	151.726	139.167	164.286
ENSG00000110108	ENSG00000110108	TMEM109	11:606806	q1	q2	0.184	-3.193	0.001	0.022	yes	153.317	140.683	165.95	184.224	169.442	199.007
ENSG00000136045	ENSG00000136045	PWP1	12:108079	q1	q2	0.193	-3.295	0.001	0.016	yes	136.396	124.914	147.879	165.366	151.951	178.78
ENSG00000149716	ENSG00000149716	ORAOV1	11:694558	q1	q2	0.197	-3.472	0.001	0.009	yes	207.925	191.045	224.805	253.209	233.12	273.299
ENSG00000113269	ENSG00000113269	RNF130	5:1793459	q1	q2	0.2	-3.193	0.001	0.022	yes	129.465	118.046	140.885	158.135	144.067	172.204
ENSG00000102893	ENSG00000102893	PHKB	16:474952	q1	q2	0.204	-3.309	0.001	0.015	yes	16.8596	15.377	18.3421	20.6684	18.8872	22.4496
ENSG00000187608	ENSG00000187608	ISG15	1:948802-9	q1	q2	0.205	-3.252	0.001	0.018	yes	993.318	904.347	1082.29	1219.72	1111.14	1328.31
ENSG00000210082	ENSG00000210082	J01415.4	MT:1670-3	q1	q2	0.215	-3.314	0.001	0.015	yes	1755.43	1595.18	1915.69	2176.43	1975.81	2377.06
ENSG00000078140	ENSG00000078140	UBE2K	4:3969966	q1	q2	0.223	-3.38	0.001	0.012	yes	147.402	133.808	160.996	184.174	166.839	201.509
ENSG00000135709	ENSG00000135709	KIAA0513	16:850613	q1	q2	0.23	-3.219	0.001	0.02	yes	23.688	21.2967	26.0792	29.8096	26.7984	32.8208
ENSG00000113810	ENSG00000113810	SMC4	3:1599434	q1	q2	0.233	-3.182	0.001	0.022	yes	68.1595	60.8818	75.4373	86.0234	77.4221	94.6247
ENSG00000111237	ENSG00000111237	VPS29	12:110928	q1	q2	0.24	-3.393	0.001	0.012	yes	144.674	129.893	159.455	183.844	165.918	201.77
ENSG00000087470	ENSG00000087470	DNM1L	12:328321	q1	q2	0.254	-3.201	0.001	0.021	yes	80.8205	71.8224	89.8187	104.231	92.4055	116.056
ENSG00000091490	ENSG00000091490	SEL1L3	4:2574905	q1	q2	0.263	-3.424	0.001	0.011	yes	65.8289	58.3744	73.2834	85.6334	76.745	94.5217
ENSG00000116171	ENSG00000116171	SCP2	1:5339290	q1	q2	0.265	-3.462	0.001	0.009	yes	57.5847	51.2407	63.9286	75.0789	67.0793	83.0785
ENSG00000188313	ENSG00000188313	PLSCR1	3:1462329	q1	q2	0.269	-3.247	0.001	0.018	yes	139.233	122.264	156.202	182.262	161.75	202.774
ENSG00000197586	ENSG00000197586	ENTPD6	20:251763	q1	q2	0.269	-3.28	0.001	0.017	yes	112.274	103.19	121.357	146.962	125.972	167.952
ENSG00000084676	ENSG00000084676	NCOA1	2:2471478	q1	q2	0.272	-3.396	0.001	0.012	yes	21.4335	19.5263	23.3408	28.1363	24.3859	31.8867
ENSG00000102401	ENSG00000102401	ARMCX3	X:1008779	q1	q2	0.275	-3.41	0.001	0.011	yes	53.0341	46.8523	59.2159	69.7886	62.0348	77.5425
ENSG00000163083	ENSG00000163083	INHBB	2:1211037	q1	q2	0.275	-3.403	0.001	0.011	yes	28.7586	25.4083	32.109	37.8714	33.6203	42.1224
ENSG00000064652	ENSG00000064652	SNX24	5:1221791	q1	q2	0.276	-3.324	0.001	0.015	yes	176.224	155.264	197.184	232.321	205.332	259.309
ENSG00000176715	ENSG00000176715	ACSF3	16:891547	q1	q2	0.276	-3.427	0.001	0.011	yes	87.5129	78.5328	96.4929	115.325	101.01	129.639
ENSG00000095002	ENSG00000095002	MSH2	2:4763010	q1	q2	0.283	-3.479	0.001	0.009	yes	35.6669	31.8801	39.4537	47.3543	41.5017	53.2068
ENSG00000196455	ENSG00000196455	PIK3R4	3:1303977	q1	q2	0.284	-3.37	0.001	0.013	yes	61.9006	54.5689	69.2323	82.2019	72.3686	92.0351
ENSG00000174013	ENSG00000174013	FBXO45	3:1962810	q1	q2	0.291	-3.442	0.001	0.01	yes	10.5201	9.25054	11.7896	14.0714	12.4063	15.7364
ENSG00000214753	ENSG00000214753	HNRNPUL2	11:624577	q1	q2	0.301	-3.322	0.001	0.015	yes	45.3752	39.4713	51.2791	61.3393	53.5802	69.0984
ENSG00000109180	ENSG00000109180	OCIAD1	4:4880722	q1	q2	0.309	-3.332	0.001	0.014	yes	65.9231	57.926	73.9202	89.7808	77.194	102.368
ENSG00000244580	ENSG00000244580	AL121959.1	6:1335617	q1	q2	0.309	-3.317	0.001	0.015	yes	1076.11	930.479	1221.75	1466.26	1278.01	1654.51
ENSG00000145494	ENSG00000145494	NDUFS6	5:1801513	q1	q2	0.31	-3.353	0.001	0.013	yes	241.143	210.054	272.232	328.714	285.196	372.232
ENSG00000134824	ENSG00000134824	FADS2	11:615670	q1	q2	0.313	-3.363	0.001	0.013	yes	47.4931	41.3958	53.5904	64.9557	56.1956	73.7158
ENSG00000171163	ENSG00000171163	ZNF692	1:2491442	q1	q2	0.315	-3.409	0.001	0.011	yes	102.44	93.9129	110.966	140.347	117.205	163.489

ENSG00000166997	ENSG00000166997	CNPY4	7:9969035	q1	q2	0.316	-3.436	0.001	0.01	yes	29.4572	25.48	33.4343	40.4159	35.3578	45.4741
ENSG00000172590	ENSG00000172590	MRPL52	14:232990	q1	q2	0.323	-3.375	0.001	0.012	yes	471.286	405.117	537.455	651.091	566.303	735.879
ENSG00000139719	ENSG00000139719	VPS33A	12:122688	q1	q2	0.33	-3.238	0.001	0.019	yes	37.0257	31.4866	42.5648	51.4962	44.3713	58.6211
ENSG00000253729	ENSG00000253729	PRKDC	8:4868566	q1	q2	0.331	-3.444	0.001	0.01	yes	75.051	64.7528	85.3493	104.503	90.4319	118.575
ENSG00000094880	ENSG00000094880	CDC23	5:1374754	q1	q2	0.335	-3.384	0.001	0.012	yes	49.6849	42.4696	56.9003	69.4584	60.1107	78.8062
ENSG00000093009	ENSG00000093009	CDC45	22:194669	q1	q2	0.354	-3.216	0.001	0.02	yes	85.5283	70.3624	100.694	121.851	105.955	137.746
ENSG00000059378	ENSG00000059378	PARP12	7:1397235	q1	q2	0.358	-3.215	0.001	0.02	yes	62.8852	52.6466	73.1238	89.9415	76.2905	103.593
ENSG00000111615	ENSG00000111615	KRR1	12:758744	q1	q2	0.365	-3.29	0.001	0.016	yes	32.6967	26.9315	38.4619	47.0802	40.759	53.4014
ENSG00000108671	ENSG00000108671	PSMD11	17:307483	q1	q2	0.369	-3.358	0.001	0.013	yes	56.1784	49.0514	63.3053	81.2658	66.6711	95.8606
ENSG00000196353	ENSG00000196353	CPNE4	3:1312523	q1	q2	0.379	-3.276	0.001	0.017	yes	16.1883	13.2125	19.1641	23.6473	20.3251	26.9695
ENSG00000250656	ENSG00000250656	RP11-453E17.2	4:6856699	q1	q2	0.383	-3.274	0.001	0.017	yes	0.126267	0.105579	0.146955	0.185126	0.154272	0.21598
ENSG00000115816	ENSG00000115816	CEBPZ	2:3739496	q1	q2	0.384	-3.471	0.001	0.009	yes	18.4618	15.342	21.5815	27.102	23.2343	30.9698
ENSG00000251705	ENSG00000251705	RN5-8S6	Y:1003776	q1	q2	0.392	-3.335	0.001	0.014	yes	1693.66	1400.52	1986.8	2505.71	2107.86	2903.56
ENSG00000249242	ENSG00000249242	TMEM150C	4:8340574	q1	q2	0.395	-3.216	0.001	0.02	yes	65.8773	53.9955	77.7591	97.778	81.4822	114.074
ENSG00000049883	ENSG00000049883	PTCD2	5:7151523	q1	q2	0.405	-3.309	0.001	0.015	yes	8.0977	6.61253	9.58286	12.1373	10.173	14.1016
ENSG00000160087	ENSG00000160087	UBE2J2	1:1189288	q1	q2	0.438	-3.323	0.001	0.015	yes	57.2018	47.7364	66.6672	88.596	70.4482	106.744
ENSG00000130560	ENSG00000130560	UBAC1	9:1388248	q1	q2	0.439	-3.189	0.001	0.022	yes	55.7344	49.6659	61.8029	86.4445	64.5889	108.3
ENSG00000115977	ENSG00000115977	AAK1	2:6968641	q1	q2	0.455	-3.349	0.001	0.014	yes	17.0627	13.6788	20.4465	26.9015	21.8992	31.9039
ENSG00000252150	ENSG00000252150	AC092574.2	4:419223-4	q1	q2	0.455	-3.263	0.001	0.018	yes	5.62089	4.52381	6.71798	8.85783	7.09506	10.6206
ENSG00000142856	ENSG00000142856	ITGB3BP	1:6390644	q1	q2	0.461	-3.471	0.001	0.009	yes	18.4647	14.5286	22.4008	29.2818	24.6382	33.9254
ENSG00000088833	ENSG00000088833	NSFL1C	20:142280	q1	q2	0.465	-3.314	0.001	0.015	yes	27.9695	24.8098	31.1292	44.5098	33.0862	55.9335
ENSG00000243350	ENSG00000243350	RP11-379F12.3	10:809241	q1	q2	0.485	-3.394	0.001	0.012	yes	19.0644	15.0189	23.1099	30.9607	25.038	36.8834
ENSG00000174516	ENSG00000174516	PELI3	11:662342	q1	q2	0.489	-3.321	0.001	0.015	yes	18.4362	14.6056	22.2668	30.0587	23.7888	36.3287
ENSG00000185112	ENSG00000185112	FAM43A	3:1944066	q1	q2	0.492	-3.266	0.001	0.017	yes	2.83444	2.19475	3.47413	4.63485	3.71107	5.55863
ENSG00000233937	ENSG00000233937	CTC-338M12.4	5:1806639	q1	q2	0.497	-3.391	0.001	0.012	yes	14.4399	11.1278	17.752	23.7372	19.4039	28.0705
ENSG00000004399	ENSG00000004399	PLXND1	3:1292740	q1	q2	0.508	-3.179	0.001	0.023	yes	32.299	25.8773	38.7207	53.7041	40.2448	67.1633
ENSG00000215039	ENSG00000215039	AC005840.1	12:654816	q1	q2	0.535	-3.343	0.001	0.014	yes	16.6664	12.3825	20.9503	28.4559	23.0317	33.8802
ENSG00000112739	ENSG00000112739	PRPF4B	6:4021500	q1	q2	0.538	-3.353	0.001	0.013	yes	9.88317	7.39999	12.3664	16.9245	13.5464	20.3027
ENSG00000084112	ENSG00000084112	SSH1	12:109176	q1	q2	0.54	-3.407	0.001	0.011	yes	20.2222	14.4498	25.9946	34.6987	29.9197	39.4778
ENSG00000103550	ENSG00000103550	C16orf88	16:197176	q1	q2	0.543	-3.386	0.001	0.012	yes	3.84481	2.91397	4.77565	6.61519	5.22614	8.00424
ENSG00000255773	ENSG00000255773	RP11-566K11.1	16:899785	q1	q2	0.566	-3.398	0.001	0.012	yes	2.61618	1.9544	3.27795	4.6087	3.60881	5.60859
ENSG00000256929	ENSG00000256929	AC067852.1	17:406108	q1	q2	0.586	-3.282	0.001	0.017	yes	38.524	27.9128	49.1351	69.2497	53.4821	85.0173
ENSG00000109625	ENSG00000109625	CPZ	4:8560451	q1	q2	0.6	-3.477	0.001	0.009	yes	6.25615	4.67544	7.83685	11.4043	8.71955	14.089
ENSG00000147099	ENSG00000147099	HDAC8	X:7154936	q1	q2	0.623	-3.237	0.001	0.019	yes	11.4959	8.16216	14.8296	21.4428	16.0067	26.8789
ENSG00000171723	ENSG00000171723	GPHN	14:666795	q1	q2	0.644	-3.28	0.001	0.017	yes	27.8122	20.8328	34.7916	52.9341	36.9662	68.9021
ENSG00000139350	ENSG00000139350	NEDD1	12:973010	q1	q2	0.649	-3.474	0.001	0.009	yes	20.5732	15.777	25.3694	39.389	27.8762	50.9018
ENSG00000224680	ENSG00000224680	PLA2G12AP1	1:5283434	q1	q2	0.661	-3.455	0.001	0.01	yes	11.4744	8.01473	14.934	22.2267	16.9884	27.465
ENSG00000092470	ENSG00000092470	WDR76	15:441191	q1	q2	0.667	-3.214	0.001	0.02	yes	8.11013	5.4455	10.7747	15.8003	11.7939	19.8067
ENSG00000204622	ENSG00000204622	HLA-J	6:2996878	q1	q2	0.671	-3.307	0.001	0.015	yes	3.93271	2.6508	5.21462	7.69152	5.83425	9.54879
ENSG00000245750	ENSG00000245750	AC100826.1	15:698540	q1	q2	0.672	-3.337	0.001	0.014	yes	2.45635	1.66969	3.24301	4.81041	3.63528	5.98553
ENSG00000258376	ENSG00000258376	RP4-647C14.2	14:737042	q1	q2	0.689	-3.299	0.001	0.016	yes	0.0245322	0.0171355	0.031929	0.0488407	0.0347416	0.0629397
ENSG00000134255	ENSG00000134255	CEPT1	1:1116599	q1	q2	0.705	-3.308	0.001	0.015	yes	4.01862	2.71022	5.32702	8.13415	5.89506	10.3732
ENSG00000182472	ENSG00000182472	CAPN12	19:391383	q1	q2	0.788	-3.25	0.001	0.018	yes	0.827716	0.500487	1.15494	1.81952	1.30908	2.32995
ENSG00000236646	ENSG00000236646	HBXIPP1	1:6220814	q1	q2	0.813	-3.296	0.001	0.016	yes	30.5127	18.1919	42.8334	68.7749	49.3139	88.2359
ENSG00000164180	ENSG00000164180	TMEM161B	5:8748544	q1	q2	0.842	-3.3	0.001	0.016	yes	3.86704	2.13047	5.60361	8.97132	6.8022	11.1404
ENSG00000136521	ENSG00000136521	NDUFB5	3:1793224	q1	q2	0.853	-3.446	0.001	0.01	yes	88.9572	55.6625	122.252	208.759	141.121	276.397
ENSG00000196085	ENSG00000196085	AC013442.1	2:3544444	q1	q2	0.886	-3.363	0.001	0.013	yes	43.6628	24.6068	62.7189	105.866	74.6481	137.085
ENSG00000148814	ENSG00000148814	LRRC27	10:134145	q1	q2	0.887	-3.248	0.001	0.018	yes	1.90709	1.21051	2.60367	4.62778	2.75053	6.50504
ENSG00000135926	ENSG00000135926	TMBIM1	2:2191351	q1	q2	0.966	-3.477	0.001	0.009	yes	3.37497	1.57937	5.17058	8.8679	7.44772	10.2881
ENSG00000099953	ENSG00000099953	MMP11	22:241052	q1	q2	1.013	-3.234	0.001	0.019	yes	4.52318	1.98046	7.0659	12.454	9.01495	15.8931

ENSG00000132010	ENSG00000132010	ZNF20	19:122412	q1	q2	1.044	-3.209	0.001	0.021	yes	3.0946	1.42254	4.76665	8.78705	5.60395	11.9701
ENSG00000103021	ENSG00000103021	CCDC113	16:582838	q1	q2	1.161	-3.353	0.001	0.013	yes	0.23963	0.1038	0.375461	0.765019	0.460883	1.06916
ENSG00000240137	ENSG00000240137	RP11-103G8.2	3:1503776	q1	q2	1.196	-3.345	0.001	0.014	yes	0.133186	0.0655928	0.200778	0.440454	0.218543	0.662364
ENSG00000138100	ENSG00000138100	TRIM54	2:2750525	q1	q2	1.237	-3.274	0.001	0.017	yes	0.0642617	0.0625838	0.0659396	0.221304	0.0542441	0.388364
ENSG00000224985	ENSG00000224985	RP11-297K8.2	1:1611225	q1	q2	1.418	-3.249	0.001	0.018	yes	3.5571	0.800354	6.31385	14.681	8.78875	20.5732
ENSG00000131773	ENSG00000131773	KHDRBS3	8:1364696	q1	q2	1.439	-3.459	0.001	0.01	yes	5.47737	3.38813	7.56661	23.1048	6.01121	40.1983
ENSG00000134864	ENSG00000134864	A2LD1	13:101183	q1	q2	1.54	-3.346	0.001	0.014	yes	3.50407	0.718161	6.28998	16.3507	8.76113	23.9402
ENSG00000111752	ENSG00000111752	PHC1	12:906649	q1	q2	1.612	-3.464	0.001	0.009	yes	0.323654	0.297687	0.349622	1.62279	0.117965	3.12761
ENSG00000258660	ENSG00000258660	RP4-693M11.3	14:741115	q1	q2	1.74	-3.334	0.001	0.014	yes	0.143284	0.113568	0.173	0.816034	0	1.65065
ENSG00000185038	ENSG00000185038	HEATR7B1	2:2346843	q1	q2	1.751	-3.423	0.001	0.011	yes	0.159657	0.0727153	0.2466	0.919875	0.123066	1.71668
ENSG00000189410	ENSG00000189410	SH2D5	1:2104622	q1	q2	1.931	-3.28	0.001	0.017	yes	1.49087	0	3.15306	10.282	6.38599	14.178
ENSG00000248121	ENSG00000248121	AC005562.2	17:289035	q1	q2	2.15	-3.225	0.001	0.02	yes	0.705975	0	1.52117	6.06079	2.02026	10.1013
ENSG00000116652	ENSG00000116652	DLEU2L	1:6390644	q1	q2	2.346	-3.47	0.001	0.009	yes	0.0614739	0.0554019	0.0675459	0.642154	0	1.50817
ENSG00000202031	ENSG00000202031	SNORD38A	1:4520548	q1	q2	2.655	-3.255	0.001	0.018	yes	16.9005	15.9587	17.8423	240.321	0	632.11
ENSG00000243444	ENSG00000243444	PALM2	9:1124030	q1	q2	2.691	-3.377	0.001	0.012	yes	0.124197	0	0.314199	1.83101	1.01313	2.64889
ENSG00000224078	ENSG00000224078	UBE3A-AS1	15:250687	q1	q2	3.049	-3.344	0.001	0.014	yes	0.0290516	0	0.0752956	0.61306	0.0670839	1.15904
ENSG00000023171	ENSG00000023171	GRAMD1B	11:123396	q1	q2	3.23	-3.323	0.001	0.015	yes	0.0654802	0	0.184322	1.65459	0.504372	2.8048
ENSG00000156150	ENSG00000156150	ALX3	1:1105741	q1	q2	1.79769e+	1.79769e+	0.001	0.01	yes	0	0	0	0.705794	0.271581	1.14001
ENSG00000168484	ENSG00000168484	SFTPC	8:2200433	q1	q2	1.79769e+	1.79769e+	0.001	0.013	yes	0	0	0	0.021238	0.0077774	0.0346987
ENSG00000240801	ENSG00000240801	AC132217.4	11:215034	q1	q2	1.79769e+	1.79769e+	0.001	0.022	yes	0	0	0	5.45417	1.80116	9.10718
ENSG00000242588	ENSG00000242588	RP11-274B21.1	7:1281737	q1	q2	1.79769e+	1.79769e+	0.001	0.013	yes	0	0	0	1.67118	0.614234	2.72813
ENSG00000244684	ENSG00000244684	AL358786.1	9:7314994	q1	q2	1.79769e+	1.79769e+	0.001	0.013	yes	0	0	0	9.52941	3.50248	15.5563
ENSG00000249199	ENSG00000249199	CTD-2139B15.5	5:1736933	q1	q2	1.79769e+	1.79769e+	0.001	0.021	yes	0	0	0	10.6399	3.54663	17.7331
ENSG00000258227	ENSG00000258227	CLEC5A	7:1416076	q1	q2	1.79769e+	1.79769e+	0.001	0.021	yes	0	0	0	0.0452927	0.0150558	0.0755295
ENSG00000259046	ENSG00000259046	RP11-857B24.2	14:453460	q1	q2	1.79769e+	1.79769e+	0.001	0.018	yes	0	0	0	3.44074	1.18732	5.69415
ENSG00000143321	ENSG00000143321	HDGF	1:1567118	q1	q2	0.142	-3.114	0.002	0.027	yes	272.167	254.492	289.842	313.813	293.606	334.02
ENSG00000048828	ENSG00000048828	FAM120A	9:9620877	q1	q2	0.149	-3.061	0.002	0.032	yes	116.273	108.296	124.25	134.921	125.632	144.21
ENSG00000159176	ENSG00000159176	CSRP1	1:2014526	q1	q2	0.15	-3.067	0.002	0.031	yes	396.123	370.637	421.61	460.004	426.31	493.698
ENSG00000223501	ENSG00000223501	VPS52	6:3321731	q1	q2	0.153	-3.131	0.002	0.026	yes	189.354	176.527	202.181	220.703	205.118	236.288
ENSG00000129084	ENSG00000129084	PSMA1	11:144649	q1	q2	0.17	-3.086	0.002	0.03	yes	147.99	136.407	159.572	175.413	161.807	189.019
ENSG00000187741	ENSG00000187741	FANCA	16:897735	q1	q2	0.184	-3.132	0.002	0.026	yes	63.0697	57.8647	68.2746	75.7828	69.4691	82.0965
ENSG00000119723	ENSG00000119723	COQ6	14:743185	q1	q2	0.189	-3.063	0.002	0.032	yes	111.589	101.137	122.04	134.773	123.972	145.574
ENSG00000100811	ENSG00000100811	YY1	14:100657	q1	q2	0.192	-3.064	0.002	0.032	yes	184.616	168.249	200.982	223.707	203.874	243.541
ENSG00000140525	ENSG00000140525	FANCI	15:897871	q1	q2	0.192	-3.04	0.002	0.034	yes	32.8374	29.8671	35.8077	39.7755	36.281	43.2701
ENSG00000103018	ENSG00000103018	CYB5B	16:694584	q1	q2	0.195	-3.131	0.002	0.026	yes	46.4101	42.4256	50.3947	56.4001	51.3123	61.4879
ENSG00000140943	ENSG00000140943	MBTPS1	16:840873	q1	q2	0.197	-3.059	0.002	0.032	yes	89.686	81.4748	97.8972	109.229	99.323	119.134
ENSG00000158089	ENSG00000158089	GALNT14	2:3113333	q1	q2	0.197	-3.081	0.002	0.03	yes	81.0252	73.7966	88.2538	98.6571	89.6263	107.688
ENSG00000136492	ENSG00000136492	BRIP1	17:597599	q1	q2	0.212	-3.068	0.002	0.031	yes	47.9289	43.1959	52.6618	59.2755	53.5165	65.0344
ENSG00000213780	ENSG00000213780	GTF2H4	6:3087596	q1	q2	0.227	-3.082	0.002	0.03	yes	64.8203	57.3497	72.2909	81.3047	73.879	88.7304
ENSG00000177119	ENSG00000177119	ANO6	12:455668	q1	q2	0.243	-3.032	0.002	0.034	yes	9.61163	8.46557	10.7577	12.2563	10.9428	13.5698
ENSG00000102024	ENSG00000102024	PLS3	X:1147524	q1	q2	0.244	-3.066	0.002	0.031	yes	110.237	100.822	119.652	140.691	121.806	159.576
ENSG00000120533	ENSG00000120533	ENY2	8:1102531	q1	q2	0.259	-3.169	0.002	0.023	yes	123.399	109.044	137.755	159.9	141.517	178.284
ENSG00000197114	ENSG00000197114	ZGPAT	20:622891	q1	q2	0.26	-3.056	0.002	0.032	yes	51.4699	45.3408	57.599	66.7558	58.64	74.8716
ENSG00000179195	ENSG00000179195	ZNF664	12:124247	q1	q2	0.273	-3.026	0.002	0.035	yes	174.561	152.214	196.909	229.408	200.191	258.625
ENSG00000143164	ENSG00000143164	DCAF6	1:1678859	q1	q2	0.277	-3.104	0.002	0.028	yes	38.3881	33.6964	43.0797	50.6358	44.0537	57.2179
ENSG00000112305	ENSG00000112305	SMAP1	6:7137747	q1	q2	0.289	-3.07	0.002	0.031	yes	23.6893	20.4453	26.9333	31.6185	27.5414	35.6956
ENSG00000114383	ENSG00000114383	TUSC2	3:5035522	q1	q2	0.295	-3.115	0.002	0.027	yes	52.3749	44.6459	60.1038	70.3575	61.9948	78.7202
ENSG00000114302	ENSG00000114302	PRKAR2A	3:4878202	q1	q2	0.297	-3.162	0.002	0.024	yes	39.8494	34.2344	45.4644	53.6449	46.9669	60.323
ENSG00000185507	ENSG00000185507	IRF7	11:612552	q1	q2	0.297	-3.088	0.002	0.03	yes	43.009	38.5002	47.5177	57.8852	48.5451	67.2253
ENSG00000065150	ENSG00000065150	IPO5	13:986057	q1	q2	0.306	-3.049	0.002	0.033	yes	92.0324	75.6076	108.457	125.032	113.472	136.591

ENSG00000159079	ENSG00000159079	C21orf59	21:339488	q1	q2	0.31	-3.128	0.002	0.026	yes	36.8183	32.1694	41.4672	50.2085	42.5302	57.8869
ENSG00000078304	ENSG00000078304	PPP2R5C	14:102228	q1	q2	0.312	-3.17	0.002	0.023	yes	88.8272	75.4468	102.208	121.343	105.978	136.709
ENSG00000198894	ENSG00000198894	KIAA1737	14:775644	q1	q2	0.318	-3.05	0.002	0.033	yes	28.5135	24.1684	32.8585	39.1956	33.6096	44.7817
ENSG00000143499	ENSG00000143499	SMYD2	1:2144544	q1	q2	0.348	-3.146	0.002	0.025	yes	26.9843	22.4332	31.5354	38.2199	32.7441	43.6956
ENSG00000157800	ENSG00000157800	SLC37A3	7:1399934	q1	q2	0.356	-3.147	0.002	0.025	yes	20.5889	17.2	23.9777	29.3848	24.8303	33.9393
ENSG00000197989	ENSG00000197989	SNHG12	1:2887959	q1	q2	0.369	-3.049	0.002	0.033	yes	29.9983	24.921	35.0755	43.4012	35.8799	50.9224
ENSG00000120705	ENSG00000120705	ETF1	5:1378417	q1	q2	0.378	-3.054	0.002	0.032	yes	28.4102	23.5853	33.2351	41.4408	33.9974	48.8841
ENSG00000214167	ENSG00000214167	AC005544.1	17:649610	q1	q2	0.403	-3.155	0.002	0.024	yes	32.6172	26.4604	38.774	48.7998	40.4041	57.1955
ENSG00000121749	ENSG00000121749	TBC1D15	12:722334	q1	q2	0.428	-3.049	0.002	0.033	yes	35.713	27.5301	43.896	54.8058	45.8977	63.7139
ENSG00000155957	ENSG00000155957	TMBIM4	12:665168	q1	q2	0.442	-3.15	0.002	0.025	yes	134.892	107.102	162.682	209.934	169.859	250.01
ENSG00000152348	ENSG00000152348	ATG10	5:8126784	q1	q2	0.443	-3.132	0.002	0.026	yes	20.3058	16.3403	24.2712	31.6351	25.1516	38.1186
ENSG00000259141	ENSG00000259141	AC027323.1	5:8981142	q1	q2	0.451	-3.069	0.002	0.031	yes	2.25978	1.76534	2.75422	3.54646	2.85175	4.24116
ENSG00000180098	ENSG00000180098	TRNAU1AP	1:2887959	q1	q2	0.456	-3.066	0.002	0.031	yes	14.6283	11.3715	17.8851	23.0783	18.527	27.6297
ENSG00000129465	ENSG00000129465	RIPK3	14:247875	q1	q2	0.459	-3.124	0.002	0.027	yes	14.0829	10.9622	17.2035	22.285	17.9861	26.584
ENSG00000241404	ENSG00000241404	EGFL8	6:3211613	q1	q2	0.472	-3.065	0.002	0.031	yes	19.3918	14.7643	24.0193	31.094	25.0338	37.1541
ENSG00000073614	ENSG00000073614	KDM5A	12:389222	q1	q2	0.486	-3.108	0.002	0.028	yes	18.9488	14.5525	23.345	30.8014	24.3476	37.2551
ENSG00000132330	ENSG00000132330	SCLY	2:2388754	q1	q2	0.489	-3.155	0.002	0.024	yes	14.1079	10.7553	17.4606	22.9958	18.4271	27.5646
ENSG00000254452	ENSG00000254452	RP11-867G23.4	11:660360	q1	q2	0.521	-3.136	0.002	0.026	yes	24.177	18.1259	30.228	40.7022	31.8142	49.5901
ENSG00000150054	ENSG00000150054	MPP7	10:283399	q1	q2	0.563	-3.068	0.002	0.031	yes	11.9966	8.0226	15.9707	21.0752	17.7298	24.4206
ENSG00000233719	ENSG00000233719	GOT2P3	12:979439	q1	q2	0.65	-3.145	0.002	0.025	yes	3.48861	2.34156	4.63565	6.67941	5.00956	8.34926
ENSG00000113593	ENSG00000113593	PPWD1	5:6485906	q1	q2	0.667	-3.172	0.002	0.023	yes	14.9605	11.1925	18.7286	29.1492	19.3328	38.9656
ENSG00000119608	ENSG00000119608	PROX2	14:752300	q1	q2	0.681	-3.043	0.002	0.033	yes	1.45748	0.940709	1.97426	2.87867	2.09364	3.6637
ENSG00000163590	ENSG00000163590	PPM1L	3:1604733	q1	q2	0.774	-3.121	0.002	0.027	yes	5.34438	2.84372	7.84505	11.5836	9.68874	13.4784
ENSG00000058091	ENSG00000058091	CDK14	7:8996453	q1	q2	0.831	-3.095	0.002	0.029	yes	6.90802	4.17692	9.63913	15.8541	10.0981	21.6101
ENSG00000230530	ENSG00000230530	RP11-697K23.1	3:4559688	q1	q2	0.869	-3.105	0.002	0.028	yes	2.05525	1.10269	3.00782	4.89924	3.36337	6.43511
ENSG00000184304	ENSG00000184304	PRKD1	14:300456	q1	q2	0.906	-3.078	0.002	0.03	yes	5.27695	2.83167	7.72222	13.0593	8.31711	17.8015
ENSG00000239557	ENSG00000239557	RP11-168J18.6	3:5235033	q1	q2	0.912	-3.056	0.002	0.032	yes	1.49152	0.750671	2.23236	3.71347	2.4836	4.94334
ENSG00000108010	ENSG00000108010	GLRX3	10:131934	q1	q2	0.996	-3.084	0.002	0.03	yes	16.3147	10.0659	22.5634	44.1889	21.1905	67.1874
ENSG00000223960	ENSG00000223960	AC009948.5	2:1792786	q1	q2	1.012	-3.125	0.002	0.027	yes	3.03556	1.39076	4.68035	8.35466	5.38761	11.3217
ENSG00000247670	ENSG00000247670	AL078611.1	22:466399	q1	q2	1.038	-3.07	0.002	0.031	yes	0.636324	0.271669	1.00098	1.79644	1.15182	2.44106
ENSG00000236383	ENSG00000236383	AC109326.1	17:413638	q1	q2	1.076	-3.035	0.002	0.034	yes	1.404	0.556098	2.2519	4.11615	2.58955	5.64275
ENSG00000216895	ENSG00000216895	AC009403.2	7:1554039	q1	q2	1.131	-3.052	0.002	0.033	yes	17.1885	6.55454	27.8225	53.2382	31.5421	74.9344
ENSG00000232220	ENSG00000232220	AC008440.5	19:543578	q1	q2	1.145	-3.155	0.002	0.024	yes	4.39759	1.65271	7.14246	13.82	8.70052	18.9394
ENSG00000117122	ENSG00000117122	MFAP2	1:1730099	q1	q2	1.183	-3.117	0.002	0.027	yes	1.98721	0.577358	3.39707	6.48855	4.73224	8.24485
ENSG00000185513	ENSG00000185513	L3MBTL1	20:421363	q1	q2	1.196	-3.142	0.002	0.025	yes	3.32315	1.1349	5.5114	10.9875	6.7937	15.1812
ENSG00000258813	ENSG00000258813	RP11-109N23.4	14:735251	q1	q2	1.294	-3.146	0.002	0.025	yes	2.49676	0.697396	4.29612	9.10969	5.49363	12.7257
ENSG00000205794	ENSG00000205794	RP11-333E13.4	4:4004453	q1	q2	1.343	-3.097	0.002	0.029	yes	0.91821	0.222576	1.61384	3.51685	2.03249	5.00121
ENSG00000256309	ENSG00000256309	AC009469.1	2:2186645	q1	q2	1.531	-3.102	0.002	0.028	yes	0.381027	0.0431749	0.718878	1.7617	0.99739	2.526
ENSG00000255692	ENSG00000255692	RP1-127H14.3	12:120123	q1	q2	1.653	-3.078	0.002	0.03	yes	0.280688	0.260951	0.300424	1.4658	0	3.03657
ENSG00000246731	ENSG00000246731	CTD-2514K5.2	17:721997	q1	q2	1.688	-3.078	0.002	0.03	yes	0.273244	0.0604651	0.486023	1.47799	0.336438	2.61955
ENSG00000233611	ENSG00000233611	AC079135.1	2:2370738	q1	q2	1.696	-3.166	0.002	0.024	yes	0.208531	0.146583	0.27048	1.13653	0	2.30613
ENSG00000214846	ENSG00000214846	RP11-115L11.1	4:1560616	q1	q2	1.948	-3.054	0.002	0.032	yes	0.0794909	0.0724922	0.0864897	0.557711	0	1.26749
ENSG00000169282	ENSG00000169282	KCNAB1	3:1557554	q1	q2	2.253	-3.095	0.002	0.029	yes	0.185751	0	0.450472	1.76764	1.23977	2.29552
ENSG00000174093	ENSG00000174093	RP11-1407O15.2	17:363377	q1	q2	3.271	-3.066	0.002	0.031	yes	0.0676498	0	0.208292	1.78251	0.922683	2.64233
ENSG00000178550	ENSG00000178550	AC010170.1	3:4526595	q1	q2	3.448	-3.106	0.002	0.028	yes	0.0217522	0	0.0652566	0.68391	0.024383	1.34344
ENSG00000237669	ENSG00000237669	HCG4P3	6:2996878	q1	q2	1.79769e+	1.79769e+	0.002	0.031	yes	0	0	0	1.07356	0.320764	1.82636
ENSG00000249055	ENSG00000249055	RP11-428B4.2	4:9979283	q1	q2	1.79769e+	1.79769e+	0.002	0.029	yes	0	0	0	7.70701	2.35337	13.0607
ENSG00000255467	ENSG00000255467	RP11-144G7.2	11:107879	q1	q2	1.79769e+	1.79769e+	0.002	0.025	yes	0	0	0	1.19239	0.382536	2.00225
ENSG00000181789	ENSG00000181789	COPG	3:1289684	q1	q2	0.139	-2.968	0.003	0.042	yes	370.861	346.017	395.705	426.086	398.268	453.904
ENSG00000007384	ENSG00000007384	RHBDF1	16:108057	q1	q2	0.163	-3.023	0.003	0.035	yes	225.838	212.037	239.64	265.828	242.199	289.458

ENSG00000143878	ENSG00000143878	RHOB	2:2064683	q1	q2	0.187	-2.997	0.003	0.038	yes	303.327	276.514	330.14	365.725	333.485	397.964
ENSG00000024048	ENSG00000024048	UBR2	6:4253179	q1	q2	0.206	-2.922	0.003	0.047	yes	14.0846	12.7468	15.4224	17.3006	15.5035	19.0977
ENSG00000241343	ENSG00000241343	RPL36A	X:1006458	q1	q2	0.22	-2.965	0.003	0.042	yes	294.954	262.174	327.734	367.486	331.393	403.579
ENSG00000131747	ENSG00000131747	TOP2A	17:385447	q1	q2	0.226	-2.967	0.003	0.042	yes	35.6991	31.6903	39.7079	44.74	40.1469	49.3331
ENSG00000053770	ENSG00000053770	MUDENG	14:576705	q1	q2	0.25	-3	0.003	0.038	yes	42.9857	37.8569	48.1146	55.189	48.7733	61.6047
ENSG00000119396	ENSG00000119396	RAB14	9:1239404	q1	q2	0.256	-3.003	0.003	0.038	yes	39.3574	34.4667	44.2481	50.834	44.9053	56.7627
ENSG00000168092	ENSG00000168092	PAFAH1B2	11:117014	q1	q2	0.332	-2.921	0.003	0.047	yes	26.3637	21.5863	31.1411	36.7409	31.7021	41.7797
ENSG00000168807	ENSG00000168807	SNTB2	16:692210	q1	q2	0.373	-3.018	0.003	0.036	yes	96.1069	75.3998	116.814	139.49	122.678	156.303
ENSG00000133111	ENSG00000133111	RFXAP	13:373933	q1	q2	0.383	-3.003	0.003	0.038	yes	7.07051	5.74198	8.39905	10.3687	8.58191	12.1554
ENSG00000126368	ENSG00000126368	NR1D1	17:382184	q1	q2	0.384	-3.022	0.003	0.035	yes	5.84297	4.7486	6.93735	8.57788	7.10483	10.0509
ENSG00000148634	ENSG00000148634	HERC4	10:696816	q1	q2	0.414	-3.021	0.003	0.036	yes	7.80745	6.13902	9.47587	11.8123	9.78362	13.8409
ENSG00000132849	ENSG00000132849	INADL	1:6220814	q1	q2	0.438	-3.019	0.003	0.036	yes	16.6901	14.1424	19.2378	25.8532	19.4822	32.2242
ENSG00000249042	ENSG00000249042	CTD-2015H6.3	5:7977811	q1	q2	0.458	-2.95	0.003	0.044	yes	10.3083	7.86989	12.7467	16.2896	13.0211	19.558
ENSG00000176208	ENSG00000176208	ATAD5	17:291589	q1	q2	0.502	-2.953	0.003	0.043	yes	0.950534	0.707127	1.19394	1.56959	1.21917	1.92002
ENSG00000151164	ENSG00000151164	RAD9B	12:110928	q1	q2	0.627	-3.016	0.003	0.036	yes	6.08523	4.17806	7.9924	11.3935	8.27876	14.5082
ENSG00000205268	ENSG00000205268	PDE7A	8:6655612	q1	q2	0.675	-2.96	0.003	0.043	yes	3.13399	2.00863	4.25935	6.15482	4.42456	7.88507
ENSG00000114786	ENSG00000114786	RP11-155D18.11	3:5198932	q1	q2	0.747	-2.976	0.003	0.041	yes	2.27331	1.31773	3.22889	4.79643	3.48287	6.10999
ENSG00000175455	ENSG00000175455	CCDC14	3:1236161	q1	q2	0.751	-2.967	0.003	0.042	yes	30.0782	25.2979	34.8584	63.7198	33.1058	94.3338
ENSG00000177721	ENSG00000177721	C5orf39	5:4301451	q1	q2	0.897	-3.012	0.003	0.037	yes	0.763014	0.38594	1.14009	1.87035	1.24923	2.49147
ENSG00000100154	ENSG00000100154	TTC28	22:282024	q1	q2	0.987	-2.969	0.003	0.042	yes	1.51693	1.027	2.00687	4.07189	1.70372	6.44005
ENSG00000258802	ENSG00000258802	RP11-589M4.2	14:532595	q1	q2	1.2	-2.94	0.003	0.045	yes	57.6513	16.8857	98.417	191.443	113.286	269.599
ENSG00000152457	ENSG00000152457	DCLRE1C	10:149208	q1	q2	1.306	-2.962	0.003	0.042	yes	7.43593	3.00021	11.8717	27.4598	9.61687	45.3028
ENSG00000254400	ENSG00000254400	RP11-732A19.8	11:662487	q1	q2	1.313	-2.946	0.003	0.044	yes	3.13565	0.681133	5.59016	11.6618	6.68167	16.6419
ENSG00000166670	ENSG00000166670	MMP10	11:102617	q1	q2	1.371	-2.999	0.003	0.038	yes	0.0774686	0.0306329	0.124304	0.305297	0.0957519	0.514842
ENSG00000147874	ENSG00000147874	HAUS6	9:1905314	q1	q2	1.374	-2.945	0.003	0.044	yes	0.377423	0.170295	0.584552	1.4911	0.365717	2.61648
ENSG00000163806	ENSG00000163806	SPDYA	2:2897450	q1	q2	1.642	-2.952	0.003	0.043	yes	0.64241	0	1.29512	3.31683	1.81807	4.81559
ENSG00000221817	ENSG00000221817	RP11-137L10.6	10:751961	q1	q2	1.703	-2.944	0.003	0.044	yes	0.539028	0	1.11111	2.95909	1.59576	4.32242
ENSG00000230534	ENSG00000230534	RP11-297A16.2	10:353869	q1	q2	2.148	-2.995	0.003	0.038	yes	0.0762267	0.0643221	0.0881313	0.652893	0	1.58363
ENSG00000258283	ENSG00000258283	RP11-386G11.3	12:493889	q1	q2	2.413	-2.921	0.003	0.047	yes	0.164822	0.160483	0.169161	1.83978	0	4.87793
ENSG00000213760	ENSG00000213760	ATP6V1G2	6:3149649	q1	q2	2.671	-2.997	0.003	0.038	yes	0.130485	0	0.346486	1.88606	0.639198	3.13292
ENSG00000100926	ENSG00000100926	TM9SF1	14:246583	q1	q2	0.137	-2.913	0.004	0.048	yes	146.088	136.363	155.814	167.459	156.409	178.509
ENSG00000151465	ENSG00000151465	CDC123	10:121716	q1	q2	0.226	-2.907	0.004	0.049	yes	158.035	140.092	175.979	198.105	177.058	219.152
ENSG00000006607	ENSG00000006607	FARP2	2:2422956	q1	q2	0.264	-2.919	0.004	0.048	yes	77.2092	67.3943	87.0241	100.576	87.5961	113.556
ENSG00000183773	ENSG00000183773	AIFM3	22:213193	q1	q2	0.355	-2.918	0.004	0.048	yes	21.1133	17.6139	24.6128	30.1014	24.7474	35.4554
ENSG00000115504	ENSG00000115504	EHBP1	2:6290098	q1	q2	0.441	-2.918	0.004	0.048	yes	30.4733	24.4332	36.5134	47.384	36.5549	58.2131
ENSG00000072121	ENSG00000072121	ZFYVE26	14:681686	q1	q2	0.582	-2.916	0.004	0.048	yes	25.8896	17.3625	34.4168	46.3274	35.8857	56.7692
ENSG00000137098	ENSG00000137098	SPAG8	9:3579215	q1	q2	0.709	-2.913	0.004	0.048	yes	3.44314	2.24864	4.63764	6.99935	4.60659	9.3921
ENSG00000229759	ENSG00000229759	MRPS18AP1	3:4828258	q1	q2	0.868	-2.901	0.004	0.05	yes	3.99782	2.02004	5.9756	9.52725	6.31504	12.7395
ENSG00000176533	ENSG00000176533	GNG7	19:251121	q1	q2	2.285	-2.908	0.004	0.049	yes	0.0421936	0	0.105049	0.414573	0.207286	0.621859
ENSG00000254872	ENSG00000254872	RP13-870H17.3	11:104987	q1	q2	2.742	-2.911	0.004	0.049	yes	0.0644629	0	0.155627	1.00028	0	2.24472
ENSG00000228275	ENSG00000228275	ARMCX3-AS1	X:1008779	q1	q2	1.79769e+	1.79769e+	0.004	0.05	yes	0	0	0	2.60848	0.660104	4.55686
ENSG00000227962	ENSG00000227962	RP11-382D8.3	1:2352944	q1	q2	1.79769e+	1.79769e+	8.6493300	9.6179000	yes	0	0	0	0.43591	0.412839	0.458981
