# Supplementary figures and images for: Prosaposin activates the androgen receptor and potentiates resistance to endocrine treatment in breast cancer
Source: Breast Cancer Res. 2015 Sep 4;17(1):123. doi: 10.1186/s13058-015-0636-6 (PMC4560928; doi:10.1186/s13058-015-0636-6)

Supplemental Figure 2

A (i)

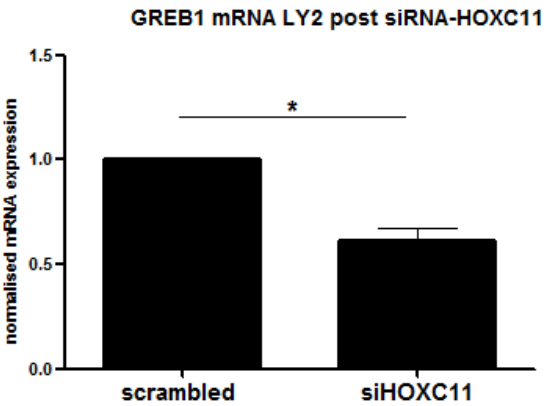

(ii)

| Gene ID         | Gene  | Locus               | fpkmscram | fpkmsiHOXC11 |
|-----------------|-------|---------------------|-----------|--------------|
| ENSG00000196208 | GREB1 | 2:11674241-11782914 | 46.778    | 31.369       |

B

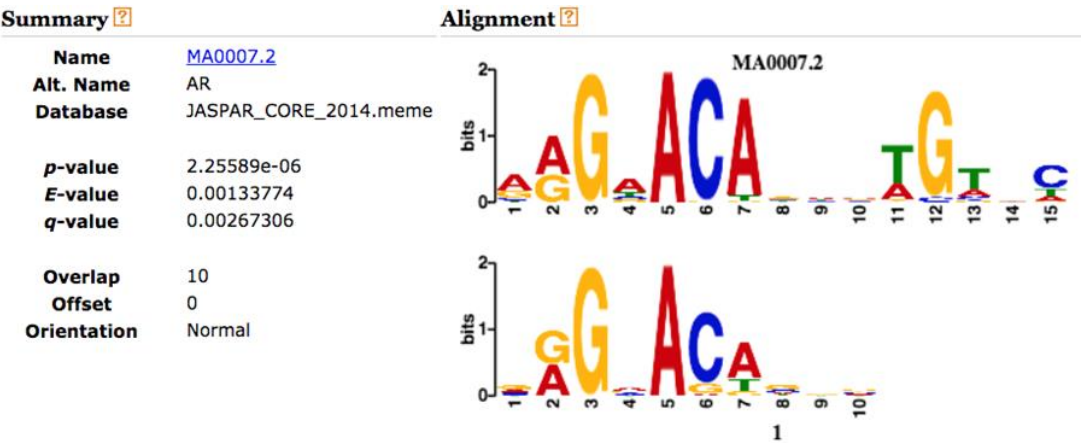

Supplement: Additional file 4: Figure S2. — a (i) Validation of HOXC11 target gene, GREB1, in LY2 cells in which HOXC11 was knocked down by siRNA. (ii) Levels of concomitant GREB1 decrease when HOXC11 is silenced is comparable to alterations observed in HOXC11 RNA-seq data. b The top novel motif returned from MEME was then compared to all known motifs annotated in the JARSPAR database (JARSPAR CORE 2014) using the TOMTOM program. The top matched motif is shown to be androgen receptor (AR) (p value: 2.26e-6) and the second most similar motif is NR3C1 glucocorticoid receptor (GR) (p value: 2.81e-4). (PDF 209 kb) [file 13058_2015_636_MOESM4_ESM.pdf]

Supplemental Figure 3

MCF7 cells – AR nuclear translocation

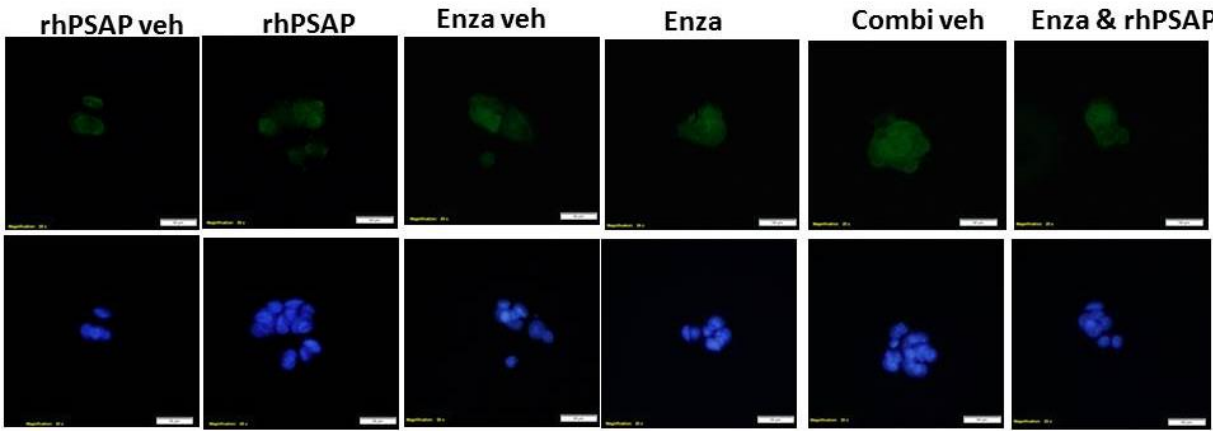

Supplement: Additional file 7: Figure S3. — Representative images of androgen receptor (AR) nuclear translocation in MCF7 cells following individual treatments with recombinant human prosaposin (rhPSAP) and enzalutamide (Enza) and combination treatments. (PDF 114 kb) [file 13058_2015_636_MOESM7_ESM.pdf]

Supplemental Figure 4

A

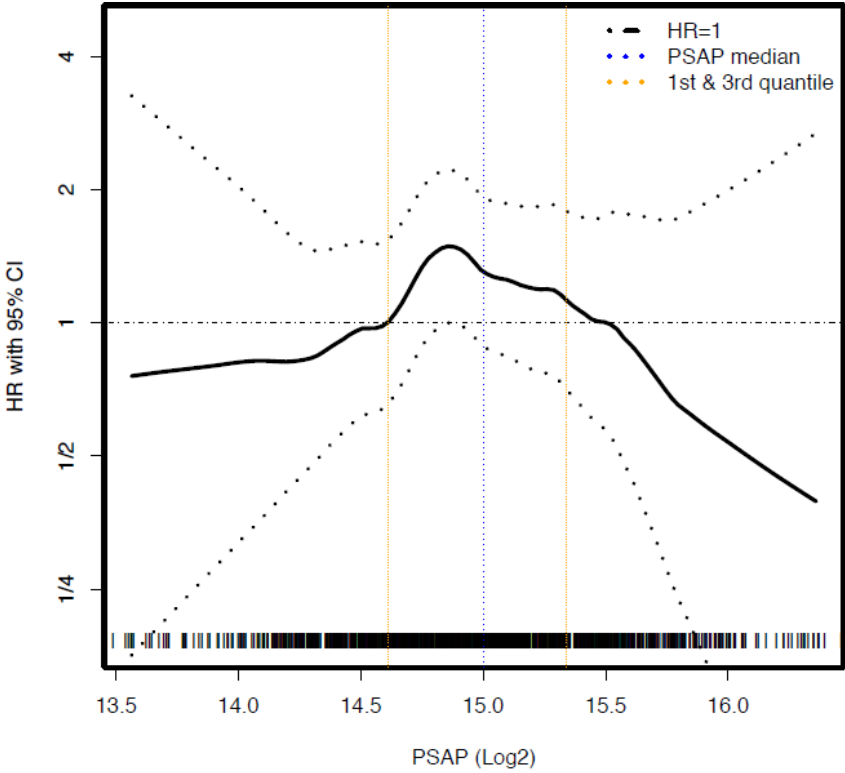

B

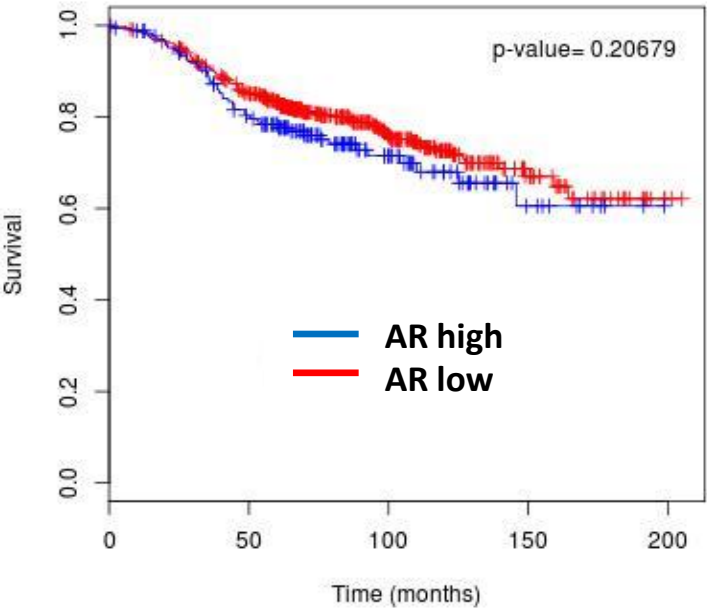

n= 661, number of events= 158  
Hazard ratio = 1.247 (0.8845 - 1.758)  
Score (logrank) test = 1.59 on 1 df, p=0.2069

Supplement: Additional file 8: Figure S4. — a Hazard ratio (HR) curve was generated for prosaposin (PSAP) using the TCGA dataset. PSAP expressions between 25 and 75 % quantiles have a HR consistently >1. b Androgen receptor (AR) mRNA does not associate with poor disease-free survival (DFS) in endocrine-treated breast cancer. Kaplan-Meier survival curves were generated to assess the impact of high androgen receptor transcript levels on survival of endocrine-treated patients with breast cancer (n = 661). (PDF 204 kb) [file 13058_2015_636_MOESM8_ESM.pdf]

Supplemental Figure 5

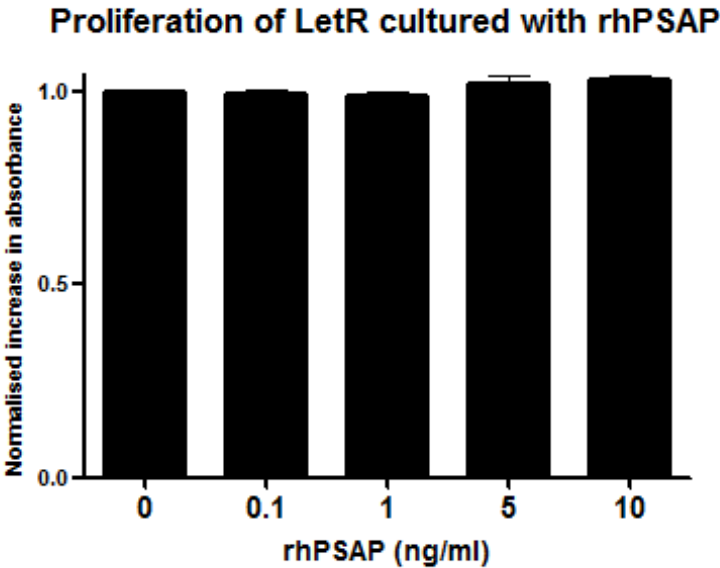

Supplement: Additional file 9: Figure S5. — An MTS assay was utilised to assay the impact of increasing doses of recombinant human prosaposin (rhPSAP) on letrozole-resistant (LetR) cell proliferation after 24 hours treatment. No change in LetR cell proliferation was detected following increased doses in rhPSAP (n = 3). (PDF 86 kb) [file 13058_2015_636_MOESM9_ESM.pdf]

Supplemental Figure 6

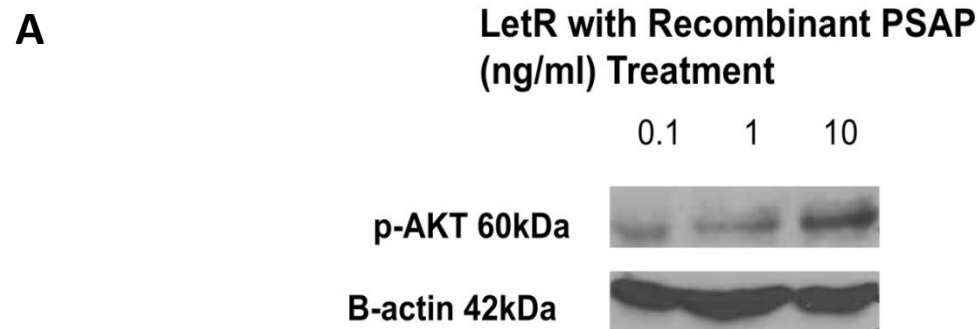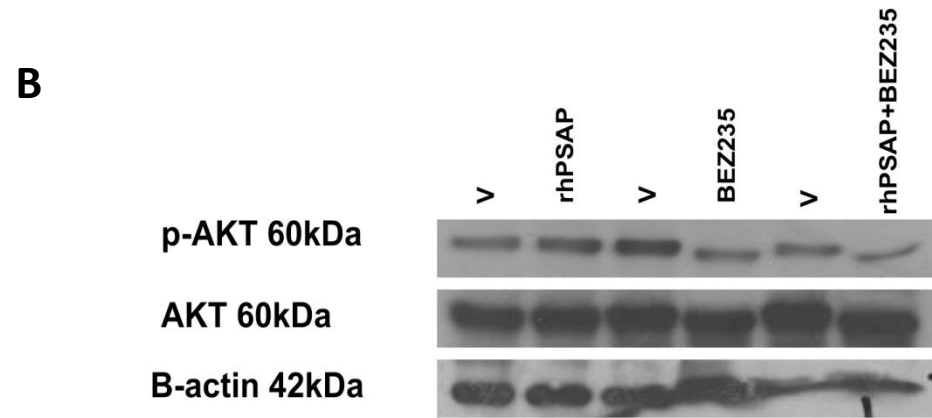

Supplement: Additional file 10: Figure S6. — a Letrozole-resistant (LetR) cells were treated with increasing doses of recombinant human prosaposin (rhPSAP). p-AKT expression in these cells showed a dose-dependent increase with rhPSAP treatment (n = 3). b In LetR cells, there is increased protein expression of p-AKT when treated with rhPSAP. However, when treated with BEZ235 (PI3K inhibitor), there is marked reduction in p-AKT expression. This reduction is also similarly seen in the combination group of rhPSAP and BEZ235 treatments (n = 3). (PDF 138 kb) [file 13058_2015_636_MOESM10_ESM.pdf]
