# Supplementary material for: Prosaposin activates the androgen receptor and potentiates resistance to endocrine treatment in breast cancer
Source: Breast Cancer Res. 2015 Sep 4;17(1):123. doi: 10.1186/s13058-015-0636-6 (PMC4560928; doi:10.1186/s13058-015-0636-6)
Supplement: Additional file 5: — Table: Androgen receptor (AR) motif results p <0.001. A 400-bp-sized window surrounding starting sites of HOXC11 target genes was selected for AR motif searching. The searching process was performed using the FIMO program available in MEME-suite with the p value significant cutoff set at 0.001. (PDF 804 kb) [file 13058_2015_636_MOESM5_ESM.pdf]

motif	gene	start	stop	strand	p.value	matched.sequence
AR	ENSG00000255958	28	42	+	7.57E-07	AAGAACAGAATGCTC
AR	ENSG00000168890	98	112	-	1.09E-06	AAGTACACAATGTAC
AR	ENSG00000100399	200	214	-	2.29E-06	AGGGACAGGCTGTCC
AR	ENSG00000221978	336	350	-	2.56E-06	GAGTACAGCCTGTCC
AR	ENSG00000175324	275	289	-	3.02E-06	AGGAACACGGTGTCT
AR	ENSG00000125148	321	335	-	3.62E-06	GAGGACACAGTGTAC
AR	ENSG00000254827	253	267	+	5.00E-06	GAGCACAGGATGTGC
AR	ENSG00000115977	52	66	-	5.09E-06	GGGAACACACTGTGA
AR	ENSG00000138495	231	245	-	5.09E-06	AAGTACAGGAAGTCC
AR	ENSG00000126217	9	23	-	5.65E-06	GGGAACAGCCTGCCC
AR	ENSG00000237669	296	310	-	5.65E-06	AGGCACAGACTGACC
AR	ENSG00000229628	320	334	+	5.86E-06	AAGCACAAACTGTAC
AR	ENSG00000251018	292	306	-	6.54E-06	AAGTACAGATAGTTC
AR	ENSG00000107371	284	298	+	7.25E-06	AGGTACACCGAGTCC
AR	ENSG00000184084	184	198	+	7.89E-06	AGGCACATAGAGTCC
AR	ENSG00000113269	218	232	-	8.14E-06	CGGAACACAAAGTCC
AR	ENSG00000104320	203	217	-	9.02E-06	GGGAACATCATGTTT
AR	ENSG00000225782	28	42	+	9.61E-06	GGGAACAAACTGAAC
AR	ENSG00000148737	175	189	-	1.19E-05	AGGAACACACTGCCT
AR	ENSG00000251357	88	102	-	1.24E-05	GAGAACACAGAGTGA
AR	ENSG00000163743	128	142	+	1.26E-05	AAGAACAGCAAGTAA
AR	ENSG00000207314	196	210	+	1.31E-05	CAGAACAGCATGTTT
AR	ENSG00000250174	252	266	-	1.35E-05	GAGAACAAGCTGACC
AR	ENSG00000237317	168	182	-	1.41E-05	TAGAACAGTCAGTGC
AR	ENSG00000166337	32	46	-	1.41E-05	GGGAACACAAAGTTA
AR	ENSG00000213641	27	41	+	1.41E-05	CAGTACACCATGTTC
AR	ENSG00000196353	283	297	+	1.41E-05	AAGAACATACTGACA
AR	ENSG00000050426	301	315	+	1.48E-05	AAGGACAGGGTGCCC
AR	ENSG00000138496	201	215	-	1.48E-05	AGGAACACGAAGCCC
AR	ENSG00000151150	246	260	-	1.50E-05	AGGAACACCAAGCGC
AR	ENSG00000204775	27	41	-	1.54E-05	GAGAACACCCAGCTC
AR	ENSG00000186298	237	251	-	1.57E-05	AAGAACAAAATGGCC
AR	ENSG00000110619	70	84	-	1.69E-05	CGGGACAGGGTGTGC
AR	ENSG00000079337	5	19	-	1.69E-05	AAGCACACCCTGTCT
AR	ENSG00000138100	232	246	+	1.71E-05	AGGGACAAACAGTGC
AR	ENSG00000230118	174	188	+	1.74E-05	AGGAACAGGCAGGCC
AR	ENSG00000227323	271	285	-	1.74E-05	AAGCACATTGTGTTT
AR	ENSG00000249967	375	389	+	1.76E-05	GGGAACACCCAGCTC
AR	ENSG00000107331	103	117	-	1.79E-05	GAGGACAGGCTGACC
AR	ENSG00000235267	159	173	+	1.95E-05	GAGAACACTTTGCTC
AR	ENSG00000012048	172	186	-	1.98E-05	AGGGACAGAAAGAGC
AR	ENSG00000198496	89	103	+	1.98E-05	AGGGACAGAAAGAGC
AR	ENSG00000168000	168	182	-	2.03E-05	AAGTACAGCGTGGCC
AR	ENSG00000254140	294	308	+	2.06E-05	AAGAACAGAGAGAAA
AR	ENSG00000248820	36	50	+	2.15E-05	AGGCACAGAAAGAAC
AR	ENSG00000163468	123	137	+	2.17E-05	AAGAACAGAGACTGC
AR	ENSG00000129484	184	198	-	2.17E-05	GGGAACACCCAGCGC
AR	ENSG00000175768	215	229	+	2.17E-05	GGGAACAGGAAGTGA
AR	ENSG00000150054	157	171	+	2.23E-05	AAGAACATTGAGGTC

AR	ENSG00000257446	309	323	+	2.30E-05	GGGGACAGACTGTAA
AR	ENSG00000221182	246	260	+	2.49E-05	TGGAACACAATGAAC
AR	ENSG00000227328	44	58	-	2.49E-05	GGGAACAATGTGCAC
AR	ENSG00000247828	173	187	-	2.55E-05	AGGAACTTCCTGTCC
AR	ENSG00000144118	384	398	-	2.65E-05	GAGTACATGCTGTGT
AR	ENSG00000145494	363	377	+	2.69E-05	GGGTACAGGATGCAC
AR	ENSG00000230005	145	159	-	2.72E-05	GGGAACATTATGGCC
AR	ENSG00000231357	176	190	-	2.72E-05	GAGAACAGCATGAGA
AR	ENSG00000133808	238	252	-	2.76E-05	CAGCACATCCTGTCC
AR	ENSG00000236383	39	53	+	2.80E-05	GGGGACAGACAGAGC
AR	ENSG00000231951	362	376	-	2.80E-05	TAGAACAATATGTAC
AR	ENSG00000249199	275	289	+	2.87E-05	AGGGACAGCCAGACC
AR	ENSG00000155506	166	180	-	2.91E-05	AGGTACATCCAGATC
AR	ENSG00000235092	231	245	+	2.94E-05	CAGTACAGTAAGTGC
AR	ENSG00000224078	133	147	-	3.01E-05	GGGAACAAAAAGCTC
AR	ENSG00000175455	234	248	+	3.05E-05	AAGAACAAAATCTTC
AR	ENSG00000234324	262	276	+	3.10E-05	AGGGACACACAGTTA
AR	ENSG00000196923	367	381	-	3.14E-05	AAGCACAGCCTGGAC
AR	ENSG00000227638	41	55	+	3.18E-05	AAGGACAGCTTGAGC
AR	ENSG00000116299	314	328	-	3.21E-05	AAGAACACAGTGGAA
AR	ENSG00000179902	252	266	+	3.21E-05	AAGAACACAGTGGAA
AR	ENSG00000228021	334	348	-	3.21E-05	GGGTACAAAATGTTT
AR	ENSG00000253518	106	120	-	3.26E-05	AAGAACACACTGGAA
AR	ENSG00000258959	379	393	+	3.34E-05	GGGAACAAAATGGAC
AR	ENSG00000244122	122	136	-	3.38E-05	AAGTACAGATAGAAC
AR	ENSG00000232564	34	48	-	3.38E-05	GGGCACACATAGTCC
AR	ENSG00000217624	187	201	+	3.38E-05	GGGAACATCCAGTCA
AR	ENSG00000259141	301	315	-	3.38E-05	GAGGACAAAGTGTTT
AR	ENSG00000161179	352	366	-	3.50E-05	AGGGACACGCTGGTC
AR	ENSG00000138641	245	259	+	3.50E-05	AAGTACATGAAGTTT
AR	ENSG00000138794	64	78	-	3.77E-05	GGGGACACACAGACC
AR	ENSG00000059378	46	60	-	3.77E-05	GGGAACAGTGTGGTT
AR	ENSG00000141380	190	204	-	3.81E-05	AAGGACAAGGTGAAC
AR	ENSG00000163900	98	112	+	3.81E-05	AAGGACAGAGAGGGC
AR	ENSG00000256029	94	108	+	3.85E-05	GAGAACAGATTGAGA
AR	ENSG00000061938	356	370	+	3.85E-05	AGGAACAGGGTGGGA
AR	ENSG00000199916	237	251	-	3.85E-05	AGGAACAGAGTCCTC
AR	ENSG00000231563	248	262	-	3.90E-05	TGGAACAGCATGCCC
AR	ENSG00000244604	268	282	-	4.00E-05	GGGCACAGTTAGTGC
AR	ENSG00000160746	271	285	+	4.00E-05	GAGCACAGGTAGTAC
AR	ENSG00000237806	312	326	-	4.00E-05	GAGCACAGGTTGATC
AR	ENSG00000235423	285	299	-	4.04E-05	AAGAACACCATGGGT
AR	ENSG00000258388	162	176	-	4.04E-05	CAGAACACGAAGTTT
AR	ENSG00000090530	339	353	+	4.09E-05	AGGAACAGGAAGCCT
AR	ENSG00000228477	278	292	+	4.14E-05	AAGGACATGAAGATC
AR	ENSG00000234750	381	395	+	4.14E-05	AAGGACATGAAGATC
AR	ENSG00000215450	386	400	+	4.14E-05	AAGGACATGAAGATC
AR	ENSG00000135245	7	21	+	4.14E-05	CAGAACACAATGACT
AR	ENSG00000226769	375	389	+	4.18E-05	GAGAACAGAAAGCTA
AR	ENSG00000147677	297	311	-	4.23E-05	AGGCACAGAGGGTCC

AR	ENSG00000235681	352	366	+	4.28E-05	AAGTACAGGGTGCAT
AR	ENSG00000240776	375	389	+	4.33E-05	AGGAACAGTTTGCCT
AR	ENSG00000249921	50	64	-	4.33E-05	CAGAACAGGCAGTCA
AR	ENSG00000214593	361	375	+	4.33E-05	AGGAACAGTTTGCCT
AR	ENSG00000233436	375	389	+	4.39E-05	AGGCACTCACTGTGC
AR	ENSG00000257894	339	353	-	4.54E-05	AGGCACATGCAGAGC
AR	ENSG00000152465	288	302	+	4.75E-05	GAGGACAGCGAGTCT
AR	ENSG00000245148	197	211	-	4.75E-05	GAGGACACATTGCTC
AR	ENSG00000244266	321	335	+	4.75E-05	AAGTACAACATGTGA
AR	ENSG00000219433	304	318	+	4.80E-05	AAGAACAGAGTCTCA
AR	ENSG00000248423	341	355	+	4.80E-05	AAGAACAACAAGGTC
AR	ENSG00000227589	189	203	+	4.86E-05	AAGCACACTCAGCAC
AR	ENSG00000258136	86	100	-	4.91E-05	AAGCACATCCAGTCT
AR	ENSG00000171189	285	299	-	4.91E-05	GGGTACAGGGTGAGT
AR	ENSG00000224660	2	16	+	4.91E-05	GAGAACTGAATGAAC
AR	ENSG00000169583	177	191	+	4.91E-05	CAGCACACTCAGTAC
AR	ENSG00000143627	114	128	+	5.02E-05	GGGGACAGGGTGGCC
AR	ENSG00000165238	189	203	-	5.08E-05	AAGGACACACAGGCC
AR	ENSG00000240682	253	267	-	5.14E-05	AGGGACAGGAAGTGA
AR	ENSG00000244580	146	160	+	5.20E-05	CAGAACAGCTTGCAC
AR	ENSG00000250328	78	92	+	5.26E-05	AAGAACAGTATGATG
AR	ENSG00000256757	155	169	-	5.31E-05	CGGGACAGAATGCCC
AR	ENSG00000111331	9	23	-	5.31E-05	GGGTACATACTGAGT
AR	ENSG00000137965	164	178	+	5.37E-05	GGGCACACTGAGTTT
AR	ENSG00000205424	260	274	+	5.43E-05	AGGCACACACAGGCC
AR	ENSG00000229674	209	223	-	5.43E-05	CAGAACATTTTGTTT
AR	ENSG00000134825	212	226	+	5.55E-05	GAGAACAGAGTCTCT
AR	ENSG00000168496	341	355	-	5.55E-05	GAGAACAGAGTCTCT
AR	ENSG00000257077	281	295	+	5.55E-05	AGGAACAGTTTGTGG
AR	ENSG00000258790	306	320	-	5.61E-05	AGGTACAGGAAGGGC
AR	ENSG00000254633	350	364	-	5.61E-05	CAGCACATGCAGTGC
AR	ENSG00000256309	239	253	-	6.19E-05	CAGAACACCGTGATA
AR	ENSG00000010256	252	266	+	6.25E-05	GAGCACATGGAGAGC
AR	ENSG00000154783	376	390	-	6.25E-05	AGGTACACACTGGCT
AR	ENSG00000203301	220	234	-	6.39E-05	GGGGACATAGAGCCC
AR	ENSG00000185507	174	188	+	6.39E-05	AAGAACAGGCGGCGC
AR	ENSG00000197728	300	314	-	6.39E-05	TGGAACAAAGTGCAC
AR	ENSG00000133316	233	247	-	6.45E-05	AAGAACAGATACTAC
AR	ENSG00000167355	122	136	-	6.45E-05	GAGAACAGCATGGGA
AR	ENSG00000119640	358	372	-	6.45E-05	AGGCACAGCCAGGGC
AR	ENSG00000175892	40	54	+	6.52E-05	GGGGACACTCTGGCC
AR	ENSG00000120306	143	157	-	6.59E-05	GGGGACAGAATCTTC
AR	ENSG00000166598	88	102	+	6.73E-05	AAGAACACGTTGCCA
AR	ENSG00000255478	336	350	-	6.86E-05	GAGAACATCCTGGCT
AR	ENSG00000184608	199	213	+	6.86E-05	CAGAACAGACTGGAT
AR	ENSG00000180815	195	209	+	6.86E-05	GGGCACATGGAGAGC
AR	ENSG00000134255	324	338	+	7.00E-05	GAGAACAGAAAGGTA
AR	ENSG00000189410	327	341	-	7.15E-05	GGGAACTGACAGATC
AR	ENSG00000233611	27	41	-	7.15E-05	GGGGACATTCAGAGC
AR	ENSG00000126777	290	304	-	7.30E-05	AAGCACACTGAGGCC

AR	ENSG00000182472	90	104	-	7.30E-05	CGGAACAGCCGGTCC
AR	ENSG00000249540	359	373	+	7.30E-05	AGGGACACTGTGAGA
AR	ENSG00000053747	203	217	-	7.37E-05	AGGAACTGACAGCGC
AR	ENSG00000138442	13	27	-	7.37E-05	CGGCACACAATGTGA
AR	ENSG00000152056	285	299	-	7.37E-05	AGGAACTCCCTGCGC
AR	ENSG00000177731	27	41	+	7.52E-05	AAGTACAGGCACTTC
AR	ENSG00000226099	165	179	+	7.52E-05	AAGTACACAAAGAGA
AR	ENSG00000259163	157	171	-	7.59E-05	CAGTACACAATGGCC
AR	ENSG00000198400	33	47	-	7.67E-05	GAGCACAGTGTGACA
AR	ENSG00000120805	358	372	+	7.67E-05	GAGGACAGGGGGTTC
AR	ENSG00000111291	64	78	-	7.91E-05	AAGGACAGAAAGACA
AR	ENSG00000078319	37	51	+	7.91E-05	AAGTACAAAATGACA
AR	ENSG00000189376	289	303	+	7.91E-05	GAGGACTCGGTGTTC
AR	ENSG00000058091	73	87	-	7.98E-05	GGGGACATGCTGGGC
AR	ENSG00000251705	275	289	+	7.98E-05	CAGGACACATTGATC
AR	ENSG00000230165	252	266	-	8.06E-05	AAGAACACCAAGGCT
AR	ENSG00000243350	379	393	-	8.14E-05	GGGAACAATGTCTCC
AR	ENSG00000110171	253	267	+	8.14E-05	AGGTACAGACGGTTT
AR	ENSG00000173465	124	138	+	8.14E-05	GGGCACAGCGAGGTC
AR	ENSG00000197381	13	27	+	8.14E-05	TGGAACAGTCAGTAA
AR	ENSG00000182199	36	50	+	8.21E-05	GAGGACAATCTGTCA
AR	ENSG00000174483	373	387	-	8.37E-05	GAGAACAGCAGGAGC
AR	ENSG00000258588	266	280	-	8.37E-05	AGGAACAGAGCGCGC
AR	ENSG00000127928	173	187	+	8.37E-05	GGGAACTCCCTGACC
AR	ENSG00000023839	84	98	-	8.46E-05	AGGGACAGGGAGACT
AR	ENSG00000134571	51	65	+	8.54E-05	AGGGACAATGTGGCC
AR	ENSG00000249633	276	290	-	8.71E-05	AAGCACAGAGAGCCA
AR	ENSG00000109971	186	200	+	8.87E-05	AAGAACACACTCGCC
AR	ENSG00000103056	162	176	+	8.87E-05	AAGAACTATAAGTCC
AR	ENSG00000253502	17	31	-	8.96E-05	GAGCACATTAAGTAT
AR	ENSG00000144504	88	102	-	9.05E-05	CGGGACAGCATGTCA
AR	ENSG00000232830	310	324	+	9.05E-05	GAGGACAGCCAGGCC
AR	ENSG00000196085	276	290	+	9.22E-05	AAGGACATGCAGGGC
AR	ENSG00000243431	64	78	+	9.40E-05	AAGTACAGAGTGCTG
AR	ENSG00000112576	11	25	-	9.48E-05	CAGAACAAGATGGCC
AR	ENSG00000196372	11	25	+	9.57E-05	AAGCACACACACTCC
AR	ENSG00000213707	108	122	+	9.57E-05	CAGTACACTGAGCTC
AR	ENSG00000071082	169	183	-	9.66E-05	GGGAACTGCGTGTCA
AR	ENSG00000223960	369	383	+	9.66E-05	CAGTACAGTAAGTAT
AR	ENSG00000111817	192	206	+	9.66E-05	GAGAACTCTCTGCGC
AR	ENSG00000204977	168	182	-	9.85E-05	CAGAACACGCTGTAG
AR	ENSG00000251154	309	323	+	9.85E-05	AGGAACAATCAGAAA
AR	ENSG00000226746	313	327	-	1.00E-04	CAGGACAGCGTGGGC
AR	ENSG00000258509	261	275	+	0.000101	GGGGACTCCGTGTGC
AR	ENSG00000170289	294	308	+	0.000101	GAGAACAATGAGAAT
AR	ENSG00000135916	53	67	+	0.000102	GGGGACAGGTAGAGC
AR	ENSG00000198894	248	262	+	0.000105	GAGAACAGCGTGCGG
AR	ENSG00000256566	4	18	+	0.000106	GGGGACAGAGACTTC
AR	ENSG00000157800	335	349	+	0.000106	AGGCACAGGCAGCGT
AR	ENSG00000225505	165	179	+	0.000107	AAGAACAATTTGAAA

AR	ENSG00000116455	25	39	+	0.000108	CGGGACAGCATGGTC
AR	ENSG00000067182	206	220	-	0.00011	CGGGACAGGAAGAGC
AR	ENSG00000103023	353	367	+	0.000111	TGGAACATGGTGACT
AR	ENSG00000256142	126	140	-	0.000112	GGGAACAAAAAGAAA
AR	ENSG00000131469	42	56	-	0.000112	GGGAACACAATCTGA
AR	ENSG00000256682	310	324	+	0.000113	AGGAACTGAAAGGTC
AR	ENSG00000129226	300	314	+	0.000113	CAGTACAGGAAGTGA
AR	ENSG00000220739	96	110	+	0.000113	AGGAACTCTAAGTTT
AR	ENSG00000106682	57	71	+	0.000115	CAGCACATAATGGGC
AR	ENSG00000239775	147	161	-	0.000115	GGGAACAGCCTGCGG
AR	ENSG00000215208	343	357	-	0.000116	CAGGACATGGAGATC
AR	ENSG00000115541	208	222	-	0.000116	TAGCACACCCTGCGC
AR	ENSG00000240596	261	275	+	0.000116	AAGCACAGTTTGCCT
AR	ENSG00000229780	60	74	-	0.000117	AGGAACAGTTGGAGC
AR	ENSG00000247372	52	66	+	0.000117	AAGAACAGGCACCGC
AR	ENSG00000258974	232	246	-	0.000117	GAGTACTGAATGTCT
AR	ENSG00000230753	237	251	+	0.000118	CAGAACAGTAAGAGA
AR	ENSG00000106336	161	175	+	0.000118	GGGCACAGGATGTGG
AR	ENSG00000180398	103	117	+	0.000119	AAGGACACAGAGCAA
AR	ENSG00000174516	352	366	-	0.00012	GGGGACACCCAGGGC
AR	ENSG00000239607	126	140	-	0.00012	TGGAACAAACTGACT
AR	ENSG00000100280	293	307	-	0.000121	AGGGACAGGAAGAGA
AR	ENSG00000178685	173	187	+	0.000121	CAGCACACTGAGTCT
AR	ENSG00000224295	168	182	+	0.000122	AAGTACTTACTGCGC
AR	ENSG00000207437	90	104	-	0.000122	AGGAACAAGATGGAA
AR	ENSG00000232682	81	95	+	0.000124	AGGCACACAGGGTTT
AR	ENSG00000213689	127	141	+	0.000124	AAGGACAAGCTCTTC
AR	ENSG00000234076	5	19	+	0.000124	GAGGACAGACAGTGG
AR	ENSG00000213443	88	102	+	0.000125	AAGAACAGTATGGGG
AR	ENSG00000226363	174	188	+	0.000125	TGGTACAGGCAGTGT
AR	ENSG00000235529	20	34	+	0.000125	GAGAACAGGCAGATG
AR	ENSG00000163806	338	352	+	0.000128	AAGTACATGCTGGGA
AR	ENSG00000179222	193	207	+	0.000128	AGGCACAGGAAGTTG
AR	ENSG00000133027	64	78	-	0.000129	GAGAACTACAAGTCC
AR	ENSG00000155744	158	172	-	0.00013	GAGAACACGCTCTCA
AR	ENSG00000093100	269	283	-	0.00013	AAGTACAGTCTGAAG
AR	ENSG00000139350	105	119	-	0.000133	GGGTACAGAGGGTCT
AR	ENSG00000243725	289	303	+	0.000134	TGGAACAACCTGGGC
AR	ENSG00000255909	110	124	+	0.000134	AAGCACAGTCTCAGC
AR	ENSG00000140943	192	206	+	0.000134	CGGGACAGACAGGGC
AR	ENSG00000179409	84	98	+	0.000134	GGGAACATTTTGGTT
AR	ENSG00000207110	91	105	-	0.000134	AAGCACATCTAGCAC
AR	ENSG00000255329	316	330	+	0.000135	TAGGACAATCAGTCC
AR	ENSG00000255990	299	313	+	0.000137	CAGCACACTGAGCCC
AR	ENSG00000171853	94	108	+	0.000137	AAGTACAGGGTCCGC
AR	ENSG00000224020	107	121	-	0.000137	AAGCACATTTAGTCA
AR	ENSG00000115267	230	244	+	0.000138	AAGAACTGCCTGACA
AR	ENSG00000256390	19	33	+	0.000139	CAGGACAGACTGTGG
AR	ENSG00000172794	248	262	+	0.000139	TAGTACACGCTGGGC
AR	ENSG00000234336	352	366	-	0.000139	GAGCACATCGAGGAC

AR	ENSG00000173281	354	368	-	0.000139	GGGAACAGGGACAGC
AR	ENSG00000197892	116	130	+	0.000139	GGGAACTACAAGTCC
AR	ENSG00000143416	269	283	-	0.00014	TGGTACACTTTGATC
AR	ENSG00000100316	171	185	+	0.00014	GAGAACTGCTTGAAC
AR	ENSG00000227848	62	76	+	0.000141	TAGAACATGGAGGCC
AR	ENSG00000133193	278	292	+	0.000141	AGGGACACAATGATG
AR	ENSG00000204498	104	118	-	0.000141	GGGTACTGCCTGAGC
AR	ENSG00000116171	111	125	+	0.000143	GGGGACAGGTTGACT
AR	ENSG00000121310	260	274	-	0.000143	GGGGACAGGTTGACT
AR	ENSG00000142949	203	217	+	0.000145	GAGAACACGAGGTCA
AR	ENSG00000228838	108	122	+	0.000145	GGGGACAGAATGGAA
AR	ENSG00000255010	278	292	-	0.000146	GAGCACATCAAGGCC
AR	ENSG00000252304	170	184	-	0.000147	AGGAACAGTGGGAAT
AR	ENSG00000165792	71	85	-	0.000149	GAGAACTCTGAGTCT
AR	ENSG00000153046	183	197	-	0.00015	AGGAACAAAGGGGTC
AR	ENSG00000247670	89	103	-	0.00015	CGGAACACTCAGAAA
AR	ENSG00000176444	185	199	-	0.000151	AGGAACAGAGGGTGG
AR	ENSG00000224055	329	343	-	0.000151	TAGAACATGTAGACC
AR	ENSG00000152672	380	394	-	0.000152	AAGGACAGGCAGGCT
AR	ENSG00000242247	3	17	-	0.000154	AAGGACAGCAGGTTT
AR	ENSG00000230042	169	183	+	0.000154	TGGCACAGCCTGGGC
AR	ENSG00000242761	319	333	+	0.000154	AAGGACAAACACTTC
AR	ENSG00000234271	178	192	+	0.000155	CAGGACAGGGTGAGA
AR	ENSG00000251447	85	99	+	0.000155	CAGCACATTGTGATT
AR	ENSG00000151164	144	158	-	0.000156	CAGAACAGGAAGTAG
AR	ENSG00000080815	254	268	+	0.000156	TGGGACAGGCAGCTC
AR	ENSG00000163661	238	252	+	0.000156	AAGGACTCTCTGCTC
AR	ENSG00000228834	173	187	+	0.000156	AAGAACACAGCGGAC
AR	ENSG00000235763	194	208	-	0.000159	TAGAACATGATGCTA
AR	ENSG00000232398	258	272	+	0.000159	CAGAACAATGTCTAC
AR	ENSG00000100811	159	173	+	0.00016	AAGAACAAACACCTC
AR	ENSG00000250487	125	139	-	0.00016	AAGTACACAATGCAG
AR	ENSG00000223878	209	223	-	0.000161	AAGAACATGGTGGGG
AR	ENSG00000164163	11	25	-	0.000163	CAGGACATTCAGTTA
AR	ENSG00000248881	142	156	-	0.000163	AAGAACTCACAGACT
AR	ENSG00000182899	75	89	+	0.000164	GGGAACATCCTCCAC
AR	ENSG00000248399	210	224	+	0.000164	AAGTACATTAAGCCA
AR	ENSG00000168237	372	386	-	0.000165	AGGCACTGACTGGGC
AR	ENSG00000148344	159	173	-	0.000165	AAGCACAAAGTGGGA
AR	ENSG00000255276	115	129	+	0.000167	GAGCACACCATGGCT
AR	ENSG00000100385	10	24	-	0.000167	GGGGACACAGAGCCA
AR	ENSG00000249310	89	103	+	0.000168	AAGTACTTGATGTCT
AR	ENSG00000229732	313	327	+	0.00017	GAGCACAGAGGGCCC
AR	ENSG00000249319	114	128	-	0.00017	GAGGACAGGGAGTGG
AR	ENSG00000114388	57	71	-	0.000171	GGGTACACGTTGACA
AR	ENSG00000231980	183	197	+	0.000171	GGGCACTGGCTGACC
AR	ENSG00000161980	267	281	+	0.000172	GGGAACGGGCTGATC
AR	ENSG00000072201	231	245	+	0.000172	TGGAACTGTAAGTCC
AR	ENSG00000258607	1	15	-	0.000172	CAGGACAGTGGGTGC
AR	ENSG00000244723	155	169	-	0.000174	CAGAACAATTAGTCT

AR	ENSG00000114942	185	199	+	0.000176	AAGGACAACTTGGAC
AR	ENSG00000164032	268	282	+	0.000176	GGGAACATTCTGCAG
AR	ENSG00000256159	182	196	+	0.000178	GAGAACAGCCACCAC
AR	ENSG00000066136	181	195	+	0.000179	GAGCACAGCTTCTGC
AR	ENSG00000255158	184	198	+	0.000179	AAGCACAGAGAGAGG
AR	ENSG00000096093	105	119	+	0.000179	GAGAACGCGGAGTCC
AR	ENSG00000073614	138	152	-	0.000181	GGGCACAAGCTCTTC
AR	ENSG00000248492	113	127	+	0.000181	CAGCACAGAAAGAAT
AR	ENSG00000253950	381	395	-	0.000181	GAGAACTCAGGGTGC
AR	ENSG00000229212	204	218	-	0.000183	GAGTACACGCGGAGC
AR	ENSG00000104325	219	233	-	0.000183	CGGGACAAAAAGAAC
AR	ENSG00000095574	63	77	-	0.000185	GGGTACTGAGTGGGC
AR	ENSG00000254452	26	40	+	0.000185	AAGCACAAGCAGCTT
AR	ENSG00000137817	263	277	-	0.000185	AGGAACAAGCAGGAA
AR	ENSG00000047648	356	370	-	0.000185	GGGGACACCGAGCGT
AR	ENSG00000183020	157	171	+	0.000186	AAGGACAGTGAGGCA
AR	ENSG00000168386	44	58	-	0.000186	AAGCACAACCTGGGT
AR	ENSG00000251095	174	188	+	0.000186	TAGAACAATATGCTT
AR	ENSG00000248772	61	75	-	0.000188	TGGAACAACAAGTGA
AR	ENSG00000005022	4	18	-	0.000188	GAGAACACCCGGGAC
AR	ENSG00000237416	348	362	+	0.000189	CAGAACAGGAAGGAT
AR	ENSG00000176532	174	188	+	0.000189	GGGAACGCGAAGTTC
AR	ENSG00000206874	139	153	-	0.000189	AAGAACTTACTGCAA
AR	ENSG00000233885	261	275	+	0.000191	GGGAACAGGCAGCGG
AR	ENSG00000125733	269	283	-	0.000192	AAGAAGAGGAAGTCC
AR	ENSG00000126226	366	380	-	0.000194	GGGGACACACGGAAC
AR	ENSG00000172345	97	111	-	0.000194	CAGGACAAGGAGACC
AR	ENSG00000109046	298	312	+	0.000194	GGGTACAGGGTCTAT
AR	ENSG00000145016	306	320	-	0.000194	AAGGACACGGAGGAT
AR	ENSG00000256268	78	92	+	0.000195	CAGAACACCCAGGCT
AR	ENSG00000100934	192	206	-	0.000195	GAGAACGGCGTGAAC
AR	ENSG00000155269	259	273	+	0.000195	AGGGACACTGAGGCT
AR	ENSG00000120738	348	362	-	0.000197	GGGAACACTGAGAAG
AR	ENSG00000201207	158	172	+	0.000197	AGGGACAAATTGCCT
AR	ENSG00000203276	117	131	-	2.00E-04	AGGAACTGTTTGATT
AR	ENSG00000253536	344	358	-	2.00E-04	AAGAACTGGCAGCTT
AR	ENSG00000107201	313	327	+	2.00E-04	AGGCACAGCCTGCGG
AR	ENSG00000245532	297	311	+	0.000201	GGGGACAGACAGGGA
AR	ENSG00000258725	219	233	+	0.000201	TGGAACAAACAGACT
AR	ENSG00000255905	151	165	-	0.000201	GGGAACTACCTGCAC
AR	ENSG00000213592	206	220	-	0.000203	AAGAACACGGGGTTG
AR	ENSG00000198553	231	245	-	0.000203	GGGAACTTGCAGTAT
AR	ENSG00000130813	67	81	+	0.000203	GAGAACAGAGAGGAG
AR	ENSG00000170509	371	385	-	0.000203	GAGAACAATCTCCCC
AR	ENSG00000224892	213	227	+	0.000204	AGGCACTCTCTGTCA
AR	ENSG00000174748	230	244	+	0.000206	AGGGACACAGCGAGC
AR	ENSG00000224315	10	24	-	0.000207	GGGAACTTTATGGCC
AR	ENSG00000115211	17	31	-	0.000207	GGGGACTCGCTGAGC
AR	ENSG00000243364	214	228	-	0.000211	AGGTACAAAGTGAAG
AR	ENSG00000225374	152	166	+	0.000211	AAGAAGAGACTGTCA

AR	ENSG00000039523	44	58	-	0.000212	CAGAAGAGAATGTGC
AR	ENSG00000169762	305	319	+	0.000212	AAGGACACAGTGGTG
AR	ENSG00000149564	375	389	-	0.000214	AGGAACAAAAACCGC
AR	ENSG00000258830	140	154	-	0.000214	GGGAACTTCTTGAAC
AR	ENSG00000232371	89	103	+	0.000214	CAGGACAGAAAGCCT
AR	ENSG00000236472	250	264	+	0.000215	AGGAACTCGATCTCC
AR	ENSG00000229638	138	152	-	0.000215	AGGAAAAGCATGTTC
AR	ENSG00000229534	350	364	-	0.000217	GAGCACTTGGTGTTT
AR	ENSG00000111615	198	212	+	0.000217	AAGAACAGTTTCATT
AR	ENSG00000164621	218	232	+	0.000219	AAGCACAAAATGATG
AR	ENSG00000232912	375	389	+	0.00022	AGGAAGAGTGTGAGC
AR	ENSG00000145741	350	364	+	0.00022	AGGTACAAAAAGGCT
AR	ENSG00000106628	358	372	+	0.00022	GGGGACAACGAGGAC
AR	ENSG00000167085	128	142	+	0.000224	CAGAACTCACAGCGC
AR	ENSG00000250644	269	283	+	0.000225	CAGGACACTGTGTCG
AR	ENSG00000242777	284	298	+	0.000225	GAGGACAGAAACATC
AR	ENSG00000101654	122	136	-	0.000227	CGGAACTACGAGTCC
AR	ENSG00000257851	355	369	+	0.000229	GAGGACATTTTGAGA
AR	ENSG00000164051	125	139	+	0.00023	AGGCAGAGCGTGTTC
AR	ENSG00000112695	151	165	-	0.00023	CAGAACATAATCTCA
AR	ENSG00000237259	161	175	+	0.000232	CAGCACATGCTGCAT
AR	ENSG00000100154	259	273	+	0.000232	GGGAACATGGCGCGC
AR	ENSG00000134014	18	32	-	0.000232	GGGCACTGCCTGTGA
AR	ENSG00000223522	336	350	-	0.000236	AGGCACGGAGTGTTT
AR	ENSG00000234483	174	188	-	0.000239	AAGAAAAACCATGTTC
AR	ENSG00000256813	87	101	+	0.000241	GGGAACAGTGAGGTG
AR	ENSG00000226784	146	160	-	0.000241	AGGTACAAAAGGTTA
AR	ENSG00000109079	97	111	+	0.000246	AAGCACTTACAGCCC
AR	ENSG00000141219	122	136	+	0.000246	GGGAACAGAGGGGGT
AR	ENSG00000105675	332	346	+	0.000246	GAGAACTATGAGCTC
AR	ENSG00000114270	378	392	+	0.000246	CAGCACAGGGAGAGA
AR	ENSG00000250623	45	59	-	0.00025	TGGTACTTTCTGTGC
AR	ENSG00000233793	252	266	-	0.00025	TGGGACATCTTGAAC
AR	ENSG00000237827	22	36	-	0.000251	AAGGACTTTATGTCA
AR	ENSG00000258088	22	36	-	0.000251	AGGTACAGTTTCTCT
AR	ENSG00000145730	171	185	-	0.000251	AGGGACACTGAGGAA
AR	ENSG00000085563	226	240	+	0.000251	TAGAACATGAAGAAA
AR	ENSG00000139163	127	141	-	0.000255	GGGAACTGCTAGTCT
AR	ENSG00000078687	128	142	-	0.000255	AAGCACTAGCTGCTC
AR	ENSG00000151466	41	55	-	0.000255	TGGGACAGTGTCTCC
AR	ENSG00000115841	226	240	-	0.000257	AGGGACAGTGACAAC
AR	ENSG00000200091	38	52	+	0.000257	AGGGACTAACTGTGA
AR	ENSG00000179988	62	76	+	0.000259	CGGTACAGCGTGGAA
AR	ENSG00000151006	363	377	-	0.000259	AAGAACACTTTGGCG
AR	ENSG00000188313	200	214	-	0.00026	AGGAACTGGGACTCC
AR	ENSG00000111845	163	177	-	0.000262	CGGGACATCCTCTGC
AR	ENSG00000138434	275	289	-	0.000264	GGGGACAGGGAGGGA
AR	ENSG00000178836	385	399	+	0.000264	GAGGACAGGGAGGAA
AR	ENSG00000147403	33	47	+	0.000264	GGGAAGACAGTGACC
AR	ENSG00000198056	217	231	-	0.000268	GGGAACAGCACGGCC

AR	ENSG00000134490	338	352	-	0.000268	AGGTACAAAAGGTGA
AR	ENSG00000238567	318	332	+	0.000268	AAGAACAACTGGTAT
AR	ENSG00000232073	291	305	+	0.000268	GGGAACATAATCCCT
AR	ENSG00000119509	95	109	-	0.000268	GAGGACTCAGTGGAC
AR	ENSG00000258653	117	131	+	0.000272	CAGTACAGCCTCTTT
AR	ENSG00000197429	295	309	+	0.000274	GGGAACTTCCTCTTC
AR	ENSG00000257241	380	394	+	0.000274	AGGTACATGCACAGC
AR	ENSG00000207601	80	94	-	0.000278	AGGGACTTGTAGTCC
AR	ENSG00000028116	247	261	-	0.000279	GAGGACAATCAGAGA
AR	ENSG00000243135	173	187	+	0.000279	AGGCACAGCGTGGGG
AR	ENSG00000153071	146	160	-	0.000279	AGGAACTGACTCTTA
AR	ENSG00000159884	371	385	+	0.000279	GGGCACACCCAGGTA
AR	ENSG00000149735	227	241	-	0.000281	AGGAACAGAGCGGCT
AR	ENSG00000133111	93	107	-	0.000281	CGGGACTGACTGCGC
AR	ENSG00000223703	61	75	-	0.000281	AGGCACTCTGTGATT
AR	ENSG00000184304	78	92	+	0.000283	AAGAAAAGAAAGTAC
AR	ENSG00000241499	169	183	-	0.000283	TAGCACAGAAAGCCT
AR	ENSG00000130702	221	235	-	0.000283	AGGGACAGCGCGCGC
AR	ENSG00000011198	124	138	-	0.000283	AGGTACTTCGAGTTA
AR	ENSG00000236256	342	356	+	0.000283	GAGCACTGAAAGTCA
AR	ENSG00000254872	45	59	-	0.000285	AGGGACAGGCAGCTG
AR	ENSG00000184635	272	286	-	0.000285	CAGGACACAGAGTAG
AR	ENSG00000136045	163	177	-	0.000287	GGGGACAAAATGGAA
AR	ENSG00000258212	347	361	+	0.000287	AGGCACTTCCAGACC
AR	ENSG00000100296	372	386	-	0.000289	GGGAACAGGGTCTAG
AR	ENSG00000177868	293	307	+	0.000291	AGGTACAGACTCCCA
AR	ENSG00000152778	9	23	-	0.000291	GAGTACGCTGAGTTC
AR	ENSG00000139626	253	267	+	0.000291	CAGTACACAAAGGCT
AR	ENSG00000170191	141	155	+	0.000291	AAGAACACCACGCTT
AR	ENSG00000205413	364	378	+	0.000291	AAGTACAATATCTAT
AR	ENSG00000089127	252	266	+	0.000293	AAGGACAAAGGGTAA
AR	ENSG00000214167	13	27	-	0.000293	GGGGACAAAGTCAGC
AR	ENSG00000197114	179	193	+	0.000293	GGGAACACACTCGCT
AR	ENSG00000155016	160	174	+	0.000293	AGGGACAGTCACCGC
AR	ENSG00000248546	234	248	+	0.000293	CGGAACAGGGCGCCC
AR	ENSG00000198742	387	401	-	0.000293	CGGGACACAAACTCC
AR	ENSG00000230953	64	78	+	0.000295	GGGAACAGTGGGCAA
AR	ENSG00000109475	153	167	-	0.000295	CAGGACAGGAAGTTG
AR	ENSG00000112096	369	383	+	0.000295	AGGGACGGAGTGCGC
AR	ENSG00000250641	159	173	+	0.000295	GGGGACACCAGGTCT
AR	ENSG00000239783	138	152	-	0.000297	AGGAACATGCAGGAG
AR	ENSG00000131381	328	342	+	0.000297	GGGTACATACAGAGG
AR	ENSG00000144802	152	166	+	0.000297	GAGAACTGAAAGCGA
AR	ENSG00000237996	183	197	-	0.000299	GAGGACTGCTTGAGC
AR	ENSG00000185482	25	39	-	0.000302	AGGGACAGTTTCTGT
AR	ENSG00000010404	105	119	-	0.000306	TGGAACAGGCACATC
AR	ENSG00000233246	99	113	-	0.000308	GGGGACAGCGCGAGC
AR	ENSG00000127080	199	213	-	0.000308	AGGTACTGGTAGAGC
AR	ENSG00000244336	17	31	-	0.000308	GAGTACAGTTTCTGT
AR	ENSG00000198431	193	207	-	0.00031	GGGAACTGACGGAGC

AR	ENSG00000178623	117	131	+	0.00031	CAGGACTGGGTGTGT
AR	ENSG00000216895	254	268	+	0.00031	GAGGACACACAGAAG
AR	ENSG00000104388	112	126	+	0.00031	CGGAACAACCGGAGC
AR	ENSG00000189337	170	184	-	0.000312	AAGGACACCGCGCGC
AR	ENSG00000149089	72	86	+	0.000316	CAGCACAGCCTGATG
AR	ENSG00000119638	132	146	+	0.000316	GGGAAGAAGGTGTAC
AR	ENSG00000126062	152	166	-	0.000316	TAGAACTACAAGTCC
AR	ENSG00000249242	290	304	-	0.000316	AAGGACAGAATCTTG
AR	ENSG00000255782	291	305	+	0.000318	TGGGACACCCAGGAC
AR	ENSG00000168724	332	346	+	0.000318	GAGGACTGCCAGCGC
AR	ENSG00000247872	276	290	+	0.000318	CGGGACAGGCAGTGG
AR	ENSG00000224665	275	289	-	0.000318	GGGTACAGCAGGGCC
AR	ENSG00000197746	100	114	-	0.000321	AAGAACTTGGAGAAA
AR	ENSG00000186615	225	239	+	0.000321	CGGTACTCGCTGCTC
AR	ENSG00000099953	327	341	-	0.000321	GGGGACAGACAGCCG
AR	ENSG00000238300	285	299	+	0.000321	TAGGACTGAGTGTCT
AR	ENSG00000254964	338	352	+	0.000323	AAGAACTCCCAGCCA
AR	ENSG00000213145	160	174	+	0.000323	CGGGACAGACTCCGC
AR	ENSG00000258011	36	50	+	0.000325	GGGAACACACACACA
AR	ENSG00000227528	88	102	-	0.000325	AAGGACAACCAGGTA
AR	ENSG00000123349	219	233	+	0.000327	GAGAACTTTAAGATT
AR	ENSG00000114735	169	183	+	0.000327	CGGAACACCCGGGCC
AR	ENSG00000137210	171	185	+	0.000327	AAGGACACTGCGCAC
AR	ENSG00000106028	222	236	-	0.000327	GGGAACGCAAAGCTC
AR	ENSG00000228775	191	205	+	0.000327	GGGAACGCAAAGCTC
AR	ENSG00000244663	63	77	+	0.000327	CAGGACAATCTGCAT
AR	ENSG00000167588	241	255	-	0.000329	GGGTACAGACAGGTG
AR	ENSG00000075415	16	30	+	0.000331	AGGGACAAAGCGAGC
AR	ENSG00000093009	164	178	-	0.000331	AAGGACAATGGGCCC
AR	ENSG00000189269	231	245	-	0.000331	GAGCACAGTTGGAGC
AR	ENSG00000204642	325	339	+	0.000331	AAGAAGAAACTGTCT
AR	ENSG00000241404	160	174	+	0.000331	GAGGACTCTCTGGCC
AR	ENSG00000254876	15	29	+	0.000331	GGGGACTGGGAGCGC
AR	ENSG00000233603	131	145	+	0.000334	AAGCACTGCCTCTGC
AR	ENSG00000249055	161	175	-	0.000334	TGGGACAGAGAGCCA
AR	ENSG00000156170	71	85	-	0.000334	CGGGACAGCTAGGGC
AR	ENSG00000254460	56	70	-	0.000336	CAGGACAGGAAGCCA
AR	ENSG00000084754	224	238	-	0.000336	GAGCACTGCCTGACT
AR	ENSG00000070601	332	346	-	0.000336	GGGGACAGGCCGTCT
AR	ENSG00000119608	375	389	+	0.000338	GAGCACATCCAGGCA
AR	ENSG00000105877	16	30	-	0.000338	TGGGACAAGCAGCGC
AR	ENSG00000175147	244	258	+	0.00034	GGGTACATATGGTGT
AR	ENSG00000033011	277	291	+	0.00034	GAGGACAGGGGGGAC
AR	ENSG00000233221	129	143	-	0.000343	GAGCACAGCCCGCGC
AR	ENSG00000228172	210	224	-	0.000345	GGGGACACAGACCCC
AR	ENSG00000237149	342	356	+	0.000345	AGGTACTCGGGGTGC
AR	ENSG00000163918	376	390	+	0.000347	CAGGACAAGGAGGAC
AR	ENSG00000171097	188	202	+	0.000347	AAGCACAAGGAGGCA
AR	ENSG00000092470	350	364	-	0.000349	GAGCACACCTTGGAA
AR	ENSG00000183077	91	105	+	0.000349	AGGGACTGGGAGGGC

AR	ENSG00000163815	321	335	+	0.000349	TGGAACACTGAGGAA
AR	ENSG00000185885	196	210	-	0.000352	GAGAACTGACTCCCC
AR	ENSG00000243284	197	211	+	0.000354	GGGCACTCATTGCAC
AR	ENSG00000119703	329	343	+	0.000354	AAGTACATGGGGATA
AR	ENSG00000114544	2	16	+	0.000354	AAGCACATGCAGCTG
AR	ENSG00000198492	44	58	+	0.000356	GGGGACTGTGTGACT
AR	ENSG00000121749	384	398	+	0.000356	AGGGACACGAGGGAC
AR	ENSG00000213741	318	332	+	0.000356	TAGAACTGTCTGAGT
AR	ENSG00000222020	341	355	+	0.000356	GGGCACACAATGGGG
AR	ENSG00000099901	246	260	+	0.000356	AAGCACAGGCCGGGC
AR	ENSG00000076554	146	160	+	0.000359	GAGGACAAAGGGTGA
AR	ENSG00000159423	1	15	-	0.000361	AAGGACTCGCTGCGT
AR	ENSG00000170027	62	76	+	0.000361	AAGGACGGGGTGCCC
AR	ENSG00000212125	147	161	-	0.000363	CAGCACTGGCTGTGA
AR	ENSG00000257298	326	340	+	0.000363	AGGAAAAATCTGTTC
AR	ENSG00000258064	270	284	-	0.000363	AAGAAAAGTGTGTTT
AR	ENSG00000222489	373	387	-	0.000363	AGGTACATTTTCTTA
AR	ENSG00000247853	357	371	-	0.000366	AAGGACTGTATCTGC
AR	ENSG00000259118	361	375	+	0.000366	GGGAACACAGGGGAA
AR	ENSG00000232485	44	58	+	0.000366	AGGCAGATAGAGTTC
AR	ENSG00000235652	145	159	-	0.000366	CGGGACTGCGTGCGC
AR	ENSG00000130222	351	365	+	0.000366	AAGCACAGCCAGGTG
AR	ENSG00000242517	273	287	+	0.000366	GAGGACACTCTGGTG
AR	ENSG00000172543	248	262	+	0.000368	TGGCACTGACTGCCC
AR	ENSG00000125691	281	295	-	0.000368	AAGGACACTGGGATA
AR	ENSG00000240766	251	265	+	0.000368	AAGGACTGGTAGAAC
AR	ENSG00000228275	72	86	+	0.000368	TGGAAGAGCCTGTCC
AR	ENSG00000110108	208	222	+	0.00037	TAGAACTACATGACC
AR	ENSG00000105397	299	313	+	0.00037	GGGGACAAGCAGTAG
AR	ENSG00000072274	375	389	-	0.00037	GAGGACACGAGGGTC
AR	ENSG00000171604	199	213	-	0.00037	AAGAAAACGCAGTCC
AR	ENSG00000245521	5	19	+	0.00037	GGGAACTGAGGGCCC
AR	ENSG00000174791	92	106	+	0.000373	TAGCACTGAGTGTCA
AR	ENSG00000207392	377	391	+	0.000373	GGGAACTTTATGTAG
AR	ENSG00000073910	76	90	+	0.000375	GAGCACACCCGGGCC
AR	ENSG00000197102	22	36	+	0.000375	TAGGACTCGCAGTGC
AR	ENSG00000253256	163	177	-	0.000378	CGGGACAGGATCAGC
AR	ENSG00000215301	245	259	-	0.000378	GAGAACAGAGGGAAG
AR	ENSG00000237779	364	378	-	0.000378	AAGAACTTTATGGAA
AR	ENSG00000151491	320	334	-	0.00038	GGGGACAGGGACCCC
AR	ENSG00000129194	149	163	+	0.00038	TGGGACAAACAGGAC
AR	ENSG00000083845	18	32	-	0.00038	CAGCACACAATCCCC
AR	ENSG00000105640	37	51	+	0.00038	TGGAACAGGTGGAGC
AR	ENSG00000232626	202	216	-	0.000383	CAGCACACGAAGCCA
AR	ENSG00000126368	287	301	-	0.000383	TGGGACAGAGGGCTC
AR	ENSG00000237380	149	163	+	0.000383	AGGAACGAAGTGATT
AR	ENSG00000154277	349	363	-	0.000383	AAGCACAAATTCAAC
AR	ENSG00000213760	158	172	-	0.000383	GGGAACAAGGTCATA
AR	ENSG00000064652	8	22	+	0.000385	AGGAACAACAGGAAA
AR	ENSG00000164855	104	118	-	0.000385	GGGTACAGCTGGCAC

AR	ENSG00000134508	52	66	-	0.000388	GGGGACAGGGTGGAG
AR	ENSG00000151725	165	179	-	0.000388	CAGGACTTCCTGAGC
AR	ENSG00000129696	45	59	+	0.000388	AAGAACAAACACAAA
AR	ENSG00000160712	48	62	+	0.00039	AGGAACATCGCCTCC
AR	ENSG00000250317	238	252	-	0.00039	CAGAACAAAGTGGGG
AR	ENSG00000110047	386	400	+	0.000392	GGGGACAGCTTCCTC
AR	ENSG00000244134	331	345	-	0.000392	AAGCACAGGAAGGTG
AR	ENSG00000119684	198	212	+	0.000392	TGGAACAACTGGTGC
AR	ENSG00000244468	39	53	+	0.000392	GGGGACTTGGAGAAC
AR	ENSG00000107165	360	374	+	0.000392	AAGCACTACAAGTTT
AR	ENSG00000167996	6	20	+	0.000395	GGGCACAGAGACGCC
AR	ENSG00000003056	44	58	+	0.000395	CGGAACAGCGTCCCT
AR	ENSG00000184508	14	28	+	0.000395	GAGCACACTGAGCTG
AR	ENSG00000234751	285	299	+	0.000397	CAGTACTGCCAGCTC
AR	ENSG00000216306	218	232	+	0.000397	AGGCACAGGGGCTCC
AR	ENSG00000198003	132	146	-	0.000397	GGGCACAGGGAGCGG
AR	ENSG00000227199	52	66	+	0.000397	CGGCACTTGCTGAGC
AR	ENSG00000254088	90	104	+	0.000397	AAGGACATTTTGAAG
AR	ENSG00000205794	76	90	+	4.00E-04	AGGCACTCGGAGGCC
AR	ENSG00000119723	13	27	-	0.000403	TAGTACTCTATGACC
AR	ENSG00000197128	273	287	+	0.000403	CAGAACGCAATGTCA
AR	ENSG00000226143	317	331	+	0.000403	AAGAACACATACACA
AR	ENSG00000166278	332	346	-	0.000403	GGGGACAGAGACGCC
AR	ENSG00000177685	148	162	-	0.000405	AGGCACATCAGGGAC
AR	ENSG00000111229	221	235	+	0.000405	CGGAACTGCAAGGCC
AR	ENSG00000258521	270	284	-	0.000405	CAGCACATCTTGAAA
AR	ENSG00000151117	6	20	+	0.000408	AAGAAGAGTAAGTAT
AR	ENSG00000230259	293	307	+	0.000408	GAGAACTCTAACTCC
AR	ENSG00000164180	289	303	+	0.000408	GGGAACAGAAGCAAC
AR	ENSG00000145425	332	346	+	0.00041	AAGAAGAAAGTGTAA
AR	ENSG00000243806	219	233	+	0.00041	AAGAAGAGAAGGTTC
AR	ENSG00000134333	47	61	+	0.000413	AAGAAGAAACAGATC
AR	ENSG00000258545	24	38	-	0.000413	AAGAACGCTGTGAAA
AR	ENSG00000176029	80	94	+	0.000415	GAGAACACCCCCTCC
AR	ENSG00000139351	173	187	-	0.000415	GAGAAAAGCCAGTCC
AR	ENSG00000248341	85	99	+	0.000415	GGGAACTTCCACTTC
AR	ENSG00000168484	161	175	-	0.000415	GAGCACACAGCGGCC
AR	ENSG00000226677	316	330	+	0.000418	CAGAAGAAGGTGTTC
AR	ENSG00000224281	184	198	+	0.000418	AGGCACACGCGCTTC
AR	ENSG00000129515	271	285	-	0.00042	GAGAACACCCACCAT
AR	ENSG00000235546	160	174	-	0.00042	AGGGACAGGACGGCC
AR	ENSG00000145014	64	78	-	0.00042	GAGAACAAATACCCC
AR	ENSG00000258227	91	105	-	0.00042	AAGGACAATTTGGAA
AR	ENSG00000235471	272	286	-	0.00042	CAGCACACTGTGAAG
AR	ENSG00000186868	34	48	+	0.000423	GAGGACACCCACCCC
AR	ENSG00000130714	381	395	+	0.000423	GGGGACACCGGGGAC
AR	ENSG00000230116	213	227	+	0.000426	AAGAACAAGCACCTT
AR	ENSG00000215127	261	275	+	0.000426	GAGTACATGAACTTA
AR	ENSG00000249144	310	324	-	0.000426	TAGCACATACTGATG
AR	ENSG00000125954	34	48	-	0.000428	GGGCACAGTCTCCTT

AR	ENSG00000207556	253	267	-	0.000428	AAGCACAGCGGGGTT
AR	ENSG00000251467	175	189	-	0.000428	TGGCACTGCCTGCTC
AR	ENSG00000229117	94	108	+	0.000431	AAGAACTAACTCAAC
AR	ENSG00000204618	168	182	-	0.000431	TAGCACACCTGGTTC
AR	ENSG00000135114	255	269	-	0.000434	GGGCACAGGAGGACT
AR	ENSG00000166548	348	362	-	0.000434	CAGAACACGGCGGGC
AR	ENSG00000064489	297	311	+	0.000434	CGGGACAGAGGGGGC
AR	ENSG00000178297	237	251	-	0.000434	GGGCACGAGGTGTAC
AR	ENSG00000095002	32	46	+	0.000434	GGGCACATTACGAGC
AR	ENSG00000132394	54	68	-	0.000434	AGGAACTGGAGGTCA
AR	ENSG00000180929	260	274	-	0.000434	TGGTACTGGCTGTCA
AR	ENSG00000130717	93	107	-	0.000434	CGGCACACCAGGAGC
AR	ENSG00000147586	328	342	+	0.000436	AGGCACTGAGAGTGG
AR	ENSG00000249007	123	137	+	0.000439	GGGTACAGGTGGGTC
AR	ENSG00000135631	209	223	+	0.000439	CGGAACAGAGGGGTA
AR	ENSG00000088298	100	114	-	0.000442	GGGGACGGAATGCAC
AR	ENSG00000204622	93	107	-	0.000442	GAGAACTGGGTCCGC
AR	ENSG00000077782	302	316	+	0.000442	GGGAACTACAAGGCC
AR	ENSG00000155962	53	67	+	0.000442	TAGCACTGCATGTGT
AR	ENSG00000178796	85	99	+	0.000444	CAGCACAAAGGGAGC
AR	ENSG00000137642	280	294	-	0.000444	CAGGAGAGAATGTGC
AR	ENSG00000244462	201	215	-	0.000444	AGGAACGGCGTGGTT
AR	ENSG00000214846	315	329	+	0.000444	AAGAACTATGACTCC
AR	ENSG00000245043	219	233	-	0.000444	GAGAACACTGGCATC
AR	ENSG00000142856	345	359	+	0.000447	TGGAACACCTTCACC
AR	ENSG00000226232	224	238	-	0.000447	AGGAACTGTGGGGTC
AR	ENSG00000220891	181	195	+	0.000447	AGGGACTGTATGCAA
AR	ENSG00000242251	247	261	+	0.000447	TGGGACAGGAGGATC
AR	ENSG00000128595	309	323	-	0.000447	CAGCACAGGATGCCG
AR	ENSG00000207165	215	229	-	0.000447	AGGAACTCAGTCGGC
AR	ENSG00000249755	203	217	+	0.00045	GGGCACAAGTAGCTT
AR	ENSG00000205089	351	365	-	0.00045	AGGAACGCCCAGGGC
AR	ENSG00000236507	204	218	+	0.00045	AAGCACTGAAAGTGG
AR	ENSG00000127720	190	204	-	0.000453	TAGCACAGGCAGGCT
AR	ENSG00000167165	82	96	+	0.000453	GGGAACAGGAACTCG
AR	ENSG00000165675	91	105	+	0.000453	AAGAACAGCCTCCAG
AR	ENSG00000154309	292	306	+	0.000455	CGGGACTGGGAGACC
AR	ENSG00000236546	383	397	-	0.000455	GAGAAGAGGCAGTCT
AR	ENSG00000079819	5	19	+	0.000455	GAGAACGCCTTGCCC
AR	ENSG00000236404	201	215	-	0.000455	GAGAACATGCCGCAT
AR	ENSG00000256898	110	124	+	0.000458	CAGTACTGGAAGTTA
AR	ENSG00000256223	3	17	+	0.000458	CGGCACAGAGTGGCG
AR	ENSG00000135749	67	81	+	0.000461	AGGCACTCACAGAAA
AR	ENSG00000134013	315	329	-	0.000461	GGGCACACACGCTGC
AR	ENSG00000255354	291	305	+	0.000461	AAGAAGAAGCTGCCC
AR	ENSG00000230194	339	353	-	0.000464	AAGAACTTCAGGTCT
AR	ENSG00000125245	124	138	-	0.000466	AGGCACACAGCCTTC
AR	ENSG00000240224	73	87	+	0.000466	GGGCACTCTGTCTTC
AR	ENSG00000242515	192	206	-	0.000466	AAGTACAGGCACAAA
AR	ENSG00000174579	268	282	+	0.000466	GGGAACAGCGCGGGT

AR	ENSG00000223916	225	239	+	0.000466	GAGTACAAGGTGGTG
AR	ENSG00000233558	132	146	-	0.000466	TGGCACAGACAGGAA
AR	ENSG00000110719	347	361	-	0.000469	GGGAAGAGGGTGGGC
AR	ENSG00000178878	161	175	-	0.000469	TGGAACAGAAGCTCC
AR	ENSG00000255581	343	357	-	0.000469	CAGCACAGCTACTCC
AR	ENSG00000221585	258	272	-	0.000469	GGGAACACAGGGCTG
AR	ENSG00000102401	317	331	+	0.000469	AAGTACAAGTGGCCC
AR	ENSG00000116652	132	146	+	0.000472	CAGGACATAATCTGA
AR	ENSG00000183531	2	16	+	0.000472	AGGGACAAGGAGACG
AR	ENSG00000134982	35	49	-	0.000472	CAGCACTTACTGGCC
AR	ENSG00000231542	16	30	+	0.000472	GGGTACAAACTCAGT
AR	ENSG00000253528	204	218	-	0.000475	AAGTACATAGCCTGC
AR	ENSG00000197882	379	393	-	0.000478	AAGGACAGGTTGGAG
AR	ENSG00000137821	277	291	-	0.000478	GAGGACTTGCTGCTT
AR	ENSG00000168092	13	27	-	0.000481	AGGGACATGGGCTTC
AR	ENSG00000258660	231	245	+	0.000481	GAGTACAGTCACACT
AR	ENSG00000212978	34	48	-	0.000481	GAGGACATGTTGACG
AR	ENSG00000114670	26	40	-	0.000481	GAGAACACCTGCTCC
AR	ENSG00000171163	28	42	+	0.000484	GAGCACAGGAGGCCT
AR	ENSG00000227056	117	131	-	0.000484	TGGCACACTTTGCAT
AR	ENSG00000012822	250	264	-	0.000484	AAGGACTTCAAGGCC
AR	ENSG00000226360	270	284	-	0.000484	AGGAACTTGCGGCGC
AR	ENSG00000240053	265	279	+	0.000484	TAGGACTGCGTGTGA
AR	ENSG00000172819	147	161	-	0.000487	AGGCAGAGGGTGTGT
AR	ENSG00000204438	216	230	-	0.000487	CGGCACATGGGGTCT
AR	ENSG00000177628	167	181	-	0.00049	CGGGACTCAGAGCCC
AR	ENSG00000151743	90	104	+	0.00049	AAGAACTACAACTCC
AR	ENSG00000198211	237	251	+	0.00049	AAGAACTGTGGGGAC
AR	ENSG00000167608	287	301	-	0.00049	AGGCAGACACTGCCC
AR	ENSG00000115504	316	330	+	0.00049	TAGGACACAAGGTTA
AR	ENSG00000157851	317	331	-	0.00049	GAGAACTCACCGACC
AR	ENSG00000163870	122	136	+	0.00049	AGGGACACGGAGGGG
AR	ENSG00000085274	148	162	-	0.000492	GGGCACAGTGGGCAT
AR	ENSG00000250656	248	262	-	0.000492	AGGAAGATGAAGAGC
AR	ENSG00000202441	36	50	+	0.000492	AGGCACACAGACACA
AR	ENSG00000106069	129	143	+	0.000492	AGGCACAGGGACCTT
AR	ENSG00000172728	336	350	-	0.000492	AAGAACACCGGGCCG
AR	ENSG00000076321	318	332	+	0.000495	GAGAAGAAAGTGTCA
AR	ENSG00000074582	317	331	+	0.000495	AGGAAGAGAAAGGAC
AR	ENSG00000136883	377	391	-	0.000495	TGGTACATCCTGATG
AR	ENSG00000143093	20	34	+	0.000498	GAGGACAAGAGGTCA
AR	ENSG00000215695	292	306	-	0.000498	AAGCAGAGACAGAGC
AR	ENSG00000228703	155	169	+	0.000498	CGGGACAGGTACTCC
AR	ENSG00000257613	196	210	+	0.000498	AAGAACTGTCACTAT
AR	ENSG00000172209	261	275	-	0.000498	GAGGAAAGCCTGTTC
AR	ENSG00000139116	352	366	-	0.000501	GGGGACAAAGCGCGC
AR	ENSG00000252974	78	92	-	0.000501	GGGGACAAAGCGCGC
AR	ENSG00000258017	248	262	+	0.000501	AGGTACACATGGAAA
AR	ENSG00000134864	284	298	-	0.000501	GAGAACATTTTCCCT
AR	ENSG00000161970	63	77	-	0.000501	GGGAACTGCGAGGGA

AR	ENSG00000167741	303	317	-	0.000501	TAGGACACAAGGCCC
AR	ENSG00000203896	256	270	+	0.000501	CGGGACATCAGGAGC
AR	ENSG00000251003	340	354	-	0.000501	AAGTACTTACTCTTT
AR	ENSG00000227200	309	323	-	0.000501	TGGAACATGGAGAAG
AR	ENSG00000204859	81	95	+	0.000504	AAGAACAGGGCGAGG
AR	ENSG00000110075	16	30	+	0.000504	GGGAAAAGAATGCGC
AR	ENSG00000136044	313	327	+	0.000504	GGGCACGGAGTGGCC
AR	ENSG00000255526	23	37	+	0.000504	AGGGACTCTCAGAAT
AR	ENSG00000167635	200	214	+	0.000504	CAGGACTCCCAGACC
AR	ENSG00000100234	352	366	+	0.000504	GGGCACTCGGAGGGC
AR	ENSG00000237765	283	297	-	0.000504	AAGAACTGGATCGGC
AR	ENSG00000249494	211	225	+	0.000504	GAGAACGCCGTGCGT
AR	ENSG00000124802	208	222	-	0.000504	CAGAACAAGAACCTC
AR	ENSG00000215791	21	35	+	0.000507	GGGCACAGGAGGCGT
AR	ENSG00000110013	133	147	+	0.000507	GGGAACACTGGCTTT
AR	ENSG00000132010	156	170	-	0.000507	CAGAACTGAGTGACG
AR	ENSG00000138433	16	30	-	0.000507	CGGCACTGCTTGACC
AR	ENSG00000089101	147	161	+	0.000507	CGGAACTTGCAGGAC
AR	ENSG00000214597	151	165	+	0.000507	CAGAACTTCTAGAAC
AR	ENSG00000253773	44	58	+	0.000507	AAGAACAGGCCGAGG
AR	ENSG00000213782	186	200	+	0.00051	GAGAACTAAAACTAC
AR	ENSG00000226352	40	54	-	0.000513	CAGTACAAGCGGAAC
AR	ENSG00000080823	162	176	+	0.000513	GGGGACGGGCTGCAC
AR	ENSG00000170144	103	117	-	0.000513	GGGAACGCCTTGTGA
AR	ENSG00000159082	378	392	-	0.000513	AGGCAGAGACTGGTC
AR	ENSG00000008988	291	305	+	0.000513	AGGAACAAGTCGGTC
AR	ENSG00000148296	91	105	+	0.000513	AAGGACGGAATGAAT
AR	ENSG00000249115	77	91	+	0.000516	GGGAAGAGTGAGCGC
AR	ENSG00000068724	384	398	-	0.000516	GGGGACAGCGGCTGC
AR	ENSG00000214753	84	98	+	0.000519	AGGAACTCCGTCGTC
AR	ENSG00000089022	45	59	-	0.000519	AAGCACTGAAAGGGT
AR	ENSG00000213398	129	143	-	0.000519	AGGGACGGCCTGGCC
AR	ENSG00000204314	226	240	-	0.000519	AGGAAGATCCTGGAC
AR	ENSG00000214300	289	303	-	0.000519	CAGAAGAGTGTGTTA
AR	ENSG00000244697	322	336	+	0.000519	CAGAAGAGTGTGTTA
AR	ENSG00000173988	266	280	+	0.000522	GGGAACAGCCTCCTG
AR	ENSG00000224189	103	117	-	0.000522	CAGTACGCGCAGTGC
AR	ENSG00000237298	232	246	-	0.000522	AGGGACATAATCCAA
AR	ENSG00000145592	134	148	-	0.000522	CGGGACTCCCAGTTT
AR	ENSG00000233927	354	368	-	0.000525	CGGAACTGGGCGTCC
AR	ENSG00000102882	234	248	+	0.000528	GAGGACAGTCTCCGA
AR	ENSG00000152348	342	356	-	0.000528	CGGAACTGAATGATG
AR	ENSG00000187109	24	38	+	0.000531	AGGAAAAAACTGTTT
AR	ENSG00000006740	109	123	-	0.000531	GAGAACGCCGTGCAT
AR	ENSG00000254501	351	365	+	0.000534	GGGCACAGGGTCTGG
AR	ENSG00000258168	367	381	+	0.000534	AAGGACAAGAACCCC
AR	ENSG00000258232	309	323	+	0.000534	CAGCACTATCTGCTC
AR	ENSG00000100568	172	186	-	0.000534	CGGGACTGGCAGCGC
AR	ENSG00000137877	283	297	-	0.000534	AGGGACATCAGGAGA
AR	ENSG00000222345	139	153	-	0.000534	AAGAACTTTTAGAAA

AR	ENSG00000167100	335	349	-	0.000538	GGGAACAGGTTCGCT
AR	ENSG00000114126	199	213	-	0.000538	CAGCACAGTGCGCGC
AR	ENSG00000221267	169	183	+	0.000538	CAGCACAACCAGCCA
AR	ENSG00000023171	162	176	-	0.000541	AAGCACTGCCAGCCA
AR	ENSG00000144401	195	209	-	0.000541	AAGCACAGTGGGGGA
AR	ENSG00000215298	205	219	-	0.000541	AGGCACATGAAGGTG
AR	ENSG00000256329	30	44	+	0.000541	AAGAAGATACTCTGC
AR	ENSG00000254400	324	338	+	0.000544	CGGCACTGAGTGAAA
AR	ENSG00000185633	37	51	-	0.000544	GGGCACAGACACACA
AR	ENSG00000245750	344	358	-	0.000544	GGGAACGGCTAGAAC
AR	ENSG00000248245	84	98	+	0.000544	AGGGACAAGAACTAT
AR	ENSG00000160844	137	151	-	0.000544	CAGGACTTGTAGTCC
AR	ENSG00000147804	340	354	-	0.000544	CAGCACAGCCAGAAG
AR	ENSG00000229127	274	288	-	0.000547	TAGGACATCTGGTCC
AR	ENSG00000234353	147	161	-	0.000547	CAGGACATTGGGTCA
AR	ENSG00000241853	331	345	+	0.000547	GGGCACTCAGAGAGA
AR	ENSG00000147642	383	397	-	0.000547	GAGGACAAAATGGAG
AR	ENSG00000119396	110	124	-	0.000547	AGGGACATATAGCCG
AR	ENSG00000232739	181	195	-	0.00055	CAGAACATCGACATT
AR	ENSG00000023191	302	316	-	0.00055	CAGAACGGGTTGAAC
AR	ENSG00000129535	63	77	+	0.00055	TAGGACTCAAAGACC
AR	ENSG00000258571	199	213	+	0.00055	AGGAAAACAGAGAAC
AR	ENSG00000163590	276	290	-	0.00055	GGGTACGCAGAGCCC
AR	ENSG00000113231	83	97	+	0.00055	GGGCACGCTCTGCCC
AR	ENSG00000223501	211	225	+	0.00055	AGGAAGAGGCGGTGC
AR	ENSG00000231500	214	228	-	0.00055	AGGAAGAGGCGGTGC
AR	ENSG00000160710	349	363	-	0.000556	AAGGACAGAGGCTCT
AR	ENSG00000148814	360	374	+	0.000556	GGGTACGGCCAGCTC
AR	ENSG00000136143	223	237	+	0.000556	AAGCACGGCGTGAGT
AR	ENSG00000258930	96	110	-	0.000556	AAGAAGAGAGAGAGA
AR	ENSG00000211535	278	292	+	0.000556	TGGGACAGAGGGGGC
AR	ENSG00000112079	334	348	-	0.000556	AGGGACTCTGAGACA
AR	ENSG00000094880	233	247	-	0.00056	AGGTACTCGCAGCCA
AR	ENSG00000145868	10	24	-	0.000563	AAGCACTGAGTCTGA
AR	ENSG00000111669	11	25	-	0.000566	AGGAACTGCAGGGAC
AR	ENSG00000167553	49	63	+	0.000566	GAGAACAACGCGAGA
AR	ENSG00000100605	357	371	+	0.000566	GGGTACAGAGACGTT
AR	ENSG00000174527	374	388	+	0.000569	GAGAACTGGGCGTGT
AR	ENSG00000128645	357	371	-	0.000569	GGGCAGAGGCTGAGC
AR	ENSG00000125848	263	277	+	0.000569	CAGGACAAGCTCAGC
AR	ENSG00000169184	312	326	+	0.000569	GAGAACAGAAGCGGC
AR	ENSG00000185666	188	202	+	0.000569	GGGCAGAGGCTGAGC
AR	ENSG00000251131	26	40	-	0.000569	GGGAACGCCGGGTGC
AR	ENSG00000128590	22	36	+	0.000569	GAGGACTCACTGGCA
AR	ENSG00000250234	12	26	-	0.000576	CAGCACAGCGTGGAG
AR	ENSG00000250869	147	161	-	0.000576	GGGAAAAGAGAGACC
AR	ENSG00000243341	208	222	+	0.000579	GAGAAGAAGCTGCCC
AR	ENSG00000235939	40	54	-	0.000582	CAGAACGGGAAGTTA
AR	ENSG00000222383	216	230	-	0.000582	TAGCACATTCAGGGT
AR	ENSG00000234062	359	373	-	0.000582	GGGAACACACACATG

AR	ENSG00000186815	79	93	+	0.000585	AAGGACAGAGGGCTG
AR	ENSG00000168807	371	385	+	0.000585	GGGGAGAGCCTGAGC
AR	ENSG00000138385	229	243	+	0.000585	AAGAACGTTCAGAAT
AR	ENSG00000128335	265	279	-	0.000585	GGGCACAGCAGGTGG
AR	ENSG00000112339	144	158	+	0.000585	AAGAACAGGTGCCTC
AR	ENSG00000224011	387	401	+	0.000589	AAGAAGACTGTGCTA
AR	ENSG00000256929	32	46	-	0.000589	GGGAAGAATCTGTAT
AR	ENSG00000162441	44	58	+	0.000592	TAGGACAAAAAGCGA
AR	ENSG00000216642	334	348	+	0.000592	GGGCACTCAAAGAGA
AR	ENSG00000117122	41	55	+	0.000595	AGGCAGAGGGAGATC
AR	ENSG00000078304	169	183	-	0.000595	GGGGACTGAGTCTTT
AR	ENSG00000151445	270	284	+	0.000595	AGGAAAACAAAGAAC
AR	ENSG00000162746	160	174	+	0.000599	GGGGACTCTCAGAGT
AR	ENSG00000234650	159	173	-	0.000599	AAGGACTTCATGGCT
AR	ENSG00000066583	211	225	+	0.000599	GGGAACATGGCGGCT
AR	ENSG00000254673	21	35	-	0.000599	AAGCACAATTAGGAA
AR	ENSG00000228501	210	224	+	0.000602	GAGTACATCCAGGAG
AR	ENSG00000177981	214	228	+	0.000605	AAGCACAAAAGGCCA
AR	ENSG00000225133	352	366	-	0.000609	AAGCACAGCACCTAC
AR	ENSG00000173585	314	328	-	0.000609	TGGGACAGCAGGCAC
AR	ENSG00000137101	371	385	-	0.000609	CGGAAAACAAAGTTC
AR	ENSG00000242219	42	56	-	0.000609	TAGCACAAAAGGTTT
AR	ENSG00000225251	84	98	-	0.000612	CAGGACTTGTTGTTT
AR	ENSG00000072210	302	316	+	0.000616	AGGGACTAGCTCTCC
AR	ENSG00000214283	132	146	+	0.000616	GGGAACTGAAACCAC
AR	ENSG00000164465	160	174	-	0.000616	TAGAAGAGAATGCAC
AR	ENSG00000241279	294	308	-	0.000616	AAGCACATACACCGA
AR	ENSG00000118849	178	192	-	0.000619	GGGGACAATGGGGAC
AR	ENSG00000196542	143	157	-	0.000619	GAGGAGACACTGCAC
AR	ENSG00000132849	317	331	-	0.000622	GGGGACACCCAGGGG
AR	ENSG00000234134	110	124	-	0.000622	CAGAACTGTTTGCCA
AR	ENSG00000139974	337	351	+	0.000622	TGGAACTGGTAGTCA
AR	ENSG00000147650	378	392	-	0.000622	AGGAAAAGCAAGAGC
AR	ENSG00000177182	257	271	-	0.000622	TGGAACAATGAGACG
AR	ENSG00000226800	298	312	+	0.000626	GAGGACAGAGGGATG
AR	ENSG00000021762	71	85	-	0.000629	AAGAAAACAAAGCCC
AR	ENSG00000242257	96	110	+	0.000629	AGGCACTGGATCTTT
AR	ENSG00000230798	364	378	-	0.000633	CGGCACTGGGGGTTC
AR	ENSG00000134824	18	32	+	0.000633	AGGGACTGGTTGCCA
AR	ENSG00000172375	65	79	+	0.000633	AAGAACGGATACTTC
AR	ENSG00000108797	247	261	+	0.000633	GAGGAGAGACAGAGC
AR	ENSG00000131759	9	23	-	0.000633	GGGGACAGGAACAGT
AR	ENSG00000074416	87	101	+	0.000633	GGGCACACGTGGGCC
AR	ENSG00000248710	375	389	+	0.000633	TAGCACAGTCTGGTG
AR	ENSG00000054148	290	304	-	0.000633	CGGTACAAGGAGGCA
AR	ENSG00000231887	317	331	+	0.000636	GGGAAGATATTGTGA
AR	ENSG00000258813	36	50	-	0.000636	GGGAAGAGATTGGAC
AR	ENSG00000253172	375	389	+	0.000636	GAGAACAGTGGGGTG
AR	ENSG00000126522	369	383	+	0.000636	CAGAACTCGGAGCCA
AR	ENSG00000103037	305	319	-	0.00064	AGGAAAAGGCTGGAC

AR	ENSG00000234185	136	150	-	0.00064	AAGGACTTCCTGGGA
AR	ENSG00000123358	138	152	-	0.000643	AAGAAGACACAGCCT
AR	ENSG00000177192	179	193	-	0.000643	GAGAAGAGGCAGGGC
AR	ENSG00000083454	229	243	+	0.000643	CAGCACAGGGCGGTC
AR	ENSG00000206535	136	150	-	0.000643	AAGGACGGCCAGGTC
AR	ENSG00000257076	238	252	-	0.000643	AAGCACTACTAGTCT
AR	ENSG00000164053	77	91	-	0.000647	AAGGACTCAGTGCGG
AR	ENSG00000218014	230	244	-	0.000647	AAGGACTACTTGGCC
AR	ENSG00000065328	142	156	-	0.00065	CAGCACTGACTGGCT
AR	ENSG00000065970	253	267	-	0.00065	GGGCAGACAGTGTCA
AR	ENSG00000108298	312	326	+	0.00065	AGGGACTGTCGGTCT
AR	ENSG00000189050	298	312	-	0.00065	AAGAACAGCGGCATA
AR	ENSG00000198925	366	380	-	0.00065	AGGAACAGCGACCCG
AR	ENSG00000113758	44	58	-	0.00065	AAGCACAGAACGGGA
AR	ENSG00000226059	345	359	+	0.00065	TGGAAGACAATGTCT
AR	ENSG00000116750	327	341	-	0.000654	AGGGACATCCTCGCT
AR	ENSG00000162643	47	61	+	0.000654	GAGCACACAACCTGC
AR	ENSG00000233589	270	284	+	0.000654	GGGTACTTGAAGAAT
AR	ENSG00000251186	89	103	+	0.000654	AGGGAGAGACAGCAC
AR	ENSG00000155508	202	216	+	0.000654	GAGAACACGAGGCGG
AR	ENSG00000084234	99	113	-	0.000657	TGGAAGAGCCTGCTC
AR	ENSG00000215039	299	313	-	0.000661	AGGGAGAGTGAGACC
AR	ENSG00000164404	87	101	+	0.000661	CAGAACATTGGGGAT
AR	ENSG00000225951	262	276	+	0.000661	AGGCACTCCTTCTCC
AR	ENSG00000225733	104	118	-	0.000664	CGGAAGAGGGTGGTC
AR	ENSG00000242583	124	138	+	0.000664	GGGGACATCGGCTCC
AR	ENSG00000159899	207	221	-	0.000664	GAGAACGGGGTGGCA
AR	ENSG00000251821	213	227	+	0.000668	GAGCACATATACTAA
AR	ENSG00000004766	109	123	-	0.000668	AGGGACTACATCTCC
AR	ENSG00000148634	262	276	-	0.000672	GGGAAGAGACTGGGT
AR	ENSG00000258657	89	103	-	0.000672	GGGCACAGTTGGAAT
AR	ENSG00000244676	359	373	+	0.000672	AAGAACTCTAACTGA
AR	ENSG00000172113	29	43	-	0.000672	AAGTACGCGGAGGGC
AR	ENSG00000242683	308	322	+	0.000672	CAGAAGATAGAGACC
AR	ENSG00000253485	167	181	+	0.000672	CAGAACTAAGAGAAA
AR	ENSG00000176973	33	47	+	0.000675	AGGTACTCAGACACC
AR	ENSG00000167548	25	39	+	0.000675	TAGGACTGAGAGGCC
AR	ENSG00000172936	52	66	+	0.000675	AAGAACTGTGGCTCC
AR	ENSG00000078140	363	377	-	0.000675	GAGCACACGAACACT
AR	ENSG00000248636	155	169	+	0.000683	CAGGACACCACGCCC
AR	ENSG00000255185	4	18	+	0.000683	TAGTACTTTGTGATT
AR	ENSG00000241635	189	203	-	0.000683	AAGAACTACAGGCAC
AR	ENSG00000041802	9	23	-	0.000683	CAGCACAGCAACTAA
AR	ENSG00000166526	99	113	+	0.000683	CAGGACACGGTCACT
AR	ENSG00000110536	1	15	-	0.000686	TGGAACTGTGACTCC
AR	ENSG00000182944	157	171	-	0.000686	AGGGAGACGGAGATC
AR	ENSG00000024048	275	289	-	0.000686	AAGGACAGCGGCCTC
AR	ENSG00000233202	119	133	-	0.000686	AAGTAAACGCTGAGC
AR	ENSG00000255692	316	330	+	0.00069	CAGAACATTCACACA
AR	ENSG00000175344	31	45	-	0.00069	AGGTACTCCCGGCGC

AR	ENSG00000206053	251	265 -	0.00069	TGGAACATGTCGACC
AR	ENSG00000237953	227	241 +	0.00069	AAGTACGTTTTGTGA
AR	ENSG00000157107	105	119 +	0.00069	GGGGACAGGGCGCAT
AR	ENSG00000244307	257	271 -	0.00069	CAGAACTCCTAGATT
AR	ENSG00000143353	67	81 +	0.000694	CAGTACAGTTACAAC
AR	ENSG00000129472	207	221 +	0.000694	GAGAAGAAGAAGATC
AR	ENSG00000229920	303	317 -	0.000694	AGGTACTTTCTCAAC
AR	ENSG00000064787	61	75 -	0.000694	CAGAACACCACCTCC
AR	ENSG00000238278	130	144 -	0.000694	GAGGACTCAGAGGCT
AR	ENSG00000173210	74	88 +	0.000694	CAGGACACAAGGCCT
AR	ENSG00000055609	229	243 -	0.000694	CAGGACACGCACTCA
AR	ENSG00000208038	184	198 +	0.000697	GGGCACAGGCCCTCC
AR	ENSG00000198417	275	289 +	0.000697	AGGCACAAAGCGCCT
AR	ENSG00000196455	67	81 -	0.000697	GAGAAAATGATGCTC
AR	ENSG00000186792	206	220 +	0.000701	GGGTAGAGACTGAGT
AR	ENSG00000013810	107	121 +	0.000701	AGGCACAGCTTCCCA
AR	ENSG00000116783	372	386 -	0.000705	GGGAAGTGACAGTCC
AR	ENSG00000174564	223	237 +	0.000705	GAGGACATGAAGGGG
AR	ENSG00000105778	366	380 -	0.000705	CAGCACATGAGGGCC
AR	ENSG00000182473	294	308 +	0.000709	CGGCACAGCCACGTC
AR	ENSG00000171497	72	86 +	0.000709	AGGAACAGGTGGCGG
AR	ENSG00000136108	96	110 -	0.000712	TGGGACTGAGTCTGC
AR	ENSG00000126351	208	222 +	0.000712	CAGCACAAAGAGAAG
AR	ENSG00000164414	357	371 +	0.000712	TAGGACAAAGAGGCA
AR	ENSG00000225920	122	136 +	0.000716	AGGAACACTACCCCC
AR	ENSG00000161642	82	96 -	0.000716	GGGCACTCAGGGCTC
AR	ENSG00000168883	98	112 +	0.000716	CGGGACGTGGAGTCC
AR	ENSG00000151093	329	343 -	0.000716	GAGAAAAGGAAGTTT
AR	ENSG00000219790	241	255 -	0.000716	TGGGACATTCAGGGA
AR	ENSG00000256650	175	189 -	0.00072	AGGTACATGCACTAG
AR	ENSG00000223442	60	74 +	0.00072	GGGGAAATGGTGTCC
AR	ENSG00000208892	219	233 +	0.000724	AGGGACTTTTTGTTG
AR	ENSG00000250299	248	262 -	0.000724	GGGGACAAAGGGTGG
AR	ENSG00000140525	161	175 +	0.000724	TGGTACACCCCGCCC
AR	ENSG00000164542	353	367 +	0.000724	GAGAAAAGTGAGTCT
AR	ENSG00000104529	268	282 +	0.000724	AGGCACTTGTAGGCC
AR	ENSG00000122986	162	176 -	0.000727	AGGTAAACACTGTCA
AR	ENSG00000258283	177	191 +	0.000727	AGGAAGACGATGCCA
AR	ENSG00000236449	174	188 +	0.000727	AGGGACTCTCAGGCT
AR	ENSG00000235374	86	100 +	0.000727	GAGGAGACTCTGCCC
AR	ENSG00000180385	21	35 +	0.000727	GGGCACAGTTGCTTC
AR	ENSG00000171056	53	67 -	0.000727	AGGGACTGAGGGGGC
AR	ENSG00000205268	311	325 +	0.000727	CAGAACACAAGGAAG
AR	ENSG00000254093	90	104 -	0.000727	AGGGACTGAGGGGGC
AR	ENSG00000228932	210	224 -	0.000727	GGGGAGATGCTGTTT
AR	ENSG00000173457	190	204 +	0.000731	AGGAAGAGCGAGACA
AR	ENSG00000256028	29	43 +	0.000731	GGGAACTTAGAGCGG
AR	ENSG00000003509	304	318 -	0.000731	CGGCACACAACGGCC
AR	ENSG00000115816	166	180 +	0.000731	CGGCACACAACGGCC
AR	ENSG00000125991	221	235 +	0.000731	CGGAAGAGGGAGTCT

AR	ENSG00000113282	214	228	-	0.000731	CAGCACAGGCGCTGC
AR	ENSG00000197071	32	46	-	0.000731	AAGAACTGCAGCTGC
AR	ENSG00000246889	23	37	+	0.000735	GGGAAGAAGCAGAGC
AR	ENSG00000175197	222	236	+	0.000735	AGGCACTGAGCGTAT
AR	ENSG00000240216	256	270	-	0.000735	GGGGACTAAATGGTT
AR	ENSG00000143379	139	153	-	0.000739	GAGAAGAGACTGGCA
AR	ENSG00000231882	240	254	+	0.000739	TGGCACAATCTCAGC
AR	ENSG00000164466	230	244	+	0.000739	TGGCACAATCTCAGC
AR	ENSG00000152061	303	317	-	0.000743	GGGAACTGAGAGGGG
AR	ENSG00000175224	377	391	+	0.00075	GAGGACTAAAAGAAT
AR	ENSG00000214188	355	369	+	0.00075	CGGAACTCCGAGGGT
AR	ENSG00000115415	298	312	+	0.000754	CAGGACAAGATCTGA
AR	ENSG00000145819	371	385	+	0.000754	CGGAACAGCAGCACC
AR	ENSG00000147316	302	316	+	0.000754	AGGTACTTCCTGCTG
AR	ENSG00000159214	295	309	-	0.000758	AAGCACTTTGGGCTC
AR	ENSG00000255773	317	331	-	0.000758	CAGGACAGCAGGCCT
AR	ENSG00000143375	37	51	+	0.000762	GAGAACAAAATCCGG
AR	ENSG00000100442	378	392	-	0.000762	CAGTACACGAGGAAA
AR	ENSG00000100600	47	61	-	0.000762	AGGTACTTGAGGTCT
AR	ENSG00000100926	124	138	+	0.000762	GAGGACGGATTGGGC
AR	ENSG00000007384	123	137	-	0.000762	CAGAACATCTGGAGA
AR	ENSG00000123388	178	192	-	0.000766	AAGTAGATATTGTCA
AR	ENSG00000256804	94	108	+	0.000766	GGGTACAGCGGCCTC
AR	ENSG00000152082	260	274	+	0.000766	AGGTACTTTCTCCCC
AR	ENSG00000147687	274	288	-	0.000766	AAGGACTTCGGGAAC
AR	ENSG00000172590	244	258	-	0.00077	GAGAACAGTCCCTAA
AR	ENSG00000145040	355	369	+	0.00077	GGGGACTCTGGGATC
AR	ENSG00000240758	267	281	-	0.00077	GGGAACAGCCGGGAG
AR	ENSG00000239737	152	166	+	0.00077	AGGTAGATGCAGCTC
AR	ENSG00000188818	294	308	+	0.000774	GGGCACTGGGTCCTC
AR	ENSG00000223969	327	341	-	0.000774	CGGTACTTCGGGTCC
AR	ENSG00000148303	27	41	-	0.000774	GGGAAGAGAGACTGC
AR	ENSG00000076258	87	101	-	0.000778	CAGAACATAAACGCT
AR	ENSG00000154134	238	252	-	0.000778	GGGTACGGTCGGTGC
AR	ENSG00000231125	69	83	+	0.000778	AGGAAAATGGAGTCT
AR	ENSG00000109689	314	328	+	0.000778	GAGAAGAGGGAGATA
AR	ENSG00000250697	328	342	-	0.000778	GAGCACACCAACAGA
AR	ENSG00000234141	386	400	-	0.000778	GGGAAGAGAAAGAGA
AR	ENSG00000186106	182	196	+	0.000778	GGGGACTCCGACTAC
AR	ENSG00000152457	140	154	+	0.000782	CAGAAGAGAGAGGGC
AR	ENSG00000164742	248	262	+	0.000782	AGGTACTCGAGGTCA
AR	ENSG00000214050	260	274	+	0.000782	GAGAAAAAGCTGACC
AR	ENSG00000197620	380	394	-	0.000782	CGGGACTGACAGAAA
AR	ENSG00000158859	289	303	+	0.000786	AGGCAGAGACAGGGC
AR	ENSG00000227802	255	269	-	0.000786	TGGCACAGCGAGCGG
AR	ENSG00000088833	349	363	-	0.000786	GAGAAGAGTCAGCTT
AR	ENSG00000121716	352	366	-	0.000786	CAGTACATGCAGGTG
AR	ENSG00000214014	321	335	+	0.000786	TGGCACAGGCACTGA
AR	ENSG00000162458	296	310	+	0.00079	TGGAACAGGTGGCCT
AR	ENSG00000167491	86	100	-	0.00079	CAGGACTCCCAGGAC

AR	ENSG00000254901	378	392	+	0.00079	AGGAAAAGGGGGTTC
AR	ENSG00000122432	170	184	-	0.000794	TGGAACAGAGGGCTG
AR	ENSG00000020577	162	176	+	0.000794	AGGAACTCGGGCTGC
AR	ENSG00000124208	213	227	+	0.000794	GAGAACTGGCCGGGC
AR	ENSG00000250963	150	164	-	0.000794	GAGTACTGCCTGCAG
AR	ENSG00000129084	292	306	+	0.000798	CGGGACTGAAAGAGA
AR	ENSG00000258034	380	394	+	0.000798	CAGGACAGAGCGTTG
AR	ENSG00000171723	330	344	+	0.000798	AGGCACTGACTGGAG
AR	ENSG00000164808	40	54	-	0.000798	GAGTAAAGTCTGTGT
AR	ENSG00000143321	335	349	+	0.000802	AGGTACTTGAGGTTA
AR	ENSG00000110851	77	91	+	0.000802	AGGAACAGCAGCCCT
AR	ENSG00000171345	291	305	-	0.000802	AAGGACGACGTGGCC
AR	ENSG00000187514	245	259	+	0.000802	AGGGAGACTCAGTCT
AR	ENSG00000168028	199	213	+	0.000802	GAGTACATAAGGACG
AR	ENSG00000133872	222	236	-	0.000802	GAGCACAGCGCGGCT
AR	ENSG00000136492	113	127	+	0.000806	CGGGACTGGTTGATT
AR	ENSG00000255046	291	305	-	0.000806	AAGAAAACAAAGGCC
AR	ENSG00000227617	328	342	+	0.00081	GGGAAGAATTTGACC
AR	ENSG00000112655	243	257	+	0.00081	AGGTACTGGGCGCGC
AR	ENSG00000225839	267	281	+	0.00081	AGGCACAGTGGCTCA
AR	ENSG00000257918	192	206	-	0.000814	GAGTACACTGGCCTC
AR	ENSG00000173137	349	363	+	0.000814	GGGGACGGTGAGGTC
AR	ENSG00000158089	384	398	+	0.000818	GGGAAAATAAAGAAC
AR	ENSG00000196208	257	271	+	0.000818	AAGGACAGAGACCTG
AR	ENSG00000072121	307	321	-	0.000822	AGGGACGCTCTCTGC
AR	ENSG00000067601	281	295	+	0.000822	CGGTACATGCAGGTG
AR	ENSG00000129083	97	111	+	0.000826	AAGCACTTTCTCCCC
AR	ENSG00000258966	363	377	+	0.000826	CAGGACAGAGACCAT
AR	ENSG00000257534	159	173	-	0.000831	CAGCACAGCTCGTTA
AR	ENSG00000230870	134	148	+	0.000831	TGGAACTAACACTTC
AR	ENSG00000204209	306	320	-	0.000835	AGGAAGATAAAGAAA
AR	ENSG00000139637	203	217	-	0.000839	CGGAAGAGGAAGCGC
AR	ENSG00000225187	290	304	-	0.000839	GAGGACGGAGTGGCT
AR	ENSG00000233733	161	175	-	0.000839	AGGCAGAGAAAGGGC
AR	ENSG00000225673	233	247	+	0.000839	CAGAACACTCTCCTG
AR	ENSG00000130997	258	272	+	0.000839	CGGAACTTCCTGAGG
AR	ENSG00000010030	340	354	+	0.000839	GAGCACAGCCGCTCT
AR	ENSG00000149716	291	305	-	0.000843	AGGCACAAATGCTCC
AR	ENSG00000183426	18	32	+	0.000843	GAGCACGTGATGGAC
AR	ENSG00000214274	364	378	-	0.000847	GAGCACTGCCTCCAC
AR	ENSG00000099337	227	241	+	0.000847	TGGAACTAGGTGCCA
AR	ENSG00000224680	374	388	+	0.000852	GAGGACGGTCTCTGC
AR	ENSG00000235910	381	395	-	0.000852	AAGGAGACAGAGGGC
AR	ENSG00000258051	26	40	+	0.000852	AAGTAGACTGTGAGA
AR	ENSG00000222750	207	221	-	0.000852	CGGTACTGCCACTGC
AR	ENSG00000248487	222	236	-	0.000852	AGGTACTTAAGGGGC
AR	ENSG00000176076	53	67	-	0.000852	GGGGACAGCTGGTGG
AR	ENSG00000151465	340	354	-	0.000856	AGGAAGACCGAGAAA
AR	ENSG00000118894	282	296	+	0.000856	GAGAACGCGGGGACC
AR	ENSG00000221025	184	198	-	0.000856	GGGCAGAGCCAGCTC

AR	ENSG00000168575	40	54	+	0.000856	AGGGAGAAAATGTAA
AR	ENSG00000102572	283	297	-	0.00086	GGGAAGAGAATGGAA
AR	ENSG00000182909	364	378	-	0.00086	GGGAACTGGCACGCC
AR	ENSG00000115239	51	65	-	0.00086	GGGAACTCAAGCTCC
AR	ENSG00000204264	97	111	+	0.00086	CAGAACAGTAGGCGG
AR	ENSG00000153993	126	140	-	0.00086	TAGGACAGGCGGAGA
AR	ENSG00000224437	289	303	-	0.00086	GGGGACACCACCTCC
AR	ENSG00000185736	349	363	+	0.000864	GAGAACAATTCGAGA
AR	ENSG00000134780	125	139	+	0.000864	AAGGACGCAGCGTCC
AR	ENSG00000175773	277	291	-	0.000869	AAGGAGACGGAGTCA
AR	ENSG00000171121	264	278	+	0.000869	TAGAACTGACCGCTC
AR	ENSG00000135317	271	285	-	0.000869	AAGGACAGCCCCAGC
AR	ENSG00000196126	75	89	+	0.000869	AAGGAAATTCTGTTT
AR	ENSG00000134905	309	323	+	0.000873	GGGGACAGCGTCAGG
AR	ENSG00000154889	375	389	+	0.000873	AGGCACGGCCTGGCT
AR	ENSG00000100142	209	223	+	0.000873	AGGAAAATAGTGCTA
AR	ENSG00000145907	22	36	-	0.000873	AGGCACTGGCGGGGC
AR	ENSG00000258953	232	246	+	0.000873	CAGAAAACTCTGTCT
AR	ENSG00000205231	236	250	-	0.000877	AGGCACAGAGGGGAG
AR	ENSG00000103051	207	221	-	0.000877	CGGCACTTCCGGTCC
AR	ENSG00000048828	232	246	+	0.000877	CAGCACATGGCGGCC
AR	ENSG00000136842	188	202	+	0.000877	TGGAACTACGTGTAG
AR	ENSG00000223787	183	197	-	0.000882	CAGCACTCTGTGGGT
AR	ENSG00000241343	79	93	+	0.000882	GGGGACGGGGAGGCC
AR	ENSG00000111775	90	104	+	0.000886	AAGGACTCCGTCGTC
AR	ENSG00000216324	163	177	+	0.000886	GAGAAAAACCTGTTA
AR	ENSG00000215472	164	178	+	0.00089	AAGTACGTATTGACA
AR	ENSG00000221656	360	374	-	0.000895	GGGCACATCCGGGGA
AR	ENSG00000121753	158	172	-	0.000899	GAGGACTGTGGGTAA
AR	ENSG00000134398	221	235	+	0.000899	GAGGACTCCAGGAAC
AR	ENSG00000185513	158	172	-	0.000899	CAGGACGCAGAGCGC
AR	ENSG00000185963	346	360	-	0.000899	TGGGACAGCCGCTTC
AR	ENSG00000110011	72	86	+	0.000903	AAGAACTGTGCCTCC
AR	ENSG00000114395	182	196	-	0.000903	AGGAACGAGGAGCTA
AR	ENSG00000241830	287	301	-	0.000903	AGGCACTACAACTGC
AR	ENSG00000142937	34	48	+	0.000908	CAGAACAGCTCCTAC
AR	ENSG00000087470	154	168	+	0.000908	CAGAACTACAACTCC
AR	ENSG00000136367	32	46	+	0.000908	CGGAACTTCAGGTCT
AR	ENSG00000241839	226	240	+	0.000908	CAGAACGCGGAGAGT
AR	ENSG00000254531	170	184	-	0.000908	GGGGACAATGAGGAG
AR	ENSG00000213397	336	350	+	0.000908	AAGCAGAGCAAGGTC
AR	ENSG00000224489	250	264	+	0.000908	AGGTACTACCTCTCT
AR	ENSG00000257755	234	248	+	0.000908	CAGCAGATCCAGTGC
AR	ENSG00000116791	262	276	-	0.000912	TGGTAGAGCTTGTGC
AR	ENSG00000162623	382	396	+	0.000912	TGGTAGAGCTTGTGC
AR	ENSG00000255448	324	338	+	0.000912	GAGAAGATGAGGTAC
AR	ENSG00000227973	259	273	-	0.000912	CAGCACTGTCACTCC
AR	ENSG00000167397	218	232	+	0.000912	AAGTAGAGACTGGGA
AR	ENSG00000171302	70	84	-	0.000912	TGGCACATGAGGGCC
AR	ENSG00000244097	162	176	+	0.000912	CGGAAGACAAAGGTC

AR	ENSG00000253213	369	383	+	0.000912	AGGAAAAAGGAGAGC
AR	ENSG00000253729	303	317	+	0.000912	CAGGAGACCTTGTCC
AR	ENSG00000065150	200	214	+	0.000917	CAGAACTGTGGGATT
AR	ENSG00000166200	353	367	+	0.000917	TGGAACTCTGAGGGT
AR	ENSG00000222019	298	312	+	0.000917	GGGCACGAGGTGGTC
AR	ENSG00000083535	304	318	-	0.000921	GAGGACAGAACCAGC
AR	ENSG00000196712	217	231	-	0.000921	GGGGAGAGGGAGCGC
AR	ENSG00000241550	81	95	-	0.000921	GAGTACTGGTTCTTT
AR	ENSG00000201448	351	365	-	0.000925	AAGAAGAGGGTCTAT
AR	ENSG00000236810	233	247	-	0.000925	TGGAACAGCAGCAGC
AR	ENSG00000185278	229	243	-	0.00093	GGGAAGAAGGTGATA
AR	ENSG00000235848	235	249	-	0.00093	TGGAAGATAAAGAGC
AR	ENSG00000240695	222	236	+	0.00093	CGGGACTTCGTGTGG
AR	ENSG00000243305	228	242	-	0.00093	CAGGACTATATGACT
AR	ENSG00000008130	167	181	-	0.000934	GGGCACTGCCTGCAG
AR	ENSG00000116213	9	23	+	0.000934	GGGGACAGCAGGAGG
AR	ENSG00000124920	298	312	+	0.000934	CGGGACATGGAGGTG
AR	ENSG00000244926	218	232	+	0.000934	AAGAAAACATTGAGT
AR	ENSG00000212496	90	104	-	0.000934	TAGTACAATTAGCAA
AR	ENSG00000224884	242	256	-	0.000934	AAGAAGAAAGAGAAA
AR	ENSG00000134970	47	61	+	0.000934	AAGAAAAAGAAGAGC
AR	ENSG00000102024	385	399	+	0.000934	AGGAACGTGCGGCGC
AR	ENSG00000107771	166	180	+	0.000939	CGGGACTGCGCGTGC
AR	ENSG00000201199	25	39	+	0.000939	AGGAAGAACATGCAT
AR	ENSG00000182177	134	148	+	0.000943	AGGGACTGTAAGAGG
AR	ENSG00000117419	286	300	-	0.000948	AGGCAGAGGCAGGGC
AR	ENSG00000122406	272	286	-	0.000948	CGGAACAGAGACCGG
AR	ENSG00000226763	176	190	+	0.000948	AGGAAAACTGAGGCC
AR	ENSG00000115419	376	390	-	0.000948	GGGAACGCGGTCTGT
AR	ENSG00000134326	249	263	+	0.000948	AGGCAGAGGGTGCTA
AR	ENSG00000114503	342	356	-	0.000948	CGGTACTGGCTCAGC
AR	ENSG00000127423	1	15	-	0.000952	GAGGAGTGCGTGTTC
AR	ENSG00000162889	51	65	-	0.000952	GAGGACGCGCGGTGC
AR	ENSG00000184669	129	143	+	0.000952	GGGAACGGGGAGGGA
AR	ENSG00000207185	214	228	+	0.000952	CAGCACAGGTACTAA
AR	ENSG00000243420	267	281	-	0.000952	AAGAACTCCTGGACT
AR	ENSG00000119711	9	23	-	0.000952	GAGCACACGGGGAAG
AR	ENSG00000179583	222	236	-	0.000952	AAGCACACAGCCTCA
AR	ENSG00000131508	345	359	+	0.000952	AGGAACTGGCACTTG
AR	ENSG00000256326	133	147	-	0.000952	AGGAAAACTCTGCTA
AR	ENSG00000236842	147	161	-	0.000957	CGGAACTGTGCGCCC
AR	ENSG00000254772	135	149	+	0.000957	CAGAACTTCCTGCAG
AR	ENSG00000124302	232	246	-	0.000957	CAGCACTGCCGGATC
AR	ENSG00000175110	27	41	-	0.000957	AGGGACACTCTCCAG
AR	ENSG00000147874	327	341	+	0.000957	AAGGACGCATTGCAT
AR	ENSG00000242941	268	282	-	0.000962	GAGCAGATGTAGTAC
AR	ENSG00000255730	202	216	+	0.000962	AAGGAAAGGCAGAGC
AR	ENSG00000127399	124	138	-	0.000962	CGGAACTGAGGCTTC
AR	ENSG00000168066	321	335	+	0.000966	AAGGACTGAGGCTCC
AR	ENSG00000126803	211	225	-	0.000966	AGGCACATGGCGGCA

AR	ENSG00000087111	174	188	+	0.000966	AAGAACGGCCTCATT
AR	ENSG00000248394	358	372	+	0.000966	TGGTACAGCTTCATT
AR	ENSG00000159079	130	144	-	0.000971	CAGAACTGAGTCTTG
AR	ENSG00000224502	103	117	-	0.000971	AGGCACGTGTTGGGC
AR	ENSG00000120068	97	111	-	0.000975	CAGAACGCAGAGCGA
AR	ENSG00000141753	30	44	+	0.000975	AAGGACTTTCAGATG
AR	ENSG00000188001	282	296	-	0.000975	GAGCACGTGGTGCGT
AR	ENSG00000119922	125	139	+	0.00098	GAGGAAAAAGAGTCC
AR	ENSG00000078487	219	233	-	0.00098	GGGAAGAGGAAGTTG
AR	ENSG00000143499	321	335	+	0.000984	GGGCACAGCCGGCGG
AR	ENSG00000196498	3	17	+	0.000984	GGGGACGCGCAGGGC
AR	ENSG00000244684	150	164	-	0.000984	AAGAAAAAAGTGAGA
AR	ENSG00000212464	362	376	-	0.000989	AAGGACTTAAACTTA
AR	ENSG00000111752	37	51	-	0.000989	TAGAACAATGGGGCT
AR	ENSG00000206567	321	335	+	0.000989	GGGCACTCTCTGCGG
AR	ENSG00000169239	342	356	+	0.000989	TGGAACTCATTGGAA
AR	ENSG00000252150	236	250	-	0.000994	AAGAAAATGGTGCAT
AR	ENSG00000072518	236	250	-	0.000998	GGGGACGCGCTCTCC
AR	ENSG00000150893	312	326	-	0.000998	AGGAACTCCGCGCGT
AR	ENSG00000164306	319	333	-	0.000998	AGGAAGACCTAGATT
AR	ENSG00000232940	257	271	-	0.000998	GAGCACACCCGCCGC
