# Supplementary material for: Prosaposin activates the androgen receptor and potentiates resistance to endocrine treatment in breast cancer
Source: Breast Cancer Res. 2015 Sep 4;17(1):123. doi: 10.1186/s13058-015-0636-6 (PMC4560928; doi:10.1186/s13058-015-0636-6)
Supplement: Additional file 6: — Table: Estrogen receptor (ER) motif results ( p <0.001). A 400-bp-sized window surrounding starting sites of HOXC11 target genes was selected for ER motif searching. The searching process was performed using the FIMO program available in MEME-suite with the p value significant cutoff set at 0.001. (PDF 198 kb) [file 13058_2015_636_MOESM6_ESM.pdf]

| motif | gene | start | stop | strand | p.value | matched.sequence |
|---|---|---|---|---|---|---|
| ESR1 | ENSG00000227200 | 266 | 285 | - | 1.51E-08 | CACCCAGGTCAACATGACCC |
| ESR1 | ENSG00000105675 | 20 | 39 | - | 1.30E-07 | CCCCCATGTCAACCTGCCCT |
| ESR1 | ENSG00000253302 | 110 | 129 | + | 1.47E-07 | CTCCAAGGGCACCCAGCCCT |
| ESR1 | ENSG00000155636 | 115 | 134 | - | 2.02E-07 | GACGCGGGGGACCCTGACCC |
| ESR1 | ENSG00000050426 | 302 | 321 | - | 2.38E-07 | CGCGGAGGGCACCCTGTCCT |
| ESR1 | ENSG00000221585 | 104 | 123 | - | 3.09E-07 | CTGCCGGGTGACCTTGCCCT |
| ESR1 | ENSG00000141524 | 270 | 289 | - | 3.53E-07 | GGCCCCGGCCTCCCTGACCC |
| ESR1 | ENSG00000258388 | 69 | 88 | + | 4.42E-07 | CCTCCAGGCCACCCTGCCAC |
| ESR1 | ENSG00000165182 | 123 | 142 | + | 6.34E-07 | CGGCAGGGTCACCCCGGCCT |
| ESR1 | ENSG00000222019 | 314 | 333 | - | 1.08E-06 | GGGGCCGGTCACCCCGCCCC |
| ESR1 | ENSG00000167165 | 233 | 252 | - | 1.12E-06 | AGGGAGGGTCACACTGGCCC |
| ESR1 | ENSG00000167635 | 196 | 215 | + | 1.19E-06 | CGCCCAGGACTCCCAGACCC |
| ESR1 | ENSG00000255529 | 217 | 236 | + | 1.30E-06 | GTCCACGGTCACCCTGCTCT |
| ESR1 | ENSG00000202031 | 175 | 194 | + | 1.79E-06 | TGGGGAGGGCAGCCTGACTC |
| ESR1 | ENSG00000178550 | 196 | 215 | - | 1.88E-06 | GAGCAGGGGCAGCATGGCCT |
| ESR1 | ENSG00000250012 | 155 | 174 | + | 2.02E-06 | AGGCCGGGCCTCCCTGGCCC |
| ESR1 | ENSG00000196557 | 1 | 20 | + | 2.27E-06 | CGCCCCGGCCACGCTGGCCC |
| ESR1 | ENSG00000257108 | 358 | 377 | + | 2.29E-06 | GTCAGAAGTCTCCCTGACCT |
| ESR1 | ENSG00000100934 | 239 | 258 | + | 2.52E-06 | GGCGCATGTCACCATGCCCG |
| ESR1 | ENSG00000107331 | 102 | 121 | - | 2.69E-06 | GACAGAGGACAGGCTGACCC |
| ESR1 | ENSG00000204622 | 219 | 238 | - | 2.74E-06 | GGTTCGGGGCGCCATGACCC |
| ESR1 | ENSG00000132849 | 369 | 388 | + | 2.75E-06 | TTCCCGGGCCTGCCTGCCCT |
| ESR1 | ENSG00000117632 | 225 | 244 | - | 3.03E-06 | CCTGCAGGGCCCCCTGCCCC |
| ESR1 | ENSG00000203896 | 336 | 355 | + | 3.06E-06 | ACCCAGGGTGGCCCTGCCCC |
| ESR1 | ENSG00000127022 | 24 | 43 | + | 3.19E-06 | CCCCGAGGCCACGCAGACCT |
| ESR1 | ENSG00000139350 | 299 | 318 | - | 3.46E-06 | GGGCGGGGGCCCGCTGACCT |
| ESR1 | ENSG00000185278 | 6 | 25 | - | 3.67E-06 | TGCTAAGGTCACCCTGAATT |
| ESR1 | ENSG00000126351 | 254 | 273 | + | 3.83E-06 | AAGGGAGGGCAGCCTGCCAT |
| ESR1 | ENSG00000189410 | 13 | 32 | - | 3.85E-06 | GGAGCGGGGCACCCTGACAG |
| ESR1 | ENSG00000100399 | 194 | 213 | + | 3.89E-06 | AGGCGGGGACAGCCTGTCCC |
| ESR1 | ENSG00000138442 | 256 | 275 | - | 3.98E-06 | GCTTCAGGTCACCCCGCCCA |
| ESR1 | ENSG00000157734 | 119 | 138 | - | 4.01E-06 | CCCCCAGGCGACCCCGCCCC |
| ESR1 | ENSG00000132510 | 134 | 153 | - | 4.08E-06 | TTTCCTGGGCTCCCTGCCCC |
| ESR1 | ENSG00000186862 | 260 | 279 | - | 4.10E-06 | GTGCCTGGTCGGCCTCACCT |
| ESR1 | ENSG00000247275 | 302 | 321 | - | 4.76E-06 | GGGTTAGGTCAGCATGATCC |
| ESR1 | ENSG00000116701 | 321 | 340 | + | 4.93E-06 | TGCCAAGGTTACCCTGGCTC |
| ESR1 | ENSG00000258136 | 127 | 146 | - | 4.93E-06 | TGCTTTGGCCACTCTGACCC |
| ESR1 | ENSG00000135709 | 332 | 351 | - | 5.01E-06 | CTCCCGGGTCATCCTTCCCC |
| ESR1 | ENSG00000117983 | 118 | 137 | - | 5.07E-06 | CACGCAGGGCACCATGCTCT |
| ESR1 | ENSG00000196372 | 153 | 172 | + | 5.10E-06 | GGCCCAGCGCAGACTGGCCC |
| ESR1 | ENSG00000182934 | 90 | 109 | - | 5.10E-06 | GGGCCGGGGCCTCTTGACCC |
| ESR1 | ENSG00000125954 | 30 | 49 | + | 5.91E-06 | TGCCAAGGAGACTGTGCCCT |
| ESR1 | ENSG00000004399 | 211 | 230 | - | 6.04E-06 | GGCCCGGGTCAGCCTCGCCG |
| ESR1 | ENSG00000202058 | 203 | 222 | - | 6.25E-06 | CAGCCAGATCACCCTCACAT |
| ESR1 | ENSG00000200091 | 203 | 222 | - | 6.25E-06 | CAGCCAGATCACCCTCACAT |
| ESR1 | ENSG00000167741 | 320 | 339 | - | 6.50E-06 | GCTGCATGTGAGCCTGCCCC |
| ESR1 | ENSG00000134255 | 338 | 357 | - | 6.61E-06 | GGCTCAGCTCCCCGTGCCCT |
| ESR1 | ENSG00000162746 | 267 | 286 | - | 6.61E-06 | GAACCGGGGCACCTTGATCC |
| ESR1 | ENSG00000237317 | 228 | 247 | - | 6.68E-06 | TGACAAGGTCACTGTGGCTT |

| | | | | | |
|---|---|---|---|---|---|
| ESR1 | ENSG00000143627 | 115 | 134 - | 6.80E-06 | CCAGTAGGCCACCCTGTCCC |
| ESR1 | ENSG00000214597 | 190 | 209 + | 6.91E-06 | CTCCCTGGCCTCGCTGCCCT |
| ESR1 | ENSG00000122778 | 303 | 322 + | 7.11E-06 | CGCCGGGGTCGCGCTGGCCC |
| ESR1 | ENSG00000177731 | 113 | 132 + | 7.22E-06 | AGCGCAGGTGACGGTGCCTC |
| ESR1 | ENSG00000111780 | 287 | 306 - | 7.47E-06 | GGCCAAGGTCACAGTCCCTC |
| ESR1 | ENSG00000163812 | 4 | 23 - | 7.47E-06 | CGCCGGGTTCAGCCTGGCCC |
| ESR1 | ENSG00000197102 | 188 | 207 - | 7.55E-06 | CTGCCAGGTGGCCCCGCCCC |
| ESR1 | ENSG00000106069 | 320 | 339 - | 7.55E-06 | ATGTCTGATCACCCTGGCCT |
| ESR1 | ENSG00000148814 | 100 | 119 + | 7.72E-06 | GCGCAGGGGCACCCACACCC |
| ESR1 | ENSG00000185728 | 243 | 262 - | 7.76E-06 | GCTCCGAGCCTCCCTGACCT |
| ESR1 | ENSG00000132010 | 319 | 338 + | 7.94E-06 | GCCCCGGGTCCCTCTGCCAC |
| ESR1 | ENSG00000185507 | 336 | 355 + | 7.98E-06 | CCGTGGGGGCTCCGTGACCC |
| ESR1 | ENSG00000169062 | 61 | 80 - | 8.25E-06 | CCGCCGGGTCAGGCGGCCCC |
| ESR1 | ENSG00000236472 | 375 | 394 - | 8.57E-06 | AACCTGGGGCCTCCTGACCT |
| ESR1 | ENSG00000165238 | 188 | 207 - | 8.57E-06 | GGCCAAGGACACACAGGCCC |
| ESR1 | ENSG00000229732 | 345 | 364 - | 8.62E-06 | CTCTCAGGACCCCCTGTCCT |
| ESR1 | ENSG00000130638 | 73 | 92 + | 8.67E-06 | AACACAGGTCCCCCTCCCCC |
| ESR1 | ENSG00000187144 | 248 | 267 - | 9.00E-06 | GGCAAGGGGCTGTGTGACCT |
| ESR1 | ENSG00000213145 | 43 | 62 - | 9.00E-06 | AGCCCAGGTCACTGTCAGCC |
| ESR1 | ENSG00000161800 | 273 | 292 + | 9.20E-06 | TAGCCAAGCCTCCCTGCCCC |
| ESR1 | ENSG00000115504 | 320 | 339 + | 9.20E-06 | ACACAAGGTTAGCATGCCCC |
| ESR1 | ENSG00000167972 | 37 | 56 - | 9.46E-06 | GGCAGAGGGCGCTCTGACTT |
| ESR1 | ENSG00000012963 | 97 | 116 - | 9.61E-06 | GCGCCGGGTGACGGCGACCC |
| ESR1 | ENSG00000213689 | 37 | 56 - | 9.72E-06 | CTTGGGGGTCCCCCTGCCCT |
| ESR1 | ENSG00000104529 | 143 | 162 - | 9.72E-06 | GGCCCCAGCCAGTCTGCCCT |
| ESR1 | ENSG00000129810 | 91 | 110 + | 9.82E-06 | CCGGCGGGTCGCCGAGACCC |
| ESR1 | ENSG00000110619 | 64 | 83 + | 9.88E-06 | TCGCCAGCACACCCTGTCCC |
| ESR1 | ENSG00000178836 | 184 | 203 + | 9.88E-06 | GGCCCAGGTCGCCAAGCCTC |
| ESR1 | ENSG00000168575 | 363 | 382 + | 9.88E-06 | GGGCCGGGTGACGCGGCCCC |
| ESR1 | ENSG00000256500 | 69 | 88 + | 1.01E-05 | CGCCCGGGTCAGCCCGCGCC |
| ESR1 | ENSG00000186106 | 382 | 401 - | 1.01E-05 | AAGCCCGGGCACCCTCGCCC |
| ESR1 | ENSG00000248636 | 151 | 170 + | 1.02E-05 | GCACCAGGACACCACGCCCC |
| ESR1 | ENSG00000126777 | 140 | 159 - | 1.04E-05 | AGGGCAAGTCACTTTGACAC |
| ESR1 | ENSG00000254901 | 226 | 245 - | 1.04E-05 | CGGCCAAGGTACGCTGCCCC |
| ESR1 | ENSG00000164142 | 263 | 282 - | 1.04E-05 | GGCCCAGGTCCTCCAGGCCC |
| ESR1 | ENSG00000258545 | 110 | 129 - | 1.05E-05 | GGCCTGGGCCTGCCTGGCCT |
| ESR1 | ENSG00000225187 | 379 | 398 + | 1.06E-05 | TGCAAGGCCCAGCCTGACCT |
| ESR1 | ENSG00000134490 | 324 | 343 + | 1.07E-05 | GTCTCTGGTCGGCCTCACCT |
| ESR1 | ENSG00000246731 | 7 | 26 + | 1.08E-05 | CGGCCGGGGCCCCGTGCCCG |
| ESR1 | ENSG00000240801 | 32 | 51 - | 1.09E-05 | GAAGGCGGCCACCCTGCCCC |
| ESR1 | ENSG00000139116 | 273 | 292 + | 1.09E-05 | GGGGCAGGGCGCACTCACCC |
| ESR1 | ENSG00000110011 | 173 | 192 - | 1.11E-05 | CGGCCCGGCCACCGTGTCTC |
| ESR1 | ENSG00000187721 | 224 | 243 - | 1.13E-05 | GATGCAGGCCACTTTGGCCT |
| ESR1 | ENSG00000171169 | 373 | 392 - | 1.14E-05 | GAGCCGGGTGACCCCGCCCA |
| ESR1 | ENSG00000249293 | 355 | 374 - | 1.15E-05 | ACCTGAGGTGACACTGGCCC |
| ESR1 | ENSG00000226232 | 341 | 360 - | 1.17E-05 | GCTCAAGGGCACCAGGCCCC |
| ESR1 | ENSG00000248423 | 239 | 258 + | 1.17E-05 | ACCCCTGGGGATCCTGTCCT |
| ESR1 | ENSG00000196353 | 121 | 140 + | 1.19E-05 | TCCCAAGACCACCCTGGCCC |
| ESR1 | ENSG00000103023 | 254 | 273 + | 1.29E-05 | GTGTCAGGCCAGCCTGAGTT |
| ESR1 | ENSG00000099337 | 64 | 83 - | 1.30E-05 | AGCCCAGTGCTCCCAGACCC |

| | | | | | | |
|---|---|---|---|---|---|---|
| ESR1 | ENSG00000196923 | 18 | 37 | + | 1.31E-05 | GGCCCGGGTCGGCGAGGCCT |
| ESR1 | ENSG00000114503 | 371 | 390 | - | 1.33E-05 | CGGCCCGGCCTCCCTCACCC |
| ESR1 | ENSG00000065518 | 38 | 57 | - | 1.34E-05 | CTCGCAGTGCGCCCTGCCCC |
| ESR1 | ENSG00000176261 | 118 | 137 | - | 1.35E-05 | CTCTCACGTGACCCTGACCG |
| ESR1 | ENSG00000159214 | 329 | 348 | + | 1.38E-05 | CGCCGAGGTGAGTCTGAACT |
| ESR1 | ENSG00000177192 | 83 | 102 | - | 1.42E-05 | AGGGCGGGGCACCAGGCCCT |
| ESR1 | ENSG00000165409 | 293 | 312 | + | 1.44E-05 | TAGCCAGGGCTGCGTGCCCG |
| ESR1 | ENSG00000169964 | 87 | 106 | - | 1.45E-05 | GGGCCAGGTGGCAGAGACCC |
| ESR1 | ENSG00000207783 | 68 | 87 | - | 1.45E-05 | GGGCCAGGTGGCAGAGACCC |
| ESR1 | ENSG00000138434 | 326 | 345 | + | 1.46E-05 | TCCCCGGGGCCCCCTGCCCG |
| ESR1 | ENSG00000230165 | 175 | 194 | + | 1.47E-05 | CACCCTGGACTACCTGCCCC |
| ESR1 | ENSG00000145494 | 310 | 329 | - | 1.47E-05 | CTCCCCGGTCGGCGAGACCC |
| ESR1 | ENSG00000109452 | 130 | 149 | + | 1.51E-05 | GGCTCTGGGCACCTTTACCT |
| ESR1 | ENSG00000243284 | 88 | 107 | + | 1.54E-05 | CAGGTAGGCCACCCTGTCAC |
| ESR1 | ENSG00000169583 | 59 | 78 | + | 1.54E-05 | GGTGGAGGTGGTCCTGACCC |
| ESR1 | ENSG00000172819 | 339 | 358 | - | 1.55E-05 | AGGCCAGGGCTCACAGACAT |
| ESR1 | ENSG00000245562 | 11 | 30 | + | 1.55E-05 | GGCACATGCCACCATGCCCA |
| ESR1 | ENSG00000186815 | 240 | 259 | - | 1.59E-05 | AAGAAAGGTCAGTCAGACAC |
| ESR1 | ENSG00000108298 | 266 | 285 | - | 1.60E-05 | AGACCAGCTCGCCCTCACCT |
| ESR1 | ENSG00000256757 | 149 | 168 | + | 1.62E-05 | CCTCAGGGGCATTCTGTCCC |
| ESR1 | ENSG00000070366 | 363 | 382 | + | 1.64E-05 | GGGCCGGGCCTGCCCGCCCC |
| ESR1 | ENSG00000074582 | 246 | 265 | - | 1.66E-05 | CTCCTACCTCACCCTGCCCT |
| ESR1 | ENSG00000117419 | 184 | 203 | - | 1.70E-05 | ACCTCCGCGCACTCTGACCC |
| ESR1 | ENSG00000208038 | 354 | 373 | + | 1.70E-05 | GGATGAGGTGACCCTGGCCA |
| ESR1 | ENSG00000234751 | 307 | 326 | + | 1.75E-05 | TCCACAGGGCTCCCCGCCCC |
| ESR1 | ENSG00000134780 | 15 | 34 | + | 1.85E-05 | GACTCGGCTCTCCCTGGCCT |
| ESR1 | ENSG00000149292 | 245 | 264 | + | 1.85E-05 | CAGGCAGGTCACTGCGCCAT |
| ESR1 | ENSG00000142156 | 262 | 281 | + | 1.87E-05 | GCGGCGGCCCACTCTGCCCT |
| ESR1 | ENSG00000252974 | 4 | 23 | - | 1.90E-05 | CGGCCGGGTGAGTGCGCCCT |
| ESR1 | ENSG00000134333 | 107 | 126 | + | 1.93E-05 | ACTGAAGGTCGTCCTGACTC |
| ESR1 | ENSG00000236546 | 121 | 140 | + | 1.94E-05 | AGGACTGGGCAGCCTCACTT |
| ESR1 | ENSG00000215039 | 293 | 312 | + | 1.94E-05 | CTGCCAGGTCTCACTCTCCC |
| ESR1 | ENSG00000004766 | 196 | 215 | - | 1.97E-05 | GACCCAGGGTATCCTGTCTT |
| ESR1 | ENSG00000175892 | 41 | 60 | - | 2.00E-05 | AACCGAGGCCAGAGTGTCCC |
| ESR1 | ENSG00000003989 | 64 | 83 | - | 2.01E-05 | GGCCCCGGCCGCGCTGACCA |
| ESR1 | ENSG00000249199 | 271 | 290 | + | 2.05E-05 | TAACAGGGACAGCCAGACCC |
| ESR1 | ENSG00000088298 | 352 | 371 | + | 2.07E-05 | TGTCCCGGCGACCCAGACCT |
| ESR1 | ENSG00000138496 | 82 | 101 | - | 2.07E-05 | GACCCAGTTCCAGCTGACCT |
| ESR1 | ENSG00000155984 | 56 | 75 | - | 2.08E-05 | GACACCTGTCACCTTGGCCC |
| ESR1 | ENSG00000123104 | 43 | 62 | + | 2.10E-05 | CCCGAGGGTCGCCCCGCCCC |
| ESR1 | ENSG00000110719 | 146 | 165 | - | 2.12E-05 | GGCCAGGGACGCCCCGCCCC |
| ESR1 | ENSG00000149532 | 339 | 358 | - | 2.12E-05 | CTTCCGGCCCAACCTGCCCC |
| ESR1 | ENSG00000206567 | 381 | 400 | + | 2.13E-05 | GCCTCAGGTGAGGGTCACCC |
| ESR1 | ENSG00000121236 | 151 | 170 | + | 2.15E-05 | GGGGCTGGACAATTTGACCC |
| ESR1 | ENSG00000151006 | 153 | 172 | - | 2.15E-05 | GTGCGAGACCAGCCTGACCA |
| ESR1 | ENSG00000241738 | 114 | 133 | - | 2.15E-05 | GTGCGAGACCAGCCTGACCA |
| ESR1 | ENSG00000248127 | 129 | 148 | - | 2.15E-05 | AGGCCAGGCGAAGCAGACCC |
| ESR1 | ENSG00000116171 | 112 | 131 | - | 2.16E-05 | GGACTGAGTCAACCTGTCCC |
| ESR1 | ENSG00000121310 | 254 | 273 | + | 2.16E-05 | GGACTGAGTCAACCTGTCCC |
| ESR1 | ENSG00000103056 | 300 | 319 | - | 2.18E-05 | GCCAATGGTGACCCGGACCC |

| | | | | | | |
|---|---|---|---|---|---|---|
| ESR1 | ENSG00000115239 | 220 | 239 | - | 2.18E-05 | CGCCGAGGGCACCGCCCCCT |
| ESR1 | ENSG00000122085 | 367 | 386 | + | 2.19E-05 | CGGCCAGGTCGTGGAGCCCT |
| ESR1 | ENSG00000144802 | 10 | 29 | + | 2.21E-05 | AATCTAGGCCTCGCTGCCCC |
| ESR1 | ENSG00000205089 | 380 | 399 | + | 2.21E-05 | GGGGGAGGCCCCTCTGCCCC |
| ESR1 | ENSG00000204618 | 22 | 41 | + | 2.21E-05 | GAGCCTGGGCACCCGGCCCA |
| ESR1 | ENSG00000226746 | 312 | 331 | - | 2.24E-05 | AGGCCAGGACAGCGTGGGCC |
| ESR1 | ENSG00000131435 | 83 | 102 | - | 2.24E-05 | CCCCGCGGCCAGCCCGACCC |
| ESR1 | ENSG00000223430 | 58 | 77 | - | 2.25E-05 | TTCTTGGGTGGGCCTGACCT |
| ESR1 | ENSG00000112365 | 299 | 318 | - | 2.25E-05 | ACGCCGGGGCCCGCTGGCCC |
| ESR1 | ENSG00000222482 | 97 | 116 | - | 2.29E-05 | CTCCCTGGTGACTGTGAGCT |
| ESR1 | ENSG00000133808 | 163 | 182 | + | 2.32E-05 | GCGGAAGGGGACGCTGCCTC |
| ESR1 | ENSG00000138111 | 2 | 21 | + | 2.35E-05 | GAATCCAGGCAACCTGACCC |
| ESR1 | ENSG00000240224 | 67 | 86 | + | 2.35E-05 | GGAGGAGGGCACTCTGTCTT |
| ESR1 | ENSG00000156508 | 56 | 75 | + | 2.35E-05 | AGGCCAGCTCACGGTGACAA |
| ESR1 | ENSG00000229674 | 308 | 327 | - | 2.36E-05 | GGTGCCGGGCATGCTGACTT |
| ESR1 | ENSG00000173137 | 212 | 231 | - | 2.38E-05 | CTCCTGAGTCAGCTTGGCCT |
| ESR1 | ENSG00000255152 | 368 | 387 | + | 2.40E-05 | CCCACAGGGCCCTCAGACCC |
| ESR1 | ENSG00000006062 | 180 | 199 | - | 2.42E-05 | GCCCGTGGTCGCCCCGCCCC |
| ESR1 | ENSG00000146376 | 126 | 145 | - | 2.45E-05 | AGGGAAGGGCTTCCTGCCTC |
| ESR1 | ENSG00000228063 | 13 | 32 | + | 2.46E-05 | GGGGCAGGTGGCGCTGCCCG |
| ESR1 | ENSG00000253528 | 100 | 119 | - | 2.46E-05 | AACCCAGGGCCTTCTGGCCC |
| ESR1 | ENSG00000250101 | 196 | 215 | + | 2.47E-05 | CGCCCACGTCGGCCTCACCC |
| ESR1 | ENSG00000221817 | 92 | 111 | + | 2.48E-05 | GCTGAAGGTCTCCGAGCCCC |
| ESR1 | ENSG00000132330 | 180 | 199 | + | 2.48E-05 | GACCCAGCGCAGCCTCGCCT |
| ESR1 | ENSG00000234925 | 373 | 392 | - | 2.57E-05 | GGCCTTGGCCACGTTGGCCT |
| ESR1 | ENSG00000227802 | 205 | 224 | + | 2.57E-05 | GGCCCAGGTCTCCGAGCACT |
| ESR1 | ENSG00000214535 | 116 | 135 | + | 2.57E-05 | GTACAAGACCAGCCTGACCA |
| ESR1 | ENSG00000256566 | 206 | 225 | + | 2.59E-05 | GATCCCGGGCACGCTGGCTC |
| ESR1 | ENSG00000258966 | 158 | 177 | - | 2.63E-05 | GCTGCAGGGCACCGGGGCCT |
| ESR1 | ENSG00000078687 | 26 | 45 | - | 2.68E-05 | ACTGCAGGTTGCCCTGACTT |
| ESR1 | ENSG00000125733 | 51 | 70 | + | 2.68E-05 | CCCCCAGCTCACTCTCACCA |
| ESR1 | ENSG00000256278 | 133 | 152 | - | 2.69E-05 | ATCTCAGCTCACTGTAACCT |
| ESR1 | ENSG00000231942 | 122 | 141 | + | 2.71E-05 | ATCTGAGGTCAGCTTGCTCC |
| ESR1 | ENSG00000240848 | 218 | 237 | - | 2.73E-05 | GGCTCAGGTGATCCTCCCAC |
| ESR1 | ENSG00000240776 | 256 | 275 | - | 2.75E-05 | CTTCTTGGCCACCATGACTC |
| ESR1 | ENSG00000214593 | 241 | 260 | - | 2.75E-05 | CTTCTTGGCCACCATGACTC |
| ESR1 | ENSG00000137817 | 52 | 71 | - | 2.76E-05 | GATGGAGAGCACCCTGGCCT |
| ESR1 | ENSG00000198417 | 339 | 358 | + | 2.77E-05 | CGCTCAGGGGACCTTGCGCC |
| ESR1 | ENSG00000134571 | 47 | 66 | + | 2.83E-05 | AGCCAGGGACAATGTGGCCC |
| ESR1 | ENSG00000181826 | 248 | 267 | - | 2.83E-05 | CCCCCAGGGCGCCGCGTCCC |
| ESR1 | ENSG00000187796 | 40 | 59 | + | 2.83E-05 | TCCCCAGGACCCCCAGCCCC |
| ESR1 | ENSG00000185885 | 49 | 68 | + | 2.87E-05 | CGCCTAGGTCATCCAGCACT |
| ESR1 | ENSG00000147684 | 77 | 96 | + | 2.87E-05 | GGGACAAGTCAGAAAGACCC |
| ESR1 | ENSG00000222489 | 70 | 89 | + | 2.89E-05 | ACCACAGGCCACCATGCCTG |
| ESR1 | ENSG00000130997 | 29 | 48 | - | 2.90E-05 | CGCTGCGGGCATCCTGCCTC |
| ESR1 | ENSG00000232748 | 268 | 287 | + | 2.93E-05 | CTGGAGGGTCCCCCAGCCCC |
| ESR1 | ENSG00000109625 | 134 | 153 | + | 2.95E-05 | GGGGCGGGGCACTCCGCCCG |
| ESR1 | ENSG00000157077 | 311 | 330 | + | 2.99E-05 | CGGACGGGGCGTCCTGCCAC |
| ESR1 | ENSG00000219665 | 146 | 165 | - | 3.01E-05 | TGGATAGGGCATCCTACCCC |
| ESR1 | ENSG00000205231 | 111 | 130 | - | 3.04E-05 | CATCATGGACATTGTGACCC |

| | | | | | | |
|---|---|---|---|---|---|---|
| ESR1 | ENSG00000223878 | 159 | 178 | - | 3.04E-05 | GTTCTTGGTCTCACTGACTT |
| ESR1 | ENSG00000171056 | 83 | 102 | + | 3.04E-05 | TCCCCAGGTTGCCCCGCCCT |
| ESR1 | ENSG00000254093 | 120 | 139 | + | 3.04E-05 | TCCCCAGGTTGCCCCGCCCT |
| ESR1 | ENSG00000159337 | 50 | 69 | - | 3.05E-05 | CTGGCAGGGCTCCAGGACCT |
| ESR1 | ENSG00000121152 | 331 | 350 | - | 3.07E-05 | CGCCCAGGCCAGCCCGCCAG |
| ESR1 | ENSG00000174564 | 178 | 197 | - | 3.07E-05 | ATGCCTGGTGACTTTGCCAT |
| ESR1 | ENSG00000076554 | 247 | 266 | + | 3.07E-05 | GATCCTGGTCAGCTTCACAT |
| ESR1 | ENSG00000156150 | 266 | 285 | - | 3.08E-05 | GGCTCAGGGCGCACAGGCCT |
| ESR1 | ENSG00000108010 | 42 | 61 | - | 3.10E-05 | CCGCTGGGGGGCTCTGACCC |
| ESR1 | ENSG00000163738 | 126 | 145 | + | 3.11E-05 | GGGCCAGATCACCGTTGCCT |
| ESR1 | ENSG00000258959 | 17 | 36 | + | 3.13E-05 | TGGCCGGGGCAGCGTGGGCC |
| ESR1 | ENSG00000101082 | 269 | 288 | - | 3.13E-05 | GGCCCAGGGCCCTTTGAGCT |
| ESR1 | ENSG00000102312 | 265 | 284 | - | 3.13E-05 | TCCTCCGGGCAGCCTCCCCC |
| ESR1 | ENSG00000138495 | 345 | 364 | + | 3.14E-05 | ATGCCGGGTCTGGTTGACTC |
| ESR1 | ENSG00000167548 | 202 | 221 | - | 3.18E-05 | CGGAGTGGCCACCCTCCCCC |
| ESR1 | ENSG00000226055 | 325 | 344 | - | 3.21E-05 | CTTCCAGGTGATTCTGACTG |
| ESR1 | ENSG00000060656 | 279 | 298 | - | 3.22E-05 | GGCGCGGGGGACGCCGCCCC |
| ESR1 | ENSG00000232912 | 204 | 223 | + | 3.24E-05 | CGGAAAGATGGCCCTGCCCT |
| ESR1 | ENSG00000184381 | 69 | 88 | - | 3.25E-05 | GCCCAGGGTCAGCCAGCCTG |
| ESR1 | ENSG00000137965 | 366 | 385 | + | 3.30E-05 | CTCTCAGGTGACAGTGTCAC |
| ESR1 | ENSG00000114786 | 126 | 145 | - | 3.32E-05 | GCCCCAGCGCTCTCTGGCCC |
| ESR1 | ENSG00000248930 | 43 | 62 | - | 3.32E-05 | CACTGAGGCCAACCTGAACT |
| ESR1 | ENSG00000124693 | 326 | 345 | + | 3.32E-05 | CCGCCCGGGCACTGTGGCTC |
| ESR1 | ENSG00000150893 | 379 | 398 | - | 3.34E-05 | TGGACAGGTCAGCCTGAGAG |
| ESR1 | ENSG00000161179 | 238 | 257 | + | 3.34E-05 | GACCCAGGCGGCCATGTCCC |
| ESR1 | ENSG00000231980 | 15 | 34 | + | 3.39E-05 | GGCCCTGGTCATCCTTCCAC |
| ESR1 | ENSG00000140943 | 241 | 260 | - | 3.40E-05 | CGACCGGGCCAGCGAGGCCC |
| ESR1 | ENSG00000106608 | 264 | 283 | + | 3.40E-05 | TGCCCGGGTTACGCTGGCCA |
| ESR1 | ENSG00000252304 | 87 | 106 | + | 3.40E-05 | AGCGGAAGTCACTATGACTC |
| ESR1 | ENSG00000233246 | 323 | 342 | + | 3.44E-05 | GCACCGGGTGAACCTGCGCT |
| ESR1 | ENSG00000243135 | 85 | 104 | + | 3.45E-05 | AGAGGAGGGCACTCTGTCTT |
| ESR1 | ENSG00000236842 | 315 | 334 | + | 3.47E-05 | CCCCGACGGCGGCCTGACCC |
| ESR1 | ENSG00000114126 | 322 | 341 | + | 3.47E-05 | CGCGGGGCGCGCCCTGACCC |
| ESR1 | ENSG00000154309 | 5 | 24 | + | 3.49E-05 | AAGCCAAGCCCCTCTGCCCT |
| ESR1 | ENSG00000172345 | 91 | 110 | + | 3.49E-05 | CCCCGCGGTCTCCTTGTCCT |
| ESR1 | ENSG00000135476 | 47 | 66 | + | 3.51E-05 | CGCGCAGAGCAGCAAGACCC |
| ESR1 | ENSG00000240053 | 173 | 192 | + | 3.56E-05 | GGCCCAGCCCACCCTCCCCA |
| ESR1 | ENSG00000170421 | 140 | 159 | + | 3.58E-05 | GCACCTGGTCACTCTGGGCC |
| ESR1 | ENSG00000222020 | 55 | 74 | - | 3.65E-05 | CTCATAGGGCATCCGGCCCC |
| ESR1 | ENSG00000147642 | 345 | 364 | + | 3.68E-05 | CACCCAGCGGTGCCTGCCCT |
| ESR1 | ENSG00000007202 | 253 | 272 | + | 3.74E-05 | CCCGCGGGTCCCCGAGCCCC |
| ESR1 | ENSG00000145819 | 131 | 150 | - | 3.76E-05 | CAATCAGCGCACCCTCCCCT |
| ESR1 | ENSG00000198742 | 80 | 99 | + | 3.77E-05 | GTAGCAGCGCACCCCGCCCC |
| ESR1 | ENSG00000230798 | 41 | 60 | - | 3.79E-05 | GGTCCCGGTCCCGCTGCCCA |
| ESR1 | ENSG00000232536 | 316 | 335 | + | 3.79E-05 | GTTCGGGGCGTCCCTGCCCT |
| ESR1 | ENSG00000177868 | 184 | 203 | - | 3.85E-05 | GACCCAGCGCTGCCTGCCCA |
| ESR1 | ENSG00000154134 | 346 | 365 | + | 3.87E-05 | GGGGCTGGGCCCCCAGCCCC |
| ESR1 | ENSG00000226054 | 46 | 65 | - | 3.89E-05 | GGCCCACGGCGGCCTCACCT |
| ESR1 | ENSG00000255282 | 13 | 32 | - | 3.91E-05 | GAGAATGGGCAGACTGCCTC |
| ESR1 | ENSG00000006740 | 7 | 26 | + | 4.00E-05 | GCTGCTGGTGACGCTGCCTT |

| | | | | | | |
|---|---|---|---|---|---|---|
| ESR1 | ENSG00000214300 | 139 | 158 | + | 4.04E-05 | TTGCCAGGTTGGAATGACCT |
| ESR1 | ENSG00000215450 | 203 | 222 | - | 4.08E-05 | TCCCCAGGGCCTCCAGCCCC |
| ESR1 | ENSG00000227848 | 341 | 360 | - | 4.12E-05 | GGCGCTGGTCACCTTTACAT |
| ESR1 | ENSG00000141380 | 77 | 96 | - | 4.12E-05 | TTGGAAAGTAACCCTGACCC |
| ESR1 | ENSG00000183773 | 310 | 329 | + | 4.14E-05 | CGCCCCGGGGACCCTGGTCC |
| ESR1 | ENSG00000226312 | 273 | 292 | - | 4.16E-05 | ACTCAAAGTCACACTGTCAT |
| ESR1 | ENSG00000104976 | 166 | 185 | + | 4.18E-05 | CGCGCGGTTCTTTCTGACCT |
| ESR1 | ENSG00000205424 | 188 | 207 | - | 4.22E-05 | TGGTCATGCCTTCCTGCCCT |
| ESR1 | ENSG00000100605 | 256 | 275 | - | 4.26E-05 | GAGGAAGCGGACCCTGGCCC |
| ESR1 | ENSG00000216642 | 230 | 249 | + | 4.26E-05 | AAAAAGGGCCACTCTGCCAT |
| ESR1 | ENSG00000219481 | 148 | 167 | + | 4.35E-05 | GCCTTGGGTTATCCTGACAC |
| ESR1 | ENSG00000236132 | 359 | 378 | + | 4.37E-05 | GTTCGAGACCAGCCTGACCA |
| ESR1 | ENSG00000251314 | 319 | 338 | - | 4.37E-05 | TCCCCAAGCCAGCCTGGCCA |
| ESR1 | ENSG00000258802 | 217 | 236 | + | 4.45E-05 | TTCCAAGGGCGCCGGGCCCT |
| ESR1 | ENSG00000214184 | 326 | 345 | + | 4.45E-05 | GGCCCGGCTCTCCTTGCCTC |
| ESR1 | ENSG00000232830 | 306 | 325 | + | 4.48E-05 | CTGTGAGGACAGCCAGGCCT |
| ESR1 | ENSG00000251705 | 176 | 195 | + | 4.52E-05 | CGCCCTTGCCAAATTGACCT |
| ESR1 | ENSG00000223501 | 253 | 272 | - | 4.54E-05 | CAGCCAGGTCGCGCTGAAAC |
| ESR1 | ENSG00000231500 | 167 | 186 | + | 4.54E-05 | CAGCCAGGTCGCGCTGAAAC |
| ESR1 | ENSG00000099901 | 4 | 23 | + | 4.56E-05 | CAGCCAGGGCAGCAAGGCAC |
| ESR1 | ENSG00000072274 | 53 | 72 | - | 4.56E-05 | CGCGATGGGCGCACTGGCCT |
| ESR1 | ENSG00000170873 | 25 | 44 | + | 4.56E-05 | GGCCCAGCGCCGCCTGCCCG |
| ESR1 | ENSG00000253773 | 63 | 82 | + | 4.58E-05 | TATCCAGGCCTGCATGACTC |
| ESR1 | ENSG00000215127 | 38 | 57 | - | 4.61E-05 | GTCTCGGCTCACTGTAACCT |
| ESR1 | ENSG00000152942 | 307 | 326 | + | 4.61E-05 | TGCCCAGAGCACTCTGCGCC |
| ESR1 | ENSG00000184508 | 106 | 125 | - | 4.65E-05 | GGCCGAGTTGGGCCTGCCCC |
| ESR1 | ENSG00000228703 | 132 | 151 | - | 4.67E-05 | CCTCATGGTCTCCCTGTCCA |
| ESR1 | ENSG00000250644 | 366 | 385 | - | 4.67E-05 | GGCTGGGATCACCGAGCCCT |
| ESR1 | ENSG00000249249 | 61 | 80 | + | 4.67E-05 | CGGAAAGGCCTGCCCGCCCC |
| ESR1 | ENSG00000129472 | 265 | 284 | - | 4.70E-05 | GTTCCGGGTCCGCCCGACTT |
| ESR1 | ENSG00000224078 | 18 | 37 | + | 4.70E-05 | TTTCCAGGTCCACCTCCCCC |
| ESR1 | ENSG00000135926 | 229 | 248 | - | 4.72E-05 | CGGCTGGGGCTCCGCGCCCC |
| ESR1 | ENSG00000173210 | 336 | 355 | - | 4.79E-05 | GAGCCAGAGGCCCCTGCCCC |
| ESR1 | ENSG00000075415 | 349 | 368 | - | 4.81E-05 | AGGCCCGGCCACACTCACTC |
| ESR1 | ENSG00000202415 | 203 | 222 | - | 4.83E-05 | CAGCCAGGTTGCCCTCACAT |
| ESR1 | ENSG00000256390 | 6 | 25 | - | 4.86E-05 | TGTCCTGGTCACCAGGGCCC |
| ESR1 | ENSG00000151465 | 193 | 212 | - | 4.93E-05 | CCCTAAACGCACGCTGACCC |
| ESR1 | ENSG00000157985 | 352 | 371 | + | 4.93E-05 | CAGCGCGGCCACACTGCCCA |
| ESR1 | ENSG00000235374 | 82 | 101 | + | 4.93E-05 | GGCTGAGGAGACTCTGCCCG |
| ESR1 | ENSG00000251186 | 225 | 244 | + | 4.93E-05 | TCAGCATGTCTGCTTGACCT |
| ESR1 | ENSG00000196712 | 343 | 362 | + | 5.02E-05 | TCCCCGGGTCCCCTTCCCCT |
| ESR1 | ENSG00000067182 | 107 | 126 | - | 5.05E-05 | GTCCCAGATCTGGCTGCCTT |
| ESR1 | ENSG00000197647 | 323 | 342 | + | 5.07E-05 | GGTTCAGGTGCCTCTGCCAT |
| ESR1 | ENSG00000143878 | 44 | 63 | + | 5.07E-05 | AATCAAGCTGGCCCTGCCCC |
| ESR1 | ENSG00000244926 | 106 | 125 | - | 5.10E-05 | ACCACGTGTCTTCTTGACCT |
| ESR1 | ENSG00000213204 | 291 | 310 | - | 5.10E-05 | GGTCAAGGGCGCGGAGCCCC |
| ESR1 | ENSG00000160957 | 127 | 146 | - | 5.10E-05 | GCCCCTGGGCAGCCAGTCCG |
| ESR1 | ENSG00000164051 | 268 | 287 | + | 5.12E-05 | GAGCACGTTCGGCCTGCCCC |
| ESR1 | ENSG00000129158 | 113 | 132 | - | 5.20E-05 | CCTCCCAGTCAGCCCGCCCC |
| ESR1 | ENSG00000258509 | 329 | 348 | + | 5.22E-05 | TGAACAGATCATCGTGACCG |

| | | | | | | |
|---|---|---|---|---|---|---|
| ESR1 | ENSG00000154274 | 247 | 266 | + | 5.22E-05 | AGCCATGGGCGCCGGGACCC |
| ESR1 | ENSG00000163870 | 294 | 313 | - | 5.25E-05 | GACCGAGGTCACGCAGCCAG |
| ESR1 | ENSG00000168505 | 103 | 122 | + | 5.30E-05 | GGCCAGAGGCGCCCAGCCCC |
| ESR1 | ENSG00000249967 | 55 | 74 | + | 5.32E-05 | GGGGATGGACAGCCTGTCAC |
| ESR1 | ENSG00000102024 | 174 | 193 | - | 5.35E-05 | AAGCCAGCGCTCCCTCCCCT |
| ESR1 | ENSG00000110092 | 380 | 399 | + | 5.37E-05 | CAGCCAGGACCCACAGCCCT |
| ESR1 | ENSG00000243725 | 175 | 194 | - | 5.40E-05 | AGCGCAGGTCCCCGCGCCTT |
| ESR1 | ENSG00000235546 | 287 | 306 | - | 5.40E-05 | GCCCCAGCTCATGCTGTCTC |
| ESR1 | ENSG00000234062 | 189 | 208 | + | 5.48E-05 | GCGACAGCTCACGATGCCCG |
| ESR1 | ENSG00000137101 | 272 | 291 | - | 5.50E-05 | CAGTCATGTCACCCAGTCCA |
| ESR1 | ENSG00000023171 | 273 | 292 | + | 5.53E-05 | TCCCAGGGTCTCCTTGGCTT |
| ESR1 | ENSG00000103832 | 375 | 394 | - | 5.55E-05 | GGGGCAGGGCTGACTGACAA |
| ESR1 | ENSG00000232485 | 81 | 100 | + | 5.61E-05 | TCACCATGTCGCCGCGACCT |
| ESR1 | ENSG00000230753 | 25 | 44 | + | 5.61E-05 | GCGTCAGGGCAACGCGCCAC |
| ESR1 | ENSG00000228172 | 211 | 230 | - | 5.66E-05 | CCTCCCGGGGACACAGACCC |
| ESR1 | ENSG00000135916 | 181 | 200 | - | 5.69E-05 | GTCCAAGGCCAGCATCCCCG |
| ESR1 | ENSG00000203301 | 294 | 313 | + | 5.71E-05 | CCCCGTGCTCCCCCTGCCCT |
| ESR1 | ENSG00000246889 | 84 | 103 | - | 5.74E-05 | GACCCGGGCCGGACCGACCC |
| ESR1 | ENSG00000065361 | 338 | 357 | - | 5.80E-05 | AGCCTCGGACCGCCTGCCCC |
| ESR1 | ENSG00000213641 | 379 | 398 | - | 5.82E-05 | AGAGAATGGCTCTCTGCCCT |
| ESR1 | ENSG00000129194 | 262 | 281 | - | 5.82E-05 | GTGGCAGGCGGGCCAGACCT |
| ESR1 | ENSG00000168887 | 151 | 170 | + | 5.82E-05 | GGCCGGGGCCTCCCAGACCG |
| ESR1 | ENSG00000254827 | 114 | 133 | - | 5.85E-05 | CCCTCTGGAGTCCCTGCCCC |
| ESR1 | ENSG00000184669 | 112 | 131 | - | 5.91E-05 | CCCGCCGGGCGCCCCGCCCT |
| ESR1 | ENSG00000168291 | 69 | 88 | - | 5.91E-05 | CGTGTGGGTGGCCGTGCCCC |
| ESR1 | ENSG00000147687 | 181 | 200 | + | 5.91E-05 | CTACAAGGTCTCCCGGCCAT |
| ESR1 | ENSG00000147874 | 68 | 87 | - | 5.91E-05 | CGCTATGCTCACTGTGACTC |
| ESR1 | ENSG00000218739 | 351 | 370 | + | 5.99E-05 | TAGTCAGGTGTCACTGCCTT |
| ESR1 | ENSG00000136143 | 375 | 394 | + | 6.02E-05 | GCAGCTGGTCACCACGACTC |
| ESR1 | ENSG00000228501 | 304 | 323 | + | 6.05E-05 | TCCACAGGGCTCTCCGCCCC |
| ESR1 | ENSG00000157851 | 112 | 131 | - | 6.05E-05 | GGCTGCGGCCGCGCTGCCCT |
| ESR1 | ENSG00000145907 | 174 | 193 | + | 6.05E-05 | CGCGCGGACCACCGTGCCTC |
| ESR1 | ENSG00000176029 | 121 | 140 | + | 6.11E-05 | CTCCCAAGGCCTCCTGTCCC |
| ESR1 | ENSG00000083845 | 332 | 351 | + | 6.11E-05 | GAGACGGGTCGCGCTGCTCC |
| ESR1 | ENSG00000225611 | 164 | 183 | - | 6.14E-05 | TGCCTGGGGCAGCCTGCTTT |
| ESR1 | ENSG00000241550 | 134 | 153 | - | 6.16E-05 | CGCCCAGGTCATCTTGTATT |
| ESR1 | ENSG00000157106 | 86 | 105 | - | 6.19E-05 | ATAGCAGGCCGCCCCGCCCC |
| ESR1 | ENSG00000139154 | 202 | 221 | + | 6.25E-05 | AAGCAAGGGCAACCAGCTCC |
| ESR1 | ENSG00000126226 | 181 | 200 | + | 6.25E-05 | GGCCGGGGGCGCCAGGACCC |
| ESR1 | ENSG00000168883 | 318 | 337 | + | 6.28E-05 | ATCTCGGGGCAGCCTGCATT |
| ESR1 | ENSG00000169184 | 238 | 257 | + | 6.28E-05 | ACCCCTCGTCTCGCTGCCCT |
| ESR1 | ENSG00000250251 | 177 | 196 | + | 6.34E-05 | GGCCATGCGCGCGCTGCCCT |
| ESR1 | ENSG00000125966 | 250 | 269 | - | 6.34E-05 | GCGCCCGGCCCGCCTGCCTT |
| ESR1 | ENSG00000113231 | 266 | 285 | + | 6.43E-05 | GCGGGCGGGCGCCCTGGCCC |
| ESR1 | ENSG00000118849 | 222 | 241 | - | 6.46E-05 | GGGCCGGGTGCTTCTGGCCC |
| ESR1 | ENSG00000113282 | 304 | 323 | - | 6.55E-05 | GGCCGGGGTCACCGCCGCCC |
| ESR1 | ENSG00000130589 | 236 | 255 | - | 6.62E-05 | CTCCTGGAGCTGCCTGCCCT |
| ESR1 | ENSG00000008710 | 236 | 255 | + | 6.71E-05 | CTCCCGGAGCGGCCTGGCCC |
| ESR1 | ENSG00000176715 | 57 | 76 | + | 6.71E-05 | CTCCCCGGTCAGCTTGCCGC |
| ESR1 | ENSG00000255185 | 75 | 94 | + | 6.74E-05 | TCCACCGGGCATCCTCCCCC |

| | | | | | | |
|---|---|---|---|---|---|---|
| ESR1 | ENSG00000071794 | 327 | 346 | - | 6.74E-05 | CTTCCAGGCCCCGCAGCCCT |
| ESR1 | ENSG00000113716 | 378 | 397 | - | 6.77E-05 | CGCCCCGGCCGCTCAGCCCC |
| ESR1 | ENSG00000255158 | 115 | 134 | + | 6.80E-05 | GGCTGCAGTCACCCTGGCCA |
| ESR1 | ENSG00000173281 | 211 | 230 | - | 6.80E-05 | CCCCGCGGCCGCGCTGCCCC |
| ESR1 | ENSG00000143499 | 365 | 384 | - | 6.90E-05 | GCTCCAGGCCGCCGAGGCCC |
| ESR1 | ENSG00000131773 | 195 | 214 | - | 6.90E-05 | CGCCCCGCGCACTCTGAGCC |
| ESR1 | ENSG00000236658 | 1 | 20 | - | 6.90E-05 | GACAGAGGTCACCCTGTAAC |
| ESR1 | ENSG00000183020 | 220 | 239 | + | 6.93E-05 | GGAGCTGTGCAGCCTGGCCC |
| ESR1 | ENSG00000138100 | 102 | 121 | - | 6.93E-05 | GGAGTGAGTGACCCTGACTC |
| ESR1 | ENSG00000163083 | 269 | 288 | + | 7.03E-05 | CGCTGGGGGCCGCCTGCCTT |
| ESR1 | ENSG00000127928 | 114 | 133 | - | 7.03E-05 | AAATCGGGTCACTCCCACCC |
| ESR1 | ENSG00000139637 | 88 | 107 | + | 7.06E-05 | ATCGCAGGGCCCTTTGACCA |
| ESR1 | ENSG00000100320 | 137 | 156 | + | 7.06E-05 | GGCCCAGGGGGAGGTGGCCT |
| ESR1 | ENSG00000100234 | 148 | 167 | - | 7.13E-05 | GCCCCCGGGGACCCCGCCTC |
| ESR1 | ENSG00000130560 | 266 | 285 | - | 7.13E-05 | CGGCCGGGCCGCCGTCACTC |
| ESR1 | ENSG00000006607 | 207 | 226 | - | 7.16E-05 | CGCGCAGCGCGCCCCGCCCT |
| ESR1 | ENSG00000255432 | 224 | 243 | + | 7.20E-05 | GGGAAAGATCAACCGGCCCC |
| ESR1 | ENSG00000189343 | 241 | 260 | + | 7.20E-05 | GGCCCTGGTGGCCCTGAGAT |
| ESR1 | ENSG00000176148 | 164 | 183 | + | 7.23E-05 | GCCACTGGCCACGGAGCCCC |
| ESR1 | ENSG00000148296 | 209 | 228 | - | 7.23E-05 | GGGCCTGGCCTCAGTGCCTC |
| ESR1 | ENSG00000233202 | 231 | 250 | - | 7.23E-05 | CCTCGAGGGCAGCCTCAGCT |
| ESR1 | ENSG00000089101 | 320 | 339 | - | 7.26E-05 | CGCCCTGGACGCCCTGCACC |
| ESR1 | ENSG00000233603 | 86 | 105 | + | 7.26E-05 | TGGCTTTCCCACCCTGACCT |
| ESR1 | ENSG00000227589 | 241 | 260 | + | 7.30E-05 | AGCTCTCATCACTCTGACCT |
| ESR1 | ENSG00000258930 | 368 | 387 | + | 7.30E-05 | ATGCATGGGCTCCCTGCACT |
| ESR1 | ENSG00000109854 | 156 | 175 | - | 7.37E-05 | AGGCGCGGGGAAGGTGACCC |
| ESR1 | ENSG00000124201 | 175 | 194 | + | 7.37E-05 | ACGCCGGGCCACCCGGGCTT |
| ESR1 | ENSG00000008988 | 301 | 320 | - | 7.43E-05 | GCGCGCGGGCTTCCTGACCG |
| ESR1 | ENSG00000176386 | 177 | 196 | - | 7.47E-05 | CGCTCCGGGGGCGGTGACCC |
| ESR1 | ENSG00000224386 | 201 | 220 | + | 7.54E-05 | GCCTGGGGCCGGCCTGGCCC |
| ESR1 | ENSG00000164048 | 56 | 75 | - | 7.61E-05 | GCTGAGGCGCACTTTGACCT |
| ESR1 | ENSG00000227328 | 173 | 192 | + | 7.61E-05 | GGCCCAAGGGGCACTGTCCT |
| ESR1 | ENSG00000163126 | 246 | 265 | - | 7.65E-05 | GCCGCGCGGCAGCCTCACCC |
| ESR1 | ENSG00000237669 | 20 | 39 | + | 7.65E-05 | AGCCCCGCCCACCGAGCCCT |
| ESR1 | ENSG00000258660 | 382 | 401 | + | 7.68E-05 | CACGTTGGCCAGGCTGGCCT |
| ESR1 | ENSG00000189269 | 180 | 199 | + | 7.68E-05 | CAGCCAGGGCCAGCAGCCCT |
| ESR1 | ENSG00000093167 | 86 | 105 | + | 7.72E-05 | GTTATCAGCCACCCTGCCCC |
| ESR1 | ENSG00000243444 | 326 | 345 | - | 7.72E-05 | CGCCGCGGTCACCATGGCAA |
| ESR1 | ENSG00000147036 | 16 | 35 | + | 7.72E-05 | GGGACTGGAGACCATCACCT |
| ESR1 | ENSG00000134824 | 19 | 38 | - | 7.79E-05 | CTCTCATGGCAACCAGTCCC |
| ESR1 | ENSG00000134825 | 186 | 205 | + | 7.79E-05 | CTCTCATGGCAACCAGTCCC |
| ESR1 | ENSG00000168496 | 362 | 381 | - | 7.79E-05 | CTCTCATGGCAACCAGTCCC |
| ESR1 | ENSG00000215208 | 337 | 356 | + | 7.79E-05 | TTCCCGGATCTCCATGTCCT |
| ESR1 | ENSG00000182909 | 90 | 109 | - | 7.79E-05 | AGCTCTTGGGAGCCTGGCCC |
| ESR1 | ENSG00000249915 | 142 | 161 | + | 7.79E-05 | TGCGCGGGACCCCGTGACCA |
| ESR1 | ENSG00000151117 | 174 | 193 | + | 7.82E-05 | AACCCATGTTTGCCTGGCCC |
| ESR1 | ENSG00000111252 | 92 | 111 | + | 7.82E-05 | GCTCCTGGTGGCCCCGCCCC |
| ESR1 | ENSG00000171345 | 24 | 43 | - | 7.82E-05 | AGACCAGAGGCCCCTGCCCT |
| ESR1 | ENSG00000257298 | 308 | 327 | - | 7.86E-05 | CTGGCTGGGCACAGTGGCTC |
| ESR1 | ENSG00000144504 | 87 | 106 | - | 7.86E-05 | GACACGGGACAGCATGTCAC |

| | | | | | | |
|---|---|---|---|---|---|---|
| ESR1 | ENSG00000165819 | 336 | 355 | - | 7.90E-05 | CATCCTAGTCTCCCAGCCCT |
| ESR1 | ENSG00000231357 | 159 | 178 | - | 7.90E-05 | AGACAATGGGACTTTGCCCC |
| ESR1 | ENSG00000257069 | 304 | 323 | - | 7.93E-05 | CCCTGGGGCGTCCCTGCCCC |
| ESR1 | ENSG00000185666 | 375 | 394 | - | 7.93E-05 | GCTCCAGGGGACGCAGCCCG |
| ESR1 | ENSG00000152348 | 293 | 312 | - | 7.97E-05 | GAGCCGAGCCAGCGCGCCCC |
| ESR1 | ENSG00000111907 | 336 | 355 | + | 7.97E-05 | TGATCAGCCCACCTTGGCCT |
| ESR1 | ENSG00000232479 | 205 | 224 | - | 8.08E-05 | CTTCTGGGTCACCCTGATAG |
| ESR1 | ENSG00000170027 | 63 | 82 | - | 8.08E-05 | TCTTCCGGGCACCCCGTCCT |
| ESR1 | ENSG00000110171 | 345 | 364 | - | 8.12E-05 | CTGTCTGGGCCGCCTGCCTC |
| ESR1 | ENSG00000148344 | 20 | 39 | - | 8.12E-05 | TCTCCGGGTGTCCCGGACCC |
| ESR1 | ENSG00000175147 | 61 | 80 | - | 8.16E-05 | CAAGAAGGGGACCCCGGCCT |
| ESR1 | ENSG00000100568 | 183 | 202 | + | 8.27E-05 | CCCGCAGGTCCCCTAGGCCC |
| ESR1 | ENSG00000145014 | 238 | 257 | + | 8.27E-05 | AGGAAGGGACGCCGTGCCTT |
| ESR1 | ENSG00000109689 | 348 | 367 | - | 8.27E-05 | CCCAGAGGCAAGCCTGCCCT |
| ESR1 | ENSG00000125148 | 315 | 334 | + | 8.31E-05 | CACCCGGTACACTGTGTCCT |
| ESR1 | ENSG00000061938 | 19 | 38 | + | 8.42E-05 | TCCCTCTGGCAGCCTGGCCC |
| ESR1 | ENSG00000251467 | 124 | 143 | - | 8.46E-05 | GGCACAGCTCCGCCAGCCCC |
| ESR1 | ENSG00000212978 | 28 | 47 | + | 8.50E-05 | GGACGACGTCAACATGTCCT |
| ESR1 | ENSG00000226800 | 172 | 191 | + | 8.54E-05 | CCCGGGGGTCGCCCAGACTC |
| ESR1 | ENSG00000250174 | 246 | 265 | + | 8.58E-05 | ATTGAAGGTCAGCTTGTTCT |
| ESR1 | ENSG00000064763 | 180 | 199 | + | 8.62E-05 | GCGCGCGGCCGCCCTGCCCA |
| ESR1 | ENSG00000152503 | 125 | 144 | + | 8.62E-05 | CGGCGCTGGCAGCCAGCCCC |
| ESR1 | ENSG00000084676 | 303 | 322 | + | 8.66E-05 | CCCCGACGACGCCGTGACCT |
| ESR1 | ENSG00000066651 | 135 | 154 | - | 8.66E-05 | TGGGAGGGGCTTCCTCACCT |
| ESR1 | ENSG00000176444 | 316 | 335 | - | 8.70E-05 | GGGGCGGGGGGTCCAGACCT |
| ESR1 | ENSG00000151491 | 314 | 333 | + | 8.70E-05 | CGGGATGGGGTCCCTGTCCC |
| ESR1 | ENSG00000129515 | 145 | 164 | - | 8.74E-05 | GCCTAAGGACGGCCCGCCCC |
| ESR1 | ENSG00000249319 | 227 | 246 | + | 8.74E-05 | AGTCCTGGGGAGCCAGTCCC |
| ESR1 | ENSG00000143776 | 161 | 180 | - | 8.90E-05 | CGGCCTGGCCCCCCGGCCCC |
| ESR1 | ENSG00000090905 | 25 | 44 | + | 8.90E-05 | GGCTGGGTGCAGCGTGACAC |
| ESR1 | ENSG00000180385 | 307 | 326 | + | 8.90E-05 | AGGTCAGGGGATCGAGACCA |
| ESR1 | ENSG00000078319 | 290 | 309 | - | 9.03E-05 | TGACAATGTCACCTTCACTC |
| ESR1 | ENSG00000109079 | 1 | 20 | - | 9.11E-05 | GTACCGGCTGTCGCTGACCC |
| ESR1 | ENSG00000186868 | 260 | 279 | + | 9.11E-05 | CGCGGAGGCCGCGCTGCCCG |
| ESR1 | ENSG00000161642 | 61 | 80 | - | 9.15E-05 | GCGAGGGGGCGTCCTGGCCC |
| ESR1 | ENSG00000139988 | 173 | 192 | + | 9.15E-05 | TGATCAGCTCATCGTAACCT |
| ESR1 | ENSG00000175110 | 345 | 364 | - | 9.15E-05 | CCCAGAGGTCCTCCTGAGCT |
| ESR1 | ENSG00000257077 | 156 | 175 | + | 9.15E-05 | GGACATGGACTGCCTCACCT |
| ESR1 | ENSG00000080815 | 255 | 274 | - | 9.19E-05 | ACCCCGGAGCTGCCTGTCCC |
| ESR1 | ENSG00000258011 | 215 | 234 | + | 9.23E-05 | CTCGAAGTCCAGCCAGCCCT |
| ESR1 | ENSG00000234271 | 179 | 198 | - | 9.23E-05 | TTCTCTTCTCACCCTGTCCT |
| ESR1 | ENSG00000253172 | 201 | 220 | + | 9.28E-05 | GGGGCGGGGCTAGCTGCCTC |
| ESR1 | ENSG00000251447 | 302 | 321 | - | 9.32E-05 | GAGCCACCGCACCCAGCCCT |
| ESR1 | ENSG00000105519 | 196 | 215 | + | 9.36E-05 | TGGCTCTGCCACCCTGCCAC |
| ESR1 | ENSG00000244078 | 183 | 202 | + | 9.36E-05 | CTTATCTGGCACCCTGGCCT |
| ESR1 | ENSG00000151466 | 35 | 54 | + | 9.36E-05 | CTACCTGGAGACACTGTCCC |
| ESR1 | ENSG00000224680 | 39 | 58 | - | 9.40E-05 | AGGCAGGGCCTCGCTGGCTT |
| ESR1 | ENSG00000174791 | 5 | 24 | - | 9.40E-05 | AGGCTCTGCCAGCCTGCCTT |
| ESR1 | ENSG00000246982 | 382 | 401 | - | 9.40E-05 | GGGCGGGGGCATCGGGGCCT |
| ESR1 | ENSG00000115661 | 262 | 281 | + | 9.45E-05 | GAGCCAGGTCAGTCCGGCAG |

| | | | | | | |
|---|---|---|---|---|---|---|
| ESR1 | ENSG00000163521 | 144 | 163 | - | 9.45E-05 | GAGCCAGGTCAGTCCGGCAG |
| ESR1 | ENSG00000023839 | 78 | 97 | + | 9.53E-05 | CTGGTGAGTCTCCCTGTCCC |
| ESR1 | ENSG00000254460 | 105 | 124 | + | 9.53E-05 | TGCCTGGGGCTCTCTGAGCC |
| ESR1 | ENSG00000258466 | 75 | 94 | + | 9.53E-05 | AGGCCTGCTCACCACGACTC |
| ESR1 | ENSG00000255329 | 317 | 336 | - | 9.58E-05 | CGTCCAGGACTGATTGTCCT |
| ESR1 | ENSG00000214167 | 96 | 115 | - | 9.58E-05 | GATCAAGGCCAGGCAGCCTC |
| ESR1 | ENSG00000145868 | 100 | 119 | - | 9.58E-05 | AGCCATGTTCAGCGTGGCAT |
| ESR1 | ENSG00000230870 | 353 | 372 | - | 9.62E-05 | GTCCATGGTGATAATGACAC |
| ESR1 | ENSG00000256813 | 41 | 60 | - | 9.67E-05 | GCCTTCAGTGACCCTGGCCT |
| ESR1 | ENSG00000100811 | 12 | 31 | + | 9.67E-05 | CGCCGAGCTCGCGCCGACCC |
| ESR1 | ENSG00000075292 | 4 | 23 | + | 9.67E-05 | ACCTTAGGCCTCACAGACCT |
| ESR1 | ENSG00000143158 | 159 | 178 | + | 9.71E-05 | CAGACCCGGCAGCGTGCCCC |
| ESR1 | ENSG00000215845 | 181 | 200 | - | 9.71E-05 | AAGCAAGGGCACCGCCTCCC |
| ESR1 | ENSG00000110536 | 201 | 220 | + | 9.71E-05 | CTCCGAGGCCGCCGGGCCCT |
| ESR1 | ENSG00000214941 | 214 | 233 | + | 9.71E-05 | ACTCCGTCTCGCCCTGCCCC |
| ESR1 | ENSG00000149089 | 279 | 298 | + | 9.75E-05 | CGCCCCGTTCGCCCCGCCCC |
| ESR1 | ENSG00000172113 | 239 | 258 | + | 9.75E-05 | CGCCGAGGGCGCCCAGGCCG |
| ESR1 | ENSG00000145425 | 35 | 54 | - | 9.75E-05 | GACCAAGGCGTCGGTGGCCT |
| ESR1 | ENSG00000109685 | 369 | 388 | - | 9.80E-05 | CAGCCCGGCCTCCCGGCCCC |
| ESR1 | ENSG00000198042 | 200 | 219 | + | 9.80E-05 | AGGAGCGGGGACCCAGCCCC |
| ESR1 | ENSG00000107201 | 346 | 365 | + | 9.80E-05 | AGCAGAGGCCGGCATGACCA |
| ESR1 | ENSG00000157654 | 54 | 73 | + | 9.80E-05 | GCGCCGGGCTAGCGCGCCCT |
| ESR1 | ENSG00000116783 | 139 | 158 | - | 9.84E-05 | CGCCCGGTTGTTTCTGACCC |
| ESR1 | ENSG00000130813 | 26 | 45 | + | 9.84E-05 | GGCCCAGGTCCTCATGAGCG |
| ESR1 | ENSG00000197128 | 370 | 389 | - | 9.84E-05 | CGGCTAGGTCACTCAGGCAG |
| ESR1 | ENSG00000175029 | 60 | 79 | + | 9.89E-05 | GGCCCCTGCCACCGAGGCCC |
| ESR1 | ENSG00000166548 | 322 | 341 | - | 9.89E-05 | GGCGCTGGCCTGCGTGCCCA |
| ESR1 | ENSG00000254788 | 18 | 37 | + | 9.89E-05 | GGCGCTGGCCTGCGTGCCCA |
| ESR1 | ENSG00000236869 | 271 | 290 | - | 9.89E-05 | GCTACGGGGCGCCCGGCCCC |
| ESR1 | ENSG00000257809 | 26 | 45 | + | 9.98E-05 | TGCTCAGCTCTCCGGGACCT |
| ESR1 | ENSG00000135414 | 208 | 227 | + | 0.0001 | CCCCCAGTCCTCCCTCCCCT |
| ESR1 | ENSG00000241404 | 156 | 175 | + | 0.0001 | ATGGGAGGACTCTCTGGCCC |
| ESR1 | ENSG00000122432 | 267 | 286 | - | 0.0001 | GAGTCGGGTCGCGCTAGCCT |
| ESR1 | ENSG00000174516 | 184 | 203 | - | 0.0001 | TTCGCAGGAGACCACGCCCT |
| ESR1 | ENSG00000171853 | 149 | 168 | + | 0.0001 | CACCTAGGGCTCCAGGCCCC |
| ESR1 | ENSG00000177725 | 253 | 272 | - | 0.0001 | TTCACAGGCCACACTCCCCA |
| ESR1 | ENSG00000168807 | 367 | 386 | + | 0.0001 | GAGCGGGGAGAGCCTGAGCC |
| ESR1 | ENSG00000183426 | 53 | 72 | + | 0.0001 | CAGCCGGGCCAACCTCAGCC |
| ESR1 | ENSG00000064489 | 326 | 345 | - | 0.0001 | GGCCTTTGTCAGGCCGCCCC |
| ESR1 | ENSG00000166997 | 157 | 176 | - | 0.0001 | GCTACGGGTTTCCCTGCCTC |
| ESR1 | ENSG00000163291 | 135 | 154 | + | 0.0001 | GCGCCCCAGCACCCTGCCCC |
| ESR1 | ENSG00000112079 | 93 | 112 | + | 0.0001 | ACCCAGGTCCAGCCAGCCCT |
| ESR1 | ENSG00000234336 | 271 | 290 | + | 0.0001 | ATTCCGGGGCTCCCCGCCCG |
| ESR1 | ENSG00000063177 | 347 | 366 | - | 0.00011 | GTCGGGGGCCTCTCTGGCCC |
| ESR1 | ENSG00000167608 | 29 | 48 | + | 0.00011 | GCACAAAGGCATCCTGTCCG |
| ESR1 | ENSG00000188818 | 123 | 142 | + | 0.00011 | CCCTCTGCTCAGTGTGGCCT |
| ESR1 | ENSG00000104325 | 126 | 145 | - | 0.00011 | ACGCCCAGCGGCCCTGACCT |
| ESR1 | ENSG00000224665 | 16 | 35 | - | 0.00011 | CGGCCAGGCCTCACCCACCC |
| ESR1 | ENSG00000228021 | 307 | 326 | - | 0.00011 | CCCCCAAATCATCTTGTCCT |
| ESR1 | ENSG00000174775 | 162 | 181 | - | 0.00011 | GTGGCGCGTCACCTTCCCCT |

| | | | | | | |
|---|---|---|---|---|---|---|
| ESR1 | ENSG00000175197 | 102 | 121 | + | 0.00011 | GGCCAATGCCGGCGTGCCAC |
| ESR1 | ENSG00000141753 | 69 | 88 | - | 0.00011 | GGGGGAGGGGGCGCTGTCCT |
| ESR1 | ENSG00000205794 | 174 | 193 | + | 0.00011 | CCGTCCGGGCAGCCGGGCCC |
| ESR1 | ENSG00000143416 | 324 | 343 | + | 0.00011 | TTGCCTGCTCTGTCTGCCCC |
| ESR1 | ENSG00000175344 | 5 | 24 | - | 0.00011 | CGCTCCGGGCACCTCCACCC |
| ESR1 | ENSG00000179409 | 293 | 312 | - | 0.00011 | GGGGAGAGGGACTCTGTCCT |
| ESR1 | ENSG00000166526 | 95 | 114 | + | 0.00011 | TCTCCAGGACACGGTCACTT |
| ESR1 | ENSG00000247670 | 116 | 135 | - | 0.00011 | CGCGCCGGCGACCTCGACCC |
| ESR1 | ENSG00000111335 | 172 | 191 | + | 0.00011 | AGTTTTGGTTTCCCTGCCCC |
| ESR1 | ENSG00000134905 | 54 | 73 | - | 0.00011 | CGGCCAGCGGAGCGCGCCCC |
| ESR1 | ENSG00000114670 | 270 | 289 | - | 0.00011 | AACTAGGGGCAACCACACCC |
| ESR1 | ENSG00000234353 | 168 | 187 | + | 0.00011 | GGAGCAAGTCAACTTGGCTT |
| ESR1 | ENSG00000173402 | 229 | 248 | - | 0.00011 | GTTTGAGACCAGCCTGACCA |
| ESR1 | ENSG00000122565 | 118 | 137 | + | 0.00011 | CAGCCTGGAGATCCAGCCCC |
| ESR1 | ENSG00000084112 | 296 | 315 | - | 0.00011 | CTGCAGGGTCACCAGGGCCA |
| ESR1 | ENSG00000185633 | 7 | 26 | + | 0.00011 | GCCCCAGGTGTGTCCGACTC |
| ESR1 | ENSG00000187109 | 208 | 227 | - | 0.00011 | AGGCCTGGCCCGCGTGCCCG |
| ESR1 | ENSG00000102572 | 136 | 155 | + | 0.00011 | GACCCGCTTCATCCTGACTT |
| ESR1 | ENSG00000189050 | 169 | 188 | - | 0.00011 | GCGCGCGCGCGCCCTGCCCC |
| ESR1 | ENSG00000221025 | 135 | 154 | + | 0.00011 | CCCAGAGTCCTCCCTGCCCC |
| ESR1 | ENSG00000173585 | 185 | 204 | - | 0.00011 | AAGCCAGGCCACCCCTGCCT |
| ESR1 | ENSG00000010030 | 217 | 236 | - | 0.00011 | GTCTCAGCTCCCCCTGTCTC |
| ESR1 | ENSG00000221267 | 305 | 324 | + | 0.00011 | CTATCAGAGCAGCCTGTCAT |
| ESR1 | ENSG00000164542 | 364 | 383 | + | 0.00011 | GTCTCAGCTCAGTGTGTCTC |
| ESR1 | ENSG00000137055 | 34 | 53 | - | 0.00011 | CTGTTAGGTCATCCTCACTG |
| ESR1 | ENSG00000085831 | 127 | 146 | - | 0.00011 | GACCTGCGCCTCCCTGCCCC |
| ESR1 | ENSG00000084234 | 268 | 287 | - | 0.00011 | CGCTTAGCACACCGCGACCC |
| ESR1 | ENSG00000184182 | 226 | 245 | - | 0.00011 | GCGACGGGCCTCTCGGACCT |
| ESR1 | ENSG00000114473 | 214 | 233 | - | 0.00011 | CTAGCGGGTGACGCTGTCCA |
| ESR1 | ENSG00000156931 | 360 | 379 | + | 0.00011 | GCGACAGGTCTCTGAGCCTC |
| ESR1 | ENSG00000163743 | 36 | 55 | - | 0.00011 | GACACACTCCACCATGACCT |
| ESR1 | ENSG00000225721 | 6 | 25 | - | 0.00011 | GCACCGAGTCACAGTGGCTC |
| ESR1 | ENSG00000120805 | 203 | 222 | + | 0.00011 | TGCTCAGGCCACCGTTCCCG |
| ESR1 | ENSG00000144401 | 297 | 316 | + | 0.00011 | GACTAGAGTGACCATGCCTT |
| ESR1 | ENSG00000233436 | 8 | 27 | + | 0.00012 | TACCAGGGTCAGTCAGGCTC |
| ESR1 | ENSG00000151164 | 238 | 257 | + | 0.00012 | GAAACGGAGGACCGTGCCCC |
| ESR1 | ENSG00000103037 | 255 | 274 | - | 0.00012 | CGCCTGGGTCGCCATGGTCT |
| ESR1 | ENSG00000242515 | 277 | 296 | + | 0.00012 | GCGCAGGGTGGACCAGCCCC |
| ESR1 | ENSG00000130714 | 174 | 193 | + | 0.00012 | CACGCGCGTCACCCTCTCCC |
| ESR1 | ENSG00000091039 | 225 | 244 | - | 0.00012 | CTTTCTGGGCACCCAGAACC |
| ESR1 | ENSG00000182199 | 242 | 261 | - | 0.00012 | ACTCCAGGAGATCTAGACCC |
| ESR1 | ENSG00000159199 | 270 | 289 | + | 0.00012 | CTGGGAGGTGAGTCTGTCAC |
| ESR1 | ENSG00000112576 | 80 | 99 | + | 0.00012 | GGGGCGGGGCGGCGTGCGCC |
| ESR1 | ENSG00000251154 | 109 | 128 | - | 0.00012 | CCGCCCAGAGACTGTGACCT |
| ESR1 | ENSG00000231563 | 145 | 164 | + | 0.00012 | GGCCAAGGTCAGAATGATTG |
| ESR1 | ENSG00000083454 | 300 | 319 | - | 0.00012 | CTCCCCTGACACAGTGACAC |
| ESR1 | ENSG00000131759 | 83 | 102 | + | 0.00012 | CGCTCTGCGCCCGCTGACCC |
| ESR1 | ENSG00000167081 | 369 | 388 | + | 0.00012 | GGTGCAGGGGGGCATGGCCC |
| ESR1 | ENSG00000171097 | 141 | 160 | - | 0.00012 | CCCCCATGTCCCCTTGTCAT |
| ESR1 | ENSG00000171163 | 123 | 142 | + | 0.00012 | GCCGCGGGGCTCCGCGACAC |

| | | | | | | |
|---|---|---|---|---|---|---|
| ESR1 | ENSG00000095002 | 308 | 327 | + | 0.00012 | GGGTGTGGTCGCCGTGGCCG |
| ESR1 | ENSG00000147813 | 158 | 177 | - | 0.00012 | AGGACAGACCAGCCAGACTC |
| ESR1 | ENSG00000161970 | 305 | 324 | - | 0.00012 | GTCGGAAGCCACCATGCCCA |
| ESR1 | ENSG00000127616 | 94 | 113 | + | 0.00012 | CCTCCGGGCCACGCCCACCC |
| ESR1 | ENSG00000199719 | 203 | 222 | - | 0.00012 | CATCCAGATCGCCCTCACAT |
| ESR1 | ENSG00000161813 | 134 | 153 | + | 0.00012 | CGCCCAGGCTACAGTGCACT |
| ESR1 | ENSG00000257613 | 281 | 300 | - | 0.00012 | ATGAGTGGGCATAATGCCCT |
| ESR1 | ENSG00000234750 | 237 | 256 | + | 0.00012 | GGCCCTGGGGACCCTGGGAT |
| ESR1 | ENSG00000202441 | 161 | 180 | - | 0.00012 | CAGCAGGGTGTCTCTGAACT |
| ESR1 | ENSG00000149485 | 101 | 120 | - | 0.00012 | TGCCAAGAACACCCTTCCCT |
| ESR1 | ENSG00000124279 | 300 | 319 | - | 0.00012 | CGGCCAGGTCTTTGACACCC |
| ESR1 | ENSG00000243350 | 54 | 73 | - | 0.00012 | GCCCCCGCTCGCCCCGCCCC |
| ESR1 | ENSG00000118260 | 217 | 236 | + | 0.00012 | CGCCGGGCTCAGCCCGCCTT |
| ESR1 | ENSG00000223725 | 224 | 243 | - | 0.00012 | CGCCGGGCTCAGCCCGCCTT |
| ESR1 | ENSG00000213760 | 281 | 300 | - | 0.00012 | CGCCACGATGCCCCTGCCCT |
| ESR1 | ENSG00000105778 | 301 | 320 | - | 0.00012 | GGCCACGGCCGCCCTTCCCT |
| ESR1 | ENSG00000115977 | 318 | 337 | - | 0.00012 | TTCCTTAGGCAACCTGGCCC |
| ESR1 | ENSG00000100316 | 42 | 61 | + | 0.00012 | GTTCAAGACCAGCCTGGCCA |
| ESR1 | ENSG00000163762 | 378 | 397 | - | 0.00012 | GTTCAAGACCAGCCTGGCCA |
| ESR1 | ENSG00000230340 | 128 | 147 | + | 0.00012 | GTTCAAGACCAGCCTGGCCA |
| ESR1 | ENSG00000224892 | 62 | 81 | - | 0.00012 | GGCTCAGGTCCCCTTCAGCT |
| ESR1 | ENSG00000236383 | 378 | 397 | - | 0.00012 | GTTGGAGACCAGCCTGACCA |
| ESR1 | ENSG00000248341 | 296 | 315 | + | 0.00012 | CTTGAGGGGCCAGCTGACCC |
| ESR1 | ENSG00000064787 | 163 | 182 | + | 0.00012 | TCCCCAGGTCAACCCGGCAG |
| ESR1 | ENSG00000240137 | 278 | 297 | + | 0.00012 | GTCACCGATGTCCGTGACCT |
| ESR1 | ENSG00000213397 | 253 | 272 | + | 0.00012 | GGTACCAGTCACCCAGCCAC |
| ESR1 | ENSG00000072518 | 137 | 156 | - | 0.00013 | GTCCACGGCCATCCAGCCAC |
| ESR1 | ENSG00000259163 | 71 | 90 | + | 0.00013 | CCCCAAGCAGTCCCTGCCCC |
| ESR1 | ENSG00000161980 | 11 | 30 | + | 0.00013 | CAGCGAGGCCGCCCAGCCAC |
| ESR1 | ENSG00000217624 | 226 | 245 | - | 0.00013 | CTGCTCGGCCAGTTTGGCCT |
| ESR1 | ENSG00000023191 | 26 | 45 | + | 0.00013 | CGCCCCCGGCACCAAGCCCC |
| ESR1 | ENSG00000207145 | 65 | 84 | + | 0.00013 | GTGAGATGTGATCCTGCCAC |
| ESR1 | ENSG00000221170 | 232 | 251 | + | 0.00013 | GTGAGATGTGATCCTGCCAC |
| ESR1 | ENSG00000258168 | 243 | 262 | + | 0.00013 | TAGCAGAGGGACTCTGGCCT |
| ESR1 | ENSG00000178297 | 323 | 342 | + | 0.00013 | CAGCCTTGTCGTCCTCACCC |
| ESR1 | ENSG00000079819 | 71 | 90 | + | 0.00013 | CCCCCGGGTCTCCGCGGCCG |
| ESR1 | ENSG00000204427 | 298 | 317 | - | 0.00013 | GGCCCCGCCCTCCCTGCCTC |
| ESR1 | ENSG00000233016 | 227 | 246 | + | 0.00013 | GCGGGTGGGCCGCCTGACTT |
| ESR1 | ENSG00000228838 | 179 | 198 | + | 0.00013 | GGAGAAGGGGAGCCTAGCCC |
| ESR1 | ENSG00000255990 | 300 | 319 | - | 0.00013 | GAGCCAGGGCTCAGTGTGCT |
| ESR1 | ENSG00000120159 | 126 | 145 | + | 0.00013 | CCCCCCTGCCACCCCGGCCC |
| ESR1 | ENSG00000249115 | 366 | 385 | + | 0.00013 | GAGTGAGGACACCCCACCCT |
| ESR1 | ENSG00000125848 | 264 | 283 | - | 0.00013 | CTGCAGGCTGAGCTTGTCCT |
| ESR1 | ENSG00000151093 | 365 | 384 | - | 0.00013 | GGCCAGGGGCCTCGGGACCC |
| ESR1 | ENSG00000152464 | 136 | 155 | - | 0.00013 | GGACCGGGTCCCCCCACCCC |
| ESR1 | ENSG00000228925 | 233 | 252 | - | 0.00013 | TCTAGAGCTCACCGTGCCAC |
| ESR1 | ENSG00000230194 | 379 | 398 | - | 0.00013 | ACCACAGGCCCTGCTGACAT |
| ESR1 | ENSG00000126217 | 8 | 27 | - | 0.00013 | CGATGGGAACAGCCTGCCCC |
| ESR1 | ENSG00000184635 | 309 | 328 | - | 0.00013 | CCTGCAGGTCACAGGGCCAC |
| ESR1 | ENSG00000144554 | 294 | 313 | - | 0.00013 | GCCGCAGGGGAGCGGGGCCT |

| | | | | | | |
|---|---|---|---|---|---|---|
| ESR1 | ENSG00000152977 | 81 | 100 | + | 0.00013 | AGGCCAGGTGAGGCCGCGCC |
| ESR1 | ENSG00000251003 | 186 | 205 | - | 0.00013 | CATCCGGTCCAGCATGACTC |
| ESR1 | ENSG00000139626 | 349 | 368 | - | 0.00013 | GGACCAGGTGGGCCAGAGCT |
| ESR1 | ENSG00000105825 | 333 | 352 | - | 0.00013 | ATGCACGGGGACTGTCACCC |
| ESR1 | ENSG00000234695 | 128 | 147 | + | 0.00013 | ATGCACGGGGACTGTCACCC |
| ESR1 | ENSG00000253536 | 80 | 99 | - | 0.00014 | GAGTTGGGGTGCACTGACCT |
| ESR1 | ENSG00000244697 | 49 | 68 | + | 0.00014 | GTTTGAGATCAGCCTGGCCA |
| ESR1 | ENSG00000116213 | 175 | 194 | + | 0.00014 | CCCCTGTGTCGCCTTGTCCC |
| ESR1 | ENSG00000161618 | 36 | 55 | - | 0.00014 | GTGGCCGGGCACGATGGCTC |
| ESR1 | ENSG00000175309 | 92 | 111 | - | 0.00014 | GTCCCGGACGACCCCGCCCC |
| ESR1 | ENSG00000170191 | 354 | 373 | + | 0.00014 | CTCTCTGGGCAGTCCGCCTT |
| ESR1 | ENSG00000145040 | 349 | 368 | + | 0.00014 | GACCCAGGGGACTCTGGGAT |
| ESR1 | ENSG00000233509 | 261 | 280 | - | 0.00014 | CTACCAGGTCATTCAGACGT |
| ESR1 | ENSG00000189308 | 379 | 398 | + | 0.00014 | GGGAAGGGGGAACCGGCCCC |
| ESR1 | ENSG00000168394 | 382 | 401 | + | 0.00014 | GCGCAGGATCAGCCTGTTCC |
| ESR1 | ENSG00000228477 | 103 | 122 | - | 0.00014 | TCCCAGGGCCACCAGGGCCT |
| ESR1 | ENSG00000114383 | 335 | 354 | + | 0.00014 | TGCGCCGGCGGCCCTGACAT |
| ESR1 | ENSG00000128595 | 303 | 322 | + | 0.00014 | CGGCCACGGCATCCTGTGCT |
| ESR1 | ENSG00000221978 | 335 | 354 | - | 0.00014 | CCCGGAGTACAGCCTGTCCC |
| ESR1 | ENSG00000204311 | 295 | 314 | - | 0.00014 | TCTTAAAGTGACCCAGCCCC |
| ESR1 | ENSG00000212464 | 378 | 397 | - | 0.00014 | AGCCTGTGTCAGCCTGTCTG |
| ESR1 | ENSG00000198925 | 310 | 329 | - | 0.00014 | GGCTCGGCTCGGCGCGACCC |
| ESR1 | ENSG00000240950 | 15 | 34 | + | 0.00014 | AGCTAGGGGGATACTGCCAC |
| ESR1 | ENSG00000138794 | 356 | 375 | - | 0.00014 | GCAGCCAGACACCTTGCCCT |
| ESR1 | ENSG00000184205 | 108 | 127 | + | 0.00014 | TGGCCAGGCCGAGCGGACCC |
| ESR1 | ENSG00000021762 | 240 | 259 | - | 0.00014 | GACCCTGCTCCGCGTGGCCT |
| ESR1 | ENSG00000256690 | 150 | 169 | + | 0.00014 | CTAAGGCGCCACCGTGACCC |
| ESR1 | ENSG00000115841 | 342 | 361 | - | 0.00014 | ATGGCAGCTGATACTGACTT |
| ESR1 | ENSG00000258227 | 311 | 330 | - | 0.00014 | AGGCAGGGGCCCCGTCTCCT |
| ESR1 | ENSG00000243341 | 341 | 360 | + | 0.00014 | CCTCAAGGGCAGTCAGACAA |
| ESR1 | ENSG00000169991 | 269 | 288 | + | 0.00015 | CAGCCCGGGCGGTGAGACCC |
| ESR1 | ENSG00000180929 | 44 | 63 | + | 0.00015 | GAGCAGGCTCTGGCTGACTC |
| ESR1 | ENSG00000168818 | 103 | 122 | - | 0.00015 | AGGCACGTGGACGGTGACCC |
| ESR1 | ENSG00000241420 | 198 | 217 | + | 0.00015 | GAGGCTGGGCGTACTGGCCT |
| ESR1 | ENSG00000154889 | 345 | 364 | - | 0.00015 | CTCCCGCGCCGCGCTGCCCC |
| ESR1 | ENSG00000239737 | 252 | 271 | - | 0.00015 | GGTTGAGATCGCTTTGACCC |
| ESR1 | ENSG00000102893 | 204 | 223 | - | 0.00015 | GTCCCGGGGCTGCGCGCCAC |
| ESR1 | ENSG00000155506 | 331 | 350 | - | 0.00015 | TGCCAAGGCCACGCTGTTTT |
| ESR1 | ENSG00000083097 | 314 | 333 | + | 0.00015 | GGTAAAAGCGTCCCTGCCCC |
| ESR1 | ENSG00000198492 | 282 | 301 | - | 0.00015 | GCTCACGGCCTCCCCGCCCC |
| ESR1 | ENSG00000117122 | 245 | 264 | + | 0.00015 | CCCCGGGGACACCCCCCCCC |
| ESR1 | ENSG00000230953 | 324 | 343 | - | 0.00015 | GGCCAAGGGCCCCTTCCCCG |
| ESR1 | ENSG00000129084 | 238 | 257 | - | 0.00015 | CGGCCTGGGCACGCTGGGTC |
| ESR1 | ENSG00000080823 | 163 | 182 | - | 0.00015 | TGCGCAGTGCAGCCCGTCCC |
| ESR1 | ENSG00000127663 | 200 | 219 | - | 0.00015 | GTTGCTGGCGACCGAGCCCT |
| ESR1 | ENSG00000100412 | 290 | 309 | - | 0.00015 | AGTAGGAGGCACCCCGCCCC |
| ESR1 | ENSG00000172936 | 302 | 321 | - | 0.00015 | GAGAAGCGCCGCCCTGCCCT |
| ESR1 | ENSG00000157510 | 307 | 326 | - | 0.00015 | GGCCCCTCTTACCCTGGCCT |
| ESR1 | ENSG00000140961 | 250 | 269 | - | 0.00015 | GGGCATGGTGGCGGTCACCT |
| ESR1 | ENSG00000134508 | 306 | 325 | + | 0.00015 | GTGTAAGGTCATTTTAGCCC |

| | | | | | | |
|---|---|---|---|---|---|---|
| ESR1 | ENSG00000237765 | 104 | 123 | - | 0.00015 | CGCCTGGGGCCCTCTGACAA |
| ESR1 | ENSG00000179583 | 281 | 300 | + | 0.00015 | GAGGAGGGGCTGCCAGACTC |
| ESR1 | ENSG00000198003 | 72 | 91 | + | 0.00015 | AATCCTGGCCAGTCTCACTT |
| ESR1 | ENSG00000010322 | 346 | 365 | - | 0.00015 | GTCACTGCTCTTCCTGGCCT |
| ESR1 | ENSG00000177685 | 123 | 142 | - | 0.00015 | TGGCCGGCTCGCCCAGTCCC |
| ESR1 | ENSG00000244122 | 207 | 226 | - | 0.00015 | AGGCCAGTCCACCCTGCACG |
| ESR1 | ENSG00000248399 | 124 | 143 | + | 0.00015 | GTCCCGGGACTCCGTGGGCT |
| ESR1 | ENSG00000184047 | 144 | 163 | - | 0.00015 | GGCGCTGGCAACCCTGCCCG |
| ESR1 | ENSG00000165792 | 212 | 231 | - | 0.00015 | GGGCCTGGTCACCGGCCCCA |
| ESR1 | ENSG00000112715 | 112 | 131 | - | 0.00015 | GGGACAGGCGAGCCTCAGCC |
| ESR1 | ENSG00000230536 | 148 | 167 | - | 0.00015 | CTGTCATGGCAACCTGATCA |
| ESR1 | ENSG00000143321 | 14 | 33 | - | 0.00016 | TTTACAGATCACTTTGCCTT |
| ESR1 | ENSG00000240210 | 335 | 354 | + | 0.00016 | TGGACAGGATATCCAGCCCC |
| ESR1 | ENSG00000166348 | 150 | 169 | - | 0.00016 | GAGCCAAGGCAACCAGCTCT |
| ESR1 | ENSG00000206535 | 337 | 356 | - | 0.00016 | GCAAGAGGGCCATCTGCCCT |
| ESR1 | ENSG00000258607 | 128 | 147 | + | 0.00016 | CTATTTGGCCACCTTGCCTT |
| ESR1 | ENSG00000168066 | 21 | 40 | + | 0.00016 | AATCCCGGAGAAACTGCCCC |
| ESR1 | ENSG00000182473 | 200 | 219 | + | 0.00016 | GGCCCGGGCTCGCCTCACCT |
| ESR1 | ENSG00000134014 | 139 | 158 | + | 0.00016 | CTTCCAGGTCATCAGGTCAT |
| ESR1 | ENSG00000078304 | 10 | 29 | - | 0.00016 | CCGGCCGGCCGCCCCGCCCC |
| ESR1 | ENSG00000136367 | 279 | 298 | + | 0.00016 | TGGCCAGGGGAGCGAGGCCG |
| ESR1 | ENSG00000258663 | 41 | 60 | + | 0.00016 | TCCAAGAGCCACCCTCCCCT |
| ESR1 | ENSG00000131876 | 227 | 246 | + | 0.00016 | GGCGCGGGGCACGCTGGGAC |
| ESR1 | ENSG00000244604 | 224 | 243 | - | 0.00016 | GGCCCTTTTCAGCACGACCT |
| ESR1 | ENSG00000130294 | 41 | 60 | + | 0.00016 | GAGCCGGGTGTCGGTCCCCC |
| ESR1 | ENSG00000162441 | 353 | 372 | - | 0.00016 | CTTCCGGGCCTACCTGACAA |
| ESR1 | ENSG00000229534 | 207 | 226 | - | 0.00016 | TCTTTAGGAGACTCTGACTT |
| ESR1 | ENSG00000122026 | 236 | 255 | + | 0.00016 | CTTTCCGGGCTGTCTGACAC |
| ESR1 | ENSG00000049883 | 317 | 336 | + | 0.00016 | CTGTCAGCTGCCGCTGCCCT |
| ESR1 | ENSG00000113645 | 37 | 56 | - | 0.00016 | GGGGAAGGAGAGACCGCCCC |
| ESR1 | ENSG00000245281 | 300 | 319 | + | 0.00016 | CCAGCAGGAAATCCTGCCCT |
| ESR1 | ENSG00000256682 | 315 | 334 | + | 0.00016 | CTGAAAGGTCTCCCTGATTG |
| ESR1 | ENSG00000088876 | 128 | 147 | - | 0.00016 | AGGCCCGCCCTCCCTGCCTT |
| ESR1 | ENSG00000220739 | 4 | 23 | + | 0.00016 | GGGCAAAGACAACCTGAGAT |
| ESR1 | ENSG00000231799 | 267 | 286 | - | 0.00016 | TGGCAATGTCACCCCCCCAT |
| ESR1 | ENSG00000130702 | 40 | 59 | - | 0.00017 | CGGCCGGGCGGCCTTAACCC |
| ESR1 | ENSG00000151743 | 378 | 397 | + | 0.00017 | ACGGCGGGTCAGTCAGCTCC |
| ESR1 | ENSG00000147099 | 339 | 358 | - | 0.00017 | TTCCCAGACCAGACTCCCCT |
| ESR1 | ENSG00000238298 | 325 | 344 | - | 0.00017 | TATTGAGGTCTACTTGACAT |
| ESR1 | ENSG00000258559 | 275 | 294 | - | 0.00017 | GGCATGAGCCACCATGCCCG |
| ESR1 | ENSG00000124208 | 117 | 136 | - | 0.00017 | CGGCGAGATCACGCCGCCCA |
| ESR1 | ENSG00000116652 | 289 | 308 | - | 0.00017 | TGGATAGGTTATCCTGTCTC |
| ESR1 | ENSG00000175773 | 295 | 314 | + | 0.00017 | CGGTGGAGCCTGCGTGACCC |
| ESR1 | ENSG00000227638 | 37 | 56 | + | 0.00017 | ACAGAAGGACAGCTTGAGCC |
| ESR1 | ENSG00000129226 | 173 | 192 | + | 0.00017 | GACACTGTTGACTTTGCCCT |
| ESR1 | ENSG00000154783 | 344 | 363 | + | 0.00017 | AAGCCCAGGCTGACTGCCCC |
| ESR1 | ENSG00000211535 | 354 | 373 | + | 0.00017 | GGGTCGGTTCAGCCTTGCCT |
| ESR1 | ENSG00000249144 | 263 | 282 | - | 0.00017 | GATTCTGGTGATAGTGACTC |
| ESR1 | ENSG00000167996 | 322 | 341 | + | 0.00018 | TAGCCAGCCCTCCGTCACCT |
| ESR1 | ENSG00000139163 | 160 | 179 | + | 0.00018 | ATCGCAGGCCAACAAGCCCA |

| | | | | | | |
|---|---|---|---|---|---|---|
| ESR1 | ENSG00000258376 | 179 | 198 | - | 0.00018 | ACTCCTGGGCTCCCTGGGCT |
| ESR1 | ENSG00000007384 | 60 | 79 | - | 0.00018 | GGCAGGGGCCAATCTGCTCT |
| ESR1 | ENSG00000236939 | 258 | 277 | - | 0.00018 | CGCCCAGGGCCGCCTCCGCC |
| ESR1 | ENSG00000180098 | 163 | 182 | + | 0.00018 | CGCGCGGGCCTTCGAGCCCC |
| ESR1 | ENSG00000231365 | 60 | 79 | - | 0.00018 | CAACGCGGTGAGCCTACCCC |
| ESR1 | ENSG00000012048 | 378 | 397 | + | 0.00018 | CGCTCAGGAGGCCTTCACCC |
| ESR1 | ENSG00000218521 | 341 | 360 | - | 0.00018 | GTAACAGTTTAGAGTGACCC |
| ESR1 | ENSG00000173988 | 215 | 234 | + | 0.00018 | AGGGTGTGTCTCCTTGGCCT |
| ESR1 | ENSG00000237259 | 1 | 20 | + | 0.00018 | AAGGCATGGCACCTTCTCCC |
| ESR1 | ENSG00000099953 | 67 | 86 | + | 0.00018 | TGGAAATGGGACCCTGCTCC |
| ESR1 | ENSG00000213443 | 28 | 47 | - | 0.00018 | TGCACAAGTCCCCCTGCCTG |
| ESR1 | ENSG00000158092 | 182 | 201 | + | 0.00018 | CGCGCGGATCCGCCTGCCCA |
| ESR1 | ENSG00000151725 | 259 | 278 | + | 0.00018 | GGAGAGCGGCACCATGGCCC |
| ESR1 | ENSG00000066583 | 127 | 146 | - | 0.00018 | CGGCGATGCCAGCCCGCCCG |
| ESR1 | ENSG00000137877 | 43 | 62 | - | 0.00018 | CTCCCAGGACGCAGAGGCCC |
| ESR1 | ENSG00000126062 | 199 | 218 | + | 0.00018 | AACCAAGGACTCCGTATCCC |
| ESR1 | ENSG00000164306 | 193 | 212 | + | 0.00018 | ACCGCAGCGCGCCCCGCCCC |
| ESR1 | ENSG00000169762 | 185 | 204 | + | 0.00018 | AGCCTGGGGCACCTTCGCTC |
| ESR1 | ENSG00000167094 | 338 | 357 | - | 0.00018 | AGCCTCGGTCTCTCTGCCTG |
| ESR1 | ENSG00000254772 | 109 | 128 | - | 0.00018 | GGGAAGGGGGATTATGACCA |
| ESR1 | ENSG00000108797 | 111 | 130 | - | 0.00018 | CGCGCCGGTTTCCCGGACCC |
| ESR1 | ENSG00000135245 | 3 | 22 | + | 0.00018 | ACTCCAGAACACAATGACTC |
| ESR1 | ENSG00000233789 | 264 | 283 | + | 0.00018 | AACCCAGGCCTATCAGACTC |
| ESR1 | ENSG00000100522 | 120 | 139 | + | 0.00019 | GCCCCCGCTCTCCGAGCCCC |
| ESR1 | ENSG00000197114 | 206 | 225 | - | 0.00019 | GCTCGAGGGTGCCGTGGCCC |
| ESR1 | ENSG00000216895 | 69 | 88 | - | 0.00019 | TTGTGGGGCCATCCTGACAG |
| ESR1 | ENSG00000171346 | 22 | 41 | + | 0.00019 | CCCAGCAGTGACCCAGACCT |
| ESR1 | ENSG00000161960 | 8 | 27 | - | 0.00019 | TGCGGAGGCGACAGTGCCTT |
| ESR1 | ENSG00000115657 | 349 | 368 | - | 0.00019 | GGACCAGGCCTCACCGCCCA |
| ESR1 | ENSG00000251131 | 12 | 31 | - | 0.00019 | GGGTGCTGCGACCATGACCC |
| ESR1 | ENSG00000139173 | 138 | 157 | - | 0.00019 | CGCGCAGGTGACCGCGATCA |
| ESR1 | ENSG00000155957 | 319 | 338 | + | 0.00019 | ATCATGGCTGACCCCGACCC |
| ESR1 | ENSG00000143093 | 97 | 116 | + | 0.00019 | CAGCAGGTTCTTTATGACCT |
| ESR1 | ENSG00000233611 | 257 | 276 | - | 0.00019 | CGGCAAAGGCTTCCTGGCCA |
| ESR1 | ENSG00000126522 | 378 | 397 | - | 0.00019 | CCCCCGGGCCGGGCTGGCTC |
| ESR1 | ENSG00000233937 | 134 | 153 | + | 0.00019 | GTCCGGCCCCACCCTGCCCC |
| ESR1 | ENSG00000256175 | 292 | 311 | + | 0.00019 | GACCCAGGCCCTTCTGTCTC |
| ESR1 | ENSG00000182481 | 286 | 305 | + | 0.00019 | GCCCTATGACTCCCTCCCCT |
| ESR1 | ENSG00000255773 | 170 | 189 | - | 0.00019 | GAGGCAGGGGACCCAGGTCT |
| ESR1 | ENSG00000119638 | 53 | 72 | + | 0.00019 | GGCGTGAGCCACCTTGCCCG |
| ESR1 | ENSG00000189376 | 322 | 341 | - | 0.00019 | GACGCGGGCGGTCCTGACCG |
| ESR1 | ENSG00000234134 | 283 | 302 | + | 0.00019 | TGGAAAGGCGAGCCAGCCAT |
| ESR1 | ENSG00000116857 | 362 | 381 | - | 0.0002 | AGGCTGGGTCCTTCTGATCC |
| ESR1 | ENSG00000143379 | 120 | 139 | + | 0.0002 | GGCCACGCCTACTCTGCCCT |
| ESR1 | ENSG00000121671 | 333 | 352 | - | 0.0002 | CGCCCGGGTGGAGCTGGCAT |
| ESR1 | ENSG00000149564 | 282 | 301 | - | 0.0002 | GCCGCGGGACGCACGGACCT |
| ESR1 | ENSG00000168000 | 92 | 111 | + | 0.0002 | CTCCAAAGGGAGCCTCTCCT |
| ESR1 | ENSG00000243902 | 7 | 26 | - | 0.0002 | CCGCCCGCTCCCGGTGCCCT |
| ESR1 | ENSG00000099622 | 218 | 237 | + | 0.0002 | GGCGAGGGCCTGCCTGCCTG |
| ESR1 | ENSG00000163590 | 218 | 237 | - | 0.0002 | GGCTGGGCTCTGCCTGCCTC |

| | | | | | | |
|---|---|---|---|---|---|---|
| ESR1 | ENSG00000244513 | 53 | 72 | + | 0.0002 | GGGCCAGGGCGCGGGGCCCG |
| ESR1 | ENSG00000226363 | 18 | 37 | + | 0.0002 | GATCTTGGGCAAAATGTCCT |
| ESR1 | ENSG00000151150 | 212 | 231 | - | 0.0002 | CGCCCAGCGCGGCCTATCCT |
| ESR1 | ENSG00000129083 | 328 | 347 | + | 0.0002 | GTGCTAGGTGGCTGTGGCTT |
| ESR1 | ENSG00000174483 | 41 | 60 | + | 2.00E-04 | TGGAAAGGTGGCTCTGACTG |
| ESR1 | ENSG00000257264 | 188 | 207 | + | 2.00E-04 | GGCAAGAGTCACCACGCCCA |
| ESR1 | ENSG00000112096 | 180 | 199 | - | 2.00E-04 | GGCTGGGGTCGGGCTGTCAC |
| ESR1 | ENSG00000204775 | 172 | 191 | + | 2.00E-04 | AGGCCAGCACTCCTTGCCCG |
| ESR1 | ENSG00000253502 | 315 | 334 | - | 2.00E-04 | ATTCAATTTCAGCCTGAACT |
| ESR1 | ENSG00000253950 | 105 | 124 | - | 0.0002 | CTGCGGGCTCACCGTGGTCT |
| ESR1 | ENSG00000186501 | 97 | 116 | - | 0.0002 | GGGCCCGGCCAGCCCCGCCC |
| ESR1 | ENSG00000119640 | 174 | 193 | - | 0.0002 | GGGTCAAGTGACCTTGGGCT |
| ESR1 | ENSG00000248546 | 235 | 254 | - | 0.0002 | ATCAGAGGGCGCCCTGTTCC |
| ESR1 | ENSG00000139351 | 51 | 70 | + | 0.0002 | CGGCCAAAGCACCCTGGGCC |
| ESR1 | ENSG00000240216 | 143 | 162 | - | 0.00021 | CACCCAGAACACTGTTACCT |
| ESR1 | ENSG00000151468 | 309 | 328 | - | 0.00021 | ATCCGGGGGCAGCTTCCCTC |
| ESR1 | ENSG00000124942 | 232 | 251 | - | 0.00021 | CACCCCAGTCTCTGTGCCAT |
| ESR1 | ENSG00000176533 | 263 | 282 | - | 0.00021 | GTTCGCGGGCGCCCTTACCT |
| ESR1 | ENSG00000178922 | 69 | 88 | + | 0.00021 | GCCCTCGGGCAGCCAGCTCT |
| ESR1 | ENSG00000152457 | 251 | 270 | + | 0.00021 | CGGCTGGGGCCACCTGCTCT |
| ESR1 | ENSG00000179988 | 10 | 29 | + | 0.00021 | CAGTCAGGCCAGCCCAGCCC |
| ESR1 | ENSG00000162174 | 295 | 314 | - | 0.00021 | GGACGCGGACGCCGAGACCT |
| ESR1 | ENSG00000241839 | 368 | 387 | + | 0.00021 | GGCTGGGGTCCTCGAGCCCC |
| ESR1 | ENSG00000095139 | 259 | 278 | - | 0.00021 | AGCTCTGGACCTGCTGCCCC |
| ESR1 | ENSG00000092841 | 152 | 171 | - | 0.00021 | GGGCTGGGGCACAATGAATC |
| ESR1 | ENSG00000244468 | 338 | 357 | - | 0.00021 | GTTCGTGGTCTCGCTGGCTC |
| ESR1 | ENSG00000085563 | 121 | 140 | + | 0.00021 | CTTTCAAGCCTGCCTGCCTT |
| ESR1 | ENSG00000000938 | 216 | 235 | + | 0.00021 | CGTGACTGTCTCCCTGCCAC |
| ESR1 | ENSG00000137710 | 264 | 283 | + | 0.00021 | GGCGCCGGGCAGCCCCGCCT |
| ESR1 | ENSG00000175175 | 24 | 43 | - | 0.00021 | AGCTCAGCCCACGCTGCCTA |
| ESR1 | ENSG00000105662 | 294 | 313 | - | 0.00021 | CTCGAAGGCCGCCGTCTCCT |
| ESR1 | ENSG00000256898 | 254 | 273 | + | 0.00021 | GAAACATGGCACTGTTACCT |
| ESR1 | ENSG00000258790 | 152 | 171 | - | 0.00021 | CCGACGGACGAGACTGACCC |
| ESR1 | ENSG00000179256 | 114 | 133 | - | 0.00021 | CTGCTGGGTCCCTCTGCCTG |
| ESR1 | ENSG00000133943 | 109 | 128 | + | 0.00021 | GGCCCCGCGCCCCCAGCCCC |
| ESR1 | ENSG00000067601 | 2 | 21 | - | 0.00021 | CAGGCCGGGCGCCGTGGCTC |
| ESR1 | ENSG00000077157 | 7 | 26 | - | 0.00021 | CAGTGGGGTCACCTTTGCCC |
| ESR1 | ENSG00000033011 | 233 | 252 | + | 0.00021 | GGGACGGGCGGCCGTGAGCT |
| ESR1 | ENSG00000197381 | 276 | 295 | - | 0.00021 | CACGCGGGTCCCGCCGGCCC |
| ESR1 | ENSG00000239775 | 333 | 352 | + | 0.00021 | CTCCCAAGCCAGTGGGCCCC |
| ESR1 | ENSG00000134759 | 155 | 174 | + | 0.00022 | GGGCATGCGCAGTGCGACCC |
| ESR1 | ENSG00000163467 | 332 | 351 | - | 0.00022 | TCGACTGGTCACCTTTTCCT |
| ESR1 | ENSG00000111669 | 50 | 69 | - | 0.00022 | CGCCAGGATCATCCCAACCC |
| ESR1 | ENSG00000213782 | 379 | 398 | + | 0.00022 | TGCTAGGCTCAGGGAGACCC |
| ESR1 | ENSG00000197586 | 267 | 286 | - | 0.00022 | GGCCGGCGCCACCCCGGCCT |
| ESR1 | ENSG00000230741 | 380 | 399 | - | 0.00022 | AGGTTAGGTCACTCACCCAC |
| ESR1 | ENSG00000238278 | 305 | 324 | + | 0.00022 | GGGCCCGGTAGGCCTGCCAT |
| ESR1 | ENSG00000203279 | 182 | 201 | + | 0.00022 | CAGAAAAGGCTCTCTCACCT |
| ESR1 | ENSG00000255581 | 110 | 129 | - | 0.00022 | ATCTCGGCTCACCGCAACCT |
| ESR1 | ENSG00000136383 | 31 | 50 | - | 0.00022 | ATCTCGGCTCACCGCAACCT |

| | | | | | | |
|---|---|---|---|---|---|---|
| ESR1 | ENSG00000011198 | 212 | 231 | + | 0.00022 | ATCTCGGCTCACCGCAACCT |
| ESR1 | ENSG00000145016 | 101 | 120 | + | 0.00022 | GGGCGGGGCCACCGACACTC |
| ESR1 | ENSG00000143384 | 139 | 158 | - | 0.00022 | GTTTTAGGGCGGCCAGTCCT |
| ESR1 | ENSG00000100385 | 220 | 239 | - | 0.00022 | GAAGCAAGGCACCTTCACAT |
| ESR1 | ENSG00000196670 | 377 | 396 | - | 0.00022 | GGGAGGGGGCACGCTTACTC |
| ESR1 | ENSG00000245148 | 196 | 215 | - | 0.00022 | GCCTGAGGACACATTGCTCT |
| ESR1 | ENSG00000119723 | 353 | 372 | - | 0.00022 | CGCATAGCTCTCGCTGCCAC |
| ESR1 | ENSG00000229212 | 359 | 378 | + | 0.00022 | GGCCGAGACCGCCGTGCCCA |
| ESR1 | ENSG00000112305 | 370 | 389 | + | 0.00022 | AGGTGCGTTCACTCTGCCCG |
| ESR1 | ENSG00000229628 | 243 | 262 | + | 0.00022 | GGGATTGGACAGCATGAGCC |
| ESR1 | ENSG00000107371 | 124 | 143 | - | 0.00022 | GGCCGAGGTTAGCGAGGCCG |
| ESR1 | ENSG00000122482 | 162 | 181 | + | 0.00023 | AGCGCTGGGGGAGGTGACCT |
| ESR1 | ENSG00000143569 | 189 | 208 | + | 0.00023 | AGGGCGGGTCGGCCCGACTA |
| ESR1 | ENSG00000185736 | 270 | 289 | + | 0.00023 | TTCCCCGCCCTCCCTGACCA |
| ESR1 | ENSG00000234608 | 316 | 335 | - | 0.00023 | AGGTAAGGGGGAGGTGCCCC |
| ESR1 | ENSG00000135722 | 352 | 371 | - | 0.00023 | AAGTCGGGTCTTCCTGCCGT |
| ESR1 | ENSG00000185963 | 175 | 194 | - | 0.00023 | CGCCGCCGCCGCCCTGCCCC |
| ESR1 | ENSG00000228932 | 339 | 358 | + | 0.00023 | CACACATGTCAGGCTGGCAA |
| ESR1 | ENSG00000204209 | 197 | 216 | - | 0.00023 | TTCCTCTGCCATCCTGACTC |
| ESR1 | ENSG00000164129 | 14 | 33 | - | 0.00023 | GCCGGGGGCGTCCCAGACCT |
| ESR1 | ENSG00000242761 | 61 | 80 | - | 0.00023 | GGCTCAAGTGATCCTCCCAC |
| ESR1 | ENSG00000253729 | 299 | 318 | + | 0.00023 | GCTGCAGGAGACCTTGTCCG |
| ESR1 | ENSG00000197989 | 312 | 331 | - | 0.00023 | AGGTCAGGAGATCGAGACCA |
| ESR1 | ENSG00000185201 | 275 | 294 | - | 0.00023 | AGGTCAGGAGATCGAGACCA |
| ESR1 | ENSG00000252494 | 20 | 39 | - | 0.00023 | AGGTCAGGAGATCGAGACCA |
| ESR1 | ENSG00000242833 | 2 | 21 | - | 0.00023 | AGGTCAGGAGATCGAGACCA |
| ESR1 | ENSG00000215791 | 179 | 198 | - | 0.00023 | GAACCACGTCAGGGTGAACC |
| ESR1 | ENSG00000148303 | 357 | 376 | - | 0.00023 | CCTCGGTGTCTCCGTGCCAC |
| ESR1 | ENSG00000235910 | 26 | 45 | - | 0.00023 | CGCCAAGGCCACCGAGCATC |
| ESR1 | ENSG00000236507 | 353 | 372 | - | 0.00023 | GGCCCAGCTCCCCTTTCCCT |
| ESR1 | ENSG00000107771 | 233 | 252 | - | 0.00024 | CTCCAAAGCCGCCGCGCCCC |
| ESR1 | ENSG00000173465 | 250 | 269 | + | 0.00024 | TGACAACGGCAACATGGCCC |
| ESR1 | ENSG00000237416 | 377 | 396 | + | 0.00024 | GACAAAGGTAAGTATGTCCT |
| ESR1 | ENSG00000155269 | 105 | 124 | + | 0.00024 | GGCCCAGCCCTCACTGGCCA |
| ESR1 | ENSG00000213484 | 91 | 110 | - | 0.00024 | GTTCGAGACCAGCCTGGCCA |
| ESR1 | ENSG00000255354 | 345 | 364 | + | 0.00024 | AGCTAAGGGCTCTCTGGCAG |
| ESR1 | ENSG00000065923 | 206 | 225 | - | 0.00024 | GTCCCGGGGCCCCCCGCGCC |
| ESR1 | ENSG00000242777 | 107 | 126 | + | 0.00024 | GACCCTGTTGAACTTGACTC |
| ESR1 | ENSG00000222383 | 15 | 34 | - | 0.00024 | GGGCTTGGTGGCTGTCACCT |
| ESR1 | ENSG00000156411 | 196 | 215 | - | 0.00024 | AGAAAGGCTCACCCTGGGCT |
| ESR1 | ENSG00000228275 | 129 | 148 | + | 0.00024 | CCCCACGCCCACACCGACCT |
| ESR1 | ENSG00000256223 | 81 | 100 | - | 0.00024 | CGTTCAGGCCACGCTCCGCC |
| ESR1 | ENSG00000176208 | 129 | 148 | - | 0.00024 | CGAGAGGGCCAAAATGACTC |
| ESR1 | ENSG00000223960 | 165 | 184 | - | 0.00024 | GGAGCAGGGCCGCGAGGCCC |
| ESR1 | ENSG00000100926 | 268 | 287 | - | 0.00024 | GGCCAGTCCCACCCTGGCTC |
| ESR1 | ENSG00000003509 | 111 | 130 | + | 0.00024 | CTCCAAAGGCTCCTTGACTG |
| ESR1 | ENSG00000115816 | 354 | 373 | - | 0.00024 | CTCCAAAGGCTCCTTGACTG |
| ESR1 | ENSG00000175066 | 263 | 282 | - | 0.00024 | GTCCCCGGGCGGCCCAACCC |
| ESR1 | ENSG00000254732 | 13 | 32 | - | 0.00024 | CACCCACGTCCCTCTCCCCC |
| ESR1 | ENSG00000166598 | 89 | 108 | - | 0.00024 | TAGCCATGGCAACGTGTTCT |

| | | | | | | |
|---|---|---|---|---|---|---|
| ESR1 | ENSG00000238111 | 324 | 343 | + | 0.00024 | AAGAAGGGTCTGCCTCCCTC |
| ESR1 | ENSG00000160746 | 308 | 327 | - | 0.00024 | TGACCGGGTCTGTCAGCCTC |
| ESR1 | ENSG00000130024 | 364 | 383 | + | 0.00024 | CGGCCGTGCGACAGCGACCC |
| ESR1 | ENSG00000165675 | 294 | 313 | + | 0.00024 | CCGCGAGGGCACTACGAGCC |
| ESR1 | ENSG00000125354 | 138 | 157 | - | 0.00025 | CCCCTCGGTGACCACGTCCT |
| ESR1 | ENSG00000258839 | 321 | 340 | - | 0.00025 | GGTCTTGGTCCGAGTGGCCC |
| ESR1 | ENSG00000242583 | 181 | 200 | + | 0.00025 | CCCCAGGGTCCCCCTCTCCA |
| ESR1 | ENSG00000240857 | 283 | 302 | - | 0.00025 | GCCCGAGGGCCCACCGGCCC |
| ESR1 | ENSG00000238650 | 64 | 83 | - | 0.00025 | CGCCCAATTCCCCCTCCCCC |
| ESR1 | ENSG00000213281 | 262 | 281 | - | 0.00025 | CGCCCCGGCCACGTGGGCCT |
| ESR1 | ENSG00000152465 | 170 | 189 | - | 0.00025 | GAGCCCGGGCGCTACGCCCC |
| ESR1 | ENSG00000233719 | 104 | 123 | + | 0.00025 | CCTACTGTCCACCGTGGCCC |
| ESR1 | ENSG00000166529 | 310 | 329 | - | 0.00025 | CAGCCACGGCTCCCTCTCCT |
| ESR1 | ENSG00000147804 | 38 | 57 | - | 0.00025 | AGGGCTGGGCCCCTTCCCCT |
| ESR1 | ENSG00000204859 | 315 | 334 | - | 0.00025 | AGGCAGGTCCTCCCTGAGCT |
| ESR1 | ENSG00000147677 | 177 | 196 | + | 0.00025 | TCGCCTGACCACCTTCACCT |
| ESR1 | ENSG00000110047 | 268 | 287 | - | 0.00025 | CGCCGACCTCACACTCACCT |
| ESR1 | ENSG00000249633 | 222 | 241 | - | 0.00025 | AGGACAAAGGACCCAGACCC |
| ESR1 | ENSG00000221656 | 115 | 134 | - | 0.00025 | GTCCTGGGGCTCCCAGGCCG |
| ESR1 | ENSG00000132394 | 40 | 59 | + | 0.00025 | AACCCTGCCTACGGTGACCT |
| ESR1 | ENSG00000237779 | 250 | 269 | + | 0.00025 | GCACCATGGCACCCCAGCCT |
| ESR1 | ENSG00000218014 | 298 | 317 | - | 0.00025 | GGCCCAGTGCATGCTGAGCG |
| ESR1 | ENSG00000139211 | 373 | 392 | - | 0.00025 | CGCGCGGGGCGCCCCGCTCC |
| ESR1 | ENSG00000258657 | 145 | 164 | + | 0.00026 | TGGGCATGAGAACTTGCCCC |
| ESR1 | ENSG00000197548 | 355 | 374 | - | 0.00026 | AAACCGGCCCAGCAAGACCC |
| ESR1 | ENSG00000241651 | 291 | 310 | + | 0.00026 | TCCACAGGGCTCCCCACCCC |
| ESR1 | ENSG00000164742 | 257 | 276 | - | 0.00026 | CCGGAGGAGCCGCGTGACCT |
| ESR1 | ENSG00000244005 | 118 | 137 | + | 0.00026 | GGGCTGGGTCGCTCAGCTCT |
| ESR1 | ENSG00000250659 | 340 | 359 | - | 0.00026 | CAGCCTGGGCAACATGGCAA |
| ESR1 | ENSG00000118894 | 128 | 147 | + | 0.00026 | CACGACGTTCCTCCTGACCC |
| ESR1 | ENSG00000142794 | 149 | 168 | + | 0.00026 | GACGCGGCCCATCCGGACCC |
| ESR1 | ENSG00000241635 | 271 | 290 | + | 0.00026 | GCACAGGGTGGACCAGCCCC |
| ESR1 | ENSG00000252150 | 100 | 119 | - | 0.00026 | AGGCCTGCAGATCCTGGCCT |
| ESR1 | ENSG00000227199 | 319 | 338 | - | 0.00026 | GCGGCGAAGCCCCCTGACCC |
| ESR1 | ENSG00000240429 | 214 | 233 | - | 0.00026 | CTCCCGGCTCTGCGTGGCCG |
| ESR1 | ENSG00000215301 | 167 | 186 | + | 0.00026 | CGCCCCGGTCACCATCCTAC |
| ESR1 | ENSG00000181222 | 101 | 120 | + | 0.00026 | GGGCCTGCGCAGGCGGCCCC |
| ESR1 | ENSG00000243305 | 16 | 35 | - | 0.00026 | TGACCTGGTTAGGCTGGCTT |
| ESR1 | ENSG00000120705 | 153 | 172 | - | 0.00026 | TCTCATTGGCTCTCTGCCCC |
| ESR1 | ENSG00000254872 | 329 | 348 | - | 0.00026 | ACACATGGGCACCAGGACAC |
| ESR1 | ENSG00000197892 | 94 | 113 | + | 0.00026 | GGCCCCGCCCACGACGACCC |
| ESR1 | ENSG00000159423 | 131 | 150 | + | 0.00026 | TTGGTGTGTCACTCTGACTG |
| ESR1 | ENSG00000236646 | 290 | 309 | + | 0.00026 | TTGCCATGGGACCCTGTCAG |
| ESR1 | ENSG00000166333 | 154 | 173 | - | 0.00026 | TCCCACCGCCTCCCTGCCCT |
| ESR1 | ENSG00000164053 | 69 | 88 | - | 0.00026 | GACTCAGTGCGGCGCGACCC |
| ESR1 | ENSG00000249540 | 355 | 374 | + | 0.00026 | TACCAGGGACACTGTGAGAT |
| ESR1 | ENSG00000113758 | 289 | 308 | - | 0.00027 | ATTCCAGGCCATTCTCAGCC |
| ESR1 | ENSG00000136883 | 284 | 303 | - | 0.00027 | GTTTCCGGCCCCTCTGGCCC |
| ESR1 | ENSG00000234741 | 307 | 326 | + | 0.00027 | CGGGCGGGGGAGCCAGCGCT |
| ESR1 | ENSG00000180389 | 269 | 288 | - | 0.00027 | AGCTGAGTCCAGCCTGTCTC |

| | | | | | | |
|---|---|---|---|---|---|---|
| ESR1 | ENSG00000103021 | 302 | 321 | - | 0.00027 | GGTGAACGCTACCCTGACTT |
| ESR1 | ENSG00000065328 | 299 | 318 | + | 0.00027 | GGGCCGGGGCCGCCTCGCAC |
| ESR1 | ENSG00000196588 | 29 | 48 | - | 0.00027 | CGAAAACGGCGGCCTGACTT |
| ESR1 | ENSG00000225733 | 78 | 97 | + | 0.00027 | CCGCCAGGGCTCCCGCGCCC |
| ESR1 | ENSG00000171497 | 241 | 260 | - | 0.00027 | CGCCCGGGCCGCCCAAACTC |
| ESR1 | ENSG00000005022 | 340 | 359 | + | 0.00027 | CGCCAAGGACTTCCTGGCAG |
| ESR1 | ENSG00000142875 | 309 | 328 | + | 0.00027 | CGGCCCGGTCTTCGCGCCCG |
| ESR1 | ENSG00000152061 | 306 | 325 | + | 0.00027 | CTCTCAGTTCCCTCCGCCCT |
| ESR1 | ENSG00000233108 | 311 | 330 | + | 0.00027 | GAGCCGGGGCTCCGTCTCTC |
| ESR1 | ENSG00000177628 | 161 | 180 | + | 0.00027 | GAGGAAGGGCTCTGAGTCCC |
| ESR1 | ENSG00000075151 | 362 | 381 | + | 0.00027 | AGGTCCGGTCTCTCCGCCTC |
| ESR1 | ENSG00000159128 | 251 | 270 | + | 0.00027 | CAGCCTGGTGACTCCGTCTC |
| ESR1 | ENSG00000127364 | 86 | 105 | - | 0.00027 | GATCAAGGGCCACCTGATTT |
| ESR1 | ENSG00000233221 | 153 | 172 | + | 0.00027 | CGCGCAGGGCGCCGGGAGCC |
| ESR1 | ENSG00000255857 | 293 | 312 | + | 0.00028 | GTCCCGGTTCCCTGTGCCAC |
| ESR1 | ENSG00000112655 | 156 | 175 | - | 0.00028 | CCCGCACGGCGCCCCGCCCC |
| ESR1 | ENSG00000230042 | 231 | 250 | + | 0.00028 | ATCATGGGGGCCCCAGACCT |
| ESR1 | ENSG00000111229 | 36 | 55 | - | 0.00028 | AGGGCCGGGCATAGTGGCTC |
| ESR1 | ENSG00000160087 | 160 | 179 | + | 0.00028 | TGCCTAGAGCGGCCAGCCCT |
| ESR1 | ENSG00000213398 | 19 | 38 | - | 0.00028 | GCGCCAGGGGAAACAGAGCC |
| ESR1 | ENSG00000153993 | 159 | 178 | + | 0.00028 | GCAGCAGCGCCCCCTGCCTC |
| ESR1 | ENSG00000145022 | 382 | 401 | + | 0.00028 | GACTACGGTGACAGTACCCC |
| ESR1 | ENSG00000126368 | 281 | 300 | + | 0.00028 | TGCGCAGAGCCCTCTGTCCC |
| ESR1 | ENSG00000248121 | 296 | 315 | + | 0.00028 | CGGGCCAGTCAGACTGCGCC |
| ESR1 | ENSG00000163918 | 325 | 344 | + | 0.00028 | TCTTCAGATGTCTCTGCCCT |
| ESR1 | ENSG00000140009 | 380 | 399 | + | 0.00028 | CGCTCAGGTTACAGTCATCC |
| ESR1 | ENSG00000159079 | 77 | 96 | + | 0.00028 | CGCCCTGGCCCCTCCGTCCT |
| ESR1 | ENSG00000241830 | 12 | 31 | - | 0.00028 | GGGTTTGGGCTCCCTAGCCC |
| ESR1 | ENSG00000175206 | 267 | 286 | + | 0.00029 | GTGTGAGGCCAGCTTGAGCA |
| ESR1 | ENSG00000131051 | 124 | 143 | - | 0.00029 | GGCTCCGGCGACCGTAGCCC |
| ESR1 | ENSG00000234740 | 62 | 81 | + | 0.00029 | GTGCGCGGGCGCCCTGGGCT |
| ESR1 | ENSG00000241279 | 213 | 232 | + | 0.00029 | GAGACAGGTTAGTTTTACCC |
| ESR1 | ENSG00000254553 | 128 | 147 | - | 0.00029 | ATCGCGGGAGCGCCTGGCCT |
| ESR1 | ENSG00000235681 | 109 | 128 | - | 0.00029 | ATCTCAGCTCACTGCAACCT |
| ESR1 | ENSG00000256309 | 108 | 127 | + | 0.00029 | GGGGAGGGGCCCAGTGAGCT |
| ESR1 | ENSG00000236474 | 69 | 88 | - | 0.00029 | GCTCAAAGGCACCTTGAAAT |
| ESR1 | ENSG00000259014 | 52 | 71 | + | 0.00029 | ATCTCAGCTCACTGCAACCT |
| ESR1 | ENSG00000201448 | 49 | 68 | - | 0.00029 | GTTTAAGACCAGCCTGGCCA |
| ESR1 | ENSG00000196498 | 93 | 112 | - | 0.00029 | TGGCTCGGCCTCCCTGCTCC |
| ESR1 | ENSG00000134321 | 85 | 104 | - | 0.00029 | CGCTGAGGCGCCCATGGCCT |
| ESR1 | ENSG00000244684 | 280 | 299 | - | 0.00029 | GACACAGCACACTATAACCC |
| ESR1 | ENSG00000175324 | 233 | 252 | - | 0.00029 | GACCCCAGAGTCACTGACCT |
| ESR1 | ENSG00000257851 | 264 | 283 | + | 0.00029 | CAGGAAGAGGGCCATGACCC |
| ESR1 | ENSG00000167491 | 219 | 238 | - | 0.00029 | CTCGCGGCTCAGTCTCACAC |
| ESR1 | ENSG00000232626 | 309 | 328 | - | 0.00029 | CTTTCAGGTCATCTTCCTCC |
| ESR1 | ENSG00000174013 | 371 | 390 | + | 0.00029 | GCAGCCGGTGCGTCTGCCCT |
| ESR1 | ENSG00000078487 | 131 | 150 | + | 0.00029 | GCGATAGGTCATTATAACTC |
| ESR1 | ENSG00000237996 | 244 | 263 | + | 0.00029 | CACCCAGCCCTGAGTGACAT |
| ESR1 | ENSG00000197429 | 253 | 272 | - | 0.00029 | AACGCAGGCCCTCCTTACCC |
| ESR1 | ENSG00000229638 | 83 | 102 | - | 0.00029 | AATCATGATCATTCTGCCTT |

| | | | | | | |
|---|---|---|---|---|---|---|
| ESR1 | ENSG00000047648 | 322 | 341 | - | 0.00029 | CTCCAAAGACTGCGTGCCCA |
| ESR1 | ENSG00000198400 | 324 | 343 | + | 0.0003 | CTGCGGACTCAGCCTGAGCT |
| ESR1 | ENSG00000182993 | 155 | 174 | + | 0.0003 | CTCTTAGCGCCGCTTGACCC |
| ESR1 | ENSG00000114735 | 332 | 351 | + | 0.0003 | CCCAAGGGTCAACCCCACAC |
| ESR1 | ENSG00000240793 | 170 | 189 | - | 0.0003 | CAGCTTGGCCAGTCTCACTC |
| ESR1 | ENSG00000109180 | 102 | 121 | - | 0.0003 | CTGCAATGCCCTTATGACCT |
| ESR1 | ENSG00000204498 | 17 | 36 | + | 0.0003 | TCCCCTGCTCACTCAGCCTC |
| ESR1 | ENSG00000256142 | 94 | 113 | + | 0.0003 | ATCTCAGAGCACTTTGAACC |
| ESR1 | ENSG00000238868 | 10 | 29 | + | 0.0003 | CAGCTGTGTGAGCCTGCACT |
| ESR1 | ENSG00000129465 | 308 | 327 | + | 3.00E-04 | ACCCCTGCACAGACAGACCC |
| ESR1 | ENSG00000119917 | 16 | 35 | - | 0.0003 | TTCTAAGGCCATACTCACCA |
| ESR1 | ENSG00000120533 | 180 | 199 | - | 0.0003 | GGGCGGGGTCTCGCCACCCT |
| ESR1 | ENSG00000122952 | 168 | 187 | + | 0.0003 | CGCGAAGTCGACTGTGACTT |
| ESR1 | ENSG00000122012 | 240 | 259 | + | 0.0003 | TCCCCCGCGCGCCGTGACTC |
| ESR1 | ENSG00000117523 | 342 | 361 | + | 0.0003 | ACCCCAGCTTTCCCTCCCCC |
| ESR1 | ENSG00000115541 | 217 | 236 | + | 0.00031 | GTGCTAGCGCGCTCAGCCCT |
| ESR1 | ENSG00000109189 | 237 | 256 | + | 0.00031 | CCTCCCGGCCTCCCTCTCCC |
| ESR1 | ENSG00000230698 | 6 | 25 | - | 0.00031 | TGACAACGTCACAGTGGCAC |
| ESR1 | ENSG00000142949 | 83 | 102 | - | 0.00031 | CCTCTTGGCCTCTCTGCCTT |
| ESR1 | ENSG00000166278 | 305 | 324 | - | 0.00031 | GGCCCAGGCGTGTCTCGCCC |
| ESR1 | ENSG00000235590 | 311 | 330 | + | 0.00031 | CCGTGGGGTCACTCTTCCTC |
| ESR1 | ENSG00000243243 | 53 | 72 | + | 0.00031 | GACTATGGAAAGCATGACCT |
| ESR1 | ENSG00000133112 | 278 | 297 | + | 0.00031 | CGCGCGGCTCTTCGTGCCAC |
| ESR1 | ENSG00000242588 | 101 | 120 | + | 0.00031 | ATCTGCGGCCTTCGTGCCCC |
| ESR1 | ENSG00000092470 | 101 | 120 | + | 0.00032 | GCGCCTGGCCAAGGCGGCCT |
| ESR1 | ENSG00000237149 | 194 | 213 | + | 0.00032 | GAGCTGAATCACTCCGACCC |
| ESR1 | ENSG00000182472 | 318 | 337 | + | 0.00032 | GACCTGGGGTTTTCTGCCCC |
| ESR1 | ENSG00000240032 | 262 | 281 | - | 0.00032 | AGTCCATCTCACAGTGACTA |
| ESR1 | ENSG00000137522 | 363 | 382 | + | 0.00032 | TTGCAAGGGCACGGTACTCC |
| ESR1 | ENSG00000245532 | 72 | 91 | + | 0.00032 | CGCCTGGGAGACCATGCACC |
| ESR1 | ENSG00000119922 | 30 | 49 | - | 0.00032 | GGGTCTGGGGACATTGCTCT |
| ESR1 | ENSG00000114395 | 296 | 315 | + | 0.00032 | GCGCCTGCGCAGCGCGACTC |
| ESR1 | ENSG00000249098 | 381 | 400 | - | 0.00032 | GGCGGAGCTTACCGTGAGCC |
| ESR1 | ENSG00000235939 | 26 | 45 | - | 0.00032 | AAGTTAGGTCTCTGTCACTC |
| ESR1 | ENSG00000100600 | 228 | 247 | + | 0.00032 | TCACCGCGGCACAGTGGCCC |
| ESR1 | ENSG00000140993 | 284 | 303 | + | 0.00032 | TACCTCGGCCAGCTTGGCTT |
| ESR1 | ENSG00000250893 | 6 | 25 | - | 0.00032 | CATTCAGGTCCCCATCCCCG |
| ESR1 | ENSG00000112339 | 270 | 289 | - | 0.00032 | CCACCATGACGTAGTGACCC |
| ESR1 | ENSG00000160062 | 96 | 115 | - | 0.00032 | TCGCCCACTCCCCCTGCCCT |
| ESR1 | ENSG00000213707 | 160 | 179 | + | 0.00032 | GCACTGGGCGACTGTGCCTC |
| ESR1 | ENSG00000228676 | 25 | 44 | + | 0.00032 | AAGTCAGGAGTTCGTGACCA |
| ESR1 | ENSG00000213703 | 202 | 221 | - | 0.00032 | CTGCTTGGCCACAATGGCCG |
| ESR1 | ENSG00000235706 | 206 | 225 | - | 0.00032 | GCCCGCTGTCTCCCTGCTCC |
| ESR1 | ENSG00000224055 | 171 | 190 | + | 0.00032 | GTGCAGGGCCAGCTGCACCC |
| ESR1 | ENSG00000216324 | 330 | 349 | - | 0.00032 | CTTCCAATTCACATTGCCTT |
| ESR1 | ENSG00000104388 | 145 | 164 | - | 0.00033 | TGCCCGGTCCGCCCTGTCTT |
| ESR1 | ENSG00000199916 | 265 | 284 | - | 0.00033 | AGGTCCGGGGACTTTCCCCT |
| ESR1 | ENSG00000124920 | 54 | 73 | - | 0.00033 | GGCCGCGGGCACCACGCTCC |
| ESR1 | ENSG00000184084 | 374 | 393 | + | 0.00033 | GGACCTGCTGATACTGGCCC |
| ESR1 | ENSG00000249921 | 374 | 393 | + | 0.00033 | CTGTGGGGTCACTCCCACTT |

| | | | | | | |
|---|---|---|---|---|---|---|
| ESR1 | ENSG00000230116 | 338 | 357 | + | 0.00033 | GACGCTAGTCACCAGGACTC |
| ESR1 | ENSG00000248487 | 266 | 285 | + | 0.00033 | AGCCTGGGCCAGCCTGTGCA |
| ESR1 | ENSG00000238711 | 120 | 139 | - | 0.00033 | ATCCCAGGGTTCCAAGGCCC |
| ESR1 | ENSG00000112419 | 33 | 52 | - | 0.00033 | AGCCCCGGGCACTGGCCCCC |
| ESR1 | ENSG00000237806 | 306 | 325 | + | 0.00033 | TGGGAAGATCAACCTGTGCT |
| ESR1 | ENSG00000111845 | 215 | 234 | + | 0.00033 | GAGATATGCCTGCTTGCCCC |
| ESR1 | ENSG00000170289 | 3 | 22 | + | 0.00033 | TCACAAGATCATTTTGACTC |
| ESR1 | ENSG00000244134 | 152 | 171 | + | 0.00033 | CACACATGTGACCATGAGTC |
| ESR1 | ENSG00000254480 | 318 | 337 | - | 0.00033 | GGCAAAGGGGCGTCAGCCCT |
| ESR1 | ENSG00000231770 | 60 | 79 | - | 0.00033 | AAGGCCGGCGTTTCTGACCT |
| ESR1 | ENSG00000110013 | 98 | 117 | + | 0.00034 | GGACTGAGCCATCCTGGCTT |
| ESR1 | ENSG00000184731 | 263 | 282 | - | 0.00034 | GTCCCCGCCCCCCCTCACCT |
| ESR1 | ENSG00000175224 | 2 | 21 | - | 0.00034 | TGGCCGGCGCAATCAGACTT |
| ESR1 | ENSG00000223442 | 143 | 162 | - | 0.00034 | GTCAGAGGTGACGCAGGCAC |
| ESR1 | ENSG00000179195 | 56 | 75 | + | 0.00034 | AATACAGGTCAGCATGAAAA |
| ESR1 | ENSG00000198755 | 354 | 373 | - | 0.00034 | CCCGCAGGCCGACCCGCCTT |
| ESR1 | ENSG00000135114 | 192 | 211 | - | 0.00034 | CCGTCTAGTCAATCTGTCAT |
| ESR1 | ENSG00000235652 | 162 | 181 | + | 0.00034 | GGGGACGCGCACGCCGCCCT |
| ESR1 | ENSG00000258283 | 366 | 385 | - | 0.00034 | TAGCCATGTGAACTTGGCCA |
| ESR1 | ENSG00000120068 | 318 | 337 | - | 0.00034 | CGGCTCGCTCGCCCTCCCCC |
| ESR1 | ENSG00000100142 | 292 | 311 | + | 0.00034 | AACTCAGGGGTCCCCAACCC |
| ESR1 | ENSG00000089127 | 42 | 61 | + | 0.00034 | CTTCCTGGTTTTTCTGACTT |
| ESR1 | ENSG00000142330 | 11 | 30 | - | 0.00034 | GCCCAGGGCTATCCCGGCCT |
| ESR1 | ENSG00000147650 | 48 | 67 | + | 0.00034 | GCGCGCGAGCTCCTTGCCCT |
| ESR1 | ENSG00000088833 | 83 | 102 | - | 0.00035 | TGGCAGGGCCACACTTCCTC |
| ESR1 | ENSG00000153044 | 181 | 200 | + | 0.00035 | GGGGCGGGGCCCTCGGGCCT |
| ESR1 | ENSG00000138061 | 31 | 50 | - | 0.00035 | ACACAGGGTCAGCTTAGCTT |
| ESR1 | ENSG00000077782 | 255 | 274 | + | 0.00035 | AGCGCCGGACAGCTCGGCCC |
| ESR1 | ENSG00000177182 | 121 | 140 | + | 0.00035 | AGACAAAGGCACAATTACCC |
| ESR1 | ENSG00000248394 | 247 | 266 | - | 0.00035 | GCGCGGGGTCGCCATACCCA |
| ESR1 | ENSG00000156970 | 189 | 208 | + | 0.00035 | GGGCGTGGCCACGTCGACCG |
| ESR1 | ENSG00000024048 | 129 | 148 | + | 0.00035 | CTCCTGGGTGTTATTGCCCC |
| ESR1 | ENSG00000254964 | 229 | 248 | + | 0.00035 | AGCCTGGGCCACAATCCCAT |
| ESR1 | ENSG00000241499 | 325 | 344 | - | 0.00035 | CACCATAGTCACTCTGCTTC |
| ESR1 | ENSG00000137821 | 87 | 106 | - | 0.00035 | GTCCTCAGGCACCTTGGCTC |
| ESR1 | ENSG00000124222 | 255 | 274 | - | 0.00035 | CAGCAGGGCCTGCCTCCCCG |
| ESR1 | ENSG00000180398 | 354 | 373 | + | 0.00035 | GCGCTCGGGCTGCCTCGCCT |
| ESR1 | ENSG00000159176 | 52 | 71 | - | 0.00036 | CGAGCTGGTCAGCAGCACCC |
| ESR1 | ENSG00000225302 | 38 | 57 | + | 0.00036 | CTGCCTAGGGAGGCTGCCTC |
| ESR1 | ENSG00000250073 | 269 | 288 | - | 0.00036 | TGCCCAGATCGCCCGAACCC |
| ESR1 | ENSG00000179902 | 63 | 82 | + | 0.00036 | TACCCACGTCGCCTTGACAA |
| ESR1 | ENSG00000080573 | 220 | 239 | + | 0.00036 | CCGAGAAGTCGCCGCGCCCC |
| ESR1 | ENSG00000138382 | 115 | 134 | + | 0.00036 | GGGATGGGGCAATGAGGCCC |
| ESR1 | ENSG00000152672 | 138 | 157 | - | 0.00036 | CTCCCAGCTCTTCCTCCCTC |
| ESR1 | ENSG00000249055 | 147 | 166 | + | 0.00036 | CTCCATGATTACCCTGGCTC |
| ESR1 | ENSG00000041802 | 109 | 128 | + | 0.00036 | CCTCCGAGTCTCCGCGTCCT |
| ESR1 | ENSG00000085274 | 144 | 163 | + | 0.00036 | GCGCATGCCCACTGTGCCCA |
| ESR1 | ENSG00000233558 | 231 | 250 | - | 0.00036 | ATGTCTGGGGTCTCTGCCAC |
| ESR1 | ENSG00000224489 | 136 | 155 | + | 0.00036 | GGAATGTGCCACCATGCCCG |
| ESR1 | ENSG00000198894 | 162 | 181 | - | 0.00036 | GGGCCAGCGCTCAGAGGCCT |

| | | | | | | |
|---|---|---|---|---|---|---|
| ESR1 | ENSG00000101230 | 12 | 31 | - | 0.00036 | GGGCCGGGGGAGCAGGAGCT |
| ESR1 | ENSG00000186615 | 111 | 130 | - | 0.00036 | GTGCCGGGTCCGCCCCGCCC |
| ESR1 | ENSG00000229863 | 126 | 145 | + | 0.00036 | GGTTCTGGCTAAACTGACTT |
| ESR1 | ENSG00000164404 | 367 | 386 | + | 0.00036 | AACACTGCACAACCAGACCC |
| ESR1 | ENSG00000182004 | 49 | 68 | - | 0.00036 | GTGGAAGGTGGGCCGGTCCT |
| ESR1 | ENSG00000134398 | 159 | 178 | - | 0.00036 | AATACAGATCCGCCTGATCT |
| ESR1 | ENSG00000232564 | 136 | 155 | + | 0.00036 | CGATCCACCCACCTTGACCT |
| ESR1 | ENSG00000250487 | 288 | 307 | + | 0.00036 | CTCCGAGGCCAGCTTCACAG |
| ESR1 | ENSG00000225374 | 224 | 243 | + | 0.00036 | TTCCAGGGGCACACACTCCC |
| ESR1 | ENSG00000105694 | 342 | 361 | + | 0.00037 | GGCCCAGGTCAGTCTGCTGA |
| ESR1 | ENSG00000000419 | 132 | 151 | + | 0.00037 | CACACAGGTCATCCCCCGCT |
| ESR1 | ENSG00000168350 | 322 | 341 | + | 0.00037 | GGGCAGGGGCGGCGCGAGCC |
| ESR1 | ENSG00000074416 | 243 | 262 | + | 0.00037 | CGCGCAGCTCGTCCCGGCCC |
| ESR1 | ENSG00000247828 | 286 | 305 | - | 0.00037 | TCTCAAGGTCAGCACCGCCC |
| ESR1 | ENSG00000255046 | 149 | 168 | - | 0.00037 | GGCCCTGGCCAATCAGCGCC |
| ESR1 | ENSG00000049323 | 211 | 230 | - | 0.00037 | GGGCCCGGCGAGGGTGGCCG |
| ESR1 | ENSG00000244580 | 245 | 264 | - | 0.00037 | GGTTGAGATCATTTTGGCCC |
| ESR1 | ENSG00000223505 | 288 | 307 | + | 0.00037 | ATTGAAGGTGTTTATGCCCC |
| ESR1 | ENSG00000153071 | 226 | 245 | - | 0.00037 | AGTCAATCACATGCTGCCCT |
| ESR1 | ENSG00000230955 | 348 | 367 | + | 0.00037 | AGACGGGGTCTCCCTCTCTC |
| ESR1 | ENSG00000244676 | 77 | 96 | - | 0.00037 | TTTATTGGTCTCCCAGACAT |
| ESR1 | ENSG00000186792 | 9 | 28 | - | 0.00037 | CCTCCAGCTGGCTCTGCCTC |
| ESR1 | ENSG00000086189 | 253 | 272 | - | 0.00037 | CGCCCGCACCACTCTGGCCC |
| ESR1 | ENSG00000147439 | 304 | 323 | - | 0.00037 | CGCCTGGGCCTCACTCACCA |
| ESR1 | ENSG00000242941 | 51 | 70 | + | 0.00037 | GTCCAGAGGCACCCAAGCCC |
| ESR1 | ENSG00000164855 | 253 | 272 | - | 0.00037 | GGCCCCCGGCCTCCTGGCCT |
| ESR1 | ENSG00000185513 | 302 | 321 | + | 0.00038 | TCCCCTGGGCGCCGCGCCAC |
| ESR1 | ENSG00000250604 | 114 | 133 | - | 0.00038 | TACAGAGCTGAGGTTGACCC |
| ESR1 | ENSG00000207556 | 195 | 214 | - | 0.00038 | CGCCCAGGCCGCCATTATCT |
| ESR1 | ENSG00000239783 | 122 | 141 | - | 0.00038 | GGAGGAGGGTACCATGGCCA |
| ESR1 | ENSG00000107165 | 133 | 152 | - | 0.00038 | ATTTTAGGGCTTCAAGACCC |
| ESR1 | ENSG00000207314 | 338 | 357 | + | 0.00038 | GGACCAGGGCTGGAAGCCTT |
| ESR1 | ENSG00000197746 | 103 | 122 | + | 0.00038 | CTCCAAGTTCTTCCTCACTC |
| ESR1 | ENSG00000166710 | 163 | 182 | - | 0.00038 | GCCCGCAGGGACCGTCACCT |
| ESR1 | ENSG00000198211 | 186 | 205 | + | 0.00038 | CACCCAAGGCCCCCTGGCAG |
| ESR1 | ENSG00000255730 | 130 | 149 | + | 0.00038 | CTCCCCAGTCTCCCTCCCCA |
| ESR1 | ENSG00000251821 | 245 | 264 | + | 0.00038 | GAACATGGCCCCTGTGACTC |
| ESR1 | ENSG00000153046 | 175 | 194 | - | 0.00038 | AACAAAGGGGTCACTGAACT |
| ESR1 | ENSG00000048828 | 340 | 359 | + | 0.00038 | ACCCCCGGCCCCGCCGCCCC |
| ESR1 | ENSG00000159899 | 10 | 29 | + | 0.00038 | GCGCAGCGCCCTCCTGCCCT |
| ESR1 | ENSG00000255276 | 379 | 398 | - | 0.00038 | GGCCGGGCGGGGGCTGACCC |
| ESR1 | ENSG00000039523 | 135 | 154 | - | 0.00038 | AGTGCTGGCCAACGTGCCAG |
| ESR1 | ENSG00000078018 | 17 | 36 | - | 0.00038 | CGGGAGGGGGAGCACGACCG |
| ESR1 | ENSG00000173163 | 60 | 79 | + | 0.00038 | CGGTAAGCCCTCACTGCCTT |
| ESR1 | ENSG00000156482 | 248 | 267 | + | 0.00038 | TCACAAGTTCACGCTAACAC |
| ESR1 | ENSG00000172590 | 178 | 197 | + | 0.00038 | GCGCGTGGCCACCGAGGCCA |
| ESR1 | ENSG00000230259 | 140 | 159 | - | 0.00038 | CCGGCAGGCCGCGTCGCCCC |
| ESR1 | ENSG00000136521 | 369 | 388 | + | 0.00038 | GTGGCAGCTCTGTCTGGCCG |
| ESR1 | ENSG00000103018 | 93 | 112 | - | 0.00039 | AATTAGGGTCGTTCAGCCCC |
| ESR1 | ENSG00000181789 | 340 | 359 | - | 0.00039 | CAGGCCTGGCCCCCTCACCT |

| | | | | | | |
|---|---|---|---|---|---|---|
| ESR1 | ENSG00000171189 | 279 | 298 | + | 0.00039 | GCACCAACTCACCCTGTACC |
| ESR1 | ENSG00000131381 | 341 | 360 | - | 0.00039 | CCCCAGACTGGGCCTGACCC |
| ESR1 | ENSG00000119509 | 89 | 108 | + | 0.00039 | GCCCCGGTCCACTGAGTCCT |
| ESR1 | ENSG00000116750 | 114 | 133 | + | 0.00039 | TGATAAATTCACCCTGTCTT |
| ESR1 | ENSG00000110492 | 260 | 279 | + | 0.00039 | GAGCCCGGGTTCTGTGGCCC |
| ESR1 | ENSG00000254501 | 235 | 254 | + | 0.00039 | GGGAAGGACCACCGAGACCA |
| ESR1 | ENSG00000221182 | 112 | 131 | - | 0.00039 | ATCCAAAGACACTCTGAATC |
| ESR1 | ENSG00000255467 | 173 | 192 | - | 0.00039 | TCCCCACAGCACTCAGACCC |
| ESR1 | ENSG00000122986 | 284 | 303 | - | 0.00039 | GGCCTAGGGTCCACTGAGCT |
| ESR1 | ENSG00000256804 | 72 | 91 | - | 0.00039 | CAGCAAGAGCTGCTTGCCTC |
| ESR1 | ENSG00000234324 | 74 | 93 | + | 0.00039 | GGCCCAAGCGATCCTCCCAC |
| ESR1 | ENSG00000257727 | 347 | 366 | - | 0.00039 | CGAACAGATCCACGTGACCA |
| ESR1 | ENSG00000258830 | 345 | 364 | - | 0.00039 | TGCAAGAGACATCCTGATCC |
| ESR1 | ENSG00000136492 | 247 | 266 | - | 0.00039 | GACTGGGGCCGCCGTTACCT |
| ESR1 | ENSG00000256326 | 114 | 133 | + | 0.00039 | TGGTCAGGTGGCTCAGATCT |
| ESR1 | ENSG00000109971 | 167 | 186 | - | 0.0004 | TGGTTAGGTGAACCAGGCAT |
| ESR1 | ENSG00000110075 | 374 | 393 | + | 0.0004 | GGCGCGGGGCCCCTCCACCT |
| ESR1 | ENSG00000173457 | 133 | 152 | - | 0.0004 | TCTACAGGCCCCTCTCCCCT |
| ESR1 | ENSG00000119616 | 314 | 333 | - | 0.0004 | GGCCACGGTCTGACTACCTC |
| ESR1 | ENSG00000119682 | 40 | 59 | + | 0.0004 | GGCCACGGTCTGACTACCTC |
| ESR1 | ENSG00000129535 | 59 | 78 | + | 0.0004 | GCCTTAGGACTCAAAGACCC |
| ESR1 | ENSG00000113269 | 255 | 274 | + | 0.0004 | CTCCCGTGCCGCCCTTCCCC |
| ESR1 | ENSG00000255421 | 154 | 173 | - | 0.0004 | TTAGCCGGGCATCGTGGCAC |
| ESR1 | ENSG00000258212 | 159 | 178 | - | 0.0004 | CCTGTGTGTGACACTGACAC |
| ESR1 | ENSG00000134864 | 210 | 229 | - | 0.0004 | AGCTATGGAGAGAGTGACAC |
| ESR1 | ENSG00000138709 | 2 | 21 | - | 0.0004 | GGCTGAGGCCGCCCCGACTG |
| ESR1 | ENSG00000105640 | 336 | 355 | - | 4.00E-04 | CCTGATGGGGCCCGTGCCCC |
| ESR1 | ENSG00000196085 | 16 | 35 | + | 4.00E-04 | ATCCTTGGGTACCTTGAACT |
| ESR1 | ENSG00000113810 | 216 | 235 | + | 4.00E-04 | CCTCTCGGCGAGCCCGCCCT |
| ESR1 | ENSG00000244041 | 68 | 87 | + | 4.00E-04 | GTGCCATTGCACTCTAGCCT |
| ESR1 | ENSG00000169314 | 335 | 354 | + | 0.0004 | TGGCCCTGGGACCCAGCCAT |
| ESR1 | ENSG00000197958 | 223 | 242 | - | 0.0004 | AGGAAGTTGCACCTTGGCCT |
| ESR1 | ENSG00000224281 | 361 | 380 | + | 0.00041 | CGCCCTTGACGCCCAGGCCC |
| ESR1 | ENSG00000115267 | 292 | 311 | - | 0.00041 | GCGCCTGGGGTCCCGGACCG |
| ESR1 | ENSG00000220891 | 58 | 77 | - | 0.00041 | GCTCCGGGACACCCCACCCG |
| ESR1 | ENSG00000255909 | 59 | 78 | + | 0.00041 | GGTGTGGGGTCCCCTGGCCT |
| ESR1 | ENSG00000237813 | 110 | 129 | + | 0.00041 | GGCGCAGGGAACCGTCCCCA |
| ESR1 | ENSG00000170144 | 186 | 205 | + | 0.00041 | GGCAAAGGCCCTCTTCCCCT |
| ESR1 | ENSG00000199509 | 206 | 225 | + | 0.00041 | CGGCCATACCACCCTGAACA |
| ESR1 | ENSG00000054148 | 147 | 166 | + | 0.00041 | CGCCCCGCGCCCCCTACCCC |
| ESR1 | ENSG00000115211 | 98 | 117 | - | 0.00042 | TGCCCAGGCGATTGCGTCCC |
| ESR1 | ENSG00000155755 | 288 | 307 | - | 0.00042 | CGCAGCGGCCTCCGGGACCT |
| ESR1 | ENSG00000114270 | 59 | 78 | - | 0.00042 | CTGAGAGGTCGCCGCCCCCC |
| ESR1 | ENSG00000149735 | 67 | 86 | - | 0.00042 | CACCTGGGGGATCTTGCTCT |
| ESR1 | ENSG00000199545 | 66 | 85 | + | 0.00042 | GAGCCAGGAGAGTGTGAAAT |
| ESR1 | ENSG00000076321 | 366 | 385 | + | 0.00042 | GGGACAGCGGACAGCGCCCC |
| ESR1 | ENSG00000060339 | 246 | 265 | - | 0.00042 | CCTAAGGGTTGCCTTGGCCT |
| ESR1 | ENSG00000229951 | 320 | 339 | + | 0.00042 | CTCCAGGTACCCCCAGACCC |
| ESR1 | ENSG00000184979 | 17 | 36 | + | 0.00042 | ACCTGAGACCAGCCTGGCCA |
| ESR1 | ENSG00000181163 | 167 | 186 | - | 0.00042 | GGGTGGGCCGACTCTGACTT |

| | | | | | | |
|---|---|---|---|---|---|---|
| ESR1 | ENSG00000253966 | 178 | 197 | + | 0.00042 | CCCCCAGCTGACTATGCACC |
| ESR1 | ENSG00000137642 | 22 | 41 | + | 0.00042 | CCACCGGCGCTCGCTGCCTT |
| ESR1 | ENSG00000249042 | 121 | 140 | + | 0.00042 | CCTCCAGCGGATGCTGCCAT |
| ESR1 | ENSG00000237380 | 238 | 257 | - | 0.00043 | AGCCTGGGGCGCCGACCCCC |
| ESR1 | ENSG00000102882 | 299 | 318 | + | 0.00043 | GACCCATGGCCCCCGAACCT |
| ESR1 | ENSG00000109046 | 62 | 81 | - | 0.00043 | CAGGCTGGTTCCGCTGACTC |
| ESR1 | ENSG00000093100 | 237 | 256 | + | 0.00043 | GAGTCAGTCCAGGTTCACCT |
| ESR1 | ENSG00000241343 | 80 | 99 | - | 0.00043 | TCCTCTGGCCTCCCCGTCCC |
| ESR1 | ENSG00000003056 | 127 | 146 | + | 0.00043 | GCCCGAGGGCGAGCTGCCTG |
| ESR1 | ENSG00000258653 | 77 | 96 | - | 0.00043 | AGGCCTCGGCCCTCTGACCG |
| ESR1 | ENSG00000233579 | 346 | 365 | - | 0.00043 | CACCCAGGACACCCCGAAAG |
| ESR1 | ENSG00000077097 | 156 | 175 | + | 0.00043 | GGGGGCGGCCGCCGCGCCCT |
| ESR1 | ENSG00000187741 | 186 | 205 | - | 0.00043 | GCCCGAGCCCGCGCTGCCTT |
| ESR1 | ENSG00000119684 | 165 | 184 | - | 0.00044 | ATCCCAGGGTTCAGTGAACC |
| ESR1 | ENSG00000196141 | 324 | 343 | - | 0.00044 | ACACATGGGCATCGTGGGCT |
| ESR1 | ENSG00000186352 | 362 | 381 | + | 0.00044 | CACTCGGGACAGCCATCCCC |
| ESR1 | ENSG00000106336 | 179 | 198 | - | 0.00044 | AGTGCAGCTCATTCTCTCCC |
| ESR1 | ENSG00000157657 | 27 | 46 | - | 0.00044 | GCCCCCCGCCGCCCTGCCCG |
| ESR1 | ENSG00000159409 | 270 | 289 | - | 0.00044 | CCGCTGGGGCTGCCTGCTTT |
| ESR1 | ENSG00000247853 | 341 | 360 | - | 0.00044 | CTGCCCTGCCTCCCTGTCCA |
| ESR1 | ENSG00000154146 | 77 | 96 | + | 0.00044 | AAGCCAGGGGATTGCGCCCG |
| ESR1 | ENSG00000234076 | 93 | 112 | - | 0.00044 | GGGCGGGGCCACCACCTCCT |
| ESR1 | ENSG00000133111 | 137 | 156 | + | 0.00044 | GGCGCCGCGCTCCCTGACAG |
| ESR1 | ENSG00000121067 | 227 | 246 | - | 0.00044 | TCCCTGGGTTTCCCTGCTCC |
| ESR1 | ENSG00000233547 | 209 | 228 | + | 0.00044 | CAGAGAGGTCTCCCCAACAT |
| ESR1 | ENSG00000258725 | 146 | 165 | - | 0.00044 | TGGGGAGGTGTTCCTGCTCT |
| ESR1 | ENSG00000240766 | 252 | 271 | - | 0.00044 | AGTCAAGTTCTACCAGTCCT |
| ESR1 | ENSG00000111291 | 63 | 82 | - | 0.00044 | CCGGAAGGACAGAAAGACAC |
| ESR1 | ENSG00000130717 | 257 | 276 | - | 0.00044 | GCATCGGGTCCCCGCGCCCG |
| ESR1 | ENSG00000253720 | 204 | 223 | - | 0.00045 | GGTCCGCGGCGCCCTCACTT |
| ESR1 | ENSG00000243916 | 236 | 255 | - | 0.00045 | TCCCAGGGCCCCCGAGTCCT |
| ESR1 | ENSG00000255905 | 299 | 318 | - | 0.00045 | CCTCCTAGGCAGCCTCCCAC |
| ESR1 | ENSG00000089159 | 155 | 174 | - | 0.00045 | GTGCACGCGCGCCCCGCCCC |
| ESR1 | ENSG00000247315 | 42 | 61 | + | 0.00045 | GGCGCTGGCCAATATGCGCC |
| ESR1 | ENSG00000155508 | 135 | 154 | - | 0.00045 | GCGGCGGGCCGCCCTCAGCC |
| ESR1 | ENSG00000236997 | 112 | 131 | + | 0.00045 | TACACTGGTCAGAATGGTCT |
| ESR1 | ENSG00000224985 | 77 | 96 | + | 0.00046 | GCGCCTGCGCATCCACACCC |
| ESR1 | ENSG00000227694 | 343 | 362 | + | 0.00046 | AGCCGAAGACACTGCGACTC |
| ESR1 | ENSG00000250900 | 8 | 27 | - | 0.00046 | CGTGCGGGTGGCCCTGGACC |
| ESR1 | ENSG00000204390 | 351 | 370 | - | 0.00046 | AAGGCGGGTCTCCGTGACGA |
| ESR1 | ENSG00000058091 | 67 | 86 | + | 0.00046 | TCTCAGGCCCAGCATGTCCC |
| ESR1 | ENSG00000198431 | 309 | 328 | - | 0.00046 | CCCTCGGTCGTACCTGACCT |
| ESR1 | ENSG00000235529 | 294 | 313 | - | 0.00046 | ACAAAAGGTCTCCCAGCCTG |
| ESR1 | ENSG00000185112 | 378 | 397 | + | 0.00046 | CGGCCAGGCCAGCCCGGACA |
| ESR1 | ENSG00000138400 | 127 | 146 | + | 0.00046 | CAGCCATGACAGCTTCCCCG |
| ESR1 | ENSG00000236256 | 293 | 312 | - | 0.00046 | CCCAAATGCCATCATGAACT |
| ESR1 | ENSG00000115415 | 353 | 372 | - | 0.00046 | TGAAAAGGTCTCTTTTACCC |
| ESR1 | ENSG00000167588 | 138 | 157 | - | 0.00046 | GCCCCAGCCCAGCTTCCCAC |
| ESR1 | ENSG00000204438 | 101 | 120 | + | 0.00046 | TTGTCAGGTCCCTTTCACCG |
| ESR1 | ENSG00000035115 | 98 | 117 | + | 0.00047 | ATGCAAGGATACACTTCCCC |

| | | | | | | |
|---|---|---|---|---|---|---|
| ESR1 | ENSG00000114942 | 181 | 200 | + | 0.00047 | CGGGAAGGACAACTTGGACC |
| ESR1 | ENSG00000249715 | 72 | 91 | + | 0.00047 | AGGCCCAGTCACCTATACCC |
| ESR1 | ENSG00000207437 | 349 | 368 | + | 0.00047 | TCCTGATGACAGTCTGCCTC |
| ESR1 | ENSG00000250963 | 357 | 376 | + | 0.00047 | TGCAACTGACATCCTCACCT |
| ESR1 | ENSG00000242095 | 119 | 138 | + | 0.00047 | GCGCCCGGCCAATCTTACTT |
| ESR1 | ENSG00000096093 | 150 | 169 | + | 0.00047 | ATGCCTGCGCCTCCTGGCCC |
| ESR1 | ENSG00000101654 | 150 | 169 | + | 0.00047 | GCGTCGCGGCACTCTCGCCT |
| ESR1 | ENSG00000223522 | 346 | 365 | + | 0.00047 | TGCCTGGTGCAGCCTGGTCT |
| ESR1 | ENSG00000203466 | 337 | 356 | - | 0.00047 | ATTCAAGACCAGCCTGATCA |
| ESR1 | ENSG00000212496 | 355 | 374 | - | 0.00047 | GGCGTGAGCCACCGTGCCTG |
| ESR1 | ENSG00000137210 | 56 | 75 | + | 0.00047 | GGCGCTAGCCGCGATGCCCC |
| ESR1 | ENSG00000065150 | 122 | 141 | + | 0.00048 | TAGCAGGAGCCAGCTGACCT |
| ESR1 | ENSG00000148737 | 20 | 39 | + | 0.00048 | CCCCCCGGCTTCCCCGCCCC |
| ESR1 | ENSG00000244336 | 345 | 364 | - | 0.00048 | AGCCCGGGAGATCCTGCATC |
| ESR1 | ENSG00000138297 | 230 | 249 | - | 0.00049 | CCTACTTCACACGCTGACCT |
| ESR1 | ENSG00000172954 | 328 | 347 | + | 0.00049 | GGCGTCGCGCGGCGTGCCCT |
| ESR1 | ENSG00000178685 | 143 | 162 | + | 0.00049 | GGGCACTCCCTCCATGACCT |
| ESR1 | ENSG00000224884 | 345 | 364 | - | 0.00049 | GAGCCACTGCACCCAGCCAT |
| ESR1 | ENSG00000131508 | 214 | 233 | + | 0.00049 | GACTCCGGCCAGCCTGAGTG |
| ESR1 | ENSG00000164466 | 309 | 328 | + | 0.00049 | ACCATACGCCACCATGCCCA |
| ESR1 | ENSG00000116455 | 21 | 40 | + | 0.0005 | CACCCGGGACAGCATGGTCA |
| ESR1 | ENSG00000236439 | 201 | 220 | + | 0.0005 | CGCACATGAGAACATGCCTC |
| ESR1 | ENSG00000136044 | 95 | 114 | + | 0.0005 | CGGGACTGGCACCCAGAGCC |
| ESR1 | ENSG00000072310 | 1 | 20 | + | 0.0005 | CGGCCAGAGGCCGCTGCCAT |
| ESR1 | ENSG00000010404 | 2 | 21 | - | 0.0005 | CGCTGCAGGCACCCTATCCC |
| ESR1 | ENSG00000230371 | 54 | 73 | - | 0.0005 | CGGCCTGGTGGCATTGCTCT |
| ESR1 | ENSG00000112739 | 352 | 371 | - | 0.0005 | CGCGGCGGCCATCTTGAACT |
| ESR1 | ENSG00000255471 | 83 | 102 | + | 0.0005 | GAAACTGGGCAGCCAGCCAG |
| ESR1 | ENSG00000065970 | 92 | 111 | - | 0.0005 | GAGCCGGGAGACGCCGAGCC |
| ESR1 | ENSG00000071082 | 377 | 396 | - | 0.0005 | TCCCAGGGTCACTAAGACGC |
| ESR1 | ENSG00000213592 | 242 | 261 | + | 0.0005 | GCCCCTGGGCCGCATCTCCT |
| ESR1 | ENSG00000255729 | 319 | 338 | - | 0.00051 | GCCGCTTGGCAGAGTGATCC |
| ESR1 | ENSG00000255782 | 371 | 390 | - | 0.00051 | TTTCAGGGAGATTCTCACCT |
| ESR1 | ENSG00000061455 | 127 | 146 | + | 0.00051 | TCTTCTGGCCTGCCTCCCCT |
| ESR1 | ENSG00000059378 | 252 | 271 | - | 0.00051 | AGCAAAGGACCCTCTCACAC |
| ESR1 | ENSG00000166181 | 10 | 29 | + | 0.00051 | TTCCCCGCCCACCCACACCC |
| ESR1 | ENSG00000185567 | 282 | 301 | + | 0.00051 | CGCGACGGGCCGCCAGGCCC |
| ESR1 | ENSG00000233927 | 291 | 310 | - | 0.00051 | GCTCCGTGTGTCCGGGACCC |
| ESR1 | ENSG00000249825 | 349 | 368 | + | 0.00051 | TGCCAAGGTGAGCATGGAAC |
| ESR1 | ENSG00000142856 | 114 | 133 | - | 0.00051 | GGCCCGCGGCGCCCTCCCTC |
| ESR1 | ENSG00000052841 | 270 | 289 | + | 0.00051 | GCTCCGGCCCAGGCTGGCTC |
| ESR1 | ENSG00000136108 | 370 | 389 | - | 0.00051 | CGGACTGCGCCCTCTGACTC |
| ESR1 | ENSG00000244462 | 58 | 77 | + | 0.00051 | CCGCCAGGGCCACTAGCCCA |
| ESR1 | ENSG00000114544 | 29 | 48 | + | 0.00051 | CAGCCAGGCCGCTCACACTC |
| ESR1 | ENSG00000205268 | 214 | 233 | - | 0.00051 | CCGCCGTGACTCCCTTCCCT |
| ESR1 | ENSG00000258588 | 288 | 307 | - | 0.00052 | AATCCAAGGCACCTTAAACT |
| ESR1 | ENSG00000227051 | 282 | 301 | + | 0.00052 | CTCGCTGCTCAGCCCGATCC |
| ESR1 | ENSG00000170889 | 3 | 22 | - | 0.00052 | ATTTCGGCTCACTGAGGCCC |
| ESR1 | ENSG00000149716 | 89 | 108 | + | 0.00052 | CAGCGCGGACAGACAGGCCC |
| ESR1 | ENSG00000177981 | 356 | 375 | - | 0.00052 | GAGCCACCGCACCCAGCCAC |

| | | | | | | |
|---|---|---|---|---|---|---|
| ESR1 | ENSG00000257894 | 46 | 65 | + | 0.00052 | AAAGATGGTGACCCACCCCT |
| ESR1 | ENSG00000166046 | 84 | 103 | + | 0.00052 | GCCAGACGGCACAGAGCCCT |
| ESR1 | ENSG00000113593 | 317 | 336 | - | 0.00052 | CCTCCGGGTCCCGGCGCCTT |
| ESR1 | ENSG00000139289 | 264 | 283 | + | 0.00052 | AAATATGGCCATTTTGACAT |
| ESR1 | ENSG00000184117 | 330 | 349 | - | 0.00052 | CGGTCAGGTGGGCGTTTCCT |
| ESR1 | ENSG00000182944 | 44 | 63 | + | 0.00052 | CACACCCGGCCGCGTGACCC |
| ESR1 | ENSG00000259118 | 340 | 359 | + | 0.00053 | CCTCCAGGACACTCATACTT |
| ESR1 | ENSG00000124444 | 284 | 303 | - | 0.00053 | CCCCGTGGTTTCCGCGCCCT |
| ESR1 | ENSG00000226763 | 59 | 78 | - | 0.00053 | CCCCGTGGTTTCCGCGCCCT |
| ESR1 | ENSG00000168484 | 65 | 84 | - | 0.00053 | CACCCGGCGCGGTCAGCCCC |
| ESR1 | ENSG00000224660 | 138 | 157 | - | 0.00053 | AGCCCAGGAGTTCGAGACCA |
| ESR1 | ENSG00000242257 | 140 | 159 | + | 0.00053 | ATGGAATCTCACTCTGTCAC |
| ESR1 | ENSG00000142541 | 307 | 326 | - | 0.00053 | GCCCCTTGCCGCCCCGGCCC |
| ESR1 | ENSG00000224623 | 174 | 193 | - | 0.00053 | GTTTGAGACCAGCCTGGCCA |
| ESR1 | ENSG00000207185 | 377 | 396 | + | 0.00053 | ATCTCAGCTCACTGCACCCT |
| ESR1 | ENSG00000123349 | 380 | 399 | - | 0.00053 | CCGGGAGGCCTCGCTGCGCC |
| ESR1 | ENSG00000254452 | 86 | 105 | - | 0.00054 | TGGCTTGGTTTCTGTGTCCT |
| ESR1 | ENSG00000245750 | 299 | 318 | - | 0.00054 | GGATCTGGTGCTCGAGACCT |
| ESR1 | ENSG00000114391 | 15 | 34 | + | 0.00054 | AGACAGAGACAGAATGACTC |
| ESR1 | ENSG00000242683 | 244 | 263 | + | 0.00054 | GGGCGTGGTGGCTCTCGCCT |
| ESR1 | ENSG00000164543 | 42 | 61 | - | 0.00054 | GGGTGATGAGATCCTGATCT |
| ESR1 | ENSG00000258232 | 159 | 178 | - | 0.00054 | TTTAAATGTCGAGCTGACTT |
| ESR1 | ENSG00000073910 | 249 | 268 | + | 0.00054 | GGCCGGAGGCTGCCTGCACC |
| ESR1 | ENSG00000178878 | 212 | 231 | - | 0.00054 | CGCCCCGCGCACCCCCGCCT |
| ESR1 | ENSG00000229127 | 203 | 222 | - | 0.00054 | AACCTAGGAGACAATGGCCA |
| ESR1 | ENSG00000174748 | 179 | 198 | + | 0.00054 | ATCCCTGGGCGCTGGGGCCT |
| ESR1 | ENSG00000139624 | 324 | 343 | + | 0.00054 | GCCCTGGGGCCGCCTCCCCG |
| ESR1 | ENSG00000255692 | 299 | 318 | - | 0.00054 | CTGAAATGCCGTCGTGGCCC |
| ESR1 | ENSG00000232739 | 227 | 246 | + | 0.00055 | GGACCAGTGCTACCCGGCCT |
| ESR1 | ENSG00000237827 | 134 | 153 | - | 0.00055 | CACTCAGATTACTCTCACTT |
| ESR1 | ENSG00000010361 | 64 | 83 | - | 0.00055 | TTCCAAGTTGAGGACGACCT |
| ESR1 | ENSG00000093009 | 163 | 182 | - | 0.00055 | TCGTAAGGACAATGGGCCCT |
| ESR1 | ENSG00000224424 | 188 | 207 | - | 0.00055 | CCGCCGGGGCTCACGGAGCT |
| ESR1 | ENSG00000233885 | 174 | 193 | + | 0.00055 | ATCCACTGACAGCCTCACTC |
| ESR1 | ENSG00000087470 | 221 | 240 | - | 0.00055 | GCCCGCCGGCTACCTGCCCC |
| ESR1 | ENSG00000174527 | 263 | 282 | + | 0.00055 | AATCCAAGGTAATGTGACTT |
| ESR1 | ENSG00000100442 | 109 | 128 | - | 0.00055 | GGACCAGACCACGTTCACTT |
| ESR1 | ENSG00000196542 | 299 | 318 | + | 0.00055 | CGCCACTCTCAGCAAGCCCT |
| ESR1 | ENSG00000133027 | 1 | 20 | + | 0.00056 | ATCCAAGGGGATACTGCTAC |
| ESR1 | ENSG00000148634 | 97 | 116 | + | 0.00056 | GCCTGTGGCCAGCCACCCCC |
| ESR1 | ENSG00000167100 | 291 | 310 | + | 0.00056 | CCGTCAGGTCTCCTGGTCCA |
| ESR1 | ENSG00000256885 | 198 | 217 | + | 0.00056 | CTCCTGGGCTACAGTGATCC |
| ESR1 | ENSG00000111249 | 241 | 260 | - | 0.00056 | GCCGCCGGCCGCCCGGGCCC |
| ESR1 | ENSG00000183273 | 85 | 104 | + | 0.00056 | GTTCACGACCCCCATGACCC |
| ESR1 | ENSG00000089775 | 42 | 61 | + | 0.00056 | AGCCTCGGGCTCCGGGACAC |
| ESR1 | ENSG00000198189 | 16 | 35 | - | 0.00056 | CTCGCCTGTGATCCTGGCAC |
| ESR1 | ENSG00000249494 | 300 | 319 | - | 0.00056 | GTTAGATGACGCCCTAACCT |
| ESR1 | ENSG00000104549 | 8 | 27 | + | 0.00056 | CGCGCAGGTTGCGCCGCCTC |
| ESR1 | ENSG00000201207 | 62 | 81 | - | 0.00056 | ATTTCAGCTCACTGCAACCT |
| ESR1 | ENSG00000257918 | 193 | 212 | - | 0.00057 | TCCCCTGAGTACACTGGCCT |

| | | | | | | |
|---|---|---|---|---|---|---|
| ESR1 | ENSG00000254909 | 150 | 169 | - | 0.00057 | TTGCTCTGTCACCCAGACTG |
| ESR1 | ENSG00000226769 | 295 | 314 | + | 0.00057 | TCCTCCATTGACCTTGACCT |
| ESR1 | ENSG00000001631 | 52 | 71 | + | 0.00057 | CGGGAAGCGCTGCGTGAGCC |
| ESR1 | ENSG00000257755 | 6 | 25 | - | 0.00057 | TGCCCAGGCCTGATTCACAC |
| ESR1 | ENSG00000256029 | 133 | 152 | - | 0.00058 | CTGTAAGCGCCCTGTGGCCT |
| ESR1 | ENSG00000174579 | 340 | 359 | - | 0.00058 | GTCAGAGGGCTCTAAGGCCC |
| ESR1 | ENSG00000159082 | 125 | 144 | - | 0.00058 | GACCACGCCCACTCTGCGCC |
| ESR1 | ENSG00000162458 | 230 | 249 | - | 0.00058 | AGCTCGGGTGTCGATGTCAC |
| ESR1 | ENSG00000204856 | 2 | 21 | + | 0.00058 | GCCCGCGGGGCCCATGACCA |
| ESR1 | ENSG00000232021 | 213 | 232 | + | 0.00058 | GGGCAGCGGCCCGCTCACCT |
| ESR1 | ENSG00000197375 | 75 | 94 | + | 0.00058 | TCGCCAGGTCCCCAGGACAG |
| ESR1 | ENSG00000102401 | 335 | 354 | + | 0.00059 | GCCCCAGGGGAAGATGCCGT |
| ESR1 | ENSG00000256329 | 194 | 213 | + | 0.00059 | AGCCCAGGGACCTTTGGCCT |
| ESR1 | ENSG00000110108 | 204 | 223 | + | 0.00059 | GCTCTAGAACTACATGACCC |
| ESR1 | ENSG00000115419 | 45 | 64 | + | 0.00059 | CTCTCCGCGCACCCAGAGCC |
| ESR1 | ENSG00000236810 | 155 | 174 | - | 0.00059 | ACGTCAGTTCCGCTCGACCC |
| ESR1 | ENSG00000240695 | 103 | 122 | + | 0.00059 | GTTGGAGACCAGCCTGGCCA |
| ESR1 | ENSG00000230005 | 2 | 21 | + | 0.0006 | CAGCAAAGCCACCCTGCTTG |
| ESR1 | ENSG00000167552 | 27 | 46 | - | 0.0006 | GGAAAATGACTGCCAGCCCC |
| ESR1 | ENSG00000252412 | 88 | 107 | - | 0.0006 | TGCCAGGGTCACTGGGTGCT |
| ESR1 | ENSG00000257076 | 26 | 45 | - | 0.0006 | GTGGCCAGGCACAGTGGCTC |
| ESR1 | ENSG00000143256 | 90 | 109 | - | 0.0006 | GGCCGCGGTGAATCCCACCT |
| ESR1 | ENSG00000176973 | 273 | 292 | - | 0.0006 | GAGAAAAGGCAACGGGACAT |
| ESR1 | ENSG00000232633 | 309 | 328 | - | 0.0006 | AAGGCTGGAGAATCTGCCTC |
| ESR1 | ENSG00000214753 | 165 | 184 | + | 6.00E-04 | TGTTTTGGGCTCCGTGAGCC |
| ESR1 | ENSG00000224356 | 132 | 151 | + | 6.00E-04 | TTGTTCTGTCAGCCTCACTT |
| ESR1 | ENSG00000239688 | 251 | 270 | + | 6.00E-04 | GTAAGAGGACCGCCTGAGCC |
| ESR1 | ENSG00000245521 | 201 | 220 | + | 0.0006 | AGCCCAGGACGCTAGGCCTT |
| ESR1 | ENSG00000241762 | 283 | 302 | + | 0.0006 | ACGTCATTTCATCTTCACCC |
| ESR1 | ENSG00000053747 | 358 | 377 | - | 0.00061 | CGCCTGCGCCTCCCGGACCT |
| ESR1 | ENSG00000220378 | 12 | 31 | - | 0.00061 | ATCTCGGCTCACTGCAACCT |
| ESR1 | ENSG00000168092 | 201 | 220 | - | 0.00061 | ACTTCCGGTTCCCCTGCCAC |
| ESR1 | ENSG00000139719 | 193 | 212 | - | 0.00061 | ACGGCAGCGCACGCCAACCC |
| ESR1 | ENSG00000256861 | 95 | 114 | - | 0.00061 | ACGGCAGCGCACGCCAACCC |
| ESR1 | ENSG00000257337 | 93 | 112 | + | 0.00061 | GCCCCCTGGCGGCCTCGCCT |
| ESR1 | ENSG00000121749 | 379 | 398 | - | 0.00061 | GTCCCTCGTGTCCCTCCCCC |
| ESR1 | ENSG00000226963 | 371 | 390 | - | 0.00061 | CTCACGGTGCACCCCGTCTC |
| ESR1 | ENSG00000249310 | 38 | 57 | + | 0.00061 | CTGTGATCTCGCCCTGCCTC |
| ESR1 | ENSG00000013810 | 83 | 102 | - | 0.00061 | GGCTCCGCCCTCCCTCCCCC |
| ESR1 | ENSG00000234141 | 107 | 126 | + | 0.00061 | GCGTAAGCTCAGTGTGATTC |
| ESR1 | ENSG00000254088 | 194 | 213 | + | 0.00061 | ACAGAAGGTCACCCCAGCTC |
| ESR1 | ENSG00000215217 | 37 | 56 | + | 0.00062 | ACACGTGGCCACCGAGAGCT |
| ESR1 | ENSG00000242180 | 369 | 388 | + | 0.00062 | TGGCAATGGCACCCTCCTTC |
| ESR1 | ENSG00000242219 | 114 | 133 | - | 0.00062 | GGCTCATGCGATCCTCCCAT |
| ESR1 | ENSG00000250068 | 210 | 229 | + | 0.00063 | GTGGATGGCGAGTAAGACCT |
| ESR1 | ENSG00000180815 | 105 | 124 | + | 0.00063 | GTGCCGGGACCGCCTGCGCG |
| ESR1 | ENSG00000135473 | 79 | 98 | + | 0.00063 | CCACCGGCGCCCCGTAACCT |
| ESR1 | ENSG00000250656 | 287 | 306 | + | 0.00063 | CGCCATGGTGGCCACCACCT |
| ESR1 | ENSG00000225839 | 261 | 280 | + | 0.00063 | GAGGCCAGGCACAGTGGCTC |
| ESR1 | ENSG00000234233 | 292 | 311 | - | 0.00064 | TTCGAAGGTGAGCCTTAACT |

| | | | | | | |
|---|---|---|---|---|---|---|
| ESR1 | ENSG00000173113 | 48 | 67 | + | 0.00064 | CGCACACGCCAGCTAGTCCC |
| ESR1 | ENSG00000128645 | 227 | 246 | - | 0.00064 | CGGCCACGTGGCGCTGGCCG |
| ESR1 | ENSG00000159884 | 155 | 174 | - | 0.00064 | CGGGGAGGCCACGCCCACTC |
| ESR1 | ENSG00000256349 | 376 | 395 | + | 0.00064 | AAGATGTGTGGCCTTGCCCC |
| ESR1 | ENSG00000094880 | 271 | 290 | + | 0.00064 | GCGGCAGTGGCGCCTGTCCT |
| ESR1 | ENSG00000133872 | 191 | 210 | + | 0.00064 | GGCCCCGCCCACACCGCCTC |
| ESR1 | ENSG00000140416 | 56 | 75 | - | 0.00065 | GCGGCCGGAGAGGCCGCCCT |
| ESR1 | ENSG00000110446 | 20 | 39 | + | 0.00065 | AGGCTTGTTGAAAGTGCCCC |
| ESR1 | ENSG00000089737 | 182 | 201 | + | 0.00065 | GGGGCGGGACTTCCTCCCCA |
| ESR1 | ENSG00000223916 | 173 | 192 | - | 0.00065 | CTCCGCGCTCACCGCCACCC |
| ESR1 | ENSG00000214188 | 11 | 30 | + | 0.00065 | GGCCCGCGGCGCCCACCCCT |
| ESR1 | ENSG00000166839 | 294 | 313 | + | 0.00065 | GGCCGAGGCCGAGAGGACCC |
| ESR1 | ENSG00000174374 | 285 | 304 | - | 0.00066 | CGACAGGCTCACTGCAACCT |
| ESR1 | ENSG00000186298 | 287 | 306 | - | 0.00066 | CCCCCCTGCCACCCCGCTCC |
| ESR1 | ENSG00000090006 | 380 | 399 | + | 0.00066 | GATCCTGGACTATCCGCCCT |
| ESR1 | ENSG00000152580 | 105 | 124 | + | 0.00066 | AAAGAAGGTCTCCCAGATTC |
| ESR1 | ENSG00000120306 | 137 | 156 | + | 0.00066 | AGGACAGAAGATTCTGTCCC |
| ESR1 | ENSG00000250234 | 359 | 378 | - | 0.00066 | GACCCCTCTGGGCCTGCCCC |
| ESR1 | ENSG00000183495 | 369 | 388 | - | 0.00066 | CCGGCCGGCGAGCGGGCCCT |
| ESR1 | ENSG00000175416 | 27 | 46 | + | 0.00066 | AGCCCAGGTGTCCTCGACGC |
| ESR1 | ENSG00000218186 | 338 | 357 | - | 0.00066 | TGCCCAGGCCACCTTGGAAG |
| ESR1 | ENSG00000197620 | 103 | 122 | + | 0.00066 | GCCCCGCCTCTCCCAGCCCC |
| ESR1 | ENSG00000100099 | 40 | 59 | + | 0.00066 | CCCCCGGGGCGCCGCCTCCC |
| ESR1 | ENSG00000162623 | 62 | 81 | - | 0.00067 | GGAGAAGGTGTGGCAGTCCT |
| ESR1 | ENSG00000229117 | 96 | 115 | + | 0.00067 | GAACTAACTCAACCTACCCT |
| ESR1 | ENSG00000227528 | 160 | 179 | - | 0.00067 | GGAGGAAGTCAGCATGAGTC |
| ESR1 | ENSG00000238862 | 2 | 21 | - | 0.00067 | GCGGCCAGGCACGGTGGCTC |
| ESR1 | ENSG00000119608 | 40 | 59 | - | 0.00067 | ATGCCAGGGCTCATGAACCT |
| ESR1 | ENSG00000182177 | 115 | 134 | - | 0.00067 | TGCGGAGGGCCACCTGCCAG |
| ESR1 | ENSG00000125991 | 379 | 398 | - | 0.00067 | CCTCCACGCGACCCCGGCCC |
| ESR1 | ENSG00000114302 | 259 | 278 | - | 0.00067 | TACCACGGCCGACCTGGCAC |
| ESR1 | ENSG00000235105 | 238 | 257 | + | 0.00067 | AAGATGAATCACTCAGACCT |
| ESR1 | ENSG00000118263 | 167 | 186 | - | 0.00067 | GCCAATTGTTTGCTTGACCT |
| ESR1 | ENSG00000232073 | 86 | 105 | - | 0.00067 | TCTCAAGGACACTCAAGCCT |
| ESR1 | ENSG00000254208 | 317 | 336 | - | 0.00067 | GGTAACGGGCGCTTTGCCCG |
| ESR1 | ENSG00000225951 | 361 | 380 | + | 0.00068 | GGCTTGCCTCACAATGCCCT |
| ESR1 | ENSG00000248168 | 74 | 93 | + | 0.00068 | AGGGCAGCTGACAGCGGCCC |
| ESR1 | ENSG00000171121 | 376 | 395 | - | 0.00068 | CAGCGGGAGCCCCCAGCCCC |
| ESR1 | ENSG00000189337 | 169 | 188 | - | 0.00068 | CAAGAAGGACACCGCGCGCC |
| ESR1 | ENSG00000241853 | 231 | 250 | + | 0.00068 | AAGAAGGGCTATTCTGCCAT |
| ESR1 | ENSG00000250641 | 28 | 47 | + | 0.00068 | AGTGGTGGTCACTTTATCCC |
| ESR1 | ENSG00000254633 | 122 | 141 | - | 0.00068 | TGCAGGACTCTGCCTGCCCC |
| ESR1 | ENSG00000162889 | 316 | 335 | + | 0.00068 | CCCACGGAGCCCCGCGACCC |
| ESR1 | ENSG00000234264 | 295 | 314 | - | 0.00068 | AACCCTTGCCAGACTCACCG |
| ESR1 | ENSG00000249007 | 39 | 58 | + | 0.00068 | CACCCTGGCCTCTGTTCCCC |
| ESR1 | ENSG00000254400 | 361 | 380 | - | 0.00068 | AATCCAGGGGACTCGGCGCC |
| ESR1 | ENSG00000228604 | 119 | 138 | + | 0.00068 | AAATCAGATCATCTTCACTT |
| ESR1 | ENSG00000163900 | 99 | 118 | - | 0.00068 | GACTCCGCCCTCTCTGTCCT |
| ESR1 | ENSG00000149380 | 154 | 173 | - | 0.00069 | CGCCCCTGTCTCCGCCCCCT |
| ESR1 | ENSG00000226677 | 364 | 383 | + | 0.00069 | ATGTTATCGCAGCTTGACTT |

| | | | | | | |
|---|---|---|---|---|---|---|
| ESR1 | ENSG00000254140 | 100 | 119 | + | 0.00069 | TTTCCCAGGCAACGGGACCC |
| ESR1 | ENSG00000258395 | 320 | 339 | - | 0.00069 | GTACAAAGACACGCGGACTC |
| ESR1 | ENSG00000160712 | 195 | 214 | - | 0.00069 | AACAGGGGACCGCCAGCCCC |
| ESR1 | ENSG00000078140 | 378 | 397 | - | 0.00069 | TCCCTCGGCGATTCAGACCT |
| ESR1 | ENSG00000141219 | 29 | 48 | + | 0.00069 | GGAAAGGGGGTCCCTTCCCT |
| ESR1 | ENSG00000138433 | 15 | 34 | - | 0.00069 | GGCGCGGCACTGCTTGACCG |
| ESR1 | ENSG00000227617 | 132 | 151 | - | 0.00069 | GAGCCACCACACCCGGCCCC |
| ESR1 | ENSG00000171604 | 223 | 242 | + | 0.00069 | GAGGAAGGTCGGCCTTGCAC |
| ESR1 | ENSG00000105793 | 248 | 267 | + | 0.00069 | CTTTGGGTTCATACTGCCTT |
| ESR1 | ENSG00000230424 | 252 | 271 | - | 0.0007 | CCCCAGACTCACCTTTACCC |
| ESR1 | ENSG00000236404 | 307 | 326 | - | 0.0007 | GTGCCAGGCTACTGAGTCAC |
| ESR1 | ENSG00000155744 | 353 | 372 | - | 0.0007 | TTTCCAGACCACCCTCGCCA |
| ESR1 | ENSG00000127080 | 368 | 387 | + | 0.0007 | CTGACAGGCGCTCCTCCCCC |
| ESR1 | ENSG00000171401 | 303 | 322 | + | 0.00071 | GGCCCAGGCCAAGCAAGCTT |
| ESR1 | ENSG00000258974 | 271 | 290 | - | 0.00071 | GTCCATGGCCACGTTGCTCA |
| ESR1 | ENSG00000140525 | 289 | 308 | - | 0.00071 | CTCTGCAGTCACCTCGGCCT |
| ESR1 | ENSG00000135631 | 128 | 147 | - | 0.00071 | AACCGAGGGCGCCCTCTGCT |
| ESR1 | ENSG00000224189 | 285 | 304 | - | 0.00071 | CGACGTGCTCAGCTTGGCAC |
| ESR1 | ENSG00000187747 | 380 | 399 | + | 0.00071 | AGGCAATGGCATCCTAATTT |
| ESR1 | ENSG00000162643 | 316 | 335 | - | 0.00072 | ACCTCAGGTCCTACTGAATC |
| ESR1 | ENSG00000163661 | 234 | 253 | + | 0.00072 | TATTAAGGACTCTCTGCTCC |
| ESR1 | ENSG00000257529 | 12 | 31 | - | 0.00072 | TGGTCCACTCACCCCGACTT |
| ESR1 | ENSG00000177119 | 121 | 140 | + | 0.00072 | TGCTCAGGGAACCCAGTCCA |
| ESR1 | ENSG00000204264 | 279 | 298 | - | 0.00072 | CACCCACAAGAGCGTGCCCT |
| ESR1 | ENSG00000232371 | 22 | 41 | + | 0.00072 | GGCGTGAGCCACCATGCCTG |
| ESR1 | ENSG00000135317 | 160 | 179 | - | 0.00073 | CCTGAGGGGCAACCGGAGCT |
| ESR1 | ENSG00000133316 | 375 | 394 | + | 0.00073 | GGAACGGTGCACTCTCCCTT |
| ESR1 | ENSG00000254876 | 16 | 35 | - | 0.00073 | GGACCCGCGCTCCCAGTCCC |
| ESR1 | ENSG00000103550 | 227 | 246 | - | 0.00073 | AGCCAGCTCCGCCGTGACCC |
| ESR1 | ENSG00000167085 | 233 | 252 | - | 0.00074 | ACCTGCTTCCACTCTGACCT |
| ESR1 | ENSG00000198496 | 308 | 327 | + | 0.00074 | AGAAGAGGTCCCAATCCCCC |
| ESR1 | ENSG00000142669 | 204 | 223 | - | 0.00074 | CTCAGATGTCCCCCTGGTCC |
| ESR1 | ENSG00000167553 | 147 | 166 | - | 0.00074 | GGCCGCGGGCAGCCCGTCTG |
| ESR1 | ENSG00000250317 | 347 | 366 | + | 0.00074 | TGCAAATTGCAAACTGACAT |
| ESR1 | ENSG00000182575 | 201 | 220 | + | 0.00075 | CGCGCAGGTCCCCAGCACAT |
| ESR1 | ENSG00000242247 | 64 | 83 | - | 0.00075 | GCAGAAGGTGCCTCTGTCAC |
| ESR1 | ENSG00000204310 | 31 | 50 | - | 0.00075 | TTGGCATTGCACATTGACTT |
| ESR1 | ENSG00000254673 | 246 | 265 | + | 0.00075 | GGCCACGTGCAGCCGGCCAC |
| ESR1 | ENSG00000133193 | 156 | 175 | - | 0.00075 | TGTTCAGCCCAGCGTCACAC |
| ESR1 | ENSG00000238567 | 124 | 143 | + | 0.00075 | GTTCCAGGCTGCAGTGAGCT |
| ESR1 | ENSG00000100154 | 87 | 106 | + | 0.00075 | GGCGGGGGTGGGCGAGACAC |
| ESR1 | ENSG00000235897 | 102 | 121 | + | 0.00075 | TCTCCAGGGCAACAGGGCCG |
| ESR1 | ENSG00000164180 | 55 | 74 | + | 0.00075 | CATACAGGTGTCCAGGACCG |
| ESR1 | ENSG00000198804 | 319 | 338 | + | 0.00075 | GAGCTGGGCCAGCCAGGCAA |
| ESR1 | ENSG00000143164 | 234 | 253 | - | 0.00075 | GCCGCCGGTGCCCGAGGCCT |
| ESR1 | ENSG00000247708 | 305 | 324 | + | 0.00075 | CGGAGAGCTCAGCGAGCTCT |
| ESR1 | ENSG00000226059 | 153 | 172 | - | 0.00075 | AGCGCAGGGCACAATGCATG |
| ESR1 | ENSG00000235848 | 303 | 322 | - | 0.00076 | CGCCAAAGTTTCAATGCCAT |
| ESR1 | ENSG00000224502 | 250 | 269 | + | 0.00076 | GTCCGAGGTCACACAGCGTG |
| ESR1 | ENSG00000202314 | 241 | 260 | - | 0.00076 | GGCCATTTTCATTCAGCCCC |

| | | | | | | |
|---|---|---|---|---|---|---|
| ESR1 | ENSG00000229759 | 350 | 369 | + | 0.00076 | AGGCCGTATCACAGCGACTC |
| ESR1 | ENSG00000112182 | 106 | 125 | + | 0.00076 | CGCCGGGCGTACCGCGCCCC |
| ESR1 | ENSG00000185684 | 279 | 298 | - | 0.00077 | AGCCAAGGGCTCCCGGCGCG |
| ESR1 | ENSG00000105877 | 313 | 332 | - | 0.00077 | TCCACAGCGCCCACTGCCTC |
| ESR1 | ENSG00000152778 | 50 | 69 | - | 0.00077 | GCAGCTGGGCTCCTTCACTT |
| ESR1 | ENSG00000242251 | 267 | 286 | + | 0.00077 | GGCCCAGGAGTTCCAGACTA |
| ESR1 | ENSG00000213741 | 310 | 329 | - | 0.00077 | CAGACAGTTCTAAGTGCCTT |
| ESR1 | ENSG00000214283 | 243 | 262 | + | 0.00077 | CAGTTTGGCGACTCTGACAG |
| ESR1 | ENSG00000224020 | 146 | 165 | - | 0.00077 | TGCTCAGCTCGCATTGAACT |
| ESR1 | ENSG00000226784 | 380 | 399 | + | 0.00078 | GAAGAGAGTGATCCGGACCC |
| ESR1 | ENSG00000223998 | 127 | 146 | - | 0.00078 | GTTCGAGACCAGGCTGGCCA |
| ESR1 | ENSG00000079337 | 4 | 23 | - | 0.00078 | AGTTAAGCACACCCTGTCTA |
| ESR1 | ENSG00000197728 | 49 | 68 | - | 0.00078 | CCCCCCTTCCCCCCTGCCCC |
| ESR1 | ENSG00000145741 | 4 | 23 | + | 0.00078 | AGGACCGCGCTCTCCGACCC |
| ESR1 | ENSG00000234084 | 298 | 317 | - | 0.00078 | CCAAAAAGTCTCCTTGCTCT |
| ESR1 | ENSG00000055609 | 75 | 94 | + | 0.00078 | ACCCCGGGGGCCGGCGCCCC |
| ESR1 | ENSG00000111237 | 372 | 391 | - | 0.00079 | GGGCCCTGACGCCGAGGCCT |
| ESR1 | ENSG00000010256 | 59 | 78 | + | 0.00079 | GTTTGATATCAGCCTGGCCA |
| ESR1 | ENSG00000219790 | 171 | 190 | - | 0.00079 | AGCAAGGGCCATTCTCAGCC |
| ESR1 | ENSG00000257534 | 194 | 213 | + | 0.00079 | CAGACAGGGCTCTCCCTCCT |
| ESR1 | ENSG00000138768 | 362 | 381 | + | 0.00079 | AGGGCGGGGGAAGTTGTCTT |
| ESR1 | ENSG00000169239 | 44 | 63 | - | 0.00079 | AACCAGGTCCACGCTCTCCC |
| ESR1 | ENSG00000171302 | 339 | 358 | + | 0.0008 | AGCCAAGCCCAGCCAAGCCC |
| ESR1 | ENSG00000183684 | 70 | 89 | + | 0.0008 | TCCCCGCCTCACCCCGTCCC |
| ESR1 | ENSG00000257355 | 362 | 381 | - | 0.0008 | CCGCATGCTCACCATTTCCT |
| ESR1 | ENSG00000152056 | 222 | 241 | - | 0.0008 | CTGCCGCGCGAGTGTGTCCT |
| ESR1 | ENSG00000240596 | 190 | 209 | - | 0.0008 | CCTCCAGCCCTTTCTGCCAT |
| ESR1 | ENSG00000215695 | 330 | 349 | + | 0.00081 | GACTCAGATCGCATTGAACC |
| ESR1 | ENSG00000012822 | 249 | 268 | - | 0.00081 | AGCAAAGGACTTCAAGGCCC |
| ESR1 | ENSG00000113068 | 38 | 57 | - | 0.00081 | CGGTCAGGTCTGGCTTGCTC |
| ESR1 | ENSG00000225251 | 178 | 197 | + | 0.00081 | GATGCAGGTCTCTGTTCCAC |
| ESR1 | ENSG00000207601 | 74 | 93 | + | 0.00081 | ATCGCAGGACTACAAGTCCC |
| ESR1 | ENSG00000207110 | 36 | 55 | + | 0.00081 | CTTCAAGGATCCCCTGTCAT |
| ESR1 | ENSG00000178796 | 17 | 36 | + | 0.00081 | TCTAGAGGTGTGTCTGCCTC |
| ESR1 | ENSG00000243364 | 265 | 284 | - | 0.00081 | GGTCCGGTTTGGTCTGGCCT |
| ESR1 | ENSG00000173451 | 123 | 142 | + | 0.00081 | TACACTCGTCTCACAGACCT |
| ESR1 | ENSG00000239607 | 208 | 227 | - | 0.00082 | GGCATGCGCCACCACGCCCC |
| ESR1 | ENSG00000121578 | 306 | 325 | - | 0.00082 | GAGGCTGGTCCGCCCGGCTC |
| ESR1 | ENSG00000127837 | 117 | 136 | - | 0.00082 | GCCCCACCTGACTCCGCCCC |
| ESR1 | ENSG00000127838 | 131 | 150 | + | 0.00082 | GCCCCACCTGACTCCGCCCC |
| ESR1 | ENSG00000183531 | 267 | 286 | + | 0.00082 | CCCCTAGGTGAGCTAAACAC |
| ESR1 | ENSG00000175455 | 357 | 376 | + | 0.00082 | CTGGCGGGTCCTCGTCCCCG |
| ESR1 | ENSG00000129484 | 197 | 216 | + | 0.00083 | CCGCCTAGTGACACTGGGCC |
| ESR1 | ENSG00000215424 | 58 | 77 | - | 0.00083 | AGCCCCGCCCCTCATGCCCC |
| ESR1 | ENSG00000108094 | 160 | 179 | + | 0.00083 | GAAGCTTGGCTCCCTGCGCT |
| ESR1 | ENSG00000219545 | 89 | 108 | - | 0.00083 | TTCCGGACTCATCCAGCCCC |
| ESR1 | ENSG00000244307 | 68 | 87 | - | 0.00083 | GCAGCTTGTCTGCTTGCCTT |
| ESR1 | ENSG00000124302 | 315 | 334 | - | 0.00083 | GGCGCGGGGCGGCGTTCCTC |
| ESR1 | ENSG00000128590 | 241 | 260 | - | 0.00083 | GAGGAGGGCCGCCCTGCATC |
| ESR1 | ENSG00000238300 | 281 | 300 | + | 0.00083 | CAGCTAGGACTGAGTGTCTC |

| | | | | | | |
|---|---|---|---|---|---|---|
| ESR1 | ENSG00000243156 | 147 | 166 | + | 0.00084 | GCGCCCGCTCGCGTTGCCAC |
| ESR1 | ENSG00000134982 | 175 | 194 | - | 0.00084 | GTGAGAGGTGTTGCTGGCTT |
| ESR1 | ENSG00000073614 | 268 | 287 | - | 0.00084 | GACGCCTGTGTGTCTGGCCT |
| ESR1 | ENSG00000087111 | 83 | 102 | - | 0.00084 | CTCTTAGGGTGCCCTCTCCT |
| ESR1 | ENSG00000172543 | 332 | 351 | + | 0.00084 | AGGTAAGTGCTCCCAAACCC |
| ESR1 | ENSG00000196357 | 237 | 256 | + | 0.00084 | AGACACGCGCACGCAGACAC |
| ESR1 | ENSG00000168386 | 285 | 304 | - | 0.00084 | GCTGCAGGATGAGGTGACCC |
| ESR1 | ENSG00000232398 | 239 | 258 | - | 0.00084 | GTGGAAGGCTAGCTTGCCAG |
| ESR1 | ENSG00000106682 | 68 | 87 | - | 0.00085 | CTGCCCGGCCTACGCGGCCC |
| ESR1 | ENSG00000248772 | 311 | 330 | + | 0.00085 | CCCCAAACCCAGACTGATCT |
| ESR1 | ENSG00000253256 | 47 | 66 | - | 0.00086 | GGCGCCTGTCACAGTGCGCG |
| ESR1 | ENSG00000230534 | 85 | 104 | + | 0.00086 | GGAGACGGCGAGCCCGACCG |
| ESR1 | ENSG00000072210 | 303 | 322 | - | 0.00086 | TTCCCTGGAGAGCTAGTCCC |
| ESR1 | ENSG00000120738 | 59 | 78 | + | 0.00086 | ACTCCGGGTCCTCCCGGCCG |
| ESR1 | ENSG00000162244 | 83 | 102 | - | 0.00087 | CGCCCCCTTCAGGCTGCCTC |
| ESR1 | ENSG00000215472 | 25 | 44 | - | 0.00087 | CACCAAGGGCTTGCTTTCCC |
| ESR1 | ENSG00000249755 | 224 | 243 | - | 0.00087 | AAGACAGGACATACTTCCAT |
| ESR1 | ENSG00000111837 | 42 | 61 | + | 0.00087 | AGCTCAGCCGACAGTCCCCC |
| ESR1 | ENSG00000119711 | 361 | 380 | + | 0.00088 | GTGCCAAGGCACCATGAAGC |
| ESR1 | ENSG00000147316 | 340 | 359 | - | 0.00088 | CGACGAGGGGTGCCGGTCCT |
| ESR1 | ENSG00000172534 | 115 | 134 | + | 0.00089 | CGGTGACGTCACCCGGCTCC |
| ESR1 | ENSG00000251357 | 358 | 377 | + | 0.00089 | CTGCAGGTTCAAACTGAGCA |
| ESR1 | ENSG00000140323 | 33 | 52 | - | 0.0009 | CCGCCAGGCCAGAGAGCCGC |
| ESR1 | ENSG00000228093 | 111 | 130 | + | 0.0009 | CCACCTGCTGATGCTGCCAT |
| ESR1 | ENSG00000258813 | 284 | 303 | - | 9.00E-04 | GAATTTTGCCATCCTGCCTC |
| ESR1 | ENSG00000234913 | 357 | 376 | - | 9.00E-04 | GGCGTGAGCCACCGCGCCCG |
| ESR1 | ENSG00000109475 | 166 | 185 | + | 9.00E-04 | TGCGCAATTCTATTTGACCT |
| ESR1 | ENSG00000222750 | 139 | 158 | + | 0.00091 | GATGGAGGAGGTTCTGCCCC |
| ESR1 | ENSG00000219433 | 142 | 161 | - | 0.00091 | GGCCAAGGCAAGCAACACCT |
| ESR1 | ENSG00000231792 | 3 | 22 | - | 0.00091 | GGCCTGAGCCGCAGTGCCCG |
| ESR1 | ENSG00000176532 | 358 | 377 | - | 0.00092 | GAGAGATGGGACCCTGGCTG |
| ESR1 | ENSG00000155962 | 280 | 299 | + | 0.00092 | GCTCCAGGTCTGACTCAGTC |
| ESR1 | ENSG00000224437 | 234 | 253 | - | 0.00092 | GAACAAGGCCACCCGCACTG |
| ESR1 | ENSG00000227973 | 281 | 300 | - | 0.00092 | CACCTTTGGGACCTTGAGCC |
| ESR1 | ENSG00000115204 | 191 | 210 | + | 0.00093 | CTGCAGACTCACAGTGACTA |
| ESR1 | ENSG00000105397 | 18 | 37 | + | 0.00093 | GGATCCGGCCGCGTTGCCCA |
| ESR1 | ENSG00000240758 | 121 | 140 | - | 0.00093 | GAGCAACGCCGCCCAGCCTC |
| ESR1 | ENSG00000213865 | 103 | 122 | - | 0.00093 | AAATGTGGTCTCTCAGACTC |
| ESR1 | ENSG00000214014 | 65 | 84 | + | 0.00094 | CTCTCATTTCCCCCAGACTT |
| ESR1 | ENSG00000139974 | 207 | 226 | - | 0.00095 | GGAGCGAGTCGACTTGATCT |
| ESR1 | ENSG00000164621 | 162 | 181 | + | 0.00095 | TGAGAAGGTTAATCTAACTT |
| ESR1 | ENSG00000113621 | 206 | 225 | - | 0.00095 | ACATCTGGCAACCCTAACCC |
| ESR1 | ENSG00000172209 | 225 | 244 | + | 0.00095 | GCGACAAGGTAGGCTGTCAT |
| ESR1 | ENSG00000127423 | 83 | 102 | + | 0.00096 | AGCGAAGGGGGCCAGGGCCT |
| ESR1 | ENSG00000163468 | 230 | 249 | - | 0.00096 | CTTCCAGATCACCTTCATAC |
| ESR1 | ENSG00000111644 | 197 | 216 | + | 0.00096 | GCCCGCGTGCGCCCCGCCCC |
| ESR1 | ENSG00000187514 | 275 | 294 | + | 0.00097 | GCGCAAGCTCTGCTGGACTT |
| ESR1 | ENSG00000253174 | 36 | 55 | - | 0.00097 | AAGACAGCTCTTCCTGAGAC |
| ESR1 | ENSG00000130222 | 297 | 316 | + | 0.00097 | GGTTGATCGCACTATGACTC |
| ESR1 | ENSG00000168769 | 363 | 382 | + | 0.00097 | GAGCGCGGGCAACGGGATCT |

| | | | | | | |
|---|---|---|---|---|---|---|
| ESR1 | ENSG00000005007 | 278 | 297 | - | 0.00098 | CGCCGCTGCCGCCGAGCCCC |
| ESR1 | ENSG00000236830 | 49 | 68 | - | 0.00098 | AGCCCAGCCCGTCGAGGCCC |
| ESR1 | ENSG00000233830 | 359 | 378 | - | 0.00098 | CATCTATGTCGCCCCGAACC |
| ESR1 | ENSG00000172375 | 219 | 238 | - | 0.00098 | GGGCCCTTTAAGCTTGACCT |
| ESR1 | ENSG00000177721 | 372 | 391 | + | 0.00098 | GGCCCAGGCCGGTCTCAGCA |
| ESR1 | ENSG00000253518 | 42 | 61 | + | 0.00098 | TGCCAATACCACATTGCCTT |
| ESR1 | ENSG00000257446 | 203 | 222 | + | 0.00099 | GCGCTCGGGGGTTCTGTCCT |
| ESR1 | ENSG00000164924 | 41 | 60 | + | 0.00099 | TTTGCAGGTCATCCAAGCAC |
| ESR1 | ENSG00000171863 | 196 | 215 | + | 0.001 | TTCCTGGGACTCTCTAGCCT |
